# Summer weather conditions influence winter survival of honey bees (*Apis mellifera*) in the northeastern United States

**DOI:** 10.1038/s41598-021-81051-8

**Published:** 2021-01-15

**Authors:** Martina Calovi, Christina M. Grozinger, Douglas A. Miller, Sarah C. Goslee

**Affiliations:** 1 Department of Ecosystem Science and Management, The Pennsylvania State University, University Park, PA 16802 USA; 2 Department of Entomology, Center for Pollinator Research, Huck Institutes of the Life Sciences, The Pennsylvania University, University Park, PA 16802 USA; 3 Department of Geography, The Pennsylvania State University, University Park, PA 16802 USA; 4 Department of Ecosystem Science and Management, The Pennsylvania State University, University Park, PA 16802 USA; 5 USDA-ARS Pasture Systems and Watershed Management Research Unit, University Park, PA 16802 USA

**Keywords:** Ecology, Ecosystem ecology

## Abstract

Honey bees are crucial pollinators for agricultural and natural ecosystems, but are experiencing heavy mortality in North America and Europe due to a complex suite of factors. Understanding the relative importance of each factor would enable beekeepers to make more informed decisions and improve assessment of local and regional habitat suitability. We used 3 years of Pennsylvania beekeepers’ survey data to assess the importance of weather, topography, land use, and management factors on overwintering mortality at both apiary and colony levels, and to predict survival given current weather conditions and projected climate changes. Random Forest, a tree-based machine learning approach suited to describing complex nonlinear relationships among factors, was used. A Random Forest model predicted overwintering survival with 73.3% accuracy for colonies and 65.7% for apiaries where Varroa mite populations were managed. Growing degree days and precipitation of the warmest quarter of the preceding year were the most important predictors at both levels. A weather-only model was used to predict colony survival probability, and to create a composite map of survival for 1981–2019. Although 3 years data were likely not enough to adequately capture the range of possible climatic conditions, the model performed well within its constraints.

## Introduction

Honey bees (*Apis mellifera*) contribute more than $20 billion in pollination services to agriculture in the United States^1^, and contribute substantial economic value to downstream industrial sectors^2^. Honey production generates an additional $300 million annually for US beekeepers^3^. However, winter colony mortality has a strong negative effect on economic and ecosystem potentials, with approximately 53.5% overwintering mortality of US honey bee colonies estimated from survey data from 2016 to 2019^4^. However, winter mortality is known to vary regionally in both the US and Europe, but the landscape or weather factors underlying this variation are poorly understood^5,6^.

Honey bee colonies are not dormant during the winter: they remain active and maintain the hive temperature between 24 and 34 °C by forming a thermoregulating cluster^7^. This enables them to survive long periods of cold temperatures^8–10^. During the winter, the colony ceases foraging for nectar and pollen and relies on its existing stores, collected during the plant growing season. Furthermore, brood rearing ceases, and the colony is dependent on the survival of a long-lived cohort of bees that is produced in the autumn. These bees will live for several months, while worker bees produced in the summer only live for a few weeks. Thus, factors which undermine the ability of the bees to collect and store adequate amounts of food during the summer and fall, or to thermoregulate effectively during the winter, or reduce the lifespan of winter bees, can contribute to colony mortality. These factors include: beekeeper management practices that affect parasite and pathogen loads, particularly control of *Varroa* mites^11–13^; forage quality and pesticide exposure due to the surrounding land use^14^; and weather factors which influence the availability of forage, the thermoregulatory ability of the bees in the winter, and the amount of time before bees are able to initiate brood rearing in the spring^15^. Modeling and predicting honey bee winter survival requires consideration of all of these factors.

Previous studies have evaluated how colony growth, honey production and survival correlates with particular land use practices, such as the percentage of agricultural land or the percentage of certain crops in the area surrounding the hive^16–19^. However, while several studies have indicated that honey bees show reduced growth or higher mortality with increasing urban or agricultural land use^16,17,20^, others have found that agricultural land use is positively correlated with colony survival^18^. These measures of land use do not necessarily correlate directly with forage quality, as bees can collect substantial resources from wildflowers in both agricultural and urban areas, and crops can vary greatly in the resources they provide to bees or their pesticide regimes^21–24^. Indices of forage quality and of pesticide loading based on surrounding land cover have been developed that are intended to incorporate specific effects of crop and habitat types on a broad scale^25–27^, but thus far these have not been applied to studies of honey bee winter survival or health.

Seasonal weather conditions affect both forage availability and thermoregulatory success, and thereby directly and indirectly influence honey bee health^28^. During the growing season, weather conditions can affect the onset and decline of specific foraging resources, lengthen or shorten the time in which resources are available for bees, change the quality of these resources, and alter the span during which bees can actively forage^29,30^. Indeed, even small variations in temperature can dramatically change the numbers of available flowers and the amount of nectar they produce^31^. Winter temperature conditions influence the efficiency of maintaining hive internal temperatures. The optimal external temperatures that maximize efficiency of this thermoregulation are from − 5° to 10 °C^32^. When temperatures drop below 10 °C, the bees form a thermoregulating cluster^8,10^. In previous studies in Austria, warmer and drier climates have been associated with higher winter losses^15^.

Few studies have simultaneously evaluated the effects of multiple landscape and weather factors on honey bee colony winter survival. A study of honey bee winter survival in the Netherlands evaluated survival of 1106 colonies across 2 years, using 24 variables in a generalized linear mixed model^14^. Overall, there were positive effects of forest and grassland, and negative effects of increased annual mean temperature, and no effect of predicted toxicity of insecticides applied to agricultural areas. Another study was conducted in Belgium, where the apiary-level winter colony survival rates were assessed for 147 apiaries across two years (encompassing 607 colonies) using 26 variables using regression analyses^33^. In that study, *Varroa* infestation was by far the most correlated variable with winter mortality rates, followed by temperature conditions (in terms of frost days and flying hours), beekeeper practice (e.g., involvement in beekeeping organizations), potential pesticide exposure (calculated from surrounding agricultural lands), and landscape connectivity.

Evaluating how these complex factors influence honey bee winter survival requires large data sets that span multiple types of habitats, microclimates, and years. Collaborations with beekeepers through citizen science projects can provide the necessary large and varied data sets, and are becoming increasingly important for studying both managed honey bee and wild bee health^33–35^. The voluntary involvement of beekeepers as key collaborators in the collection of data has the dual benefits of generating the necessary large data sets while also directly engaging stakeholders in scientific research, such that the outcomes are more likely to be translated to directly benefiting the stakeholders^35^. One example of this citizen science approach is the annual winter loss survey conducted by the Pennsylvania State Beekeepers Association (PSBA) across the entire state. Voluntary survey data entails certain limitations, such as effective coverage of target populations, data accuracy, potential bias of the survey sample, the survey modes, missing data, and incomplete responses^36^, but nonetheless the PSBA survey provides the best available information, a geographically robust data set covering hundreds of apiaries and 3 years.

Using this unique data set, our objective was to develop a predictive model of the overwintering survival of honey bee colonies in Pennsylvania that incorporates weather, topographic variables that affect temperature and moisture, and the composition of the surrounding landscape as it determines foraging resources and potential pesticide load^25–27^ (Supplementary Table S2). Our goal was to understand relative contribution of these factors to honey bee winter losses in this region, and to develop a model predicting honey bee winter survival across Pennsylvania to support beekeeper management decisions and processes.

## Methods

The complex nature of the factors influencing overwintering survival of European honey bees necessitated the integration of multiple datasets comprising weather and topographic variables that determine temperature and moisture conditions, and landscape variables that determine the availability of foraging resources and insecticide exposure risk. The analysis of these diverse datasets can best be addressed with randomization-based machine learning techniques that do not require the data to meet standard statistical assumptions or expect relationships between overwintering success and environment to follow any predetermined form. Thus, unlike previous studies which examined landscape and/or weather condition effects on winter survival using general linear mixed models or regression approaches^14,32^, we used a machine learning approach suitable for complex multivariate datasets.

### Data sources

#### Honey bee overwintering survival

Our main dataset originates from the Pennsylvania State Beekeepers Association Winter Loss Survey (see sample questions in the Supplementary S1). The survey began to collect apiary locations information in 2017, resulting in data for three winters in our analysis: 2016–2017, 2017–2018, and 2018–2019. Variables extracted from the survey were: beekeeper ID, spring year, colony number in November and in April, beekeepers’ years of experience, use of *Varroa* mite treatment. The ID that identifies each beekeeper is randomly assigned each year to protect personally identifiable information. Unfortunately, as a result it is not possible to follow individual apiaries throughout the years of the survey, making historical tracking of the individual beekeepers and their apiaries impossible. Furthermore, detailed information is not available on the genetic background of the colonies, whether the colonies were newly established or persisted over the previous winter, the age or quality of the queens, or how and when the colonies were established (e.g., from packages or splits).

**Figure 1:**
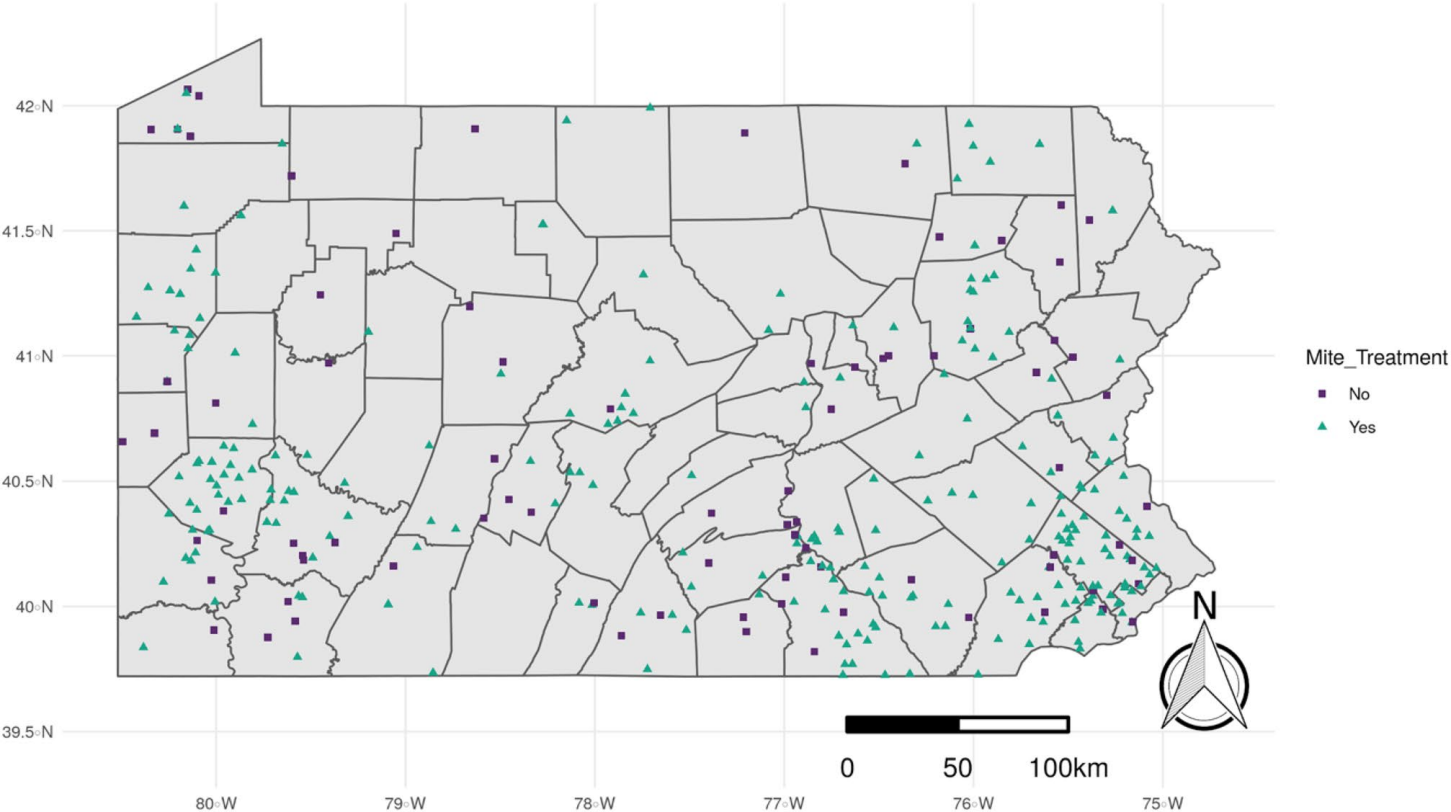
Locations of Pennsylvania beekeeper survey respondents from 2016 to 2019, stratified by use of treatment for *Varroa* mites (257 treated and 85 untreated apiaries). Only treated apiaries were modeled. The map has been generated by the authors in R 3.6.2^50^, using the sf^72^, the raster^56^, the ggplot2^73^ and the cowplot^74^ packages.

In the survey, beekeepers noted whether their operations were involved in migratory beekeeping practices (meaning they moved colonies to different sites for pollination services throughout the year). These operations were not used in this study. Beekeepers could only provide information on their total colony numbers and location information for one apiary: thus, it is possible that some beekeepers combined information from multiple apiaries and this is a limitation of this data set. However, most of the non-migratory respondents reported fewer than ten hives: this suggests they were likely small-scale beekeepers with a single apiary, or a few nearby apiaries. After filtering, 342 apiaries with 1726 colonies had adequate data (Fig. 1).

**Figure 2:**
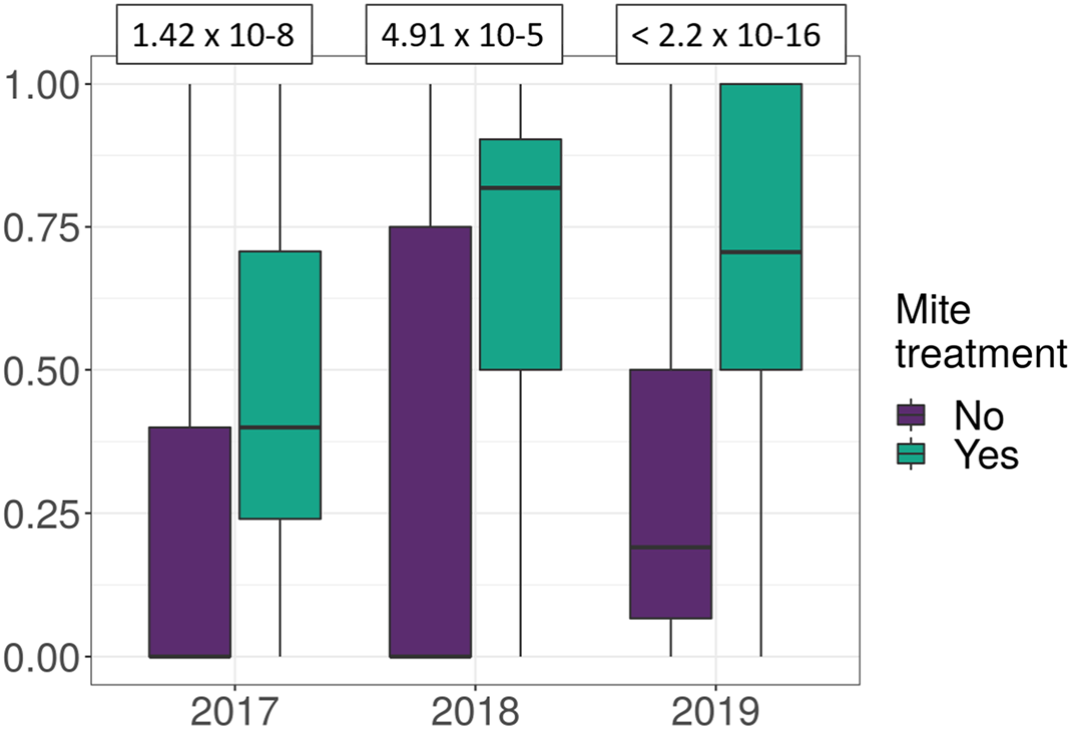
Survival of mite-treated and untreated honey bee colonies by year. In each of the three years, 80 out of 375 (21%), 25 out of 377 (7%) and 192 out of 974 (20%) colonies were untreated (297 out of 1,726, or 17% overall). In the white boxes the p-values results of the one-sided t-test for each year. Evidence rejects H0 in favor of H1: the average survival is higher when the colonies are treated.

Preliminary analysis of the dataset clearly demonstrated that treating for *Varroa* mites was a key factor in determining overwintering survival across all three years (Fig. 2). For each of the three winter years, difference in the survival of mite-treated and untreated honey bee colonies was evaluated using a one-sided t-test. For each of the 3 years the evidence is to reject the null hypothesis that means were equal; when the colonies are treated the average survival is higher (see Fig. 2). Only 17% of beekeepers did not treat their colony in some way. Because of the clear effect of the treatment and the small number of untreated colonies, we chose to model only the treated apiaries. Thus, the final dataset comprised 1429 colonies within 257 apiaries.

#### Weather and topographic variables

For each reported apiary location, we generated annual and seasonal weather variables from 4-km gridded daily temperature and precipitation data^37^. The variables created include standard bioclimatic and agronomic indices such as BIOCLIM^38^ that have been used in previous modeling studies (e.g., Ref.^39^). Agronomic indices include consecutive dry days and growing degree days, a measure of heat accumulation over a base temperature of 5 °C. Only the variables most relevant for honey bees (rather than other taxa) have been included in the study (Table 1). Bee-specific weather indices relevant for overwintering include winter days within bee optimal thermoregulating temperature range (− 5° to 10 °C) and the number of winter days suitable for flight and foraging, with maximum temperature above 16 °C and total precipitation below 3 mm (Table 1)^9,38^. Note that while there is little blooming in the winter months (December, January, February, in our analysis) some plants in Pennsylvania do bloom during these times (such as witch hazel), and bees in the northeastern United States begin brood rearing in late February^9^. Furthermore, bees take cleansing flight when the weather permits to void their intestines and prevent pathogen and parasite transmission in the colony^40^. Topographic variables were included in the analysis because they modify the local climate at finer scales than can be represented by the gridded climate data^39^. These were calculated from 30-m resolution gridded elevation data^41^ using GRASS GIS^42^, and include slope, curvature, and the northerly and westerly components of aspect.

**Table 1:** Weather and topographic variables hypothesized to affect honey bee overwintering survival.

Variable description	Unit	Variable importance
Weather		
BEE1: Winter minimum temperature	°C	0.0151
BEE2: Winter total precipitation	mm	0.0164
BEE3: Winter days within the bee-optimal temperature range − 5 °C to + 10 °C	D	0.0111
BEE4: Winter days with maximum temperature above 16 °C and precipitation below 3 mm	D	0.0119
BEE5: Winter minimum temperature variation	°C	0.0118
BEE6: Autumn total precipitation	mm	0.0128
Growing degree days (base 5 C)	°C	**0.0252**
Days between rain events > 0.25 mm	mm	0.0127
BIOCLIM 2: Mean diurnal temperature range	°C	0.0151
BIOCLIM 3: Temperature isothermality		0.0196
BIOCLIM 4: Temperature seasonality	°C	0.0132
BIOCLIM 5: Maximum temperature of warmest month	°C	**0.0201**
BIOCLIM 6: Minimum temperature of coldest month	°C	0.0131
BIOCLIM 7: Temperature annual range	°C	0.0130
BIOCLIM 8: Mean temperature of wettest quarter	°C	0.0140
BIOCLIM 9: Mean temperature of driest quarter	°C	0.0147
BIOCLIM 12: Annual precipitation	mm	0.0148
BIOCLIM 16: Precipitation of wettest quarter	mm	**0.0202**
BIOCLIM 17: Precipitation of driest quarter	mm	0.0122
BIOCLIM 18: Precipitation of warmest quarter	mm	**0.0213**
BIOCLIM 19: Precipitation of coldest quarter	mm	0.0157
Topography		
Elevation	m	0.0154
Slope		0.0126
Potential incident solar radiation, 21 Dec	Wh × m − 2 × d − 1	0.0156
Profile curvature	m − 1	0.0111
Terrain curvature	m − 1	0.0110
Topographic wetness index		0.0116
East/West orientation of slope		0.0102
North/South orientation of slope		0.0103
Landscape		
Distance-weighted Insect Toxic Load		0.0112
Distance-weighted Forage Quality autumn		0.0104
Management		
Beekeeper years of experience		0.0040
Number of colonies in November		0.0057

Weather variables include both BIOCLIM^38^ and agronomic indices, as well as bee-specific variables developed for this study (BEE#). Permutation variable importance values are from the full colony model; larger values are more influential. The most important four variables are bold. Autumn: September, October, November. Winter: December, January, February.

#### Forage resource index and insecticide toxic load

Two honey bee-specific distance-weighted landscape descriptors were generated for each apiary using the 30 m resolution USDA-NASS Cropland Data Layer (CDL)^43^. The Forage Resource Index (FRI was calculated for each floral season. This index describes the quality and abundance of floral resources available for both managed and wild bees for each land use category^25,26,44^. Following Koh et al. (2016), we generated the seasonal FRI at each apiary location using a weighted distance decay function that extends to the 5-km foraging radius of honey bees^45^. The Insect Toxic Load (ITL) characterized the amount of active ingredient used for each insecticide based on statewide records of per-hectare use by crop type, and converted this to an aggregated insect toxic load using honey bee LD50s^27^. The same CDL data and distance-weighting function were used for the FRI and ITL to maintain consistency. Although CDL is an annual product, these indices integrate over large areas and are extremely highly correlated between years, so for simplicity we only used 2017 indices in the model.

### Statistical analyses

The survey data were extremely unbalanced, with 1429 colonies within 257 apiaries. The apiaries contained from 1 to 34 colonies, with a median value of 3. Our objective was to predict survival at the colony level, but we analyzed the data at both apiary and colony scales to ensure that results were consistent at both levels. We used a binary classification to model survival at the colony scale: 0 for mortality, and 1 for survival. Thus, if an apiary had 5 colonies in November and 3 of those colonies survived in April, each of the 3 surviving colonies was assigned a score of 1, while each of the 2 dead colonies was assigned a score of 0. To provide comparable results, and because of the highly unbalanced dataset, we modeled survival at the apiary scale as a binary variable as well. Thus, if any colonies in an apiary died, the apiary was assigned a score of 0. If no colonies in an apiary died, the apiary was assigned a score of 1. A tenfold cross-validation across cutoff values from 50 to 100% showed that the highest accuracy was obtained by modelling the survival with this 100% cutoff.

A probability Random Forest (RF), a flexible tree-based machine learning approach, was used to analyze overwintering mortality in relation to environmental and landscape factors within the 1429 colonies that had been treated for Varroa mites. Random Forests develop a large number of decision trees using a random sampling of variables, then average across all trees to produce an ensemble (forest) fit^39,46^. The RF technique is very efficient when working with datasets comprising a large number of predictors^47^, and when the relationships among variables are nonlinear or complex, because it is a flexible distribution-free method^48^. Given the complexity and nonlinearity of the dataset used in this study, RF was preferred to a linear regression method, and allowed the development of a reliable empirical model without prior knowledge of the relationship between the survival and the predictors^49^. Importantly for a study of this scope, RF models are robust to correlated predictors.

All analyses were conducted in R 3.6.2^50^, using the ranger package 0.11.2^51^ for RF models of survival probability and permutation-based variable importance, and the caret package 6.0-84^52^ for model evaluation. Variable importance was calculated using the permutation-based method in the ranger package, which indicates the prediction accuracy lost if that variable is omitted^53,54^. The form of the relationship between survival probability and the major independent variables was assessed using partial dependence plots (pdp 0.7.0 package^55^). All maps presented in this manuscript were produced with the raster (3.0-12^56^) and sp (1.4-0^57^) packages.

Our initial intent was to train the model with the first 2 years of data and test it on the third, but the weather was very different across the 3 years: 2016 was warm and dry, 2017 was warm and wet, and 2018 was very wet. Instead, to ensure that the data used to train the model spanned the greatest possible range of weather conditions, we used cross-validation stratified by year to train and evaluate the model. Ten repetitions of a tenfold cross-validation were used to tune the model on a gridded parameter search with the number of trees between 2000 and 5000 on an increment of 500, and number of variables per tree (mtry) from 3 to 8. For both colony and apiary models, the best number of variables was 3, and best number of trees was 4000 and 4500 respectively; mtry was more influential than number of trees. An independent set of ten repetitions of a tenfold cross-validation using the tuned parameters was used to obtain the error estimates. The final model was fitted on the full dataset, in order to obtain the most reliable estimates of variable importance and the best model for prediction. Such a model overestimates accuracy, so cross-validation error estimates are given. These estimates show how the model is likely to perform when presented with new data. The same cross-validation and analysis methods were used at both the apiary and colony scales.

### Ethics declaration

No humans or honey bees have been directly used.

## Results

**Figure 3:**
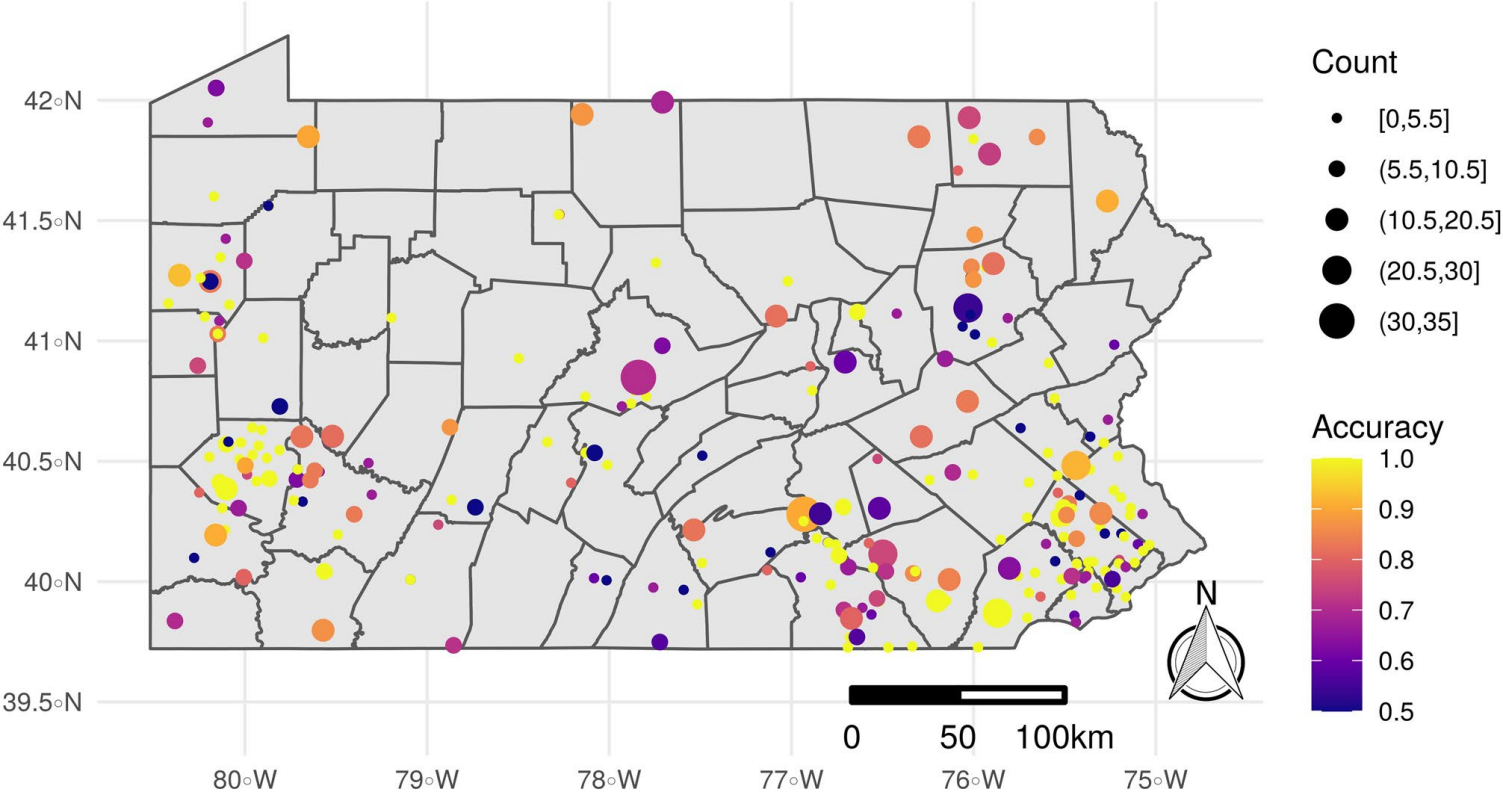
Prediction accuracy of the Random Forest model of overwintering survival probability from the colony model, averaged by apiary for mapping purposes. The dataset used to generate the accuracy map contained 1429 colonies within 257 apiaries. The color indicates the mean overall model accuracy at that apiary, and the circle size is proportional to the number of colonies in November. The map has been generated by the authors in R 3.6.2^50^, using the sf^72^, the raster^56^, the ggplot2^73^ and the cowplot^74^ packages.

As described in the methods, we created models using data both at the apiary and individual colony level. The apiary model had cross-validated out-of-bag (OOB) error of 22% on the training data set, and a prediction accuracy of 65.7% on the test data (95% confidence interval 59.6–76.15%); the colony model had an OOB error of 19% and prediction accuracy of 73.3% (95% confidence interval 70.9–75.5%). More detailed assessments of model performance and variable importance are only presented for the colony model, since variable importance was similar for both models. Model accuracy was not notably related to geography within Pennsylvania (Fig. 3).

The four most important variables were: growing degree days, maximum temperature of the warmest quarter, precipitation of the warmest and of the wettest quarter (Table 1). Based on the sorted variable importance for all four models, growing degree days in the prior summer was the strongest predictor of overwintering survival; this agronomic index of heat accumulation may relate to floral resource availability. Landscape (FRI and ITL) and topographic factors did not contribute substantially to the colony survival model. The beekeeper’s years of experience had no relationship to colony survival, though it was identified as important in other studies^58^. The colony level model was nearly twice as likely to predict that colonies survived when they died than that they died when they actually survived (Table 2), suggesting that there is an additional source of mortality we have not considered.

**Table 2:** Confusion matrix for model predictions of colony-level honey bee overwintering survival for the full Random Forest model.

Full model	Actual	
	Mortality	Survival
**Predicted**		
Mortality	337 (24%)	87 (6%)
Survival	192 (13%)	813 (57%)

The four most influential variables were further evaluated using partial dependence plots. These plots help understand how the variable affects the prediction, more specifically they show the dependence pattern between the probability of survival, and the variable investigated^59,60^, independent of all other variables in the model. This kind of visualization is able to provide a powerful interpretation on how the variables affect the probability of survival^61^.

**Figure 4:**
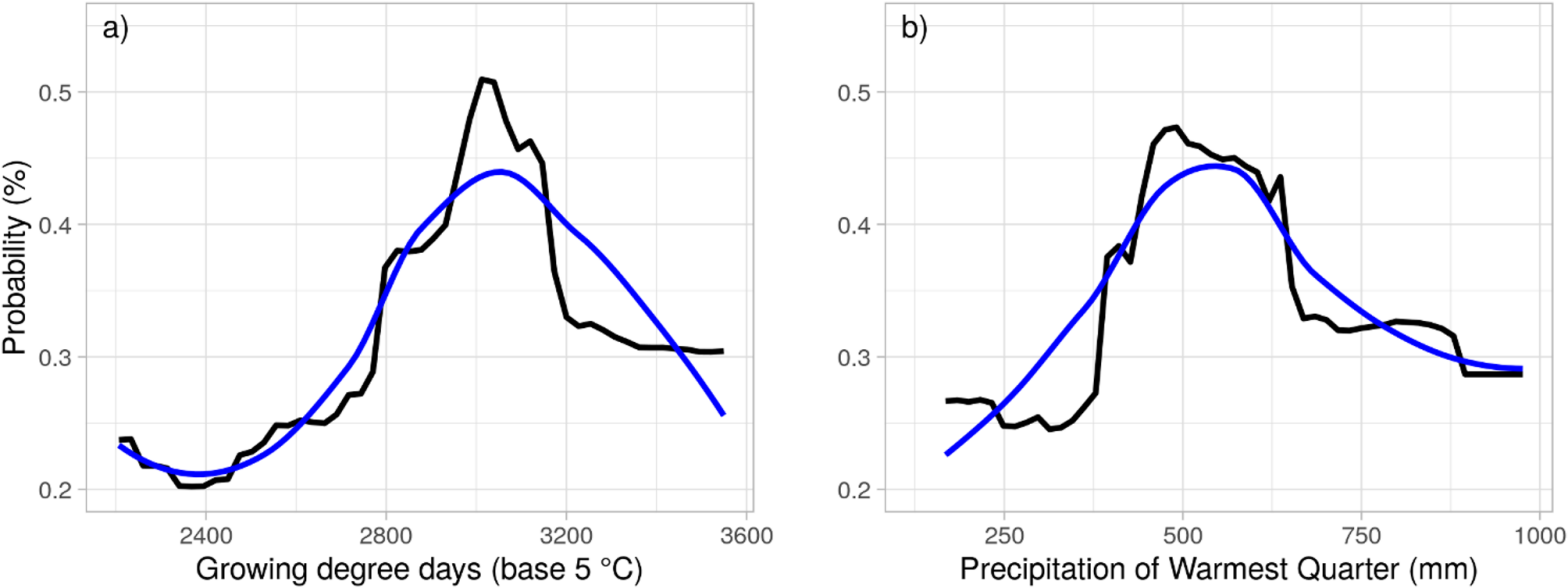
Partial dependence plot for the two variables that most explain overwintering survival in the prediction analysis at the apiary level. Plot (**a**) describes the relationship between the growing degree days (along the x axis) and the probability of overwintering survival (y axis), given all the other variables in the model. Plot (**b**) describes the relationship between the precipitation during the warmest quarter (along the x axis) and the probability of overwintering survival (y axis), given all the other variables in the model. In both the plots, the black line represents the modeled relationship between survival and the variables, while the blue line shows a spline-smoothed fit.

The partial dependence plot for growing degree days (Fig. 4a) shows a unimodal pattern: survival was highest at intermediate values. Maximum temperature of the warmest quarter also had a high importance value and showed a similar partial dependence pattern to growing degree days and thus is not shown. Both precipitation of the warmest quarter and of the wettest quarter were important, but the partial dependence plot is only presented for precipitation of the warmest quarter as this variable was more important, and the other was very similar (Fig. 4b). Precipitation also showed a unimodal relationship with survival; neither too dry nor too wet resulted in greatest survival.

**Figure 5:**
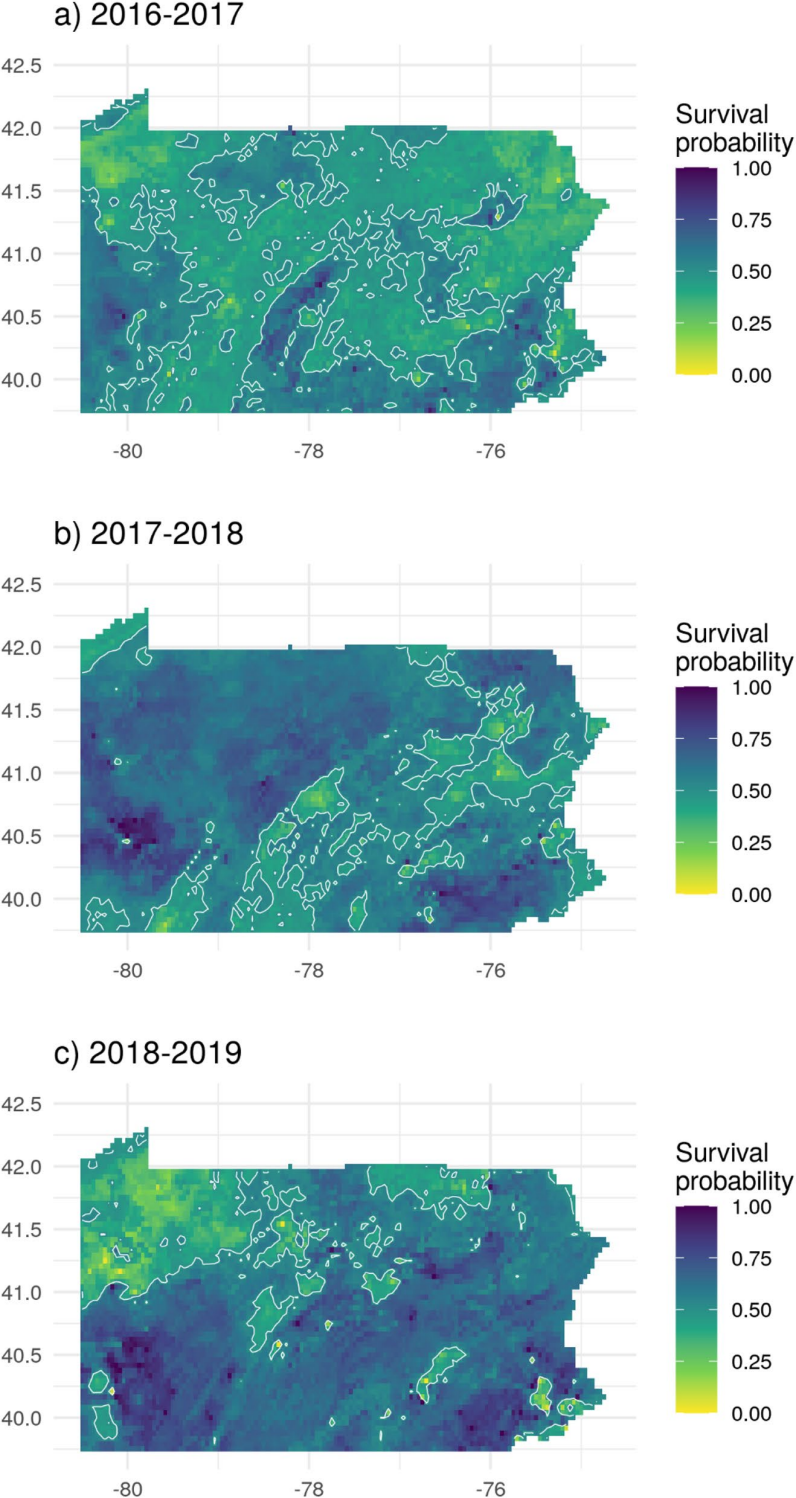
Weather-based prediction maps of the probability of honey bee colony survival from the weather-only colony model for the most recent 3 years of PRISM data. Contour lines show the 0.5 probability level. The three maps show the survival probability for Pennsylvania, based on the results of the weather-based model. The map (**a**) represents results from year 2016, which had a mean annual temperature of 10.4 °C and a mean annual precipitation of 1007 mm across the state. Map (**b**) represents results from year 2017, which had a mean temperature of 10.2 °C and a mean annual precipitation of 1205 mm. Map (**c**) represents results from year 2018, which had a mean annual temperature of 9.7 °C and mean annual precipitation of 1653 mm. For the whole period of record, from 1981 to 2018, the mean annual temperature was 9.4 °C, with a mean annual precipitation of 1082 mm. The maps have been generated by the authors in R 3.6.2^50^, using the sf^72^, the raster^56^, the ggplot2^73^ and the cowplo^74^ packages.

**Figure 6:**
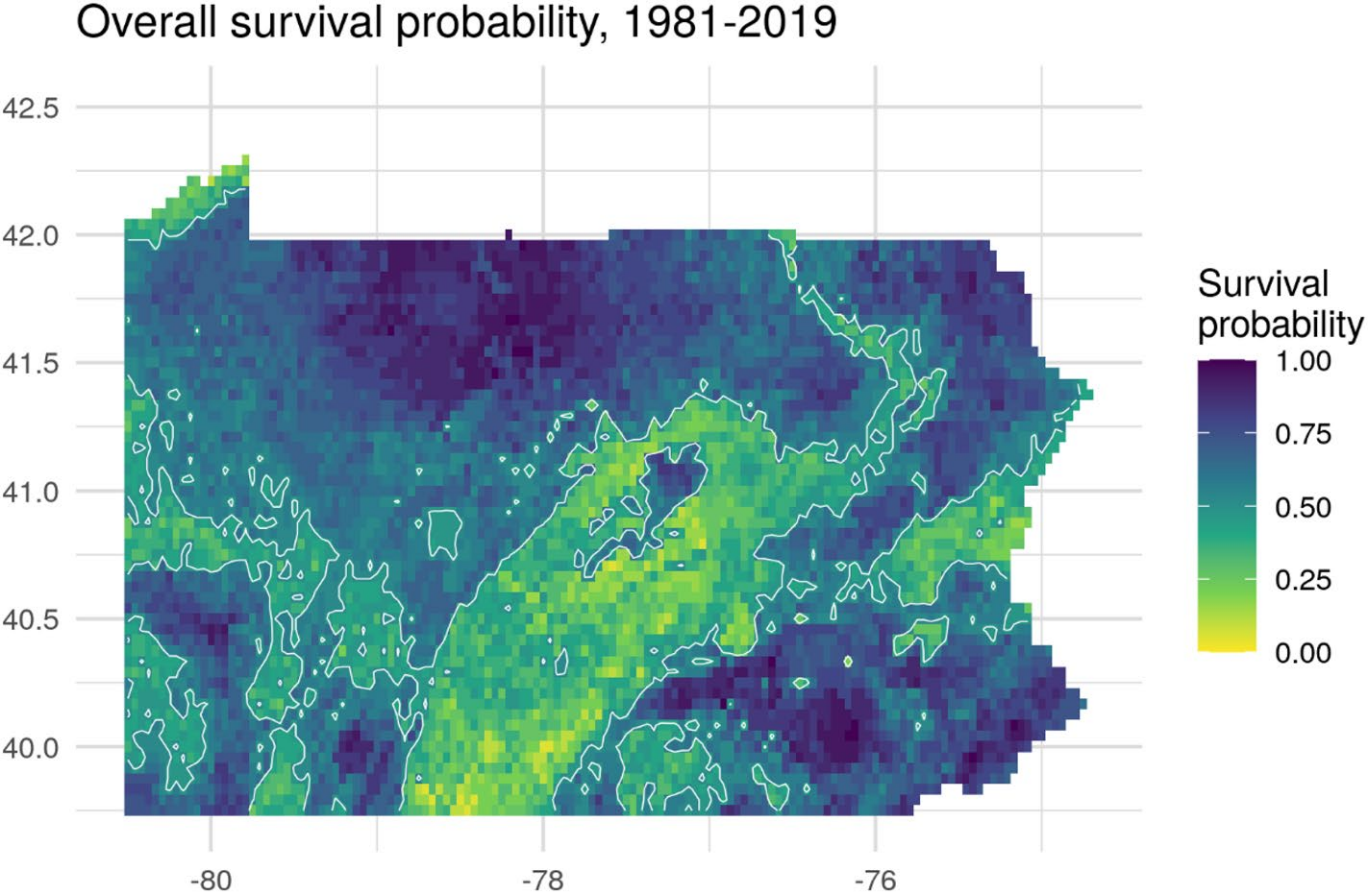
Mean probability of colony survival for 1981–2019 from the weather-only colony model. Contour lines show the 0.5 probability level. The map has been generated by the authors in R 3.6.2^50^, using the sf^72^, the raster^56^, the ggplot2^73^ and the cowplot^74^ packages.

Because the climatic variables were the most important overall, we also modeled colony survival using only weather variables, generating a prediction for each of the three winter years (Fig. 5) and over the PRISM period of record, 1981–2019 (Fig. 6). The weather-only model performed about as well as the full model, with an OOB error of 19% and 73% prediction accuracy (95% confidence interval 70.6–75.2%).

No part of Pennsylvania was always good or always bad for honey bee survival; there was substantial spatial and temporal variability. The maps of predicted honey bee survival for the three winters studied showed considerable variability, both between years and across the state (Fig. 5). Winter 2016–2017 showed a predicted mean survival of 49.2% (range 5–97.6%); 2017–2018 had a predicted mean survival of 59.2%, (range 9.8–100%); and 2018–2019 winter had a mean predicted survival of 59.7% (range 17–100%). Mean predicted long-term survival probability across Pennsylvania based on weather data from 1981 to 2019 (Fig. 6) was 59.5% (range 5.3–100%). The mean is consistent with values reported by the Bee Informed Team (53.5%)^4^.

## Discussion

In colonies where beekeepers controlled for *Varroa* mite populations, weather factors, particularly summer temperatures and precipitation for the prior year, were the strongest predictors of overwintering survival in European honey bee colonies in Pennsylvania in our data set. Topographic factors and landscape quality factors (forage and insecticide toxic load) were not important, contrary to expectations.

In our initial analysis, we found that beekeepers who used management practices to control *Varroa* mite levels overall had higher winter survival. Winter mortality of honey bee colonies has been strongly correlated with uncontrolled *Varroa* mite populations in multiple studies^11,12^. Parasitized, virus-infected bees have reduced nutritional stores and a reduced lifespa^62^. Thus, high levels of *Varroa* reduce the probability of winter survival^32^. In our data set, we found the majority of responding beekeepers did use management practices to control *Varroa* mite populations (83%), and thus we focused on these beekeepers for the rest of the analysis. However, approximately 70% of new beekeepers (< 1 year of experience) treated for *Varroa* mites, while 77–89% of the beekeepers in the other categories treated for *Varroa* (data not shown): thus, encouragement of new beekeepers to implement *Varroa* management strategies could be beneficial.

There are several explanations as to why the landscape quality factors included in our analysis were not significant predictors of winter survival. The foraging index is based on expert opinion, and the insecticide toxic load index does not account for variation in local crop management practices or exposure rates of bees^25–27,44,45^; thus, there is clearly room for improvement in methods for assessing the suitability of surrounding land use for pollinator use. Moreover, supplementary feeding from the beekeeper (which was not included in the survey data) may have mitigated impacts of floral resource availability, while insecticide exposure can have complex effects on bees which may not be captured by winter survival rates^24^.

It is important to note that there are many factors that can contribute to winter survival which were not possible to assess, due to limited information about the colonies used in this analysis. In Europe, for example, the genotype of the colony influences its survival rates, and colonies from local stock perform better^63^. In our previous studies, however, we did not find an effect of colony genotype on winter survival in Pennsylvania: rather, colony size was a major factor^64^. Colony size can be influenced by the surrounding landscape conditions, but may also be influenced by beekeeper practices and the origin of the colony—for example, if a colony is initiated earlier in the growing season, it reaches a larger size in the fall and is more likely to survive the winter^9^. Queen age and quality can also influence winter survival^11^ Moreover, the methods used for controlling *Varroa* populations, the timing of application and the conditions of application (including weather conditions) can influence winter survival^65^. Even the relative distribution of the colonies within the apiary can influence survival, likely by influencing disease dynamics: colonies in “low density” apiaries had higher winter survival than colonies in “high density” apiaries^66^. Finally, the survey did not include information about levels of or evidence for parasites or pathogens, and thus we could not evaluate whether these parameters correlated with survival. While citizen science data is a powerful tool to generate the necessary large and varied data sets needed for studies of the effects of landscape and weather conditions on bee health, there are limitations in the extent of the data that can be collected^36^. Future surveys can seek to collect additional information to address these issues.

Despite the limitations in the data set, the importance of weather conditions in predicting winter bee survival are quite clear from our analysis, and consistent with results from previous studies in other countries. In Austria, Switanek et al.^15^ found that hot, dry summers reduced overwintering survival. Similarly, studies in the Netherlands found reduced survival with increased annual mean temperatures^14^. In colonies in Belgium, more frost free days were associated with positive survival outcomes, while more flying days were associated with negative outcomes^33^. Our approach allowed for a more nuanced analysis of climatic variables, and we found adverse effects of both too-cool and too-hot summers. This could be the result of effects on plant flowering patterns (flowering could be reduced in both cool and hot conditions), which could negatively effect colony growth. Periods of drought can dramatically decrease weight gain in colonies in the summer^67^. Alternatively, mismatches between colony behavior (in terms of timing of brood rearing, which is triggered by temperature conditions) and local flowering patterns can also influence colony growth, by reducing nectar collection and honey production^68^. Smaller colonies are less likely to survive the winter^64^. Additionally, altered colony behavior as a result of environmental conditions can result in increased disease levels. In a study in Germany, bee colonies relocated to warmer or cooler regions exhibit differences in brood rearing, and colonies with longer period of brood rearing had higher levels of *Varroa*^68^. Thus, longer summers could result in high *Varroa* levels in the fall, which could negatively affect winter survival. Evaluation of colony growth patterns throughout the season using automated hive scales may provide insights into how weather conditions during the summer are influencing fall colony size and winter mortality^21,69^.

Interestingly, topographical features were not important variables in predicting bee winter survival. While topographical features should influence the microclimate surrounding the colony, it is possible that the foraging range of honey bees (which can span several kilometers from the colony^70^) reduce any observed influence of microclimate on forage or habitat conditions. Additionally, colony thermoregulatory behavior may have mitigated the effects of microclimate^8,10^.

The 3 years of data that we had available undoubtedly does not represent the full range of weather conditions possible in Pennsylvania. Thus, as additional years of data become available and are included in the model, the ability to predict outcomes will be improved. Nonetheless, this model worked well in the 3 years for which it was developed, with an acceptable accuracy given the extreme variability within the dataset, and the multitude of factors that affect overwintering survival.

Because the important variables were all weather-related, we were able to develop a predictive RF model created without landscape or topographic variables that was equally as accurate as the full model. Doing so reduced the data needs for the predictive model, and simplified analysis and mapping. Because it does not rely on landscape or management factors, this model can be used to characterize changes in overwintering survival with the changing climate independent of other factors. With slight modifications to use current data, this model has been used to develop a real-time tool to predict honey bee survival probability as a function of GDD^71^. The tool (BeeWinterWise) has been incorporated into the Beescape decision support system (https://beescape.org/), used by beekeepers and technical advisors. As apparent from Fig. 5, there is substantial variation across different regions of Pennsylvania and among years, and thus it is critical to develop site-specific decision support tools.

The presented model can be used to predict the probability of overwintering success, both for the current year and as a function of projected future climate change scenarios. The modeling framework used allows for the quantification of variable importance. Thus, modeling results can be used to develop decision support tools for overwintering survival, to better understand the roles of weather and landscape on honey bee success, and to characterize the effects of climate change on honey bee survival in the future. To the best of our knowledge this is the first study on honey bee overwintering survival that combines weather, topography, and derived land use factors. Our results, within the study limitations presented above, demonstrate both the predictive power of weather variables on analyses of honey bee overwintering survival, and the efficacy of addressing this type of question with machine learning methods such as Random Forest that are capable of identifying complex nonlinear relationships with correlated predictors.

## Supplementary Information

Supplementary Information.

## Data Availability

The anonymized survey data with corresponding weather and topographic data (geographic information is missing for privacy concerns) are being submitted as Supplementary Material (Supplementary Table S2). The columns’ names in Supplementary Table S2 correspond to the variables described in Table 1. Spring year and binary survival variables have been included in Supplementary Table S2.

## Abbreviations

USDA-NASS: United States Department of Agriculture—National Agricultural Statistics Service
CDL: Cropland data layer
FRI: Forage resource index
ITL: Insect toxic load
RF: Random forest
OOB: Out-of-bag
