# Supplementary material for: Summer weather conditions influence winter survival of honey bees (Apis mellifera) in the northeastern United States
Source: Sci Rep. 2021 Jan 15;11:1553. doi: 10.1038/s41598-021-81051-8 (PMC7811010; doi:10.1038/s41598-021-81051-8)
Supplement: Supplementary file 1 — Supplementary Information. [file 41598_2021_81051_MOESM1_ESM.pdf]

SUPPLEMENTARY MATERIALS

TITLE

Summer weather conditions influence winter survival of honey bees (*Apis mellifera*) in the northeastern United States

AUTHORS

Martina Calovi[1]*, Christina M. Grozinger[2], Douglas A. Miller[3,4], Sarah C. Goslee[5]*

*co-corresponding authors: mcalovi@psu.edu , Sarah.Goslee@usda.gov

1. Department of Ecosystem Science and Management, The Pennsylvania State University, University Park, PA, 16802, USA.
2. Department of Entomology, Center for Pollinator Research, Huck Institutes of the Life Sciences, The Pennsylvania University, University Park, PA, 16802, USA.
3. Department of Geography, The Pennsylvania State University, University Park, PA, 16802, USA.
4. Department of Ecosystem Science and Management, The Pennsylvania State University, University Park, PA, 16802, USA.
5. USDA-ARS Pasture Systems and Watershed Management Research Unit, University Park, PA, 16802, USA.

Sample questions from Pennsylvania State Beekeepers Survey S1 (please note that the exact list of questions may have varied between years, and thus we focused on questions that were consistently used)

Are you a current member of the PA State Beekeepers Association?

How many colonies of honey bees did you have alive in November of 20???

How many of these colonies have survived, as of mid-April 20???

Do you monitor for mites?

Did you use any mite treatments on these colonies?

Have you found small hive beetles in your colonies?

Were any of your colonies used to provide pollination services?

If so, what crops did they primarily pollinate?

GPS coordinates (if known)

How did you replace losses?

When do you have your primary nectar flow?

What is your primary nectar flow of the year?

Are you raising nucs for sale in Pennsylvania in 2019?

How many years experience do you have keeping honey bees?

Table S2: Anonymized survey database included with corresponding weather, topographic, landscape and management data (geographic information is missing for privacy concerns). A description of all the variables is included, and it follows Table 1 presented in the main manuscript.

	**Description**
id	Sequential number
spring_yr	Spring year
survival	Binary survival response
bee1	Winter minimum temperature
bee2	Winter total precipitation
bee3	Winter days within the bee-optimal temperature range -5 °C to +10 °C
bee4	Winter days with maximum temperature above 16 °C and precipitation below 3 mm
bee5	Winter minimum temperature variation
bee6	Autumn total precipitation
gdd	Growing degree days (base 5 C)
dd_rain	Days between rain events > 0.25 mm
bioc02	Mean diurnal temperature range
bioc03	Temperature isothermality
bioc04	Temperature seasonality
bioc05	Maximum temperature of warmest month
bioc06	Minimum Temperature of Coldest Month
bioc07	Temperature Annual Range
bioc08	Mean Temperature of Wettest Quarter
bioc09	Mean Temperature of Driest Quarter
bioc12	Annual Precipitation
bioc16	Precipitation of Wettest Quarter
bioc17	Precipitation of Driest Quarter
bioc18	Precipitation of Warmest Quarter
bioc19	Precipitation of Coldest Quarter
elevation	Elevation
slope	Slope
solar_rad	Potential incident solar radiation, 21 Dec
pcurv	Profile curvature
tcurv	Terrain curvature
twi	Topographic wetness index
EW	East/West orientation of slope
NS	North/South orientation of slope
ITL	Distance-weighted Insect Toxic Load
FRI	Distance-weighted Forage Quality autumn
exp_yrs	Beekeeper years of experience
col_nov	Number of colonies in November

Table S2 - Anonymized database

id	spring_yr	survival	bee1	bee2	bee3	bee4	bee5	bee6
1	2017	1	-4.36069	255.949	42	3	5.219447	199.134
2	2017	1	-4.36069	255.949	42	3	5.219447	199.134
3	2017	1	-3.91699	250.93	41	4	5.13138	158.388
4	2017	1	-3.91699	250.93	41	4	5.13138	158.388
5	2017	0	-3.91699	250.93	41	4	5.13138	158.388
6	2017	0	-3.91699	250.93	41	4	5.13138	158.388
7	2017	0	-3.91699	250.93	41	4	5.13138	158.388
8	2017	0	-3.91699	250.93	41	4	5.13138	158.388
9	2017	0	-3.91699	250.93	41	4	5.13138	158.388
10	2017	0	-3.82073	260.689	43	3	5.227614	191.46
11	2017	1	-2.84711	309.225	43	4	5.231623	420.546
12	2017	1	-4.23402	254.908	41	4	5.25069	174.998
13	2017	1	-4.23402	254.908	41	4	5.25069	174.998
14	2017	1	-4.23402	254.908	41	4	5.25069	174.998
15	2017	1	-4.23402	254.908	41	4	5.25069	174.998
16	2017	1	-3.11921	256.488	39	9	5.628216	249.682
17	2017	0	-2.66657	279.187	39	9	5.705937	251.112
18	2017	1	-2.34964	250.488	43	8	5.578132	205.594
19	2017	1	-2.34964	250.488	43	8	5.578132	205.594
20	2017	1	-3.25281	268.095	38	9	5.634371	255.319
21	2017	1	-3.25281	268.095	38	9	5.634371	255.319
22	2017	1	-3.25281	268.095	38	9	5.634371	255.319
23	2017	1	-3.25281	268.095	38	9	5.634371	255.319
24	2017	1	-3.25281	268.095	38	9	5.634371	255.319
25	2017	0	-3.25281	268.095	38	9	5.634371	255.319
26	2017	0	-3.25281	268.095	38	9	5.634371	255.319
27	2017	0	-1.1028	254.063	47	7	4.569406	154.869
28	2017	0	-1.1028	254.063	47	7	4.569406	154.869
29	2017	1	-1.67815	243.274	42	6	4.674064	172.809
30	2017	1	-0.82113	231.405	47	7	4.717594	179.124
31	2017	1	-0.82113	231.405	47	7	4.717594	179.124
32	2017	0	-0.82113	231.405	47	7	4.717594	179.124
33	2017	0	-5.50916	271.421	28	4	5.703766	245.691
34	2017	0	-5.50916	271.421	28	4	5.703766	245.691
35	2017	1	-2.44371	217.816	48	5	4.640893	138.423
36	2017	1	-5.89067	255.451	29	3	5.851359	271.748
37	2017	1	-5.89067	255.451	29	3	5.851359	271.748
38	2017	1	-5.89067	255.451	29	3	5.851359	271.748
39	2017	1	-5.89067	255.451	29	3	5.851359	271.748
40	2017	0	-5.89067	255.451	29	3	5.851359	271.748
41	2017	1	-2.41516	281.324	51	4	4.609469	135.313
42	2017	1	-0.62281	196.601	50	9	5.000262	120.801
43	2017	0	-4.15284	241.354	44	4	5.075255	165.691
44	2017	0	-4.15284	241.354	44	4	5.075255	165.691
45	2017	1	-4.02763	320.581	39	5	5.949704	283.617
46	2017	0	-4.02763	320.581	39	5	5.949704	283.617
47	2017	0	-4.02763	320.581	39	5	5.949704	283.617
48	2017	0	-4.02763	320.581	39	5	5.949704	283.617
49	2017	0	-4.02763	320.581	39	5	5.949704	283.617

Table S2 - Anonymized database

50	2017	1	-3.91699	250.93	41	4	5.13138	158.388
51	2017	1	-3.91699	250.93	41	4	5.13138	158.388
52	2017	1	-3.91699	250.93	41	4	5.13138	158.388
53	2017	0	-3.91699	250.93	41	4	5.13138	158.388
54	2017	0	-3.91699	250.93	41	4	5.13138	158.388
55	2017	0	-3.91699	250.93	41	4	5.13138	158.388
56	2017	0	-3.91699	250.93	41	4	5.13138	158.388
57	2017	1	-2.44569	267.856	50	6	4.747708	161.22
58	2017	1	-2.19266	223.705	40	6	4.845217	179.86
59	2017	1	-2.19266	223.705	40	6	4.845217	179.86
60	2017	1	-2.19266	223.705	40	6	4.845217	179.86
61	2017	0	-6.27829	280.736	29	1	5.385093	166.786
62	2017	0	-6.27829	280.736	29	1	5.385093	166.786
63	2017	0	-6.27829	280.736	29	1	5.385093	166.786
64	2017	0	-6.27829	280.736	29	1	5.385093	166.786
65	2017	0	-1.74929	187.55	51	6	4.50047	158.041
66	2017	0	-1.74929	187.55	51	6	4.50047	158.041
67	2017	0	-2.76385	342.135	39	5	5.909051	323.233
68	2017	0	-1.51202	234.967	46	5	4.498084	158.335
69	2017	0	-1.51202	234.967	46	5	4.498084	158.335
70	2017	0	-4.71991	235.65	40	4	5.499929	190.248
71	2017	0	-4.71991	235.65	40	4	5.499929	190.248
72	2017	1	-3.37782	317.836	33	7	6.291442	235.771
73	2017	1	-3.37782	317.836	33	7	6.291442	235.771
74	2017	0	-3.37782	317.836	33	7	6.291442	235.771
75	2017	0	-3.37782	317.836	33	7	6.291442	235.771
76	2017	1	-2.37481	292.987	41	9	5.935042	294.079
77	2017	1	-6.08353	278.206	33	2	5.33728	208.905
78	2017	1	-6.08353	278.206	33	2	5.33728	208.905
79	2017	1	-6.08353	278.206	33	2	5.33728	208.905
80	2017	0	-6.08353	278.206	33	2	5.33728	208.905
81	2017	0	-6.08353	278.206	33	2	5.33728	208.905
82	2017	0	-6.08353	278.206	33	2	5.33728	208.905
83	2017	0	-6.08353	278.206	33	2	5.33728	208.905
84	2017	0	-6.08353	278.206	33	2	5.33728	208.905
85	2017	0	-4.41719	332.607	34	4	5.723377	344.114
86	2017	0	-4.41719	332.607	34	4	5.723377	344.114
87	2017	1	-4.35438	342.828	33	3	5.835702	311.989
88	2017	0	-4.35438	342.828	33	3	5.835702	311.989
89	2017	0	-0.95466	262.192	47	6	4.689568	144.347
90	2018	1	-6.88819	301.882	29	2	6.529027	195.725
91	2018	0	-6.88819	301.882	29	2	6.529027	195.725
92	2018	0	-6.88819	301.882	29	2	6.529027	195.725
93	2018	0	-6.88819	301.882	29	2	6.529027	195.725
94	2018	0	-6.88819	301.882	29	2	6.529027	195.725
95	2018	0	-6.88819	301.882	29	2	6.529027	195.725
96	2018	0	-7.43247	332.114	25	3	6.71717	208.137
97	2018	1	-6.48333	394.557	17	3	7.808361	262.725
98	2018	1	-6.48333	394.557	17	3	7.808361	262.725
99	2018	1	-6.48333	394.557	17	3	7.808361	262.725

Table S2 - Anonymized database

100	2018	1	-6.48333	394.557	17	3	7.808361	262.725
101	2018	1	-6.48333	394.557	17	3	7.808361	262.725
102	2018	1	-6.48333	394.557	17	3	7.808361	262.725
103	2018	0	-6.48333	394.557	17	3	7.808361	262.725
104	2018	1	-5.90725	366.045	21	2	7.512705	252.161
105	2018	1	-5.90725	366.045	21	2	7.512705	252.161
106	2018	1	-5.90725	366.045	21	2	7.512705	252.161
107	2018	1	-6.40208	277.682	30	2	6.105073	258.973
108	2018	0	-6.40208	277.682	30	2	6.105073	258.973
109	2018	1	-7.39365	313.661	20	2	7.515178	245.655
110	2018	1	-7.39365	313.661	20	2	7.515178	245.655
111	2018	1	-7.39365	313.661	20	2	7.515178	245.655
112	2018	1	-5.77559	374.622	16	2	7.911587	269.805
113	2018	0	-5.77559	374.622	16	2	7.911587	269.805
114	2018	1	-6.21015	363.865	21	2	7.552881	264.371
115	2018	1	-6.21015	363.865	21	2	7.552881	264.371
116	2018	1	-6.21015	363.865	21	2	7.552881	264.371
117	2018	1	-6.49495	310.095	24	1	6.916134	351.541
118	2018	0	-6.49495	310.095	24	1	6.916134	351.541
119	2018	1	-4.617	272.42	34	2	5.883047	224.837
120	2018	1	-4.617	272.42	34	2	5.883047	224.837
121	2018	1	-4.617	272.42	34	2	5.883047	224.837
122	2018	1	-4.617	272.42	34	2	5.883047	224.837
123	2018	1	-4.617	272.42	34	2	5.883047	224.837
124	2018	1	-4.617	272.42	34	2	5.883047	224.837
125	2018	1	-4.617	272.42	34	2	5.883047	224.837
126	2018	1	-8.65423	308.168	20	2	7.117683	236.414
127	2018	0	-7.20009	364.583	20	2	7.820609	262.127
128	2018	1	-4.87848	263.795	31	3	5.976067	224.208
129	2018	1	-4.87848	263.795	31	3	5.976067	224.208
130	2018	0	-4.87848	263.795	31	3	5.976067	224.208
131	2018	0	-4.87848	263.795	31	3	5.976067	224.208
132	2018	0	-4.87848	263.795	31	3	5.976067	224.208
133	2018	1	-4.4763	261.526	31	2	5.876564	215.879
134	2018	1	-4.4763	261.526	31	2	5.876564	215.879
135	2018	1	-6.33545	385.796	20	2	7.653489	281.62
136	2018	1	-6.33545	385.796	20	2	7.653489	281.62
137	2018	1	-6.33545	385.796	20	2	7.653489	281.62
138	2018	0	-6.351	266.57	33	3	6.343261	241.322
139	2018	1	-6.90174	306.527	25	3	6.60448	168.631
140	2018	0	-5.69859	365.818	22	2	7.792316	251.577
141	2018	1	-4.74038	330.101	31	2	6.290736	216.878
142	2018	1	-7.82124	334.567	18	1	7.513873	250.932
143	2018	1	-7.82124	334.567	18	1	7.513873	250.932
144	2018	0	-7.82124	334.567	18	1	7.513873	250.932
145	2018	0	-7.82124	334.567	18	1	7.513873	250.932
146	2018	0	-7.82124	334.567	18	1	7.513873	250.932
147	2018	1	-5.39716	365.214	28	2	7.43428	266.66
148	2018	1	-4.89354	249.698	28	3	6.306965	191.885
149	2018	0	-4.89354	249.698	28	3	6.306965	191.885

Table S2 - Anonymized database

150	2018	1	-5.50224	229.409	26	3	6.216757	249.32
151	2018	1	-6.75184	351.38	15	2	7.815742	280.961
152	2018	1	-4.7954	320.677	31	2	6.34931	176.38
153	2018	0	-4.7954	320.677	31	2	6.34931	176.38
154	2018	0	-5.1961	326.94	31	2	6.173674	222.067
155	2018	1	-6.58408	381.708	16	3	7.864085	312.963
156	2018	0	-6.58408	381.708	16	3	7.864085	312.963
157	2018	0	-6.58408	381.708	16	3	7.864085	312.963
158	2018	1	-5.14502	320.559	29	3	6.399948	186.715
159	2018	1	-5.14502	320.559	29	3	6.399948	186.715
160	2018	1	-7.94169	345.115	18	2	7.964071	283.577
161	2018	0	-7.94169	345.115	18	2	7.964071	283.577
162	2018	0	-4.61864	274.952	35	3	5.960754	208.252
163	2018	1	-7.55909	356.968	19	1	7.863856	261.97
164	2018	1	-5.02796	330.199	33	2	5.989155	220.014
165	2018	1	-5.02796	330.199	33	2	5.989155	220.014
166	2018	1	-5.02796	330.199	33	2	5.989155	220.014
167	2018	1	-5.02796	330.199	33	2	5.989155	220.014
168	2018	1	-5.02796	330.199	33	2	5.989155	220.014
169	2018	1	-5.02796	330.199	33	2	5.989155	220.014
170	2018	1	-5.02796	330.199	33	2	5.989155	220.014
171	2018	0	-5.02796	330.199	33	2	5.989155	220.014
172	2018	1	-4.53398	301.064	30	1	6.283117	206.422
173	2018	1	-4.25138	245.162	33	1	5.949284	183.973
174	2018	1	-4.25138	245.162	33	1	5.949284	183.973
175	2018	1	-4.25138	245.162	33	1	5.949284	183.973
176	2018	1	-4.25138	245.162	33	1	5.949284	183.973
177	2018	0	-7.07599	282.616	29	2	6.611127	164.806
178	2018	0	-7.07599	282.616	29	2	6.611127	164.806
179	2018	0	-7.07599	282.616	29	2	6.611127	164.806
180	2018	0	-7.07599	282.616	29	2	6.611127	164.806
181	2018	1	-7.81114	353.792	19	2	7.937767	285.335
182	2018	1	-7.81114	353.792	19	2	7.937767	285.335
183	2018	1	-4.56224	327.497	31	2	6.316774	179.786
184	2018	0	-4.56224	327.497	31	2	6.316774	179.786
185	2018	0	-4.92368	249.868	27	2	6.349614	181.04
186	2018	0	-4.92368	249.868	27	2	6.349614	181.04
187	2018	0	-4.92368	249.868	27	2	6.349614	181.04
188	2018	0	-4.92368	249.868	27	2	6.349614	181.04
189	2018	1	-8.36164	232.982	28	1	6.739847	155.565
190	2018	1	-8.36164	232.982	28	1	6.739847	155.565
191	2018	0	-8.36164	232.982	28	1	6.739847	155.565
192	2018	1	-6.20356	372.322	21	2	7.571872	272.363
193	2018	1	-6.30913	329.647	29	2	6.266621	226.935
194	2018	1	-6.30913	329.647	29	2	6.266621	226.935
195	2018	1	-6.30913	329.647	29	2	6.266621	226.935
196	2018	0	-6.30913	329.647	29	2	6.266621	226.935
197	2019	1	-4.32079	298.703	40	2	5.256502	468.632
198	2019	1	-4.15021	384.071	44	1	5.121034	480.147
199	2019	0	-4.24736	331.21	43	4	5.016366	519.1

Table S2 - Anonymized database

200	2019	0	-3.81418	331.612	44	2	4.772582	511.533
201	2019	1	-6.17555	270.877	38	0	5.833252	484.947
202	2019	1	-4.73384	352.958	40	1	5.403044	537.427
203	2019	1	-3.75734	322.093	44	2	4.80665	475.52
204	2019	1	-1.95247	398.256	53	3	4.525149	640.779
205	2019	0	-4.57584	303.157	40	2	5.300677	463.864
206	2019	1	-4.53618	313.966	39	2	5.291598	478.142
207	2019	1	-4.33018	342.578	39	2	5.477741	614.214
208	2019	1	-4.33018	342.578	39	2	5.477741	614.214
209	2019	1	-4.41011	336.875	47	1	5.149436	524.707
210	2019	0	-4.41011	336.875	47	1	5.149436	524.707
211	2019	1	-4.2633	325.812	40	2	5.236413	489.847
212	2019	0	-6.36062	318.007	40	0	5.700947	458.444
213	2019	1	-4.22433	320.648	46	1	5.172312	491.093
214	2019	1	-4.22433	320.648	46	1	5.172312	491.093
215	2019	0	-3.18124	322	53	1	4.868373	472.559
216	2019	0	-3.9488	354.511	46	1	5.098173	568.326
217	2019	1	-4.14487	326.941	42	2	5.308244	534.958
218	2019	0	-3.32877	405.774	47	2	5.007678	505.575
219	2019	1	-6.24288	391.685	38	0	5.22349	575.969
220	2019	1	-3.75855	321.524	48	1	5.03645	476.372
221	2019	1	-3.23505	292.894	50	2	5.061666	468.763
222	2019	0	-2.62644	335.621	49	3	4.628169	565.458
223	2019	0	-4.41796	375.687	39	1	5.141097	548.239
224	2019	1	-4.17502	356.028	45	2	5.06267	509.974
225	2019	1	-4.27281	352.552	45	1	5.109867	518.887
226	2019	0	-4.27281	352.552	45	1	5.109867	518.887
227	2019	0	-6.55951	299.438	36	0	6.000892	471.31
228	2019	1	-3.6911	330.824	47	1	4.999539	589.911
229	2019	0	-3.6911	330.824	47	1	4.999539	589.911
230	2019	0	-6.23256	263.12	35	1	5.8627	383.889
231	2019	0	-6.23256	263.12	35	1	5.8627	383.889
232	2019	0	-4.46262	322.218	37	2	5.455272	601.429
233	2019	0	-2.48211	400.639	51	2	4.783273	514.754
234	2019	0	-3.63914	334.62	46	2	4.63291	511.473
235	2019	1	-5.15019	289.288	45	1	5.376062	499.956
236	2019	1	-5.07581	330.386	32	2	5.808935	496.347
237	2019	1	-5.07581	330.386	32	2	5.808935	496.347
238	2019	0	-5.07581	330.386	32	2	5.808935	496.347
239	2019	0	-5.07581	330.386	32	2	5.808935	496.347
240	2019	1	-5.94608	339.673	38	0	5.682072	507.493
241	2019	1	-5.94608	339.673	38	0	5.682072	507.493
242	2019	1	-4.23208	365.71	43	1	5.194001	519.539
243	2019	1	-4.46704	342.849	36	2	5.522096	511.449
244	2019	1	-8.06668	248.124	28	0	5.879601	501.356
245	2019	1	-4.48699	335.255	43	2	5.329501	560.741
246	2019	1	-4.48699	335.255	43	2	5.329501	560.741
247	2019	1	-6.9887	274.518	39	0	6.269623	498.654
248	2019	1	-4.72295	327.698	44	1	5.411894	477.704
249	2019	0	-4.72295	327.698	44	1	5.411894	477.704

Table S2 - Anonymized database

250	2019	0	-4.72295	327.698	44	1	5.411894	477.704
251	2019	1	-5.31249	332.657	37	1	5.727701	529.991
252	2019	1	-6.41129	269.816	36	0	5.565534	563.718
253	2019	0	-5.40087	309.561	36	0	5.442493	484.634
254	2019	0	-5.40087	309.561	36	0	5.442493	484.634
255	2019	0	-5.40087	309.561	36	0	5.442493	484.634
256	2019	1	-3.51463	367.233	49	2	5.232931	532.969
257	2019	1	-4.05765	338.255	40	1	5.013981	520.512
258	2019	1	-4.05765	338.255	40	1	5.013981	520.512
259	2019	1	-4.05765	338.255	40	1	5.013981	520.512
260	2019	1	-5.5641	310.64	43	0	5.404042	506.256
261	2019	1	-5.5641	310.64	43	0	5.404042	506.256
262	2019	0	-5.47236	416.38	34	1	5.313608	622.153
263	2019	1	-3.66295	333.767	45	2	4.952486	513.223
264	2019	0	-3.66295	333.767	45	2	4.952486	513.223
265	2019	1	-4.79401	322.572	45	1	5.279937	571.487
266	2019	0	-4.79401	322.572	45	1	5.279937	571.487
267	2019	1	-4.75329	310.003	46	0	5.133521	487.816
268	2019	1	-4.75329	310.003	46	0	5.133521	487.816
269	2019	1	-3.28244	324.213	43	2	4.698999	534.732
270	2019	1	-3.28244	324.213	43	2	4.698999	534.732
271	2019	1	-3.28244	324.213	43	2	4.698999	534.732
272	2019	1	-3.50415	318.524	53	1	5.022956	490.892
273	2019	0	-3.50415	318.524	53	1	5.022956	490.892
274	2019	1	-6.05771	256.914	35	2	5.984195	423.871
275	2019	1	-6.05771	256.914	35	2	5.984195	423.871
276	2019	1	-4.40974	345.852	46	1	5.137047	529.694
277	2019	0	-4.40974	345.852	46	1	5.137047	529.694
278	2019	0	-7.76235	277.011	30	0	6.483886	487.437
279	2019	0	-4.07084	313.487	45	1	5.190497	514.185
280	2019	1	-5.19449	358.12	38	1	5.838659	552.653
281	2019	1	-5.19449	358.12	38	1	5.838659	552.653
282	2019	1	-4.29449	321.305	42	1	5.220583	528.529
283	2019	0	-4.29449	321.305	42	1	5.220583	528.529
284	2019	1	-4.63667	327.979	38	2	5.431978	566.958
285	2019	1	-4.63667	327.979	38	2	5.431978	566.958
286	2019	1	-4.63667	327.979	38	2	5.431978	566.958
287	2019	0	-4.63667	327.979	38	2	5.431978	566.958
288	2019	1	-4.4804	331.583	42	1	5.148194	518.009
289	2019	0	-3.75833	320.514	50	1	5.0674	485.061
290	2019	1	-3.91564	347.476	38	3	5.476024	565.449
291	2019	1	-4.061	330.015	41	2	5.321926	523.361
292	2019	1	-4.061	330.015	41	2	5.321926	523.361
293	2019	1	-3.9578	317.211	45	1	5.123914	480.404
294	2019	1	-3.9578	317.211	45	1	5.123914	480.404
295	2019	1	-6.43747	269.529	36	0	5.95026	447.542
296	2019	0	-6.43747	269.529	36	0	5.95026	447.542
297	2019	0	-6.43747	269.529	36	0	5.95026	447.542
298	2019	0	-3.55612	376.155	48	2	5.019857	559.01
299	2019	0	-3.55612	376.155	48	2	5.019857	559.01

Table S2 - Anonymized database

300	2019	1	-5.59907	348.703	37	0	5.467555	576.059
301	2019	1	-5.59907	348.703	37	0	5.467555	576.059
302	2019	0	-4.20769	318.57	43	3	4.95145	488.611
303	2019	0	-4.20769	318.57	43	3	4.95145	488.611
304	2019	0	-4.20769	318.57	43	3	4.95145	488.611
305	2019	0	-4.20769	318.57	43	3	4.95145	488.611
306	2019	1	-3.69078	312.203	44	2	5.240152	492.006
307	2019	1	-3.55705	304.823	47	2	5.08267	474.252
308	2019	0	-3.55705	304.823	47	2	5.08267	474.252
309	2019	1	-4.33927	330.623	43	2	5.00001	502.706
310	2019	1	-4.33927	330.623	43	2	5.00001	502.706
311	2019	1	-4.33927	330.623	43	2	5.00001	502.706
312	2019	1	-4.33927	330.623	43	2	5.00001	502.706
313	2019	1	-4.38503	341.272	44	1	5.119593	519.767
314	2019	1	-4.33927	330.623	43	2	5.00001	502.706
315	2019	1	-4.33927	330.623	43	2	5.00001	502.706
316	2019	1	-4.91638	309.095	41	2	5.392417	497.162
317	2019	1	-4.91638	309.095	41	2	5.392417	497.162
318	2019	1	-5.87555	315.59	40	0	5.694942	516.263
319	2019	1	-5.87555	315.59	40	0	5.694942	516.263
320	2019	0	-5.87555	315.59	40	0	5.694942	516.263
321	2019	1	-6.59369	250.609	36	0	5.62224	483.596
322	2019	0	-5.84955	308.039	32	0	5.886017	561.172
323	2019	1	-3.49504	313.277	46	2	4.914761	491.934
324	2019	0	-3.49504	313.277	46	2	4.914761	491.934
325	2019	1	-4.19045	320.987	41	2	5.221517	501.406
326	2019	1	-4.19045	320.987	41	2	5.221517	501.406
327	2019	1	-4.19045	320.987	41	2	5.221517	501.406
328	2019	1	-6.17124	287.268	41	0	5.625112	492.719
329	2019	1	-6.17124	287.268	41	0	5.625112	492.719
330	2019	1	-6.17124	287.268	41	0	5.625112	492.719
331	2019	1	-6.17124	287.268	41	0	5.625112	492.719
332	2019	1	-2.67178	364.946	50	2	4.603849	583.92
333	2019	1	-2.67178	364.946	50	2	4.603849	583.92
334	2019	0	-2.67178	364.946	50	2	4.603849	583.92
335	2019	1	-3.5203	317.732	49	1	4.986291	463.512
336	2019	1	-3.5203	317.732	49	1	4.986291	463.512
337	2019	1	-3.5203	317.732	49	1	4.986291	463.512
338	2019	1	-3.93419	340.409	38	3	5.31214	548.711
339	2019	1	-3.93419	340.409	38	3	5.31214	548.711
340	2019	1	-3.93419	340.409	38	3	5.31214	548.711
341	2019	1	-4.47043	303.935	41	1	5.195107	513.713
342	2019	1	-4.47043	303.935	41	1	5.195107	513.713
343	2019	1	-4.27204	308.602	41	2	5.232071	467.972
344	2019	1	-4.27204	308.602	41	2	5.232071	467.972
345	2019	1	-4.27204	308.602	41	2	5.232071	467.972
346	2019	1	-4.27204	308.602	41	2	5.232071	467.972
347	2019	0	-4.27204	308.602	41	2	5.232071	467.972
348	2019	0	-5.24159	310.989	43	0	5.287071	440.49
349	2019	0	-5.24159	310.989	43	0	5.287071	440.49

Table S2 - Anonymized database

350	2019	1	-3.87159	318.522	48	1	5.018917	488.66
351	2019	0	-3.87159	318.522	48	1	5.018917	488.66
352	2019	0	-3.87159	318.522	48	1	5.018917	488.66
353	2019	0	-4.45918	334.315	37	2	5.523238	511.474
354	2019	0	-4.45918	334.315	37	2	5.523238	511.474
355	2019	0	-4.45918	334.315	37	2	5.523238	511.474
356	2019	0	-7.82548	322.886	28	0	5.510701	528.394
357	2019	0	-7.82548	322.886	28	0	5.510701	528.394
358	2019	1	-4.05768	386.287	43	1	5.090078	505.107
359	2019	1	-4.05768	386.287	43	1	5.090078	505.107
360	2019	0	-4.05768	386.287	43	1	5.090078	505.107
361	2019	0	-4.05768	386.287	43	1	5.090078	505.107
362	2019	0	-4.05768	386.287	43	1	5.090078	505.107
363	2019	0	-4.05768	386.287	43	1	5.090078	505.107
364	2019	0	-7.33269	184.695	33	0	5.911776	469.051
365	2019	0	-7.33269	184.695	33	0	5.911776	469.051
366	2019	0	-7.33269	184.695	33	0	5.911776	469.051
367	2019	0	-7.33269	184.695	33	0	5.911776	469.051
368	2019	1	-3.96623	304.299	52	1	5.140466	459.523
369	2019	1	-3.96623	304.299	52	1	5.140466	459.523
370	2019	0	-3.96623	304.299	52	1	5.140466	459.523
371	2019	0	-3.96623	304.299	52	1	5.140466	459.523
372	2019	1	-7.65899	238.94	34	0	5.859651	518.333
373	2019	1	-7.65899	238.94	34	0	5.859651	518.333
374	2019	0	-7.65899	238.94	34	0	5.859651	518.333
375	2019	1	-5.6137	294.652	41	0	5.426873	543.489
376	2019	1	-5.6137	294.652	41	0	5.426873	543.489
377	2019	1	-5.6137	294.652	41	0	5.426873	543.489
378	2019	0	-5.6137	294.652	41	0	5.426873	543.489
379	2019	0	-5.6137	294.652	41	0	5.426873	543.489
380	2019	1	-7.00074	299.039	36	0	6.096522	389.577
381	2019	0	-7.00074	299.039	36	0	6.096522	389.577
382	2019	0	-7.00074	299.039	36	0	6.096522	389.577
383	2019	0	-7.00074	299.039	36	0	6.096522	389.577
384	2019	1	-2.90229	317.849	52	2	4.930131	537.539
385	2019	1	-2.90229	317.849	52	2	4.930131	537.539
386	2019	1	-2.90229	317.849	52	2	4.930131	537.539
387	2019	1	-2.90229	317.849	52	2	4.930131	537.539
388	2019	1	-3.93111	324.98	44	2	5.178228	540.026
389	2019	1	-3.93111	324.98	44	2	5.178228	540.026
390	2019	1	-3.93111	324.98	44	2	5.178228	540.026
391	2019	1	-3.4318	319.197	46	2	5.039764	515.073
392	2019	1	-3.4318	319.197	46	2	5.039764	515.073
393	2019	1	-3.4318	319.197	46	2	5.039764	515.073
394	2019	1	-4.55829	313.129	39	1	4.963728	501.908
395	2019	1	-4.55829	313.129	39	1	4.963728	501.908
396	2019	0	-4.55829	313.129	39	1	4.963728	501.908
397	2019	1	-8.35988	217.186	26	0	6.415313	407.724
398	2019	1	-8.35988	217.186	26	0	6.415313	407.724
399	2019	1	-8.35988	217.186	26	0	6.415313	407.724

Table S2 - Anonymized database

400	2019	1	-8.35988	217.186	26	0	6.415313	407.724
401	2019	1	-5.31593	314.043	36	2	5.66843	508.904
402	2019	1	-5.31593	314.043	36	2	5.66843	508.904
403	2019	1	-5.31593	314.043	36	2	5.66843	508.904
404	2019	1	-5.31593	314.043	36	2	5.66843	508.904
405	2019	1	-5.31593	314.043	36	2	5.66843	508.904
406	2019	1	-5.31593	314.043	36	2	5.66843	508.904
407	2019	1	-4.7944	356.626	44	1	5.006661	556.198
408	2019	1	-4.7944	356.626	44	1	5.006661	556.198
409	2019	1	-4.7944	356.626	44	1	5.006661	556.198
410	2019	1	-4.7944	356.626	44	1	5.006661	556.198
411	2019	1	-4.45186	362.907	39	1	5.173466	572.828
412	2019	1	-4.45186	362.907	39	1	5.173466	572.828
413	2019	1	-4.45186	362.907	39	1	5.173466	572.828
414	2019	1	-4.45186	362.907	39	1	5.173466	572.828
415	2019	1	-4.45186	362.907	39	1	5.173466	572.828
416	2019	1	-4.45186	362.907	39	1	5.173466	572.828
417	2019	1	-4.45186	362.907	39	1	5.173466	572.828
418	2019	1	-4.45186	362.907	39	1	5.173466	572.828
419	2019	1	-4.45186	362.907	39	1	5.173466	572.828
420	2019	1	-4.45186	362.907	39	1	5.173466	572.828
421	2019	1	-3.3129	348.276	48	1	4.975174	484.098
422	2019	1	-3.3129	348.276	48	1	4.975174	484.098
423	2019	1	-3.3129	348.276	48	1	4.975174	484.098
424	2019	1	-3.3129	348.276	48	1	4.975174	484.098
425	2019	1	-5.45258	304.945	43	0	5.550265	480.654
426	2019	1	-5.45258	304.945	43	0	5.550265	480.654
427	2019	1	-5.45258	304.945	43	0	5.550265	480.654
428	2019	1	-5.45258	304.945	43	0	5.550265	480.654
429	2019	1	-5.45258	304.945	43	0	5.550265	480.654
430	2019	0	-5.45258	304.945	43	0	5.550265	480.654
431	2019	0	-5.45258	304.945	43	0	5.550265	480.654
432	2019	0	-5.45258	304.945	43	0	5.550265	480.654
433	2019	0	-5.45258	304.945	43	0	5.550265	480.654
434	2017	1	-3.25281	268.095	38	9	5.634371	255.319
435	2017	0	-3.91699	250.93	41	4	5.13138	158.388
436	2017	1	-4.02763	320.581	39	5	5.949704	283.617
437	2017	0	-1.9847	238.16	43	6	4.704746	134.217
438	2017	0	-0.82113	231.405	47	7	4.717594	179.124
439	2017	0	-4.23402	254.908	41	4	5.25069	174.998
440	2017	0	-4.02763	320.581	39	5	5.949704	283.617
441	2017	1	-1.74929	187.55	51	6	4.50047	158.041
442	2017	0	-3.91699	250.93	41	4	5.13138	158.388
443	2017	1	-2.66657	279.187	39	9	5.705937	251.112
444	2017	1	-5.50916	271.421	28	4	5.703766	245.691
445	2017	0	-3.91699	250.93	41	4	5.13138	158.388
446	2017	0	-3.91699	250.93	41	4	5.13138	158.388
447	2017	1	-5.89067	255.451	29	3	5.851359	271.748
448	2017	1	-4.15284	241.354	44	4	5.075255	165.691
449	2017	1	-2.68832	269.539	49	4	4.510327	154.518

Table S2 - Anonymized database

450	2017	0	-3.91699	250.93	41	4	5.13138	158.388
451	2017	1	-4.36069	255.949	42	3	5.219447	199.134
452	2017	0	-3.91699	250.93	41	4	5.13138	158.388
453	2017	1	-3.88911	291.142	38	6	6.068213	253.926
454	2017	1	-2.50998	260.164	44	6	4.736388	137.96
455	2017	0	-2.84711	309.225	43	4	5.231623	420.546
456	2017	1	-4.23402	254.908	41	4	5.25069	174.998
457	2017	0	-3.91699	250.93	41	4	5.13138	158.388
458	2017	1	-4.23402	254.908	41	4	5.25069	174.998
459	2017	0	-4.41719	332.607	34	4	5.723377	344.114
460	2017	0	-4.41719	332.607	34	4	5.723377	344.114
461	2017	1	-4.23402	254.908	41	4	5.25069	174.998
462	2017	0	-4.71991	235.65	40	4	5.499929	190.248
463	2017	1	-2.68832	269.539	49	4	4.510327	154.518
464	2017	0	-4.02763	320.581	39	5	5.949704	283.617
465	2017	1	-3.91699	250.93	41	4	5.13138	158.388
466	2017	0	-2.58724	292.76	47	6	4.721779	135.801
467	2017	0	-2.54548	206.109	50	4	4.652779	173.933
468	2017	0	-2.76385	342.135	39	5	5.909051	323.233
469	2017	1	-1.33192	237.143	49	5	4.479017	176.606
470	2017	1	-4.36069	255.949	42	3	5.219447	199.134
471	2017	0	-4.71991	235.65	40	4	5.499929	190.248
472	2017	0	-2.85334	264.701	39	10	5.700035	238.075
473	2017	1	-3.25281	268.095	38	9	5.634371	255.319
474	2017	0	-2.41516	281.324	51	4	4.609469	135.313
475	2017	0	-2.50998	260.164	44	6	4.736388	137.96
476	2017	0	-3.91699	250.93	41	4	5.13138	158.388
477	2017	1	-1.33192	237.143	49	5	4.479017	176.606
478	2017	1	-3.37782	317.836	33	7	6.291442	235.771
479	2017	1	-2.84711	309.225	43	4	5.231623	420.546
480	2017	0	-3.91699	250.93	41	4	5.13138	158.388
481	2017	1	-4.15284	241.354	44	4	5.075255	165.691
482	2017	1	-3.11921	256.488	39	9	5.628216	249.682
483	2017	0	-1.9456	209.2	46	6	4.532762	165.345
484	2017	1	-0.95466	262.192	47	6	4.689568	144.347
485	2017	0	-4.15284	241.354	44	4	5.075255	165.691
486	2017	1	-2.34964	250.488	43	8	5.578132	205.594
487	2017	1	-4.71991	235.65	40	4	5.499929	190.248
488	2017	0	-4.02763	320.581	39	5	5.949704	283.617
489	2017	0	-3.91699	250.93	41	4	5.13138	158.388
490	2017	1	-2.43968	272.889	41	6	4.817793	185.432
491	2017	0	-2.96015	195.957	45	7	4.610741	126.263
492	2017	1	-3.37782	317.836	33	7	6.291442	235.771
493	2017	1	-0.95466	262.192	47	6	4.689568	144.347
494	2017	0	-6.08353	278.206	33	2	5.33728	208.905
495	2017	0	-3.91699	250.93	41	4	5.13138	158.388
496	2017	0	-2.76942	272.611	45	6	4.776855	143.822
497	2017	1	-6.27829	280.736	29	1	5.385093	166.786
498	2017	1	-5.89067	255.451	29	3	5.851359	271.748
499	2017	0	-0.62281	196.601	50	9	5.000262	120.801

Table S2 - Anonymized database

500	2017	1	-2.68832	269.539	49	4	4.510327	154.518
501	2017	1	-1.9456	209.2	46	6	4.532762	165.345
502	2017	0	-3.91699	250.93	41	4	5.13138	158.388
503	2017	0	-6.27829	280.736	29	1	5.385093	166.786
504	2017	0	-4.02763	320.581	39	5	5.949704	283.617
505	2017	1	-4.35438	342.828	33	3	5.835702	311.989
506	2017	0	-5.89067	255.451	29	3	5.851359	271.748
507	2017	0	-3.91699	250.93	41	4	5.13138	158.388
508	2017	1	-3.91699	250.93	41	4	5.13138	158.388
509	2017	1	-3.11921	256.488	39	9	5.628216	249.682
510	2017	1	-2.84711	309.225	43	4	5.231623	420.546
511	2017	1	-5.89067	255.451	29	3	5.851359	271.748
512	2017	1	-2.44569	267.856	50	6	4.747708	161.22
513	2017	0	-3.37782	317.836	33	7	6.291442	235.771
514	2017	1	-2.84711	309.225	43	4	5.231623	420.546
515	2017	0	-3.11921	256.488	39	9	5.628216	249.682
516	2017	1	-2.44569	267.856	50	6	4.747708	161.22
517	2017	0	-3.11921	256.488	39	9	5.628216	249.682
518	2017	0	-3.91699	250.93	41	4	5.13138	158.388
519	2017	0	-6.08353	278.206	33	2	5.33728	208.905
520	2017	1	-3.91699	250.93	41	4	5.13138	158.388
521	2017	1	-5.89067	255.451	29	3	5.851359	271.748
522	2017	1	-0.62281	196.601	50	9	5.000262	120.801
523	2017	1	-3.37782	317.836	33	7	6.291442	235.771
524	2017	0	-3.91699	250.93	41	4	5.13138	158.388
525	2017	0	-2.43968	272.889	41	6	4.817793	185.432
526	2017	1	-2.44371	217.816	48	5	4.640893	138.423
527	2017	1	-0.95466	262.192	47	6	4.689568	144.347
528	2017	1	-5.89067	255.451	29	3	5.851359	271.748
529	2017	1	-5.89067	255.451	29	3	5.851359	271.748
530	2017	1	-0.62281	196.601	50	9	5.000262	120.801
531	2017	0	-4.15284	241.354	44	4	5.075255	165.691
532	2017	0	-1.1028	254.063	47	7	4.569406	154.869
533	2017	1	-2.76942	272.611	45	6	4.776855	143.822
534	2017	1	-5.89067	255.451	29	3	5.851359	271.748
535	2017	0	-4.23402	254.908	41	4	5.25069	174.998
536	2017	1	-2.39007	261.853	45	6	4.668481	169.656
537	2017	1	-6.08353	278.206	33	2	5.33728	208.905
538	2017	0	-1.97122	263.819	46	9	5.531372	306.474
539	2017	0	-4.15284	241.354	44	4	5.075255	165.691
540	2017	1	-5.89067	255.451	29	3	5.851359	271.748
541	2017	0	-2.19266	223.705	40	6	4.845217	179.86
542	2017	1	-3.11921	256.488	39	9	5.628216	249.682
543	2017	0	-4.15284	241.354	44	4	5.075255	165.691
544	2017	0	-3.25281	268.095	38	9	5.634371	255.319
545	2017	0	-2.19266	223.705	40	6	4.845217	179.86
546	2017	0	-2.24355	265.847	46	6	4.715598	129.172
547	2017	0	-4.02763	320.581	39	5	5.949704	283.617
548	2017	0	-2.84711	309.225	43	4	5.231623	420.546
549	2017	1	-4.23402	254.908	41	4	5.25069	174.998

Table S2 - Anonymized database

550	2017	0	-2.24355	265.847	46	6	4.715598	129.172
551	2017	1	-0.62281	196.601	50	9	5.000262	120.801
552	2017	0	-0.95466	262.192	47	6	4.689568	144.347
553	2017	0	-6.08353	278.206	33	2	5.33728	208.905
554	2017	0	-3.91699	250.93	41	4	5.13138	158.388
555	2017	0	-3.91699	250.93	41	4	5.13138	158.388
556	2017	1	-3.48788	217.594	44	5	5.100494	166.871
557	2017	0	-4.15284	241.354	44	4	5.075255	165.691
558	2017	0	-6.27829	280.736	29	1	5.385093	166.786
559	2017	1	-4.36069	255.949	42	3	5.219447	199.134
560	2017	1	-1.74724	252.738	44	6	4.710191	185.309
561	2017	1	-3.91699	250.93	41	4	5.13138	158.388
562	2017	0	-4.36069	255.949	42	3	5.219447	199.134
563	2017	0	-3.25281	268.095	38	9	5.634371	255.319
564	2017	1	-4.36069	255.949	42	3	5.219447	199.134
565	2017	0	-4.02763	320.581	39	5	5.949704	283.617
566	2017	1	-2.44371	217.816	48	5	4.640893	138.423
567	2017	1	-2.19266	223.705	40	6	4.845217	179.86
568	2017	0	-3.25281	268.095	38	9	5.634371	255.319
569	2017	0	-4.41719	332.607	34	4	5.723377	344.114
570	2017	1	-2.50998	260.164	44	6	4.736388	137.96
571	2017	1	-3.91699	250.93	41	4	5.13138	158.388
572	2017	0	-6.08353	278.206	33	2	5.33728	208.905
573	2017	1	-2.19266	223.705	40	6	4.845217	179.86
574	2017	0	-2.43968	272.889	41	6	4.817793	185.432
575	2017	0	-3.25281	268.095	38	9	5.634371	255.319
576	2017	0	-6.27829	280.736	29	1	5.385093	166.786
577	2017	1	-1.71027	266.643	43	7	4.556356	157.905
578	2017	1	-5.89067	255.451	29	3	5.851359	271.748
579	2017	1	-4.71991	235.65	40	4	5.499929	190.248
580	2017	1	-3.91699	250.93	41	4	5.13138	158.388
581	2017	0	-2.43968	272.889	41	6	4.817793	185.432
582	2017	0	-3.91699	250.93	41	4	5.13138	158.388
583	2017	1	-3.37782	317.836	33	7	6.291442	235.771
584	2017	0	-0.62281	196.601	50	9	5.000262	120.801
585	2017	0	-4.15284	241.354	44	4	5.075255	165.691
586	2017	0	-3.91699	250.93	41	4	5.13138	158.388
587	2017	0	-2.40715	236.684	50	4	4.60509	201.376
588	2017	0	-4.41719	332.607	34	4	5.723377	344.114
589	2017	0	-5.50916	271.421	28	4	5.703766	245.691
590	2017	1	-2.50998	260.164	44	6	4.736388	137.96
591	2017	0	-6.27829	280.736	29	1	5.385093	166.786
592	2017	1	-1.67815	243.274	42	6	4.674064	172.809
593	2017	0	-3.91699	250.93	41	4	5.13138	158.388
594	2017	0	-3.25281	268.095	38	9	5.634371	255.319
595	2017	1	-3.48788	217.594	44	5	5.100494	166.871
596	2017	1	-3.11921	256.488	39	9	5.628216	249.682
597	2017	0	-5.50916	271.421	28	4	5.703766	245.691
598	2017	1	-5.89067	255.451	29	3	5.851359	271.748
599	2017	0	-2.84711	309.225	43	4	5.231623	420.546

Table S2 - Anonymized database

600	2017	1	-3.48788	217.594	44	5	5.100494	166.871
601	2017	0	-2.24355	265.847	46	6	4.715598	129.172
602	2017	1	-3.25281	268.095	38	9	5.634371	255.319
603	2017	1	-3.91699	250.93	41	4	5.13138	158.388
604	2017	0	-4.71991	235.65	40	4	5.499929	190.248
605	2017	1	-2.50998	260.164	44	6	4.736388	137.96
606	2017	0	-3.92423	344.363	34	3	5.911061	339.89
607	2017	0	-3.91699	250.93	41	4	5.13138	158.388
608	2017	0	-2.43968	272.889	41	6	4.817793	185.432
609	2017	1	-3.91699	250.93	41	4	5.13138	158.388
610	2017	0	-0.62281	196.601	50	9	5.000262	120.801
611	2017	0	-4.41719	332.607	34	4	5.723377	344.114
612	2017	0	-0.82113	231.405	47	7	4.717594	179.124
613	2017	1	-1.1028	254.063	47	7	4.569406	154.869
614	2017	0	-4.71991	235.65	40	4	5.499929	190.248
615	2017	1	-2.84711	309.225	43	4	5.231623	420.546
616	2017	1	-3.48788	217.594	44	5	5.100494	166.871
617	2017	0	-3.91699	250.93	41	4	5.13138	158.388
618	2017	0	-2.44569	267.856	50	6	4.747708	161.22
619	2017	0	-6.27829	280.736	29	1	5.385093	166.786
620	2017	1	-2.44569	267.856	50	6	4.747708	161.22
621	2017	1	-2.34964	250.488	43	8	5.578132	205.594
622	2017	0	-4.15284	241.354	44	4	5.075255	165.691
623	2017	1	-1.71027	266.643	43	7	4.556356	157.905
624	2017	0	-3.11921	256.488	39	9	5.628216	249.682
625	2017	1	-2.85334	264.701	39	10	5.700035	238.075
626	2017	1	-4.71991	235.65	40	4	5.499929	190.248
627	2017	1	-3.37782	317.836	33	7	6.291442	235.771
628	2017	0	-2.54548	206.109	50	4	4.652779	173.933
629	2017	1	-3.91699	250.93	41	4	5.13138	158.388
630	2017	0	-6.27829	280.736	29	1	5.385093	166.786
631	2017	1	-2.50998	260.164	44	6	4.736388	137.96
632	2017	0	-1.9847	238.16	43	6	4.704746	134.217
633	2017	1	-1.67815	243.274	42	6	4.674064	172.809
634	2017	0	-4.15284	241.354	44	4	5.075255	165.691
635	2017	1	-4.41719	332.607	34	4	5.723377	344.114
636	2017	0	-6.08353	278.206	33	2	5.33728	208.905
637	2017	0	-3.82073	260.689	43	3	5.227614	191.46
638	2017	0	-6.08353	278.206	33	2	5.33728	208.905
639	2017	0	-2.33675	267.894	48	6	4.748405	160.053
640	2018	0	-6.88819	301.882	29	2	6.529027	195.725
641	2018	1	-8.27169	305.458	25	2	6.790788	190.623
642	2018	0	-6.58408	381.708	16	3	7.864085	312.963
643	2018	1	-5.90725	366.045	21	2	7.512705	252.161
644	2018	1	-5.77559	374.622	16	2	7.911587	269.805
645	2018	1	-6.20356	372.322	21	2	7.571872	272.363
646	2018	1	-6.48333	394.557	17	3	7.808361	262.725
647	2018	1	-6.88819	301.882	29	2	6.529027	195.725
648	2018	1	-4.25138	245.162	33	1	5.949284	183.973
649	2018	0	-6.88819	301.882	29	2	6.529027	195.725

Table S2 - Anonymized database

650	2018	1	-6.49495	310.095	24	1	6.916134	351.541
651	2018	1	-7.39365	313.661	20	2	7.515178	245.655
652	2018	0	-6.58408	381.708	16	3	7.864085	312.963
653	2018	1	-6.48333	394.557	17	3	7.808361	262.725
654	2018	0	-4.87848	263.795	31	3	5.976067	224.208
655	2018	0	-6.48333	394.557	17	3	7.808361	262.725
656	2018	1	-7.39365	313.661	20	2	7.515178	245.655
657	2018	1	-7.82124	334.567	18	1	7.513873	250.932
658	2018	1	-4.617	272.42	34	2	5.883047	224.837
659	2018	1	-6.88819	301.882	29	2	6.529027	195.725
660	2018	1	-8.65423	308.168	20	2	7.117683	236.414
661	2018	1	-4.617	272.42	34	2	5.883047	224.837
662	2018	1	-6.48333	394.557	17	3	7.808361	262.725
663	2018	0	-8.27169	305.458	25	2	6.790788	190.623
664	2018	0	-4.793	281.388	34	3	6.025163	214.309
665	2018	1	-7.39365	313.661	20	2	7.515178	245.655
666	2018	0	-4.87848	263.795	31	3	5.976067	224.208
667	2018	1	-4.617	272.42	34	2	5.883047	224.837
668	2018	1	-4.617	272.42	34	2	5.883047	224.837
669	2018	1	-5.02796	330.199	33	2	5.989155	220.014
670	2018	0	-6.90174	306.527	25	3	6.60448	168.631
671	2018	0	-9.06615	295.1	24	1	6.91996	180.283
672	2018	1	-7.81114	353.792	19	2	7.937767	285.335
673	2018	1	-5.36521	366.405	27	2	7.461253	266.411
674	2018	1	-7.39365	313.661	20	2	7.515178	245.655
675	2018	0	-5.02252	263.153	27	3	6.894613	267.039
676	2018	0	-6.88819	301.882	29	2	6.529027	195.725
677	2018	0	-7.07599	282.616	29	2	6.611127	164.806
678	2018	1	-3.97457	269.805	34	2	5.878606	179.97
679	2018	1	-5.02796	330.199	33	2	5.989155	220.014
680	2018	1	-9.06615	295.1	24	1	6.91996	180.283
681	2018	0	-6.58408	381.708	16	3	7.864085	312.963
682	2018	1	-4.87848	263.795	31	3	5.976067	224.208
683	2018	1	-7.39365	313.661	20	2	7.515178	245.655
684	2018	1	-4.87848	263.795	31	3	5.976067	224.208
685	2018	0	-5.14502	320.559	29	3	6.399948	186.715
686	2018	1	-6.30913	329.647	29	2	6.266621	226.935
687	2018	1	-7.07599	282.616	29	2	6.611127	164.806
688	2018	1	-7.20009	364.583	20	2	7.820609	262.127
689	2018	1	-4.87848	263.795	31	3	5.976067	224.208
690	2018	1	-6.48333	394.557	17	3	7.808361	262.725
691	2018	1	-4.37908	311.745	32	1	6.1943	204.526
692	2018	1	-5.14502	320.559	29	3	6.399948	186.715
693	2018	1	-4.87848	263.795	31	3	5.976067	224.208
694	2018	1	-4.617	272.42	34	2	5.883047	224.837
695	2018	0	-7.82124	334.567	18	1	7.513873	250.932
696	2018	1	-4.617	272.42	34	2	5.883047	224.837
697	2018	1	-5.39716	365.214	28	2	7.43428	266.66
698	2018	1	-6.30913	329.647	29	2	6.266621	226.935
699	2018	0	-6.58408	381.708	16	3	7.864085	312.963

Table S2 - Anonymized database

700	2018	1	-4.25138	245.162	33	1	5.949284	183.973
701	2018	1	-4.37908	311.745	32	1	6.1943	204.526
702	2018	1	-5.69859	365.818	22	2	7.792316	251.577
703	2018	1	-4.87848	263.795	31	3	5.976067	224.208
704	2018	1	-9.06615	295.1	24	1	6.91996	180.283
705	2018	1	-4.92368	249.868	27	2	6.349614	181.04
706	2018	1	-6.48333	394.557	17	3	7.808361	262.725
707	2018	0	-6.75184	351.38	15	2	7.815742	280.961
708	2018	1	-5.69859	365.818	22	2	7.792316	251.577
709	2018	1	-4.617	272.42	34	2	5.883047	224.837
710	2018	1	-7.39365	313.661	20	2	7.515178	245.655
711	2018	1	-5.39716	365.214	28	2	7.43428	266.66
712	2018	0	-6.49495	310.095	24	1	6.916134	351.541
713	2018	0	-7.94169	345.115	18	2	7.964071	283.577
714	2018	0	-4.16202	331.251	33	2	6.285675	190.878
715	2018	1	-9.06615	295.1	24	1	6.91996	180.283
716	2018	0	-5.50224	229.409	26	3	6.216757	249.32
717	2018	1	-9.06615	295.1	24	1	6.91996	180.283
718	2018	0	-5.1982	337.653	28	2	6.180266	218.816
719	2018	1	-5.90725	366.045	21	2	7.512705	252.161
720	2018	1	-5.02796	330.199	33	2	5.989155	220.014
721	2018	1	-6.25511	348.549	20	3	7.64078	284.237
722	2018	1	-6.21015	363.865	21	2	7.552881	264.371
723	2018	0	-7.43247	332.114	25	3	6.71717	208.137
724	2018	1	-4.617	272.42	34	2	5.883047	224.837
725	2018	0	-5.1982	337.653	28	2	6.180266	218.816
726	2018	1	-5.02796	330.199	33	2	5.989155	220.014
727	2018	0	-6.58408	381.708	16	3	7.864085	312.963
728	2018	1	-4.87848	263.795	31	3	5.976067	224.208
729	2018	1	-6.48333	394.557	17	3	7.808361	262.725
730	2018	1	-4.89354	249.698	28	3	6.306965	191.885
731	2018	0	-6.30913	329.647	29	2	6.266621	226.935
732	2018	0	-4.92368	249.868	27	2	6.349614	181.04
733	2018	1	-8.36164	232.982	28	1	6.739847	155.565
734	2018	1	-5.14502	320.559	29	3	6.399948	186.715
735	2018	1	-4.92368	249.868	27	2	6.349614	181.04
736	2018	1	-6.21015	363.865	21	2	7.552881	264.371
737	2018	0	-4.92368	249.868	27	2	6.349614	181.04
738	2018	1	-4.617	272.42	34	2	5.883047	224.837
739	2018	1	-6.88819	301.882	29	2	6.529027	195.725
740	2018	1	-6.88819	301.882	29	2	6.529027	195.725
741	2018	1	-6.88819	301.882	29	2	6.529027	195.725
742	2018	1	-9.06615	295.1	24	1	6.91996	180.283
743	2018	1	-6.58408	381.708	16	3	7.864085	312.963
744	2018	0	-5.57762	322.333	30	2	6.382592	213.469
745	2018	0	-4.617	272.42	34	2	5.883047	224.837
746	2018	0	-4.25138	245.162	33	1	5.949284	183.973
747	2018	1	-5.36521	366.405	27	2	7.461253	266.411
748	2018	0	-6.48333	394.557	17	3	7.808361	262.725
749	2018	0	-7.07599	282.616	29	2	6.611127	164.806

Table S2 - Anonymized database

750	2018	1	-4.617	272.42	34	2	5.883047	224.837
751	2018	0	-6.58408	381.708	16	3	7.864085	312.963
752	2018	1	-4.617	272.42	34	2	5.883047	224.837
753	2018	1	-5.69859	365.818	22	2	7.792316	251.577
754	2018	0	-4.92368	249.868	27	2	6.349614	181.04
755	2018	1	-6.21015	363.865	21	2	7.552881	264.371
756	2018	1	-4.00785	282.449	33	2	6.290476	177.442
757	2018	0	-6.49495	310.095	24	1	6.916134	351.541
758	2018	0	-6.49495	310.095	24	1	6.916134	351.541
759	2018	1	-6.21015	363.865	21	2	7.552881	264.371
760	2018	0	-6.88819	301.882	29	2	6.529027	195.725
761	2018	0	-5.44999	360.827	25	2	7.2608	247.862
762	2018	1	-6.30913	329.647	29	2	6.266621	226.935
763	2018	0	-7.07599	282.616	29	2	6.611127	164.806
764	2018	0	-7.55909	356.968	19	1	7.863856	261.97
765	2018	1	-4.617	272.42	34	2	5.883047	224.837
766	2018	1	-6.48333	394.557	17	3	7.808361	262.725
767	2018	0	-5.1961	326.94	31	2	6.173674	222.067
768	2018	1	-7.39365	313.661	20	2	7.515178	245.655
769	2018	1	-5.02796	330.199	33	2	5.989155	220.014
770	2018	0	-6.88819	301.882	29	2	6.529027	195.725
771	2018	0	-4.87848	263.795	31	3	5.976067	224.208
772	2018	1	-6.25511	348.549	20	3	7.64078	284.237
773	2018	1	-4.87848	263.795	31	3	5.976067	224.208
774	2018	1	-5.39716	365.214	28	2	7.43428	266.66
775	2018	1	-7.82124	334.567	18	1	7.513873	250.932
776	2018	1	-4.617	272.42	34	2	5.883047	224.837
777	2018	1	-5.14502	320.559	29	3	6.399948	186.715
778	2018	1	-4.87848	263.795	31	3	5.976067	224.208
779	2018	1	-6.21015	363.865	21	2	7.552881	264.371
780	2018	1	-6.88819	301.882	29	2	6.529027	195.725
781	2018	1	-5.77559	374.622	16	2	7.911587	269.805
782	2018	1	-4.87848	263.795	31	3	5.976067	224.208
783	2018	1	-4.617	272.42	34	2	5.883047	224.837
784	2018	1	-4.617	272.42	34	2	5.883047	224.837
785	2018	1	-8.36164	232.982	28	1	6.739847	155.565
786	2018	1	-9.06615	295.1	24	1	6.91996	180.283
787	2018	1	-4.617	272.42	34	2	5.883047	224.837
788	2018	1	-7.86677	355.888	20	1	7.928198	277.851
789	2018	1	-4.87848	263.795	31	3	5.976067	224.208
790	2018	1	-4.74038	330.101	31	2	6.290736	216.878
791	2018	1	-5.02796	330.199	33	2	5.989155	220.014
792	2018	1	-5.02796	330.199	33	2	5.989155	220.014
793	2018	0	-6.58408	381.708	16	3	7.864085	312.963
794	2018	1	-4.617	272.42	34	2	5.883047	224.837
795	2018	0	-6.58408	381.708	16	3	7.864085	312.963
796	2018	1	-6.21015	363.865	21	2	7.552881	264.371
797	2018	1	-6.75184	351.38	15	2	7.815742	280.961
798	2018	0	-6.58408	381.708	16	3	7.864085	312.963
799	2018	1	-4.617	272.42	34	2	5.883047	224.837

Table S2 - Anonymized database

800	2018	1	-4.87848	263.795	31	3	5.976067	224.208
801	2018	1	-6.21015	363.865	21	2	7.552881	264.371
802	2018	1	-5.44999	360.827	25	2	7.2608	247.862
803	2018	1	-7.07599	282.616	29	2	6.611127	164.806
804	2018	1	-4.87848	263.795	31	3	5.976067	224.208
805	2018	1	-4.87848	263.795	31	3	5.976067	224.208
806	2018	1	-7.20009	364.583	20	2	7.820609	262.127
807	2018	1	-5.69859	365.818	22	2	7.792316	251.577
808	2018	1	-6.30913	329.647	29	2	6.266621	226.935
809	2018	0	-5.44999	360.827	25	2	7.2608	247.862
810	2018	0	-4.92368	249.868	27	2	6.349614	181.04
811	2018	1	-5.02796	330.199	33	2	5.989155	220.014
812	2018	1	-8.08821	336.689	17	1	7.084207	282.846
813	2018	1	-5.90725	366.045	21	2	7.512705	252.161
814	2018	1	-5.14502	320.559	29	3	6.399948	186.715
815	2018	1	-8.65423	308.168	20	2	7.117683	236.414
816	2018	1	-7.81114	353.792	19	2	7.937767	285.335
817	2018	1	-5.77559	374.622	16	2	7.911587	269.805
818	2018	1	-4.87848	263.795	31	3	5.976067	224.208
819	2018	1	-6.33545	385.796	20	2	7.653489	281.62
820	2018	1	-7.20009	364.583	20	2	7.820609	262.127
821	2018	1	-6.25511	348.549	20	3	7.64078	284.237
822	2018	1	-6.49495	310.095	24	1	6.916134	351.541
823	2018	1	-4.87848	263.795	31	3	5.976067	224.208
824	2018	1	-6.88819	301.882	29	2	6.529027	195.725
825	2018	1	-7.86677	355.888	20	1	7.928198	277.851
826	2018	1	-5.39716	365.214	28	2	7.43428	266.66
827	2018	0	-4.92368	249.868	27	2	6.349614	181.04
828	2018	1	-5.02796	330.199	33	2	5.989155	220.014
829	2018	0	-7.20009	364.583	20	2	7.820609	262.127
830	2018	1	-7.20009	364.583	20	2	7.820609	262.127
831	2018	1	-6.30913	329.647	29	2	6.266621	226.935
832	2018	1	-6.21015	363.865	21	2	7.552881	264.371
833	2018	1	-6.88819	301.882	29	2	6.529027	195.725
834	2018	1	-7.81114	353.792	19	2	7.937767	285.335
835	2018	1	-4.617	272.42	34	2	5.883047	224.837
836	2018	1	-6.48333	394.557	17	3	7.808361	262.725
837	2018	1	-7.39365	313.661	20	2	7.515178	245.655
838	2018	1	-7.20009	364.583	20	2	7.820609	262.127
839	2018	0	-4.92368	249.868	27	2	6.349614	181.04
840	2018	1	-5.14502	320.559	29	3	6.399948	186.715
841	2018	0	-4.617	272.42	34	2	5.883047	224.837
842	2018	1	-5.77559	374.622	16	2	7.911587	269.805
843	2018	1	-6.88819	301.882	29	2	6.529027	195.725
844	2018	1	-7.82124	334.567	18	1	7.513873	250.932
845	2018	1	-4.617	272.42	34	2	5.883047	224.837
846	2018	1	-6.88819	301.882	29	2	6.529027	195.725
847	2018	1	-4.87848	263.795	31	3	5.976067	224.208
848	2018	0	-6.88819	301.882	29	2	6.529027	195.725
849	2018	1	-6.88819	301.882	29	2	6.529027	195.725

Table S2 - Anonymized database

850	2018	1	-5.90725	366.045	21	2	7.512705	252.161
851	2018	1	-7.39365	313.661	20	2	7.515178	245.655
852	2018	0	-6.58408	381.708	16	3	7.864085	312.963
853	2018	1	-6.30913	329.647	29	2	6.266621	226.935
854	2018	1	-4.92368	249.868	27	2	6.349614	181.04
855	2018	0	-4.617	272.42	34	2	5.883047	224.837
856	2018	0	-8.27169	305.458	25	2	6.790788	190.623
857	2018	1	-5.90725	366.045	21	2	7.512705	252.161
858	2018	0	-7.39365	313.661	20	2	7.515178	245.655
859	2018	1	-4.28441	244.851	29	2	6.183555	181.364
860	2018	1	-4.4763	261.526	31	2	5.876564	215.879
861	2018	1	-4.617	272.42	34	2	5.883047	224.837
862	2018	0	-6.88819	301.882	29	2	6.529027	195.725
863	2018	0	-6.58408	381.708	16	3	7.864085	312.963
864	2018	1	-4.53398	301.064	30	1	6.283117	206.422
865	2018	0	-4.87848	263.795	31	3	5.976067	224.208
866	2018	1	-7.39365	313.661	20	2	7.515178	245.655
867	2018	1	-4.87848	263.795	31	3	5.976067	224.208
868	2018	0	-4.92368	249.868	27	2	6.349614	181.04
869	2018	1	-6.88819	301.882	29	2	6.529027	195.725
870	2018	1	-6.88819	301.882	29	2	6.529027	195.725
871	2018	1	-4.87848	263.795	31	3	5.976067	224.208
872	2018	0	-5.02796	330.199	33	2	5.989155	220.014
873	2018	1	-5.02796	330.199	33	2	5.989155	220.014
874	2018	1	-6.33545	385.796	20	2	7.653489	281.62
875	2018	1	-7.20009	364.583	20	2	7.820609	262.127
876	2018	1	-4.87848	263.795	31	3	5.976067	224.208
877	2018	0	-7.07599	282.616	29	2	6.611127	164.806
878	2018	0	-4.92368	249.868	27	2	6.349614	181.04
879	2018	0	-7.82124	334.567	18	1	7.513873	250.932
880	2018	0	-5.44999	360.827	25	2	7.2608	247.862
881	2018	0	-7.81114	353.792	19	2	7.937767	285.335
882	2018	0	-6.49495	310.095	24	1	6.916134	351.541
883	2018	1	-5.90725	366.045	21	2	7.512705	252.161
884	2018	0	-7.07599	282.616	29	2	6.611127	164.806
885	2019	1	-3.76051	346.815	47	2	4.898393	504.376
886	2019	0	-7.00074	299.039	36	0	6.096522	389.577
887	2019	1	-4.09345	382.29	43	1	5.095937	500.127
888	2019	1	-5.24159	310.989	43	0	5.287071	440.49
889	2019	0	-4.40974	345.852	46	1	5.137047	529.694
890	2019	0	-6.23996	269.162	35	0	6.072267	432.924
891	2019	1	-3.69078	312.203	44	2	5.240152	492.006
892	2019	1	-5.87555	315.59	40	0	5.694942	516.263
893	2019	1	-3.4318	319.197	46	2	5.039764	515.073
894	2019	0	-4.45918	334.315	37	2	5.523238	511.474
895	2019	1	-5.6137	294.652	41	0	5.426873	543.489
896	2019	1	-8.06668	248.124	28	0	5.879601	501.356
897	2019	1	-5.31593	314.043	36	2	5.66843	508.904
898	2019	1	-4.63667	327.979	38	2	5.431978	566.958
899	2019	1	-5.15019	289.288	45	1	5.376062	499.956

Table S2 - Anonymized database

900	2019	1	-2.48211	400.639	51	2	4.783273	514.754
901	2019	1	-5.45258	304.945	43	0	5.550265	480.654
902	2019	1	-4.29449	321.305	42	1	5.220583	528.529
903	2019	1	-4.05768	386.287	43	1	5.090078	505.107
904	2019	1	-4.64643	311.157	39	1	5.043631	474.124
905	2019	0	-5.47236	416.38	34	1	5.313608	622.153
906	2019	1	-4.4804	331.583	42	1	5.148194	518.009
907	2019	1	-3.3129	348.276	48	1	4.975174	484.098
908	2019	0	-3.88729	336.306	46	1	4.835687	499.207
909	2019	1	-4.47043	303.935	41	1	5.195107	513.713
910	2019	1	-6.16152	322.998	38	0	5.854097	462.293
911	2019	1	-4.46262	322.218	37	2	5.455272	601.429
912	2019	1	-8.35988	217.186	26	0	6.415313	407.724
913	2019	0	-3.9578	317.211	45	1	5.123914	480.404
914	2019	1	-4.47043	303.935	41	1	5.195107	513.713
915	2019	0	-3.93419	340.409	38	3	5.31214	548.711
916	2019	1	-3.3129	348.276	48	1	4.975174	484.098
917	2019	1	-5.6137	294.652	41	0	5.426873	543.489
918	2019	0	-5.24159	310.989	43	0	5.287071	440.49
919	2019	1	-4.75329	310.003	46	0	5.133521	487.816
920	2019	1	-3.49504	313.277	46	2	4.914761	491.934
921	2019	0	-4.20769	318.57	43	3	4.95145	488.611
922	2019	1	-6.59369	250.609	36	0	5.62224	483.596
923	2019	0	-3.75833	320.514	50	1	5.0674	485.061
924	2019	0	-3.87159	318.522	48	1	5.018917	488.66
925	2019	0	-5.11453	303.235	45	0	5.674451	462.749
926	2019	0	-5.87555	315.59	40	0	5.694942	516.263
927	2019	1	-2.68799	351.335	49	2	4.581138	596.635
928	2019	1	-3.4318	319.197	46	2	5.039764	515.073
929	2019	0	-7.65899	238.94	34	0	5.859651	518.333
930	2019	1	-3.3129	348.276	48	1	4.975174	484.098
931	2019	0	-5.87555	315.59	40	0	5.694942	516.263
932	2019	0	-7.65899	238.94	34	0	5.859651	518.333
933	2019	1	-3.3129	348.276	48	1	4.975174	484.098
934	2019	1	-5.6137	294.652	41	0	5.426873	543.489
935	2019	1	-5.84955	308.039	32	0	5.886017	561.172
936	2019	1	-4.47043	303.935	41	1	5.195107	513.713
937	2019	1	-7.65899	238.94	34	0	5.859651	518.333
938	2019	1	-3.9578	317.211	45	1	5.123914	480.404
939	2019	1	-6.05771	256.914	35	2	5.984195	423.871
940	2019	1	-4.4831	330.103	38	2	5.392603	562.945
941	2019	1	-5.19449	358.12	38	1	5.838659	552.653
942	2019	1	-3.87159	318.522	48	1	5.018917	488.66
943	2019	0	-5.45258	304.945	43	0	5.550265	480.654
944	2019	0	-4.45918	334.315	37	2	5.523238	511.474
945	2019	1	-5.6137	294.652	41	0	5.426873	543.489
946	2019	0	-4.72295	327.698	44	1	5.411894	477.704
947	2019	1	-3.3129	348.276	48	1	4.975174	484.098
948	2019	1	-4.7944	356.626	44	1	5.006661	556.198
949	2019	1	-3.3129	348.276	48	1	4.975174	484.098

Table S2 - Anonymized database

950	2019	0	-6.33396	266.799	37	1	6.01164	406.389
951	2019	0	-5.45258	304.945	43	0	5.550265	480.654
952	2019	0	-5.31593	314.043	36	2	5.66843	508.904
953	2019	1	-5.24159	310.989	43	0	5.287071	440.49
954	2019	0	-3.4318	319.197	46	2	5.039764	515.073
955	2019	0	-6.43747	269.529	36	0	5.95026	447.542
956	2019	0	-5.45258	304.945	43	0	5.550265	480.654
957	2019	0	-5.94688	258.055	36	2	5.751378	388.757
958	2019	1	-5.47236	416.38	34	1	5.313608	622.153
959	2019	1	-7.33269	184.695	33	0	5.911776	469.051
960	2019	0	-4.30589	344.709	43	2	5.10428	508.747
961	2019	0	-4.45918	334.315	37	2	5.523238	511.474
962	2019	0	-4.91638	309.095	41	2	5.392417	497.162
963	2019	1	-5.94608	339.673	38	0	5.682072	507.493
964	2019	1	-3.3129	348.276	48	1	4.975174	484.098
965	2019	1	-7.00074	299.039	36	0	6.096522	389.577
966	2019	0	-4.45918	334.315	37	2	5.523238	511.474
967	2019	1	-3.93419	340.409	38	3	5.31214	548.711
968	2019	0	-4.47043	303.935	41	1	5.195107	513.713
969	2019	1	-4.7944	356.626	44	1	5.006661	556.198
970	2019	1	-3.93111	324.98	44	2	5.178228	540.026
971	2019	1	-3.51463	367.233	49	2	5.232931	532.969
972	2019	0	-6.41129	269.816	36	0	5.565534	563.718
973	2019	1	-4.7944	356.626	44	1	5.006661	556.198
974	2019	1	-3.3129	348.276	48	1	4.975174	484.098
975	2019	1	-4.33927	330.623	43	2	5.00001	502.706
976	2019	0	-5.84955	308.039	32	0	5.886017	561.172
977	2019	1	-3.3129	348.276	48	1	4.975174	484.098
978	2019	0	-3.63914	334.62	46	2	4.63291	511.473
979	2019	1	-4.47043	303.935	41	1	5.195107	513.713
980	2019	1	-5.45258	304.945	43	0	5.550265	480.654
981	2019	1	-3.49504	313.277	46	2	4.914761	491.934
982	2019	1	-3.93419	340.409	38	3	5.31214	548.711
983	2019	0	-4.45186	362.907	39	1	5.173466	572.828
984	2019	0	-5.45258	304.945	43	0	5.550265	480.654
985	2019	1	-2.90229	317.849	52	2	4.930131	537.539
986	2019	0	-3.87159	318.522	48	1	5.018917	488.66
987	2019	1	-5.45258	304.945	43	0	5.550265	480.654
988	2019	1	-4.33927	330.623	43	2	5.00001	502.706
989	2019	1	-6.16152	322.998	38	0	5.854097	462.293
990	2019	1	-4.6838	319.487	38	2	5.346952	494.65
991	2019	0	-3.50415	318.524	53	1	5.022956	490.892
992	2019	1	-4.4804	331.583	42	1	5.148194	518.009
993	2019	1	-5.45258	304.945	43	0	5.550265	480.654
994	2019	1	-2.65456	369.101	50	2	4.613546	552.573
995	2019	1	-6.05771	256.914	35	2	5.984195	423.871
996	2019	1	-3.4318	319.197	46	2	5.039764	515.073
997	2019	1	-4.55829	313.129	39	1	4.963728	501.908
998	2019	1	-3.3129	348.276	48	1	4.975174	484.098
999	2019	1	-4.33927	330.623	43	2	5.00001	502.706

Table S2 - Anonymized database

1000	2019	1	-4.70182	292.193	38	2	5.359046	482.036
1001	2019	0	-2.08757	381.085	50	3	4.534825	607.024
1002	2019	1	-4.63667	327.979	38	2	5.431978	566.958
1003	2019	1	-4.38503	341.272	44	1	5.119593	519.767
1004	2019	0	-4.38503	341.272	44	1	5.119593	519.767
1005	2019	1	-4.91638	309.095	41	2	5.392417	497.162
1006	2019	0	-7.76235	277.011	30	0	6.483886	487.437
1007	2019	1	-6.17124	287.268	41	0	5.625112	492.719
1008	2019	1	-4.7944	356.626	44	1	5.006661	556.198
1009	2019	1	-4.19045	320.987	41	2	5.221517	501.406
1010	2019	1	-4.20769	318.57	43	3	4.95145	488.611
1011	2019	0	-4.29449	321.305	42	1	5.220583	528.529
1012	2019	0	-2.08757	381.085	50	3	4.534825	607.024
1013	2019	0	-5.6137	294.652	41	0	5.426873	543.489
1014	2019	0	-6.55951	299.438	36	0	6.000892	471.31
1015	2019	1	-3.69078	312.203	44	2	5.240152	492.006
1016	2019	0	-7.00074	299.039	36	0	6.096522	389.577
1017	2019	1	-4.7944	356.626	44	1	5.006661	556.198
1018	2019	1	-4.38503	341.272	44	1	5.119593	519.767
1019	2019	1	-4.55829	313.129	39	1	4.963728	501.908
1020	2019	1	-5.87555	315.59	40	0	5.694942	516.263
1021	2019	1	-4.7944	356.626	44	1	5.006661	556.198
1022	2019	1	-4.27204	308.602	41	2	5.232071	467.972
1023	2019	0	-4.75329	310.003	46	0	5.133521	487.816
1024	2019	0	-6.17492	336.916	38	0	5.527432	554.077
1025	2019	0	-5.31593	314.043	36	2	5.66843	508.904
1026	2019	1	-3.5203	317.732	49	1	4.986291	463.512
1027	2019	0	-3.55612	376.155	48	2	5.019857	559.01
1028	2019	1	-6.59369	250.609	36	0	5.62224	483.596
1029	2019	1	-3.55705	304.823	47	2	5.08267	474.252
1030	2019	0	-4.47043	303.935	41	1	5.195107	513.713
1031	2019	1	-2.66879	342.33	50	2	4.614109	571.654
1032	2019	0	-4.32788	320.592	41	4	4.962579	492.906
1033	2019	1	-5.84955	308.039	32	0	5.886017	561.172
1034	2019	1	-5.45258	304.945	43	0	5.550265	480.654
1035	2019	1	-6.16152	322.998	38	0	5.854097	462.293
1036	2019	1	-3.91564	347.476	38	3	5.476024	565.449
1037	2019	1	-8.35988	217.186	26	0	6.415313	407.724
1038	2019	1	-4.46262	322.218	37	2	5.455272	601.429
1039	2019	0	-2.68799	351.335	49	2	4.581138	596.635
1040	2019	1	-3.93111	324.98	44	2	5.178228	540.026
1041	2019	0	-4.41025	366.521	40	1	4.98413	567.192
1042	2019	0	-4.45918	334.315	37	2	5.523238	511.474
1043	2019	1	-4.05768	386.287	43	1	5.090078	505.107
1044	2019	1	-5.59907	348.703	37	0	5.467555	576.059
1045	2019	0	-7.33269	184.695	33	0	5.911776	469.051
1046	2019	1	-4.4831	330.103	38	2	5.392603	562.945
1047	2019	0	-4.061	330.015	41	2	5.321926	523.361
1048	2019	1	-4.05768	386.287	43	1	5.090078	505.107
1049	2019	0	-8.35988	217.186	26	0	6.415313	407.724

Table S2 - Anonymized database

1050	2019	1	-3.91564	347.476	38	3	5.476024	565.449
1051	2019	1	-6.09832	362.381	32	0	6.020559	542.213
1052	2019	0	-7.65899	238.94	34	0	5.859651	518.333
1053	2019	1	-4.41025	366.521	40	1	4.98413	567.192
1054	2019	0	-5.40087	309.561	36	0	5.442493	484.634
1055	2019	1	-4.7944	356.626	44	1	5.006661	556.198
1056	2019	0	-7.65899	238.94	34	0	5.859651	518.333
1057	2019	1	-3.95811	331.068	42	2	5.278865	566.068
1058	2019	1	-4.4831	330.103	38	2	5.392603	562.945
1059	2019	0	-7.65899	238.94	34	0	5.859651	518.333
1060	2019	1	-3.93111	324.98	44	2	5.178228	540.026
1061	2019	1	-5.45258	304.945	43	0	5.550265	480.654
1062	2019	1	-4.4804	331.583	42	1	5.148194	518.009
1063	2019	0	-3.96623	304.299	52	1	5.140466	459.523
1064	2019	0	-7.00074	299.039	36	0	6.096522	389.577
1065	2019	1	-3.3129	348.276	48	1	4.975174	484.098
1066	2019	0	-6.59369	250.609	36	0	5.62224	483.596
1067	2019	1	-4.45186	362.907	39	1	5.173466	572.828
1068	2019	1	-6.59369	250.609	36	0	5.62224	483.596
1069	2019	0	-3.49504	313.277	46	2	4.914761	491.934
1070	2019	0	-4.061	330.015	41	2	5.321926	523.361
1071	2019	1	-3.49504	313.277	46	2	4.914761	491.934
1072	2019	1	-3.34624	318.067	53	2	4.98291	495.029
1073	2019	0	-7.82548	322.886	28	0	5.510701	528.394
1074	2019	0	-4.66312	326.279	38	2	5.426116	570.167
1075	2019	0	-7.00074	299.039	36	0	6.096522	389.577
1076	2019	1	-4.96542	383.416	38	1	5.073697	526.242
1077	2019	1	-3.3129	348.276	48	1	4.975174	484.098
1078	2019	1	-4.46704	342.849	36	2	5.522096	511.449
1079	2019	0	-6.23996	269.162	35	0	6.072267	432.924
1080	2019	1	-3.50415	318.524	53	1	5.022956	490.892
1081	2019	0	-7.33269	184.695	33	0	5.911776	469.051
1082	2019	1	-2.32977	366.025	50	2	4.555181	616.989
1083	2019	0	-3.18124	322	53	1	4.868373	472.559
1084	2019	0	-6.33396	266.799	37	1	6.01164	406.389
1085	2019	0	-5.31593	314.043	36	2	5.66843	508.904
1086	2019	1	-4.23208	365.71	43	1	5.194001	519.539
1087	2019	0	-7.00074	299.039	36	0	6.096522	389.577
1088	2019	1	-5.47236	416.38	34	1	5.313608	622.153
1089	2019	1	-5.45258	304.945	43	0	5.550265	480.654
1090	2019	0	-7.76235	277.011	30	0	6.483886	487.437
1091	2019	1	-3.5203	317.732	49	1	4.986291	463.512
1092	2019	0	-4.32788	320.592	41	4	4.962579	492.906
1093	2019	1	-3.4318	319.197	46	2	5.039764	515.073
1094	2019	1	-4.6838	319.487	38	2	5.346952	494.65
1095	2019	1	-3.93111	324.98	44	2	5.178228	540.026
1096	2019	0	-7.33269	184.695	33	0	5.911776	469.051
1097	2019	0	-7.82548	322.886	28	0	5.510701	528.394
1098	2019	1	-4.53618	313.966	39	2	5.291598	478.142
1099	2019	1	-4.75329	310.003	46	0	5.133521	487.816

Table S2 - Anonymized database

1100	2019	1	-5.31593	314.043	36	2	5.66843	508.904
1101	2019	1	-4.48699	335.255	43	2	5.329501	560.741
1102	2019	0	-3.12136	335.365	49	1	4.576384	540.209
1103	2019	1	-4.07084	313.487	45	1	5.190497	514.185
1104	2019	1	-4.05765	338.255	40	1	5.013981	520.512
1105	2019	1	-4.33927	330.623	43	2	5.00001	502.706
1106	2019	1	-3.49504	313.277	46	2	4.914761	491.934
1107	2019	0	-6.33396	266.799	37	1	6.01164	406.389
1108	2019	1	-4.27204	308.602	41	2	5.232071	467.972
1109	2019	1	-3.66295	333.767	45	2	4.952486	513.223
1110	2019	1	-3.86198	325.153	44	1	4.846032	504.883
1111	2019	1	-5.5641	310.64	43	0	5.404042	506.256
1112	2019	0	-7.82548	322.886	28	0	5.510701	528.394
1113	2019	1	-3.4318	319.197	46	2	5.039764	515.073
1114	2019	1	-3.34624	318.067	53	2	4.98291	495.029
1115	2019	1	-4.70182	292.193	38	2	5.359046	482.036
1116	2019	0	-7.00074	299.039	36	0	6.096522	389.577
1117	2019	1	-4.19045	320.987	41	2	5.221517	501.406
1118	2019	0	-3.32877	405.774	47	2	5.007678	505.575
1119	2019	1	-8.35988	217.186	26	0	6.415313	407.724
1120	2019	0	-3.88729	336.306	46	1	4.835687	499.207
1121	2019	1	-4.45186	362.907	39	1	5.173466	572.828
1122	2019	1	-4.55829	313.129	39	1	4.963728	501.908
1123	2019	1	-3.9488	354.511	46	1	5.098173	568.326
1124	2019	1	-4.2633	325.812	40	2	5.236413	489.847
1125	2019	1	-5.45258	304.945	43	0	5.550265	480.654
1126	2019	1	-6.38046	262.27	36	0	5.918594	437.236
1127	2019	0	-3.93419	340.409	38	3	5.31214	548.711
1128	2019	0	-4.91638	309.095	41	2	5.392417	497.162
1129	2019	0	-4.45918	334.315	37	2	5.523238	511.474
1130	2019	1	-4.38503	341.272	44	1	5.119593	519.767
1131	2019	0	-3.96623	304.299	52	1	5.140466	459.523
1132	2019	1	-5.31593	314.043	36	2	5.66843	508.904
1133	2019	0	-5.59907	348.703	37	0	5.467555	576.059
1134	2019	1	-4.45186	362.907	39	1	5.173466	572.828
1135	2019	1	-4.23208	365.71	43	1	5.194001	519.539
1136	2019	0	-7.76235	277.011	30	0	6.483886	487.437
1137	2019	1	-5.45258	304.945	43	0	5.550265	480.654
1138	2019	1	-4.23208	365.71	43	1	5.194001	519.539
1139	2019	1	-7.82548	322.886	28	0	5.510701	528.394
1140	2019	1	-8.35988	217.186	26	0	6.415313	407.724
1141	2019	1	-4.55829	313.129	39	1	4.963728	501.908
1142	2019	1	-4.05768	386.287	43	1	5.090078	505.107
1143	2019	1	-3.95811	331.068	42	2	5.278865	566.068
1144	2019	1	-3.5203	317.732	49	1	4.986291	463.512
1145	2019	1	-3.55705	304.823	47	2	5.08267	474.252
1146	2019	1	-3.51463	367.233	49	2	5.232931	532.969
1147	2019	0	-4.79401	322.572	45	1	5.279937	571.487
1148	2019	1	-8.35988	217.186	26	0	6.415313	407.724
1149	2019	0	-3.4318	319.197	46	2	5.039764	515.073

Table S2 - Anonymized database

1150	2019	0	-4.55829	313.129	39	1	4.963728	501.908
1151	2019	0	-6.36062	318.007	40	0	5.700947	458.444
1152	2019	1	-6.41129	269.816	36	0	5.565534	563.718
1153	2019	0	-7.33269	184.695	33	0	5.911776	469.051
1154	2019	0	-3.69199	329.98	44	2	4.822085	483.68
1155	2019	1	-3.3129	348.276	48	1	4.975174	484.098
1156	2019	1	-4.7944	356.626	44	1	5.006661	556.198
1157	2019	1	-3.3129	348.276	48	1	4.975174	484.098
1158	2019	1	-3.55612	376.155	48	2	5.019857	559.01
1159	2019	1	-6.5841	298.407	37	0	5.971897	382.27
1160	2019	1	-4.33927	330.623	43	2	5.00001	502.706
1161	2019	1	-3.96623	304.299	52	1	5.140466	459.523
1162	2019	1	-3.69199	329.98	44	2	4.822085	483.68
1163	2019	1	-3.93419	340.409	38	3	5.31214	548.711
1164	2019	1	-3.4318	319.197	46	2	5.039764	515.073
1165	2019	0	-7.00074	299.039	36	0	6.096522	389.577
1166	2019	1	-4.7944	356.626	44	1	5.006661	556.198
1167	2019	1	-5.45258	304.945	43	0	5.550265	480.654
1168	2019	1	-6.17124	287.268	41	0	5.625112	492.719
1169	2019	1	-4.45186	362.907	39	1	5.173466	572.828
1170	2019	1	-4.27204	308.602	41	2	5.232071	467.972
1171	2019	1	-4.55829	313.129	39	1	4.963728	501.908
1172	2019	1	-4.55829	313.129	39	1	4.963728	501.908
1173	2019	1	-4.55829	313.129	39	1	4.963728	501.908
1174	2019	1	-3.93419	340.409	38	3	5.31214	548.711
1175	2019	0	-6.13421	286.197	41	0	5.574049	513.422
1176	2019	1	-2.90229	317.849	52	2	4.930131	537.539
1177	2019	0	-4.29449	321.305	42	1	5.220583	528.529
1178	2019	0	-2.68799	351.335	49	2	4.581138	596.635
1179	2019	1	-5.45258	304.945	43	0	5.550265	480.654
1180	2019	1	-4.33927	330.623	43	2	5.00001	502.706
1181	2019	1	-3.87159	318.522	48	1	5.018917	488.66
1182	2019	1	-3.55612	376.155	48	2	5.019857	559.01
1183	2019	0	-5.24159	310.989	43	0	5.287071	440.49
1184	2019	0	-7.82548	322.886	28	0	5.510701	528.394
1185	2019	1	-3.3129	348.276	48	1	4.975174	484.098
1186	2019	0	-5.24159	310.989	43	0	5.287071	440.49
1187	2019	1	-2.81745	342.717	51	2	4.929575	574.121
1188	2019	1	-3.69078	312.203	44	2	5.240152	492.006
1189	2019	1	-6.9887	274.518	39	0	6.269623	498.654
1190	2019	1	-3.55612	376.155	48	2	5.019857	559.01
1191	2019	1	-3.3129	348.276	48	1	4.975174	484.098
1192	2019	1	-4.27204	308.602	41	2	5.232071	467.972
1193	2019	0	-8.35988	217.186	26	0	6.415313	407.724
1194	2019	1	-3.75855	321.524	48	1	5.03645	476.372
1195	2019	1	-5.24159	310.989	43	0	5.287071	440.49
1196	2019	1	-5.6137	294.652	41	0	5.426873	543.489
1197	2019	1	-5.19449	358.12	38	1	5.838659	552.653
1198	2019	0	-3.9578	317.211	45	1	5.123914	480.404
1199	2019	0	-3.96623	304.299	52	1	5.140466	459.523

Table S2 - Anonymized database

1200	2019	1	-4.45186	362.907	39	1	5.173466	572.828
1201	2019	1	-4.19045	320.987	41	2	5.221517	501.406
1202	2019	1	-3.5203	317.732	49	1	4.986291	463.512
1203	2019	0	-7.82548	322.886	28	0	5.510701	528.394
1204	2019	1	-3.51463	367.233	49	2	5.232931	532.969
1205	2019	0	-3.6911	330.824	47	1	4.999539	589.911
1206	2019	1	-3.88729	336.306	46	1	4.835687	499.207
1207	2019	1	-5.87555	315.59	40	0	5.694942	516.263
1208	2019	1	-4.17502	356.028	45	2	5.06267	509.974
1209	2019	1	-4.14487	326.941	42	2	5.308244	534.958
1210	2019	0	-4.45186	362.907	39	1	5.173466	572.828
1211	2019	1	-2.90229	317.849	52	2	4.930131	537.539
1212	2019	1	-3.15825	340.806	49	3	4.469025	498.859
1213	2019	1	-3.3129	348.276	48	1	4.975174	484.098
1214	2019	1	-5.45258	304.945	43	0	5.550265	480.654
1215	2019	1	-3.28244	324.213	43	2	4.698999	534.732
1216	2019	1	-5.09752	312.814	41	0	5.247892	460.052
1217	2019	1	-3.50415	318.524	53	1	5.022956	490.892
1218	2019	1	-4.07084	313.487	45	1	5.190497	514.185
1219	2019	0	-5.6137	294.652	41	0	5.426873	543.489
1220	2019	1	-3.15825	340.806	49	3	4.469025	498.859
1221	2019	0	-7.00074	299.039	36	0	6.096522	389.577
1222	2019	0	-5.31249	332.657	37	1	5.727701	529.991
1223	2019	1	-4.09345	382.29	43	1	5.095937	500.127
1224	2019	1	-5.45258	304.945	43	0	5.550265	480.654
1225	2019	1	-3.93111	324.98	44	2	5.178228	540.026
1226	2019	1	-6.17492	336.916	38	0	5.527432	554.077
1227	2019	1	-6.09832	362.381	32	0	6.020559	542.213
1228	2019	1	-4.46704	342.849	36	2	5.522096	511.449
1229	2019	1	-3.69078	312.203	44	2	5.240152	492.006
1230	2019	0	-4.7944	356.626	44	1	5.006661	556.198
1231	2019	1	-4.45186	362.907	39	1	5.173466	572.828
1232	2019	0	-3.75833	320.514	50	1	5.0674	485.061
1233	2019	0	-2.67178	364.946	50	2	4.603849	583.92
1234	2019	0	-4.7944	356.626	44	1	5.006661	556.198
1235	2019	1	-5.31249	332.657	37	1	5.727701	529.991
1236	2019	1	-3.96623	304.299	52	1	5.140466	459.523
1237	2019	1	-3.66295	333.767	45	2	4.952486	513.223
1238	2019	0	-3.12136	335.365	49	1	4.576384	540.209
1239	2019	1	-4.45186	362.907	39	1	5.173466	572.828
1240	2019	1	-3.3129	348.276	48	1	4.975174	484.098
1241	2019	1	-4.4804	331.583	42	1	5.148194	518.009
1242	2019	1	-5.45258	304.945	43	0	5.550265	480.654
1243	2019	1	-6.13421	286.197	41	0	5.574049	513.422
1244	2019	0	-8.35988	217.186	26	0	6.415313	407.724
1245	2019	1	-4.05768	386.287	43	1	5.090078	505.107
1246	2019	1	-4.4831	330.103	38	2	5.392603	562.945
1247	2019	1	-6.17124	287.268	41	0	5.625112	492.719
1248	2019	1	-3.91564	347.476	38	3	5.476024	565.449
1249	2019	1	-4.061	330.015	41	2	5.321926	523.361

Table S2 - Anonymized database

1250	2019	1	-5.45258	304.945	43	0	5.550265	480.654
1251	2019	0	-2.62644	335.621	49	3	4.628169	565.458
1252	2019	1	-3.55612	376.155	48	2	5.019857	559.01
1253	2019	0	-3.75833	320.514	50	1	5.0674	485.061
1254	2019	1	-5.84955	308.039	32	0	5.886017	561.172
1255	2019	1	-2.90229	317.849	52	2	4.930131	537.539
1256	2019	1	-3.3129	348.276	48	1	4.975174	484.098
1257	2019	1	-6.24288	391.685	38	0	5.22349	575.969
1258	2019	1	-3.3129	348.276	48	1	4.975174	484.098
1259	2019	1	-4.45918	334.315	37	2	5.523238	511.474
1260	2019	1	-5.40563	339	38	1	5.13232	519.911
1261	2019	0	-4.40974	345.852	46	1	5.137047	529.694
1262	2019	1	-5.15019	289.288	45	1	5.376062	499.956
1263	2019	1	-6.05771	256.914	35	2	5.984195	423.871
1264	2019	0	-4.7944	356.626	44	1	5.006661	556.198
1265	2019	1	-6.59369	250.609	36	0	5.62224	483.596
1266	2019	1	-3.87159	318.522	48	1	5.018917	488.66
1267	2019	0	-5.94688	258.055	36	2	5.751378	388.757
1268	2019	0	-5.59907	348.703	37	0	5.467555	576.059
1269	2019	1	-2.90229	317.849	52	2	4.930131	537.539
1270	2019	1	-3.49504	313.277	46	2	4.914761	491.934
1271	2019	1	-3.3129	348.276	48	1	4.975174	484.098
1272	2019	1	-3.87159	318.522	48	1	5.018917	488.66
1273	2019	1	-3.4318	319.197	46	2	5.039764	515.073
1274	2019	0	-3.76051	346.815	47	2	4.898393	504.376
1275	2019	0	-7.33269	184.695	33	0	5.911776	469.051
1276	2019	1	-4.55829	313.129	39	1	4.963728	501.908
1277	2019	0	-5.5641	310.64	43	0	5.404042	506.256
1278	2019	1	-5.24159	310.989	43	0	5.287071	440.49
1279	2019	1	-3.86198	325.153	44	1	4.846032	504.883
1280	2019	1	-6.9887	274.518	39	0	6.269623	498.654
1281	2019	0	-4.38503	341.272	44	1	5.119593	519.767
1282	2019	1	-4.07084	313.487	45	1	5.190497	514.185
1283	2019	0	-6.43747	269.529	36	0	5.95026	447.542
1284	2019	1	-4.91638	309.095	41	2	5.392417	497.162
1285	2019	1	-8.35988	217.186	26	0	6.415313	407.724
1286	2019	0	-3.75833	320.514	50	1	5.0674	485.061
1287	2019	1	-6.09832	362.381	32	0	6.020559	542.213
1288	2019	0	-7.00074	299.039	36	0	6.096522	389.577
1289	2019	1	-5.59907	348.703	37	0	5.467555	576.059
1290	2019	0	-6.5841	298.407	37	0	5.971897	382.27
1291	2019	1	-3.69078	312.203	44	2	5.240152	492.006
1292	2019	1	-4.46704	342.849	36	2	5.522096	511.449
1293	2019	1	-6.17124	287.268	41	0	5.625112	492.719
1294	2019	0	-4.45918	334.315	37	2	5.523238	511.474
1295	2019	1	-5.45258	304.945	43	0	5.550265	480.654
1296	2019	0	-4.41796	375.687	39	1	5.141097	548.239
1297	2019	1	-5.45258	304.945	43	0	5.550265	480.654
1298	2019	1	-4.55829	313.129	39	1	4.963728	501.908
1299	2019	0	-8.35988	217.186	26	0	6.415313	407.724

Table S2 - Anonymized database

1300	2019	1	-2.90229	317.849	52	2	4.930131	537.539
1301	2019	0	-5.6137	294.652	41	0	5.426873	543.489
1302	2019	0	-4.91638	309.095	41	2	5.392417	497.162
1303	2019	1	-3.93111	324.98	44	2	5.178228	540.026
1304	2019	0	-6.16152	322.998	38	0	5.854097	462.293
1305	2019	0	-5.45258	304.945	43	0	5.550265	480.654
1306	2019	0	-4.33927	330.623	43	2	5.00001	502.706
1307	2019	0	-3.87159	318.522	48	1	5.018917	488.66
1308	2019	1	-3.23505	292.894	50	2	5.061666	468.763
1309	2019	1	-4.30589	344.709	43	2	5.10428	508.747
1310	2019	0	-7.00074	299.039	36	0	6.096522	389.577
1311	2019	0	-7.00074	299.039	36	0	6.096522	389.577
1312	2019	1	-5.94608	339.673	38	0	5.682072	507.493
1313	2019	1	-5.45258	304.945	43	0	5.550265	480.654
1314	2019	1	-5.6137	294.652	41	0	5.426873	543.489
1315	2019	0	-6.23256	263.12	35	1	5.8627	383.889
1316	2019	1	-3.69078	312.203	44	2	5.240152	492.006
1317	2019	1	-5.31593	314.043	36	2	5.66843	508.904
1318	2019	0	-7.00074	299.039	36	0	6.096522	389.577
1319	2019	1	-8.35988	217.186	26	0	6.415313	407.724
1320	2019	1	-4.48699	335.255	43	2	5.329501	560.741
1321	2019	1	-5.5641	310.64	43	0	5.404042	506.256
1322	2019	0	-2.48211	400.639	51	2	4.783273	514.754
1323	2019	1	-3.5203	317.732	49	1	4.986291	463.512
1324	2019	1	-8.06668	248.124	28	0	5.879601	501.356
1325	2019	1	-4.19045	320.987	41	2	5.221517	501.406
1326	2019	1	-3.91564	347.476	38	3	5.476024	565.449
1327	2019	1	-4.79401	322.572	45	1	5.279937	571.487
1328	2019	1	-4.38503	341.272	44	1	5.119593	519.767
1329	2019	1	-5.31593	314.043	36	2	5.66843	508.904
1330	2019	0	-4.20769	318.57	43	3	4.95145	488.611
1331	2019	0	-4.47043	303.935	41	1	5.195107	513.713
1332	2019	1	-4.061	330.015	41	2	5.321926	523.361
1333	2019	1	-2.67178	364.946	50	2	4.603849	583.92
1334	2019	1	-6.16152	322.998	38	0	5.854097	462.293
1335	2019	0	-3.96623	304.299	52	1	5.140466	459.523
1336	2019	0	-7.33269	184.695	33	0	5.911776	469.051
1337	2019	1	-6.43747	269.529	36	0	5.95026	447.542
1338	2019	0	-7.65899	238.94	34	0	5.859651	518.333
1339	2019	0	-7.00074	299.039	36	0	6.096522	389.577
1340	2019	1	-4.45186	362.907	39	1	5.173466	572.828
1341	2019	1	-2.90229	317.849	52	2	4.930131	537.539
1342	2019	0	-7.82548	322.886	28	0	5.510701	528.394
1343	2019	1	-3.93111	324.98	44	2	5.178228	540.026
1344	2019	0	-4.4831	330.103	38	2	5.392603	562.945
1345	2019	1	-3.93419	340.409	38	3	5.31214	548.711
1346	2019	0	-3.66295	333.767	45	2	4.952486	513.223
1347	2019	0	-3.69199	329.98	44	2	4.822085	483.68
1348	2019	1	-6.13421	286.197	41	0	5.574049	513.422
1349	2019	1	-3.9578	317.211	45	1	5.123914	480.404

Table S2 - Anonymized database

1350	2019	0	-3.63914	334.62	46	2	4.63291	511.473
1351	2019	1	-4.45186	362.907	39	1	5.173466	572.828
1352	2019	1	-5.84955	308.039	32	0	5.886017	561.172
1353	2019	1	-4.19045	320.987	41	2	5.221517	501.406
1354	2019	1	-4.55829	313.129	39	1	4.963728	501.908
1355	2019	1	-3.93111	324.98	44	2	5.178228	540.026
1356	2019	1	-4.45186	362.907	39	1	5.173466	572.828
1357	2019	1	-4.27204	308.602	41	2	5.232071	467.972
1358	2019	0	-7.00074	299.039	36	0	6.096522	389.577
1359	2019	1	-5.59907	348.703	37	0	5.467555	576.059
1360	2019	1	-4.4804	331.583	42	1	5.148194	518.009
1361	2019	1	-3.87159	318.522	48	1	5.018917	488.66
1362	2019	0	-7.76235	277.011	30	0	6.483886	487.437
1363	2019	1	-7.33269	184.695	33	0	5.911776	469.051
1364	2019	1	-5.24159	310.989	43	0	5.287071	440.49
1365	2019	1	-3.96623	304.299	52	1	5.140466	459.523
1366	2019	1	-4.57584	303.157	40	2	5.300677	463.864
1367	2019	1	-2.67178	364.946	50	2	4.603849	583.92
1368	2019	1	-6.59369	250.609	36	0	5.62224	483.596
1369	2019	0	-7.65899	238.94	34	0	5.859651	518.333
1370	2019	0	-5.6137	294.652	41	0	5.426873	543.489
1371	2019	0	-4.061	330.015	41	2	5.321926	523.361
1372	2019	0	-3.96623	304.299	52	1	5.140466	459.523
1373	2019	1	-6.55951	299.438	36	0	6.000892	471.31
1374	2019	0	-5.40087	309.561	36	0	5.442493	484.634
1375	2019	0	-5.45258	304.945	43	0	5.550265	480.654
1376	2019	1	-4.45186	362.907	39	1	5.173466	572.828
1377	2019	1	-4.33927	330.623	43	2	5.00001	502.706
1378	2019	1	-4.30589	344.709	43	2	5.10428	508.747
1379	2019	1	-4.07084	313.487	45	1	5.190497	514.185
1380	2019	0	-7.00074	299.039	36	0	6.096522	389.577
1381	2019	0	-7.65899	238.94	34	0	5.859651	518.333
1382	2019	1	-4.47043	303.935	41	1	5.195107	513.713
1383	2019	1	-8.06668	248.124	28	0	5.879601	501.356
1384	2019	0	-7.82548	322.886	28	0	5.510701	528.394
1385	2019	1	-3.51463	367.233	49	2	5.232931	532.969
1386	2019	0	-3.75833	320.514	50	1	5.0674	485.061
1387	2019	1	-2.81745	342.717	51	2	4.929575	574.121
1388	2019	1	-4.45186	362.907	39	1	5.173466	572.828
1389	2019	0	-6.41129	269.816	36	0	5.565534	563.718
1390	2019	1	-7.82548	322.886	28	0	5.510701	528.394
1391	2019	0	-8.35988	217.186	26	0	6.415313	407.724
1392	2019	0	-5.84955	308.039	32	0	5.886017	561.172
1393	2019	0	-2.67178	364.946	50	2	4.603849	583.92
1394	2019	1	-6.9887	274.518	39	0	6.269623	498.654
1395	2019	1	-3.86198	325.153	44	1	4.846032	504.883
1396	2019	0	-2.08757	381.085	50	3	4.534825	607.024
1397	2019	0	-2.67178	364.946	50	2	4.603849	583.92
1398	2019	0	-5.40563	339	38	1	5.13232	519.911
1399	2019	1	-3.4318	319.197	46	2	5.039764	515.073

Table S2 - Anonymized database

1400	2019	0	-4.40974	345.852	46	1	5.137047	529.694
1401	2019	0	-6.43747	269.529	36	0	5.95026	447.542
1402	2019	1	-5.45258	304.945	43	0	5.550265	480.654
1403	2019	1	-4.66312	326.279	38	2	5.426116	570.167
1404	2019	1	-4.79401	322.572	45	1	5.279937	571.487
1405	2019	1	-3.28244	324.213	43	2	4.698999	534.732
1406	2019	1	-5.31593	314.043	36	2	5.66843	508.904
1407	2019	1	-4.4831	330.103	38	2	5.392603	562.945
1408	2019	0	-3.55705	304.823	47	2	5.08267	474.252
1409	2019	1	-3.91564	347.476	38	3	5.476024	565.449
1410	2019	1	-4.05765	338.255	40	1	5.013981	520.512
1411	2019	1	-3.55705	304.823	47	2	5.08267	474.252
1412	2019	1	-6.59369	250.609	36	0	5.62224	483.596
1413	2019	1	-5.47236	416.38	34	1	5.313608	622.153
1414	2019	1	-5.31249	332.657	37	1	5.727701	529.991
1415	2019	1	-5.31593	314.043	36	2	5.66843	508.904
1416	2019	1	-4.19045	320.987	41	2	5.221517	501.406
1417	2019	1	-4.41025	366.521	40	1	4.98413	567.192
1418	2019	1	-3.93111	324.98	44	2	5.178228	540.026
1419	2019	1	-3.3129	348.276	48	1	4.975174	484.098
1420	2019	1	-3.55705	304.823	47	2	5.08267	474.252
1421	2019	1	-5.19449	358.12	38	1	5.838659	552.653
1422	2019	1	-4.66312	326.279	38	2	5.426116	570.167
1423	2019	1	-3.5203	317.732	49	1	4.986291	463.512
1424	2019	0	-5.84955	308.039	32	0	5.886017	561.172
1425	2019	0	-4.91638	309.095	41	2	5.392417	497.162
1426	2019	1	-6.17124	287.268	41	0	5.625112	492.719
1427	2019	1	-2.67178	364.946	50	2	4.603849	583.92
1428	2019	1	-4.7944	356.626	44	1	5.006661	556.198
1429	2019	1	-3.76051	346.815	47	2	4.898393	504.376

Table S2 - Anonymized database

gdd	dd_rain	bioc02	bioc03	bioc04	bioc05	bioc06	bioc07	bioc08
2635.501	4.419355	11.34874	0.269688	9.466324	30.462	-11.619	42.081	20.89842
2635.501	4.419355	11.34874	0.269688	9.466324	30.462	-11.619	42.081	20.89842
2726.074	4.775862	11.58785	0.276413	9.407972	30.95929	-10.963	41.92229	21.63071
2726.074	4.775862	11.58785	0.276413	9.407972	30.95929	-10.963	41.92229	21.63071
2726.074	4.775862	11.58785	0.276413	9.407972	30.95929	-10.963	41.92229	21.63071
2726.074	4.775862	11.58785	0.276413	9.407972	30.95929	-10.963	41.92229	21.63071
2726.074	4.775862	11.58785	0.276413	9.407972	30.95929	-10.963	41.92229	21.63071
2726.074	4.775862	11.58785	0.276413	9.407972	30.95929	-10.963	41.92229	21.63071
2726.074	4.775862	11.58785	0.276413	9.407972	30.95929	-10.963	41.92229	21.63071
2746.191	4.566667	11.07325	0.264385	9.51727	30.81386	-11.0691	41.883	21.4006
2848.788	3.820896	8.87108	0.220209	9.588186	29.448	-10.8369	40.28486	18.12254
2680.419	4.31746	11.44254	0.271422	9.496105	30.71857	-11.4391	42.15771	21.4881
2680.419	4.31746	11.44254	0.271422	9.496105	30.71857	-11.4391	42.15771	21.4881
2680.419	4.31746	11.44254	0.271422	9.496105	30.71857	-11.4391	42.15771	21.4881
2680.419	4.31746	11.44254	0.271422	9.496105	30.71857	-11.4391	42.15771	21.4881
3064.826	4.181818	12.12107	0.268995	9.801269	31.42757	-13.633	45.06057	20.40975
3073.599	4.166667	11.79277	0.261481	9.657075	31.17871	-13.9213	45.1	20.40782
3151.322	4.119403	11.31377	0.254159	9.756036	31.54671	-12.9679	44.51457	20.11212
3151.322	4.119403	11.31377	0.254159	9.756036	31.54671	-12.9679	44.51457	20.11212
2887.812	3.913043	11.84115	0.260928	9.662682	30.74543	-14.6354	45.38086	19.70782
2887.812	3.913043	11.84115	0.260928	9.662682	30.74543	-14.6354	45.38086	19.70782
2887.812	3.913043	11.84115	0.260928	9.662682	30.74543	-14.6354	45.38086	19.70782
2887.812	3.913043	11.84115	0.260928	9.662682	30.74543	-14.6354	45.38086	19.70782
2887.812	3.913043	11.84115	0.260928	9.662682	30.74543	-14.6354	45.38086	19.70782
2887.812	3.913043	11.84115	0.260928	9.662682	30.74543	-14.6354	45.38086	19.70782
2887.812	3.913043	11.84115	0.260928	9.662682	30.74543	-14.6354	45.38086	19.70782
3322.52	5.196429	10.60134	0.26641	9.343971	32.09771	-7.69557	39.79329	21.03012
3322.52	5.196429	10.60134	0.26641	9.343971	32.09771	-7.69557	39.79329	21.03012
3192.077	5.166667	10.88248	0.260203	9.39656	31.336	-10.487	41.823	0.303467
3474.345	4.783333	10.37154	0.256744	9.424103	32.62914	-7.76729	40.39643	21.56245
3474.345	4.783333	10.37154	0.256744	9.424103	32.62914	-7.76729	40.39643	21.56245
3474.345	4.783333	10.37154	0.256744	9.424103	32.62914	-7.76729	40.39643	21.56245
2394.186	4.637931	12.66384	0.288886	9.603697	29.97729	-13.8596	43.83686	17.9426
2394.186	4.637931	12.66384	0.288886	9.603697	29.97729	-13.8596	43.83686	17.9426
3194.596	6.369565	11.50671	0.266601	9.790155	32.58386	-10.577	43.16086	-0.42936
2243.37	4.301587	12.72776	0.291234	9.516183	29.492	-14.2109	43.70286	16.04992
2243.37	4.301587	12.72776	0.291234	9.516183	29.492	-14.2109	43.70286	16.04992
2243.37	4.301587	12.72776	0.291234	9.516183	29.492	-14.2109	43.70286	16.04992
2243.37	4.301587	12.72776	0.291234	9.516183	29.492	-14.2109	43.70286	16.04992
2243.37	4.301587	12.72776	0.291234	9.516183	29.492	-14.2109	43.70286	16.04992
3116.509	5.259259	11.55574	0.280479	9.492325	32.22557	-8.97443	41.2	0.161187
3483.082	5.557692	10.08862	0.23643	9.769134	32.43529	-10.2354	42.67071	21.54803
2677.101	4.677966	12.00808	0.288392	9.354972	30.54514	-11.0929	41.638	21.38553
2677.101	4.677966	12.00808	0.288392	9.354972	30.54514	-11.0929	41.638	21.38553
2766.065	4.151515	11.73557	0.268337	9.729124	30.32571	-13.4087	43.73443	18.35269
2766.065	4.151515	11.73557	0.268337	9.729124	30.32571	-13.4087	43.73443	18.35269
2766.065	4.151515	11.73557	0.268337	9.729124	30.32571	-13.4087	43.73443	18.35269
2766.065	4.151515	11.73557	0.268337	9.729124	30.32571	-13.4087	43.73443	18.35269
2766.065	4.151515	11.73557	0.268337	9.729124	30.32571	-13.4087	43.73443	18.35269

Table S2 - Anonymized database

2726.074	4.775862	11.58785	0.276413	9.407972	30.95929	-10.963	41.92229	21.63071
2726.074	4.775862	11.58785	0.276413	9.407972	30.95929	-10.963	41.92229	21.63071
2726.074	4.775862	11.58785	0.276413	9.407972	30.95929	-10.963	41.92229	21.63071
2726.074	4.775862	11.58785	0.276413	9.407972	30.95929	-10.963	41.92229	21.63071
2726.074	4.775862	11.58785	0.276413	9.407972	30.95929	-10.963	41.92229	21.63071
2726.074	4.775862	11.58785	0.276413	9.407972	30.95929	-10.963	41.92229	21.63071
2726.074	4.775862	11.58785	0.276413	9.407972	30.95929	-10.963	41.92229	21.63071
3126.98	5.163636	11.74875	0.28127	9.529702	32.55129	-9.21914	41.77043	0.209357
3115.696	5.109091	11.82259	0.270007	9.531748	31.62229	-12.164	43.78629	22.99034
3115.696	5.109091	11.82259	0.270007	9.531748	31.62229	-12.164	43.78629	22.99034
3115.696	5.109091	11.82259	0.270007	9.531748	31.62229	-12.164	43.78629	22.99034
2353.025	5.6	12.23752	0.286805	9.577015	29.17486	-13.4936	42.66843	20.21343
2353.025	5.6	12.23752	0.286805	9.577015	29.17486	-13.4936	42.66843	20.21343
2353.025	5.6	12.23752	0.286805	9.577015	29.17486	-13.4936	42.66843	20.21343
2353.025	5.6	12.23752	0.286805	9.577015	29.17486	-13.4936	42.66843	20.21343
3279.042	4.52459	10.46123	0.250542	9.822431	32.29186	-9.46257	41.75443	24.18505
3279.042	4.52459	10.46123	0.250542	9.822431	32.29186	-9.46257	41.75443	24.18505
2990.964	3.852941	11.77972	0.25882	9.655364	30.48171	-15.0314	45.51314	20.16635
3220.654	4.813559	10.51827	0.261617	9.314387	31.37543	-8.82943	40.20486	23.32705
3220.654	4.813559	10.51827	0.261617	9.314387	31.37543	-8.82943	40.20486	23.32705
2664.713	4.724138	11.60777	0.271668	9.607548	30.41771	-12.3101	42.72786	21.37524
2664.713	4.724138	11.60777	0.271668	9.607548	30.41771	-12.3101	42.72786	21.37524
2868.931	3.898551	13.86803	0.29415	9.617925	31.076	-16.0701	47.14614	19.47848
2868.931	3.898551	13.86803	0.29415	9.617925	31.076	-16.0701	47.14614	19.47848
2868.931	3.898551	13.86803	0.29415	9.617925	31.076	-16.0701	47.14614	19.47848
2868.931	3.898551	13.86803	0.29415	9.617925	31.076	-16.0701	47.14614	19.47848
3075.887	4.153846	11.62407	0.257163	9.630664	31.08229	-14.1189	45.20114	20.30646
2264.267	5.490196	11.37267	0.270539	9.682687	28.91757	-13.1196	42.03714	19.97095
2264.267	5.490196	11.37267	0.270539	9.682687	28.91757	-13.1196	42.03714	19.97095
2264.267	5.490196	11.37267	0.270539	9.682687	28.91757	-13.1196	42.03714	19.97095
2264.267	5.490196	11.37267	0.270539	9.682687	28.91757	-13.1196	42.03714	19.97095
2264.267	5.490196	11.37267	0.270539	9.682687	28.91757	-13.1196	42.03714	19.97095
2264.267	5.490196	11.37267	0.270539	9.682687	28.91757	-13.1196	42.03714	19.97095
2264.267	5.490196	11.37267	0.270539	9.682687	28.91757	-13.1196	42.03714	19.97095
2264.267	5.490196	11.37267	0.270539	9.682687	28.91757	-13.1196	42.03714	19.97095
2595.113	3.605634	10.89231	0.249092	9.607472	29.23657	-14.4914	43.728	18.47964
2595.113	3.605634	10.89231	0.249092	9.607472	29.23657	-14.4914	43.728	18.47964
2649.733	3.969231	11.39242	0.264656	9.714297	29.62157	-13.4246	43.04614	17.85072
2649.733	3.969231	11.39242	0.264656	9.714297	29.62157	-13.4246	43.04614	17.85072
3395.995	5.388889	10.53479	0.263448	9.377698	32.267	-7.72114	39.98814	21.23821
2676.135	4.927273	11.26454	0.26263	8.987712	30.43086	-12.4604	42.89129	18.39688
2676.135	4.927273	11.26454	0.26263	8.987712	30.43086	-12.4604	42.89129	18.39688
2676.135	4.927273	11.26454	0.26263	8.987712	30.43086	-12.4604	42.89129	18.39688
2676.135	4.927273	11.26454	0.26263	8.987712	30.43086	-12.4604	42.89129	18.39688
2676.135	4.927273	11.26454	0.26263	8.987712	30.43086	-12.4604	42.89129	18.39688
2676.135	4.927273	11.26454	0.26263	8.987712	30.43086	-12.4604	42.89129	18.39688
2498.522	4.533333	10.88513	0.254832	8.928289	29.48414	-13.2309	42.715	19.13995
2974.456	4.030769	10.95567	0.250968	8.95459	30.55286	-13.1007	43.65357	19.55171
2974.456	4.030769	10.95567	0.250968	8.95459	30.55286	-13.1007	43.65357	19.55171
2974.456	4.030769	10.95567	0.250968	8.95459	30.55286	-13.1007	43.65357	19.55171

Table S2 - Anonymized database

2974.456	4.030769	10.95567	0.250968	8.95459	30.55286	-13.1007	43.65357	19.55171
2974.456	4.030769	10.95567	0.250968	8.95459	30.55286	-13.1007	43.65357	19.55171
2974.456	4.030769	10.95567	0.250968	8.95459	30.55286	-13.1007	43.65357	19.55171
2974.456	4.030769	10.95567	0.250968	8.95459	30.55286	-13.1007	43.65357	19.55171
3024.132	4.030769	10.462	0.243544	8.942156	30.22729	-12.7301	42.95743	19.63534
3024.132	4.030769	10.462	0.243544	8.942156	30.22729	-12.7301	42.95743	19.63534
3024.132	4.030769	10.462	0.243544	8.942156	30.22729	-12.7301	42.95743	19.63534
2756.934	4.566667	10.72162	0.255948	9.032313	30.384	-11.5059	41.88986	20.65026
2756.934	4.566667	10.72162	0.255948	9.032313	30.384	-11.5059	41.88986	20.65026
2799.826	4.542373	11.07159	0.249311	9.105087	30.57157	-13.8371	44.40871	17.4494
2799.826	4.542373	11.07159	0.249311	9.105087	30.57157	-13.8371	44.40871	17.4494
2799.826	4.542373	11.07159	0.249311	9.105087	30.57157	-13.8371	44.40871	17.4494
2942.86	3.567164	10.37463	0.245893	8.69951	29.838	-12.3537	42.19171	15.74591
2942.86	3.567164	10.37463	0.245893	8.69951	29.838	-12.3537	42.19171	15.74591
2967.535	3.779412	10.5232	0.244535	9.00546	29.92871	-13.1049	43.03357	19.39375
2967.535	3.779412	10.5232	0.244535	9.00546	29.92871	-13.1049	43.03357	19.39375
2967.535	3.779412	10.5232	0.244535	9.00546	29.92871	-13.1049	43.03357	19.39375
2718.884	3.921875	8.442255	0.208246	8.998847	28.63929	-11.9006	40.53986	14.11862
2718.884	3.921875	8.442255	0.208246	8.998847	28.63929	-11.9006	40.53986	14.11862
3087.363	4.862069	10.02532	0.248886	9.004414	31.52586	-8.75486	40.28071	21.9568
3087.363	4.862069	10.02532	0.248886	9.004414	31.52586	-8.75486	40.28071	21.9568
3087.363	4.862069	10.02532	0.248886	9.004414	31.52586	-8.75486	40.28071	21.9568
3087.363	4.862069	10.02532	0.248886	9.004414	31.52586	-8.75486	40.28071	21.9568
3087.363	4.862069	10.02532	0.248886	9.004414	31.52586	-8.75486	40.28071	21.9568
3087.363	4.862069	10.02532	0.248886	9.004414	31.52586	-8.75486	40.28071	21.9568
3087.363	4.862069	10.02532	0.248886	9.004414	31.52586	-8.75486	40.28071	21.9568
2518.368	4.459016	11.51837	0.258096	9.220036	29.27157	-15.3567	44.62829	17.88371
2807.883	4.190476	10.83214	0.246376	9.035814	29.79829	-14.1676	43.96586	15.21182
3097.084	4.844828	10.92389	0.266723	8.862637	31.75329	-9.20271	40.956	22.2623
3097.084	4.844828	10.92389	0.266723	8.862637	31.75329	-9.20271	40.956	22.2623
3097.084	4.844828	10.92389	0.266723	8.862637	31.75329	-9.20271	40.956	22.2623
3097.084	4.844828	10.92389	0.266723	8.862637	31.75329	-9.20271	40.956	22.2623
3097.084	4.844828	10.92389	0.266723	8.862637	31.75329	-9.20271	40.956	22.2623
3173.256	4.862069	10.46421	0.257957	8.889374	31.798	-8.76771	40.56571	22.57521
3173.256	4.862069	10.46421	0.257957	8.889374	31.798	-8.76771	40.56571	22.57521
2953.704	3.619718	10.85687	0.250288	8.957851	30.26029	-13.1173	43.37757	19.42741
2953.704	3.619718	10.85687	0.250288	8.957851	30.26029	-13.1173	43.37757	19.42741
2953.704	3.619718	10.85687	0.250288	8.957851	30.26029	-13.1173	43.37757	19.42741
2742.281	4.633333	9.584148	0.227656	9.056959	29.57829	-12.521	42.09929	18.87965
2646.493	4.689655	11.33282	0.264203	8.937867	30.49671	-12.3977	42.89443	18.23403
2999.858	4.16129	10.43253	0.242742	8.842099	29.89586	-13.082	42.97786	19.40637
3085.205	4.7	10.54885	0.261208	8.905989	31.41229	-8.97257	40.38486	21.88501
2687.626	4.542373	10.94315	0.247359	9.105289	29.877	-14.363	44.24	17.0018
2687.626	4.542373	10.94315	0.247359	9.105289	29.877	-14.363	44.24	17.0018
2687.626	4.542373	10.94315	0.247359	9.105289	29.877	-14.363	44.24	17.0018
2687.626	4.542373	10.94315	0.247359	9.105289	29.877	-14.363	44.24	17.0018
2687.626	4.542373	10.94315	0.247359	9.105289	29.877	-14.363	44.24	17.0018
3118.855	4.046875	10.26652	0.241974	8.910968	30.34643	-12.0817	42.42814	21.63227
3077.569	4.5	10.78333	0.264992	8.648788	31.03186	-9.66114	40.693	21.40941
3077.569	4.5	10.78333	0.264992	8.648788	31.03186	-9.66114	40.693	21.40941

Table S2 - Anonymized database

2932.246	4.689655	11.01525	0.262471	8.700931	30.75229	-11.2153	41.96757	21.1399
2829.6	3.555556	11.10361	0.256655	8.846986	30.21014	-13.0526	43.26271	18.90434
3043.179	5.070175	10.62666	0.26309	8.814092	31.35014	-9.04157	40.39171	20.44843
3043.179	5.070175	10.62666	0.26309	8.814092	31.35014	-9.04157	40.39171	20.44843
2933.048	4.349206	10.49677	0.259555	8.92375	30.85714	-9.58429	40.44143	21.28388
2662.339	3.584615	10.71813	0.259567	8.442961	27.81257	-13.4797	41.29229	17.99148
2662.339	3.584615	10.71813	0.259567	8.442961	27.81257	-13.4797	41.29229	17.99148
2662.339	3.584615	10.71813	0.259567	8.442961	27.81257	-13.4797	41.29229	17.99148
3040.462	4.864407	10.83357	0.264768	8.930403	31.33086	-9.58643	40.91729	21.62754
3040.462	4.864407	10.83357	0.264768	8.930403	31.33086	-9.58643	40.91729	21.62754
2662.542	4.344262	11.35504	0.253968	9.015101	30.02743	-14.683	44.71043	16.94557
2662.542	4.344262	11.35504	0.253968	9.015101	30.02743	-14.683	44.71043	16.94557
3113.948	5.017544	9.935632	0.247075	8.996593	31.376	-8.837	40.213	22.00392
2357.397	3.742424	10.59493	0.248061	8.643704	27.30029	-15.4107	42.711	16.92964
2957.386	4.435484	10.50273	0.259459	8.880752	30.82643	-9.65286	40.47929	21.32689
2957.386	4.435484	10.50273	0.259459	8.880752	30.82643	-9.65286	40.47929	21.32689
2957.386	4.435484	10.50273	0.259459	8.880752	30.82643	-9.65286	40.47929	21.32689
2957.386	4.435484	10.50273	0.259459	8.880752	30.82643	-9.65286	40.47929	21.32689
2957.386	4.435484	10.50273	0.259459	8.880752	30.82643	-9.65286	40.47929	21.32689
2957.386	4.435484	10.50273	0.259459	8.880752	30.82643	-9.65286	40.47929	21.32689
2957.386	4.435484	10.50273	0.259459	8.880752	30.82643	-9.65286	40.47929	21.32689
2957.386	4.435484	10.50273	0.259459	8.880752	30.82643	-9.65286	40.47929	21.32689
3038.727	4.728814	9.828841	0.247999	8.850262	30.594	-9.03857	39.63257	21.49053
3205.848	4.79661	9.997283	0.249281	8.916534	31.59814	-8.50629	40.10443	22.22416
3205.848	4.79661	9.997283	0.249281	8.916534	31.59814	-8.50629	40.10443	22.22416
3205.848	4.79661	9.997283	0.249281	8.916534	31.59814	-8.50629	40.10443	22.22416
3205.848	4.79661	9.997283	0.249281	8.916534	31.59814	-8.50629	40.10443	22.22416
2661.64	4.655172	10.23391	0.241802	9.129539	29.50586	-12.8177	42.32357	10.81254
2661.64	4.655172	10.23391	0.241802	9.129539	29.50586	-12.8177	42.32357	10.81254
2661.64	4.655172	10.23391	0.241802	9.129539	29.50586	-12.8177	42.32357	10.81254
2661.64	4.655172	10.23391	0.241802	9.129539	29.50586	-12.8177	42.32357	10.81254
2699.456	4.15873	11.4184	0.255565	9.0218	30.08986	-14.5891	44.679	17.1172
2699.456	4.15873	11.4184	0.255565	9.0218	30.08986	-14.5891	44.679	17.1172
3079.782	4.881356	10.29174	0.256636	8.817044	31.299	-8.80343	40.10243	20.54207
3079.782	4.881356	10.29174	0.256636	8.817044	31.299	-8.80343	40.10243	20.54207
3077.479	4.516129	10.7899	0.264113	8.651458	31.12286	-9.73043	40.85329	21.44169
3077.479	4.516129	10.7899	0.264113	8.651458	31.12286	-9.73043	40.85329	21.44169
3077.479	4.516129	10.7899	0.264113	8.651458	31.12286	-9.73043	40.85329	21.44169
3077.479	4.516129	10.7899	0.264113	8.651458	31.12286	-9.73043	40.85329	21.44169
2418.186	5.074074	10.82791	0.252969	9.258108	28.88971	-13.9136	42.80329	9.588137
2418.186	5.074074	10.82791	0.252969	9.258108	28.88971	-13.9136	42.80329	9.588137
2418.186	5.074074	10.82791	0.252969	9.258108	28.88971	-13.9136	42.80329	9.588137
2983.579	3.666667	10.66051	0.247424	9.000824	30.092	-12.994	43.086	19.47402
2798.628	4.55	10.7441	0.257734	8.935147	30.38557	-11.3011	41.68671	20.63604
2798.628	4.55	10.7441	0.257734	8.935147	30.38557	-11.3011	41.68671	20.63604
2798.628	4.55	10.7441	0.257734	8.935147	30.38557	-11.3011	41.68671	20.63604
2798.628	4.55	10.7441	0.257734	8.935147	30.38557	-11.3011	41.68671	20.63604
2997.876	3.628571	9.728728	0.199229	10.38832	30.502	-18.3299	48.83186	11.54672
3107.093	4.142857	9.710563	0.207432	9.789497	31.84643	-14.9669	46.81329	23.03582
3098.235	3.681159	10.21051	0.210982	9.867865	32.21043	-16.1847	48.39514	22.24348

Table S2 - Anonymized database

3165.654	3.861538	10.13024	0.211984	9.836112	32.23414	-15.5536	47.78771	23.38162
2608.77	3.089744	10.0864	0.207026	10.27747	29.37543	-19.345	48.72043	15.08832
2946.573	4.245902	9.848574	0.205241	9.931942	31.47729	-16.5081	47.98543	21.71282
3147.001	4.360656	9.714091	0.204356	9.888559	31.95743	-15.5777	47.53514	22.418
3549.178	3.513889	8.810376	0.189544	10.04027	33.38714	-13.0947	46.48186	14.22411
2939.377	3.541667	9.598382	0.194586	10.42902	30.412	-18.9151	49.32714	11.47931
2952.344	3.424658	9.764739	0.196614	10.43063	30.56929	-19.0951	49.66443	11.56935
2976.449	3.135135	10.30886	0.204694	10.20132	30.60614	-19.7561	50.36229	21.2319
2976.449	3.135135	10.30886	0.204694	10.20132	30.60614	-19.7561	50.36229	21.2319
2950.969	3.96875	9.887997	0.201203	10.06161	31.76386	-17.3804	49.14429	21.69484
2950.969	3.96875	9.887997	0.201203	10.06161	31.76386	-17.3804	49.14429	21.69484
2989.027	3.361111	9.706876	0.196767	10.39114	30.61429	-18.7176	49.33186	22.89026
2506.682	3.637681	8.851797	0.185123	10.22822	28.75129	-19.0644	47.81571	19.48459
3024.271	4.216667	9.909769	0.203664	10.06437	31.67271	-16.9847	48.65743	22.78604
3024.271	4.216667	9.909769	0.203664	10.06437	31.67271	-16.9847	48.65743	22.78604
3222.86	4.288136	8.841277	0.182143	10.18231	32.28086	-16.2594	48.54029	23.67093
3042.166	3.681818	9.684217	0.200545	9.978877	31.73586	-16.5537	48.28957	21.85101
3030.293	3.450704	9.858533	0.198719	10.33327	30.87457	-18.7359	49.61043	11.79528
3184.869	4.079365	9.347069	0.202388	9.779507	31.50871	-14.6751	46.18386	23.39989
2554.498	3.731343	10.29718	0.213109	10.02711	30.34971	-17.9691	48.31886	19.85525
3129.049	4.2	9.489887	0.194885	10.12341	32.04429	-16.6506	48.69486	23.24579
3158.928	4.125	9.289349	0.195812	9.996184	31.92857	-15.5116	47.44014	22.46529
3339.664	4.174603	9.129423	0.19655	9.850078	32.232	-14.2164	46.44843	13.57277
2961.49	3.833333	9.770547	0.203823	9.890176	31.73357	-16.2029	47.93643	21.72685
3065.358	3.764706	9.782505	0.206515	9.84485	31.603	-15.7666	47.36957	22.95677
3053.863	3.907692	9.975404	0.209832	9.816095	31.66843	-15.8716	47.54	22.87124
3053.863	3.907692	9.975404	0.209832	9.816095	31.66843	-15.8716	47.54	22.87124
2610.051	3.213333	9.584712	0.202486	10.53074	29.096	-18.2391	47.33514	15.18559
3132.831	3.742424	9.895681	0.207888	9.981043	31.94986	-15.6511	47.601	22.23615
3132.831	3.742424	9.895681	0.207888	9.981043	31.94986	-15.6511	47.601	22.23615
2689.903	3.217949	9.952698	0.20402	10.44442	29.31957	-19.4633	48.78286	13.32329
2689.903	3.217949	9.952698	0.20402	10.44442	29.31957	-19.4633	48.78286	13.32329
2922.614	3.236111	10.04527	0.198393	10.28965	30.32329	-20.31	50.63329	21.06594
3355.584	3.823529	8.960777	0.194525	9.82302	32.09971	-13.9653	46.065	23.33846
3161.414	3.818182	9.904945	0.208156	9.826251	32.09357	-15.4906	47.58414	23.34493
2816.375	4.225806	10.11058	0.201421	10.25443	31.65743	-18.5389	50.19629	21.09371
2931.519	3.565217	10.45095	0.204561	10.23607	30.358	-20.7317	51.08971	11.21097
2931.519	3.565217	10.45095	0.204561	10.23607	30.358	-20.7317	51.08971	11.21097
2931.519	3.565217	10.45095	0.204561	10.23607	30.358	-20.7317	51.08971	11.21097
2931.519	3.565217	10.45095	0.204561	10.23607	30.358	-20.7317	51.08971	11.21097
2662.224	3.742424	9.530261	0.19745	10.28178	29.99014	-18.2766	48.26671	20.23103
2662.224	3.742424	9.530261	0.19745	10.28178	29.99014	-18.2766	48.26671	20.23103
3020.883	4.112903	9.512231	0.202034	9.840979	31.40357	-15.6789	47.08243	21.8846
2992.128	3.380282	10.1615	0.200691	10.28589	30.84043	-19.7921	50.63257	11.59315
2334.449	3.444444	9.741253	0.198649	10.44967	28.95714	-20.0804	49.03757	20.41729
2979.006	3.514286	9.7355	0.197383	10.40052	30.39414	-18.9287	49.32286	22.82912
2979.006	3.514286	9.7355	0.197383	10.40052	30.39414	-18.9287	49.32286	22.82912
2484.856	3.637681	10.41304	0.208482	10.37992	29.147	-20.8	49.947	19.61129
2870.028	3.830769	9.322591	0.191365	10.07967	31.28729	-17.429	48.71629	22.80193
2870.028	3.830769	9.322591	0.191365	10.07967	31.28729	-17.429	48.71629	22.80193

Table S2 - Anonymized database

2870.028	3.830769	9.322591	0.191365	10.07967	31.28729	-17.429	48.71629	22.80193
2718.073	3.12	9.238385	0.192095	10.2745	28.56386	-19.529	48.09286	20.09524
2606.346	3.632353	10.4301	0.211153	9.995482	31.43443	-17.9614	49.39586	19.88637
2786.908	3.492958	9.800266	0.210128	10.2229	30.51757	-16.122	46.63957	22.05495
2786.908	3.492958	9.800266	0.210128	10.2229	30.51757	-16.122	46.63957	22.05495
2786.908	3.492958	9.800266	0.210128	10.2229	30.51757	-16.122	46.63957	22.05495
3123.806	4.09375	9.686827	0.205102	9.774654	31.555	-15.6743	47.22929	23.08931
3007.729	3.923077	9.609654	0.200674	9.936124	32.10071	-15.7863	47.887	21.88538
3007.729	3.923077	9.609654	0.200674	9.936124	32.10071	-15.7863	47.887	21.88538
3007.729	3.923077	9.609654	0.200674	9.936124	32.10071	-15.7863	47.887	21.88538
2719.538	3.80597	10.14394	0.204419	10.16886	31.16057	-18.4626	49.62314	20.62244
2719.538	3.80597	10.14394	0.204419	10.16886	31.16057	-18.4626	49.62314	20.62244
2832.254	3.757576	10.66365	0.212504	10.19024	32.00971	-18.1711	50.18086	21.19691
3063.674	3.892308	9.501245	0.202792	9.763144	31.00114	-15.851	46.85214	21.86049
3063.674	3.892308	9.501245	0.202792	9.763144	31.00114	-15.851	46.85214	21.86049
2926.277	3.8	10.21629	0.214888	10.05581	30.92871	-16.6137	47.54243	21.35853
2926.277	3.8	10.21629	0.214888	10.05581	30.92871	-16.6137	47.54243	21.35853
2860.531	3.444444	9.393533	0.200529	10.19238	30.72586	-16.118	46.84386	22.39519
2860.531	3.444444	9.393533	0.200529	10.19238	30.72586	-16.118	46.84386	22.39519
3253.218	4.274194	9.606536	0.202983	9.875097	32.23571	-15.091	47.32671	13.2132
3253.218	4.274194	9.606536	0.202983	9.875097	32.23571	-15.091	47.32671	13.2132
3253.218	4.274194	9.606536	0.202983	9.875097	32.23571	-15.091	47.32671	13.2132
3146.171	4.129032	9.04708	0.188025	10.1896	32.14929	-15.9671	48.11643	23.42611
3146.171	4.129032	9.04708	0.188025	10.1896	32.14929	-15.9671	48.11643	23.42611
2715.243	3.597222	10.52134	0.209668	10.40458	29.67014	-20.5107	50.18086	13.72431
2715.243	3.597222	10.52134	0.209668	10.40458	29.67014	-20.5107	50.18086	13.72431
2955.56	4.147541	9.553184	0.195655	10.03982	31.40329	-17.4234	48.82671	21.63962
2955.56	4.147541	9.553184	0.195655	10.03982	31.40329	-17.4234	48.82671	21.63962
2396.535	3.337838	10.65104	0.212531	10.44231	28.519	-21.5961	50.11514	19.19645
3052.366	4.129032	9.698566	0.200726	10.05568	31.85843	-16.459	48.31743	22.07512
2746.081	3.081081	9.047838	0.191149	10.30719	28.64843	-18.6854	47.33386	10.59751
2746.081	3.081081	9.047838	0.191149	10.30719	28.64843	-18.6854	47.33386	10.59751
3066.458	4.095238	10.02045	0.20307	10.07719	31.792	-17.5527	49.34471	22.81733
3066.458	4.095238	10.02045	0.20307	10.07719	31.792	-17.5527	49.34471	22.81733
2923.503	3.148649	10.07393	0.197836	10.34481	30.52557	-20.395	50.92057	22.45725
2923.503	3.148649	10.07393	0.197836	10.34481	30.52557	-20.395	50.92057	22.45725
2923.503	3.148649	10.07393	0.197836	10.34481	30.52557	-20.395	50.92057	22.45725
2923.503	3.148649	10.07393	0.197836	10.34481	30.52557	-20.395	50.92057	22.45725
2997.424	3.865672	9.59628	0.200211	9.834272	31.40686	-16.524	47.93086	21.7804
3080.584	4.131148	9.272258	0.191442	10.11107	32.01786	-16.416	48.43386	23.07747
3041.622	3.066667	10.70262	0.210772	10.22507	30.71586	-20.0623	50.77814	19.18052
3016.139	3.478873	9.383418	0.190313	10.37424	30.54371	-18.7614	49.30514	21.4313
3016.139	3.478873	9.383418	0.190313	10.37424	30.54371	-18.7614	49.30514	21.4313
3088.305	4.35	9.927816	0.204093	10.03794	32.08886	-16.5547	48.64357	22.1844
3088.305	4.35	9.927816	0.204093	10.03794	32.08886	-16.5547	48.64357	22.1844
2626.119	3.089744	9.693341	0.202767	10.41368	29.196	-18.6094	47.80543	13.31518
2626.119	3.089744	9.693341	0.202767	10.41368	29.196	-18.6094	47.80543	13.31518
2626.119	3.089744	9.693341	0.202767	10.41368	29.196	-18.6094	47.80543	13.31518
3096.618	4.063492	9.568456	0.202182	9.796947	31.35657	-15.9694	47.326	22.03666
3096.618	4.063492	9.568456	0.202182	9.796947	31.35657	-15.9694	47.326	22.03666

Table S2 - Anonymized database

2593.717	3.441176	9.290489	0.195748	10.15841	28.16857	-19.2929	47.46143	19.6807
2593.717	3.441176	9.290489	0.195748	10.15841	28.16857	-19.2929	47.46143	19.6807
3089.078	3.850746	10.15646	0.208547	9.921092	32.163	-16.5381	48.70114	23.13301
3089.078	3.850746	10.15646	0.208547	9.921092	32.163	-16.5381	48.70114	23.13301
3089.078	3.850746	10.15646	0.208547	9.921092	32.163	-16.5381	48.70114	23.13301
3089.078	3.850746	10.15646	0.208547	9.921092	32.163	-16.5381	48.70114	23.13301
3079.435	3.342466	9.668865	0.194996	10.36921	31.41586	-18.1691	49.585	23.25553
3132.089	4.15873	9.632898	0.203246	10.0056	32.07814	-15.3171	47.39529	22.36384
3132.089	4.15873	9.632898	0.203246	10.0056	32.07814	-15.3171	47.39529	22.36384
3052.728	4.09375	10.09171	0.206352	9.916827	32.14229	-16.7631	48.90543	22.94353
3052.728	4.09375	10.09171	0.206352	9.916827	32.14229	-16.7631	48.90543	22.94353
3052.728	4.09375	10.09171	0.206352	9.916827	32.14229	-16.7631	48.90543	22.94353
3052.728	4.09375	10.09171	0.206352	9.916827	32.14229	-16.7631	48.90543	22.94353
3025.46	4.03125	9.863706	0.207216	9.835521	31.501	-16.1	47.601	22.77145
3052.728	4.09375	10.09171	0.206352	9.916827	32.14229	-16.7631	48.90543	22.94353
3052.728	4.09375	10.09171	0.206352	9.916827	32.14229	-16.7631	48.90543	22.94353
2844.379	3.457143	9.777613	0.196765	10.34554	29.874	-19.8179	49.69186	17.44609
2844.379	3.457143	9.777613	0.196765	10.34554	29.874	-19.8179	49.69186	17.44609
2703.508	3.637681	10.27859	0.206883	10.33822	30.76129	-18.9217	49.683	20.56579
2703.508	3.637681	10.27859	0.206883	10.33822	30.76129	-18.9217	49.683	20.56579
2703.508	3.637681	10.27859	0.206883	10.33822	30.76129	-18.9217	49.683	20.56579
2550.962	3.486111	9.757453	0.199574	10.23631	30.00514	-18.8861	48.89129	20.20426
2578.469	3.108108	8.448527	0.179642	10.28123	27.71686	-19.313	47.02986	19.43304
3101.577	4.0625	9.365044	0.20081	9.821972	31.23814	-15.3983	46.63643	22.05755
3101.577	4.0625	9.365044	0.20081	9.821972	31.23814	-15.3983	46.63643	22.05755
3009.203	3.628571	9.71153	0.197405	10.3599	30.71229	-18.4837	49.196	11.6938
3009.203	3.628571	9.71153	0.197405	10.3599	30.71229	-18.4837	49.196	11.6938
3009.203	3.628571	9.71153	0.197405	10.3599	30.71229	-18.4837	49.196	11.6938
2617.909	3.608696	10.25447	0.209609	10.15016	30.47357	-18.4484	48.922	21.03759
2617.909	3.608696	10.25447	0.209609	10.15016	30.47357	-18.4484	48.922	21.03759
2617.909	3.608696	10.25447	0.209609	10.15016	30.47357	-18.4484	48.922	21.03759
2617.909	3.608696	10.25447	0.209609	10.15016	30.47357	-18.4484	48.922	21.03759
3345.531	3.791045	9.12561	0.193741	9.919523	32.98914	-14.113	47.10214	13.41591
3345.531	3.791045	9.12561	0.193741	9.919523	32.98914	-14.113	47.10214	13.41591
3345.531	3.791045	9.12561	0.193741	9.919523	32.98914	-14.113	47.10214	13.41591
3157.675	4.131148	9.31247	0.192054	10.1149	32.12743	-16.3613	48.48871	22.48246
3157.675	4.131148	9.31247	0.192054	10.1149	32.12743	-16.3613	48.48871	22.48246
3157.675	4.131148	9.31247	0.192054	10.1149	32.12743	-16.3613	48.48871	22.48246
3071.319	3.118421	10.67653	0.210396	10.22518	31.22514	-19.5197	50.74486	19.21593
3071.319	3.118421	10.67653	0.210396	10.22518	31.22514	-19.5197	50.74486	19.21593
3071.319	3.118421	10.67653	0.210396	10.22518	31.22514	-19.5197	50.74486	19.21593
2969.13	3.876923	9.648569	0.198923	10.03029	31.83686	-16.6673	48.50414	21.68879
2969.13	3.876923	9.648569	0.198923	10.03029	31.83686	-16.6673	48.50414	21.68879
2987.64	3.585714	9.621104	0.194823	10.3977	30.63357	-18.7501	49.38371	22.88703
2987.64	3.585714	9.621104	0.194823	10.3977	30.63357	-18.7501	49.38371	22.88703
2987.64	3.585714	9.621104	0.194823	10.3977	30.63357	-18.7501	49.38371	22.88703
2987.64	3.585714	9.621104	0.194823	10.3977	30.63357	-18.7501	49.38371	22.88703
2987.64	3.585714	9.621104	0.194823	10.3977	30.63357	-18.7501	49.38371	22.88703
2833.384	3.666667	10.54802	0.218217	10.18309	31.266	-17.0713	48.33729	21.86225
2833.384	3.666667	10.54802	0.218217	10.18309	31.266	-17.0713	48.33729	21.86225

Table S2 - Anonymized database

3036.646	4.245902	9.330536	0.193925	10.15762	31.97314	-16.141	48.11414	22.95326
3036.646	4.245902	9.330536	0.193925	10.15762	31.97314	-16.141	48.11414	22.95326
3036.646	4.245902	9.330536	0.193925	10.15762	31.97314	-16.141	48.11414	22.95326
2971.976	3.507042	9.839538	0.196129	10.30251	30.57086	-19.5979	50.16871	11.47576
2971.976	3.507042	9.839538	0.196129	10.30251	30.57086	-19.5979	50.16871	11.47576
2971.976	3.507042	9.839538	0.196129	10.30251	30.57086	-19.5979	50.16871	11.47576
2355.08	3.507042	11.32059	0.223415	10.31362	29.91371	-20.7569	50.67057	18.80301
2355.08	3.507042	11.32059	0.223415	10.31362	29.91371	-20.7569	50.67057	18.80301
3039.765	4.16129	9.341959	0.20026	9.829737	31.31429	-15.3349	46.64914	21.90146
3039.765	4.16129	9.341959	0.20026	9.829737	31.31429	-15.3349	46.64914	21.90146
3039.765	4.16129	9.341959	0.20026	9.829737	31.31429	-15.3349	46.64914	21.90146
3039.765	4.16129	9.341959	0.20026	9.829737	31.31429	-15.3349	46.64914	21.90146
3039.765	4.16129	9.341959	0.20026	9.829737	31.31429	-15.3349	46.64914	21.90146
3039.765	4.16129	9.341959	0.20026	9.829737	31.31429	-15.3349	46.64914	21.90146
2438.76	3.666667	10.27292	0.207676	10.36404	29.74414	-19.722	49.46614	20.88198
2438.76	3.666667	10.27292	0.207676	10.36404	29.74414	-19.722	49.46614	20.88198
2438.76	3.666667	10.27292	0.207676	10.36404	29.74414	-19.722	49.46614	20.88198
2438.76	3.666667	10.27292	0.207676	10.36404	29.74414	-19.722	49.46614	20.88198
3036.521	4.098361	9.444434	0.194661	10.04042	31.90829	-16.609	48.51729	21.97524
3036.521	4.098361	9.444434	0.194661	10.04042	31.90829	-16.609	48.51729	21.97524
3036.521	4.098361	9.444434	0.194661	10.04042	31.90829	-16.609	48.51729	21.97524
3036.521	4.098361	9.444434	0.194661	10.04042	31.90829	-16.609	48.51729	21.97524
2345.926	3.266667	9.0665	0.186425	10.52026	28.78571	-19.8477	48.63343	20.50251
2345.926	3.266667	9.0665	0.186425	10.52026	28.78571	-19.8477	48.63343	20.50251
2345.926	3.266667	9.0665	0.186425	10.52026	28.78571	-19.8477	48.63343	20.50251
2729.098	3.833333	10.26425	0.20757	10.15239	31.23957	-18.2101	49.44971	20.62871
2729.098	3.833333	10.26425	0.20757	10.15239	31.23957	-18.2101	49.44971	20.62871
2729.098	3.833333	10.26425	0.20757	10.15239	31.23957	-18.2101	49.44971	20.62871
2729.098	3.833333	10.26425	0.20757	10.15239	31.23957	-18.2101	49.44971	20.62871
2729.098	3.833333	10.26425	0.20757	10.15239	31.23957	-18.2101	49.44971	20.62871
2513.247	3.088608	9.5855	0.203461	10.44502	28.597	-18.5153	47.11229	13.03295
2513.247	3.088608	9.5855	0.203461	10.44502	28.597	-18.5153	47.11229	13.03295
2513.247	3.088608	9.5855	0.203461	10.44502	28.597	-18.5153	47.11229	13.03295
2513.247	3.088608	9.5855	0.203461	10.44502	28.597	-18.5153	47.11229	13.03295
3146.277	4.163934	8.829821	0.191257	9.872304	31.41214	-14.7553	46.16743	22.35059
3146.277	4.163934	8.829821	0.191257	9.872304	31.41214	-14.7553	46.16743	22.35059
3146.277	4.163934	8.829821	0.191257	9.872304	31.41214	-14.7553	46.16743	22.35059
3146.277	4.163934	8.829821	0.191257	9.872304	31.41214	-14.7553	46.16743	22.35059
3047.869	3.56338	9.76008	0.198343	10.34188	30.90171	-18.3063	49.208	11.8681
3047.869	3.56338	9.76008	0.198343	10.34188	30.90171	-18.3063	49.208	11.8681
3047.869	3.56338	9.76008	0.198343	10.34188	30.90171	-18.3063	49.208	11.8681
3124.07	3.848485	9.289995	0.197856	9.90706	31.66843	-15.2849	46.95329	22.29269
3124.07	3.848485	9.289995	0.197856	9.90706	31.66843	-15.2849	46.95329	22.29269
3124.07	3.848485	9.289995	0.197856	9.90706	31.66843	-15.2849	46.95329	22.29269
3011.265	4.078125	10.22993	0.208462	9.95724	31.96543	-17.1079	49.07329	22.03953
3011.265	4.078125	10.22993	0.208462	9.95724	31.96543	-17.1079	49.07329	22.03953
3011.265	4.078125	10.22993	0.208462	9.95724	31.96543	-17.1079	49.07329	22.03953
2206.389	3.434211	9.759786	0.202076	10.26453	27.81157	-20.4861	48.29771	18.98945
2206.389	3.434211	9.759786	0.202076	10.26453	27.81157	-20.4861	48.29771	18.98945
2206.389	3.434211	9.759786	0.202076	10.26453	27.81157	-20.4861	48.29771	18.98945

Table S2 - Anonymized database

2206.389	3.434211	9.759786	0.202076	10.26453	27.81157	-20.4861	48.29771	18.98945
2818.002	3.118421	10.37036	0.198005	10.45229	30.28257	-22.0917	52.37429	22.04617
2818.002	3.118421	10.37036	0.198005	10.45229	30.28257	-22.0917	52.37429	22.04617
2818.002	3.118421	10.37036	0.198005	10.45229	30.28257	-22.0917	52.37429	22.04617
2818.002	3.118421	10.37036	0.198005	10.45229	30.28257	-22.0917	52.37429	22.04617
2818.002	3.118421	10.37036	0.198005	10.45229	30.28257	-22.0917	52.37429	22.04617
2818.002	3.118421	10.37036	0.198005	10.45229	30.28257	-22.0917	52.37429	22.04617
2774.309	3.521739	9.703014	0.202362	9.963909	30.53629	-17.4124	47.94871	20.75152
2774.309	3.521739	9.703014	0.202362	9.963909	30.53629	-17.4124	47.94871	20.75152
2774.309	3.521739	9.703014	0.202362	9.963909	30.53629	-17.4124	47.94871	20.75152
2774.309	3.521739	9.703014	0.202362	9.963909	30.53629	-17.4124	47.94871	20.75152
2940.962	3.907692	9.768635	0.205861	9.845118	31.60629	-15.8463	47.45257	21.61192
2940.962	3.907692	9.768635	0.205861	9.845118	31.60629	-15.8463	47.45257	21.61192
2940.962	3.907692	9.768635	0.205861	9.845118	31.60629	-15.8463	47.45257	21.61192
2940.962	3.907692	9.768635	0.205861	9.845118	31.60629	-15.8463	47.45257	21.61192
2940.962	3.907692	9.768635	0.205861	9.845118	31.60629	-15.8463	47.45257	21.61192
2940.962	3.907692	9.768635	0.205861	9.845118	31.60629	-15.8463	47.45257	21.61192
2940.962	3.907692	9.768635	0.205861	9.845118	31.60629	-15.8463	47.45257	21.61192
2940.962	3.907692	9.768635	0.205861	9.845118	31.60629	-15.8463	47.45257	21.61192
2940.962	3.907692	9.768635	0.205861	9.845118	31.60629	-15.8463	47.45257	21.61192
2940.962	3.907692	9.768635	0.205861	9.845118	31.60629	-15.8463	47.45257	21.61192
3043.4	4.666667	8.338459	0.183626	9.777416	30.71686	-14.6931	45.41	21.82645
3043.4	4.666667	8.338459	0.183626	9.777416	30.71686	-14.6931	45.41	21.82645
3043.4	4.666667	8.338459	0.183626	9.777416	30.71686	-14.6931	45.41	21.82645
3043.4	4.666667	8.338459	0.183626	9.777416	30.71686	-14.6931	45.41	21.82645
2720.62	3.924242	9.211519	0.193151	10.20257	30.18943	-17.5013	47.69071	20.48316
2720.62	3.924242	9.211519	0.193151	10.20257	30.18943	-17.5013	47.69071	20.48316
2720.62	3.924242	9.211519	0.193151	10.20257	30.18943	-17.5013	47.69071	20.48316
2720.62	3.924242	9.211519	0.193151	10.20257	30.18943	-17.5013	47.69071	20.48316
2720.62	3.924242	9.211519	0.193151	10.20257	30.18943	-17.5013	47.69071	20.48316
2720.62	3.924242	9.211519	0.193151	10.20257	30.18943	-17.5013	47.69071	20.48316
2720.62	3.924242	9.211519	0.193151	10.20257	30.18943	-17.5013	47.69071	20.48316
2720.62	3.924242	9.211519	0.193151	10.20257	30.18943	-17.5013	47.69071	20.48316
2720.62	3.924242	9.211519	0.193151	10.20257	30.18943	-17.5013	47.69071	20.48316
2887.812	3.913043	11.84115	0.260928	9.662682	30.74543	-14.6354	45.38086	19.70782
2726.074	4.775862	11.58785	0.276413	9.407972	30.95929	-10.963	41.92229	21.63071
2766.065	4.151515	11.73557	0.268337	9.729124	30.32571	-13.4087	43.73443	18.35269
3227.156	5.764706	11.46446	0.262674	9.709424	32.16843	-11.4769	43.64529	-0.05249
3474.345	4.783333	10.37154	0.256744	9.424103	32.62914	-7.76729	40.39643	21.56245
2680.419	4.31746	11.44254	0.271422	9.496105	30.71857	-11.4391	42.15771	21.4881
2766.065	4.151515	11.73557	0.268337	9.729124	30.32571	-13.4087	43.73443	18.35269
3279.042	4.52459	10.46123	0.250542	9.822431	32.29186	-9.46257	41.75443	24.18505
2726.074	4.775862	11.58785	0.276413	9.407972	30.95929	-10.963	41.92229	21.63071
3073.599	4.166667	11.79277	0.261481	9.657075	31.17871	-13.9213	45.1	20.40782
2394.186	4.637931	12.66384	0.288886	9.603697	29.97729	-13.8596	43.83686	17.9426
2726.074	4.775862	11.58785	0.276413	9.407972	30.95929	-10.963	41.92229	21.63071
2726.074	4.775862	11.58785	0.276413	9.407972	30.95929	-10.963	41.92229	21.63071
2243.37	4.301587	12.72776	0.291234	9.516183	29.492	-14.2109	43.70286	16.04992
2677.101	4.677966	12.00808	0.288392	9.354972	30.54514	-11.0929	41.638	21.38553
3028.424	5.035714	11.37895	0.278446	9.508713	31.75771	-9.10814	40.86586	-0.22815

Table S2 - Anonymized database

2726.074	4.775862	11.58785	0.276413	9.407972	30.95929	-10.963	41.92229	21.63071
2635.501	4.419355	11.34874	0.269688	9.466324	30.462	-11.619	42.081	20.89842
2726.074	4.775862	11.58785	0.276413	9.407972	30.95929	-10.963	41.92229	21.63071
2797.137	4.6	12.16245	0.27444	9.758158	30.54471	-13.7727	44.31743	19.6041
3149.185	5.351852	12.01897	0.284084	9.509554	32.63986	-9.668	42.30786	0.352786
2848.788	3.820896	8.87108	0.220209	9.588186	29.448	-10.8369	40.28486	18.12254
2680.419	4.31746	11.44254	0.271422	9.496105	30.71857	-11.4391	42.15771	21.4881
2726.074	4.775862	11.58785	0.276413	9.407972	30.95929	-10.963	41.92229	21.63071
2680.419	4.31746	11.44254	0.271422	9.496105	30.71857	-11.4391	42.15771	21.4881
2595.113	3.605634	10.89231	0.249092	9.607472	29.23657	-14.4914	43.728	18.47964
2595.113	3.605634	10.89231	0.249092	9.607472	29.23657	-14.4914	43.728	18.47964
2680.419	4.31746	11.44254	0.271422	9.496105	30.71857	-11.4391	42.15771	21.4881
2664.713	4.724138	11.60777	0.271668	9.607548	30.41771	-12.3101	42.72786	21.37524
3028.424	5.035714	11.37895	0.278446	9.508713	31.75771	-9.10814	40.86586	-0.22815
2766.065	4.151515	11.73557	0.268337	9.729124	30.32571	-13.4087	43.73443	18.35269
2726.074	4.775862	11.58785	0.276413	9.407972	30.95929	-10.963	41.92229	21.63071
3102.646	5.461538	11.78565	0.282757	9.468715	32.14257	-9.53857	41.68114	0.21083
3079.366	5.142857	11.17254	0.269231	9.602149	31.90286	-9.59514	41.498	-0.31046
2990.964	3.852941	11.77972	0.25882	9.655364	30.48171	-15.0314	45.51314	20.16635
3236.887	5.5	10.4307	0.255932	9.40033	31.69629	-9.05943	40.75571	20.69466
2635.501	4.419355	11.34874	0.269688	9.466324	30.462	-11.619	42.081	20.89842
2664.713	4.724138	11.60777	0.271668	9.607548	30.41771	-12.3101	42.72786	21.37524
3040.069	3.985294	11.61803	0.259429	9.707729	31.03271	-13.7504	44.78314	20.27443
2887.812	3.913043	11.84115	0.260928	9.662682	30.74543	-14.6354	45.38086	19.70782
3116.509	5.259259	11.55574	0.280479	9.492325	32.22557	-8.97443	41.2	0.161187
3149.185	5.351852	12.01897	0.284084	9.509554	32.63986	-9.668	42.30786	0.352786
2726.074	4.775862	11.58785	0.276413	9.407972	30.95929	-10.963	41.92229	21.63071
3236.887	5.5	10.4307	0.255932	9.40033	31.69629	-9.05943	40.75571	20.69466
2868.931	3.898551	13.86803	0.29415	9.617925	31.076	-16.0701	47.14614	19.47848
2848.788	3.820896	8.87108	0.220209	9.588186	29.448	-10.8369	40.28486	18.12254
2726.074	4.775862	11.58785	0.276413	9.407972	30.95929	-10.963	41.92229	21.63071
2677.101	4.677966	12.00808	0.288392	9.354972	30.54514	-11.0929	41.638	21.38553
3064.826	4.181818	12.12107	0.268995	9.801269	31.42757	-13.633	45.06057	20.40975
3274.577	5.259259	10.92509	0.259147	9.779255	32.21114	-9.94671	42.15786	21.05133
3395.995	5.388889	10.53479	0.263448	9.377698	32.267	-7.72114	39.98814	21.23821
2677.101	4.677966	12.00808	0.288392	9.354972	30.54514	-11.0929	41.638	21.38553
3151.322	4.119403	11.31377	0.254159	9.756036	31.54671	-12.9679	44.51457	20.11212
2664.713	4.724138	11.60777	0.271668	9.607548	30.41771	-12.3101	42.72786	21.37524
2766.065	4.151515	11.73557	0.268337	9.729124	30.32571	-13.4087	43.73443	18.35269
2726.074	4.775862	11.58785	0.276413	9.407972	30.95929	-10.963	41.92229	21.63071
3193.935	5.017544	12.02356	0.289988	9.423629	32.07857	-9.38371	41.46229	0.690434
3044.721	6	12.24529	0.277662	9.768518	32.79886	-11.3026	44.10143	-0.90751
2868.931	3.898551	13.86803	0.29415	9.617925	31.076	-16.0701	47.14614	19.47848
3395.995	5.388889	10.53479	0.263448	9.377698	32.267	-7.72114	39.98814	21.23821
2264.267	5.490196	11.37267	0.270539	9.682687	28.91757	-13.1196	42.03714	19.97095
2726.074	4.775862	11.58785	0.276413	9.407972	30.95929	-10.963	41.92229	21.63071
3056.344	5.259259	11.86426	0.28171	9.499051	32.16957	-9.94557	42.11514	0.011604
2353.025	5.6	12.23752	0.286805	9.577015	29.17486	-13.4936	42.66843	20.21343
2243.37	4.301587	12.72776	0.291234	9.516183	29.492	-14.2109	43.70286	16.04992
3483.082	5.557692	10.08862	0.23643	9.769134	32.43529	-10.2354	42.67071	21.54803

Table S2 - Anonymized database

3028.424	5.035714	11.37895	0.278446	9.508713	31.75771	-9.10814	40.86586	-0.22815
3274.577	5.259259	10.92509	0.259147	9.779255	32.21114	-9.94671	42.15786	21.05133
2726.074	4.775862	11.58785	0.276413	9.407972	30.95929	-10.963	41.92229	21.63071
2353.025	5.6	12.23752	0.286805	9.577015	29.17486	-13.4936	42.66843	20.21343
2766.065	4.151515	11.73557	0.268337	9.729124	30.32571	-13.4087	43.73443	18.35269
2649.733	3.969231	11.39242	0.264656	9.714297	29.62157	-13.4246	43.04614	17.85072
2243.37	4.301587	12.72776	0.291234	9.516183	29.492	-14.2109	43.70286	16.04992
2726.074	4.775862	11.58785	0.276413	9.407972	30.95929	-10.963	41.92229	21.63071
2726.074	4.775862	11.58785	0.276413	9.407972	30.95929	-10.963	41.92229	21.63071
3064.826	4.181818	12.12107	0.268995	9.801269	31.42757	-13.633	45.06057	20.40975
2848.788	3.820896	8.87108	0.220209	9.588186	29.448	-10.8369	40.28486	18.12254
2243.37	4.301587	12.72776	0.291234	9.516183	29.492	-14.2109	43.70286	16.04992
3126.98	5.163636	11.74875	0.28127	9.529702	32.55129	-9.21914	41.77043	0.209357
2868.931	3.898551	13.86803	0.29415	9.617925	31.076	-16.0701	47.14614	19.47848
2848.788	3.820896	8.87108	0.220209	9.588186	29.448	-10.8369	40.28486	18.12254
3064.826	4.181818	12.12107	0.268995	9.801269	31.42757	-13.633	45.06057	20.40975
3126.98	5.163636	11.74875	0.28127	9.529702	32.55129	-9.21914	41.77043	0.209357
3064.826	4.181818	12.12107	0.268995	9.801269	31.42757	-13.633	45.06057	20.40975
2726.074	4.775862	11.58785	0.276413	9.407972	30.95929	-10.963	41.92229	21.63071
2264.267	5.490196	11.37267	0.270539	9.682687	28.91757	-13.1196	42.03714	19.97095
2726.074	4.775862	11.58785	0.276413	9.407972	30.95929	-10.963	41.92229	21.63071
2243.37	4.301587	12.72776	0.291234	9.516183	29.492	-14.2109	43.70286	16.04992
3483.082	5.557692	10.08862	0.23643	9.769134	32.43529	-10.2354	42.67071	21.54803
2868.931	3.898551	13.86803	0.29415	9.617925	31.076	-16.0701	47.14614	19.47848
2726.074	4.775862	11.58785	0.276413	9.407972	30.95929	-10.963	41.92229	21.63071
3193.935	5.017544	12.02356	0.289988	9.423629	32.07857	-9.38371	41.46229	0.690434
3194.596	6.369565	11.50671	0.266601	9.790155	32.58386	-10.577	43.16086	-0.42936
3395.995	5.388889	10.53479	0.263448	9.377698	32.267	-7.72114	39.98814	21.23821
2243.37	4.301587	12.72776	0.291234	9.516183	29.492	-14.2109	43.70286	16.04992
2243.37	4.301587	12.72776	0.291234	9.516183	29.492	-14.2109	43.70286	16.04992
3483.082	5.557692	10.08862	0.23643	9.769134	32.43529	-10.2354	42.67071	21.54803
2677.101	4.677966	12.00808	0.288392	9.354972	30.54514	-11.0929	41.638	21.38553
3322.52	5.196429	10.60134	0.26641	9.343971	32.09771	-7.69557	39.79329	21.03012
3056.344	5.259259	11.86426	0.28171	9.499051	32.16957	-9.94557	42.11514	0.011604
2243.37	4.301587	12.72776	0.291234	9.516183	29.492	-14.2109	43.70286	16.04992
2680.419	4.31746	11.44254	0.271422	9.496105	30.71857	-11.4391	42.15771	21.4881
3179.685	4.847458	11.90968	0.284568	9.472485	32.63014	-9.22171	41.85186	0.573214
2264.267	5.490196	11.37267	0.270539	9.682687	28.91757	-13.1196	42.03714	19.97095
3175.236	3.970149	10.86033	0.246713	9.629837	31.165	-12.8551	44.02014	20.82295
2677.101	4.677966	12.00808	0.288392	9.354972	30.54514	-11.0929	41.638	21.38553
2243.37	4.301587	12.72776	0.291234	9.516183	29.492	-14.2109	43.70286	16.04992
3115.696	5.109091	11.82259	0.270007	9.531748	31.62229	-12.164	43.78629	22.99034
3064.826	4.181818	12.12107	0.268995	9.801269	31.42757	-13.633	45.06057	20.40975
2677.101	4.677966	12.00808	0.288392	9.354972	30.54514	-11.0929	41.638	21.38553
2887.812	3.913043	11.84115	0.260928	9.662682	30.74543	-14.6354	45.38086	19.70782
3115.696	5.109091	11.82259	0.270007	9.531748	31.62229	-12.164	43.78629	22.99034
3131.78	5.705882	11.75005	0.281206	9.412608	32.16186	-9.62257	41.78443	0.459989
2766.065	4.151515	11.73557	0.268337	9.729124	30.32571	-13.4087	43.73443	18.35269
2848.788	3.820896	8.87108	0.220209	9.588186	29.448	-10.8369	40.28486	18.12254
2680.419	4.31746	11.44254	0.271422	9.496105	30.71857	-11.4391	42.15771	21.4881

Table S2 - Anonymized database

3131.78	5.705882	11.75005	0.281206	9.412608	32.16186	-9.62257	41.78443	0.459989
3483.082	5.557692	10.08862	0.23643	9.769134	32.43529	-10.2354	42.67071	21.54803
3395.995	5.388889	10.53479	0.263448	9.377698	32.267	-7.72114	39.98814	21.23821
2264.267	5.490196	11.37267	0.270539	9.682687	28.91757	-13.1196	42.03714	19.97095
2726.074	4.775862	11.58785	0.276413	9.407972	30.95929	-10.963	41.92229	21.63071
2726.074	4.775862	11.58785	0.276413	9.407972	30.95929	-10.963	41.92229	21.63071
2810.948	5.415094	10.69609	0.250334	9.695685	30.62543	-12.1019	42.72729	19.40664
2677.101	4.677966	12.00808	0.288392	9.354972	30.54514	-11.0929	41.638	21.38553
2353.025	5.6	12.23752	0.286805	9.577015	29.17486	-13.4936	42.66843	20.21343
2635.501	4.419355	11.34874	0.269688	9.466324	30.462	-11.619	42.081	20.89842
3315.923	4.716667	11.4316	0.27779	9.46296	32.57943	-8.57257	41.152	21.11505
2726.074	4.775862	11.58785	0.276413	9.407972	30.95929	-10.963	41.92229	21.63071
2635.501	4.419355	11.34874	0.269688	9.466324	30.462	-11.619	42.081	20.89842
2887.812	3.913043	11.84115	0.260928	9.662682	30.74543	-14.6354	45.38086	19.70782
2635.501	4.419355	11.34874	0.269688	9.466324	30.462	-11.619	42.081	20.89842
2766.065	4.151515	11.73557	0.268337	9.729124	30.32571	-13.4087	43.73443	18.35269
3194.596	6.369565	11.50671	0.266601	9.790155	32.58386	-10.577	43.16086	-0.42936
3115.696	5.109091	11.82259	0.270007	9.531748	31.62229	-12.164	43.78629	22.99034
2887.812	3.913043	11.84115	0.260928	9.662682	30.74543	-14.6354	45.38086	19.70782
2595.113	3.605634	10.89231	0.249092	9.607472	29.23657	-14.4914	43.728	18.47964
3149.185	5.351852	12.01897	0.284084	9.509554	32.63986	-9.668	42.30786	0.352786
2726.074	4.775862	11.58785	0.276413	9.407972	30.95929	-10.963	41.92229	21.63071
2264.267	5.490196	11.37267	0.270539	9.682687	28.91757	-13.1196	42.03714	19.97095
3115.696	5.109091	11.82259	0.270007	9.531748	31.62229	-12.164	43.78629	22.99034
3193.935	5.017544	12.02356	0.289988	9.423629	32.07857	-9.38371	41.46229	0.690434
2887.812	3.913043	11.84115	0.260928	9.662682	30.74543	-14.6354	45.38086	19.70782
2353.025	5.6	12.23752	0.286805	9.577015	29.17486	-13.4936	42.66843	20.21343
3300.713	4.965517	11.43246	0.277964	9.426942	32.93186	-8.19743	41.12929	21.16282
2243.37	4.301587	12.72776	0.291234	9.516183	29.492	-14.2109	43.70286	16.04992
2664.713	4.724138	11.60777	0.271668	9.607548	30.41771	-12.3101	42.72786	21.37524
2726.074	4.775862	11.58785	0.276413	9.407972	30.95929	-10.963	41.92229	21.63071
3193.935	5.017544	12.02356	0.289988	9.423629	32.07857	-9.38371	41.46229	0.690434
2726.074	4.775862	11.58785	0.276413	9.407972	30.95929	-10.963	41.92229	21.63071
2868.931	3.898551	13.86803	0.29415	9.617925	31.076	-16.0701	47.14614	19.47848
3483.082	5.557692	10.08862	0.23643	9.769134	32.43529	-10.2354	42.67071	21.54803
2677.101	4.677966	12.00808	0.288392	9.354972	30.54514	-11.0929	41.638	21.38553
2726.074	4.775862	11.58785	0.276413	9.407972	30.95929	-10.963	41.92229	21.63071
3138.446	4.733333	11.16117	0.26968	9.646659	32.40114	-8.98557	41.38671	23.22381
2595.113	3.605634	10.89231	0.249092	9.607472	29.23657	-14.4914	43.728	18.47964
2394.186	4.637931	12.66384	0.288886	9.603697	29.97729	-13.8596	43.83686	17.9426
3149.185	5.351852	12.01897	0.284084	9.509554	32.63986	-9.668	42.30786	0.352786
2353.025	5.6	12.23752	0.286805	9.577015	29.17486	-13.4936	42.66843	20.21343
3192.077	5.166667	10.88248	0.260203	9.39656	31.336	-10.487	41.823	0.303467
2726.074	4.775862	11.58785	0.276413	9.407972	30.95929	-10.963	41.92229	21.63071
2887.812	3.913043	11.84115	0.260928	9.662682	30.74543	-14.6354	45.38086	19.70782
2810.948	5.415094	10.69609	0.250334	9.695685	30.62543	-12.1019	42.72729	19.40664
3064.826	4.181818	12.12107	0.268995	9.801269	31.42757	-13.633	45.06057	20.40975
2394.186	4.637931	12.66384	0.288886	9.603697	29.97729	-13.8596	43.83686	17.9426
2243.37	4.301587	12.72776	0.291234	9.516183	29.492	-14.2109	43.70286	16.04992
2848.788	3.820896	8.87108	0.220209	9.588186	29.448	-10.8369	40.28486	18.12254

Table S2 - Anonymized database

2810.948	5.415094	10.69609	0.250334	9.695685	30.62543	-12.1019	42.72729	19.40664
3131.78	5.705882	11.75005	0.281206	9.412608	32.16186	-9.62257	41.78443	0.459989
2887.812	3.913043	11.84115	0.260928	9.662682	30.74543	-14.6354	45.38086	19.70782
2726.074	4.775862	11.58785	0.276413	9.407972	30.95929	-10.963	41.92229	21.63071
2664.713	4.724138	11.60777	0.271668	9.607548	30.41771	-12.3101	42.72786	21.37524
3149.185	5.351852	12.01897	0.284084	9.509554	32.63986	-9.668	42.30786	0.352786
2653.503	3.705882	10.52719	0.252445	9.629148	28.88357	-12.8173	41.70086	20.99415
2726.074	4.775862	11.58785	0.276413	9.407972	30.95929	-10.963	41.92229	21.63071
3193.935	5.017544	12.02356	0.289988	9.423629	32.07857	-9.38371	41.46229	0.690434
2726.074	4.775862	11.58785	0.276413	9.407972	30.95929	-10.963	41.92229	21.63071
3483.082	5.557692	10.08862	0.23643	9.769134	32.43529	-10.2354	42.67071	21.54803
2595.113	3.605634	10.89231	0.249092	9.607472	29.23657	-14.4914	43.728	18.47964
3474.345	4.783333	10.37154	0.256744	9.424103	32.62914	-7.76729	40.39643	21.56245
3322.52	5.196429	10.60134	0.26641	9.343971	32.09771	-7.69557	39.79329	21.03012
2664.713	4.724138	11.60777	0.271668	9.607548	30.41771	-12.3101	42.72786	21.37524
2848.788	3.820896	8.87108	0.220209	9.588186	29.448	-10.8369	40.28486	18.12254
2810.948	5.415094	10.69609	0.250334	9.695685	30.62543	-12.1019	42.72729	19.40664
2726.074	4.775862	11.58785	0.276413	9.407972	30.95929	-10.963	41.92229	21.63071
3126.98	5.163636	11.74875	0.28127	9.529702	32.55129	-9.21914	41.77043	0.209357
2353.025	5.6	12.23752	0.286805	9.577015	29.17486	-13.4936	42.66843	20.21343
3126.98	5.163636	11.74875	0.28127	9.529702	32.55129	-9.21914	41.77043	0.209357
3151.322	4.119403	11.31377	0.254159	9.756036	31.54671	-12.9679	44.51457	20.11212
2677.101	4.677966	12.00808	0.288392	9.354972	30.54514	-11.0929	41.638	21.38553
3300.713	4.965517	11.43246	0.277964	9.426942	32.93186	-8.19743	41.12929	21.16282
3064.826	4.181818	12.12107	0.268995	9.801269	31.42757	-13.633	45.06057	20.40975
3040.069	3.985294	11.61803	0.259429	9.707729	31.03271	-13.7504	44.78314	20.27443
2664.713	4.724138	11.60777	0.271668	9.607548	30.41771	-12.3101	42.72786	21.37524
2868.931	3.898551	13.86803	0.29415	9.617925	31.076	-16.0701	47.14614	19.47848
3079.366	5.142857	11.17254	0.269231	9.602149	31.90286	-9.59514	41.498	-0.31046
2726.074	4.775862	11.58785	0.276413	9.407972	30.95929	-10.963	41.92229	21.63071
2353.025	5.6	12.23752	0.286805	9.577015	29.17486	-13.4936	42.66843	20.21343
3149.185	5.351852	12.01897	0.284084	9.509554	32.63986	-9.668	42.30786	0.352786
3227.156	5.764706	11.46446	0.262674	9.709424	32.16843	-11.4769	43.64529	-0.05249
3192.077	5.166667	10.88248	0.260203	9.39656	31.336	-10.487	41.823	0.303467
2677.101	4.677966	12.00808	0.288392	9.354972	30.54514	-11.0929	41.638	21.38553
2595.113	3.605634	10.89231	0.249092	9.607472	29.23657	-14.4914	43.728	18.47964
2264.267	5.490196	11.37267	0.270539	9.682687	28.91757	-13.1196	42.03714	19.97095
2746.191	4.566667	11.07325	0.264385	9.51727	30.81386	-11.0691	41.883	21.4006
2264.267	5.490196	11.37267	0.270539	9.682687	28.91757	-13.1196	42.03714	19.97095
3155.268	5.538462	11.65081	0.278366	9.459596	32.31814	-9.53614	41.85429	0.438879
2676.135	4.927273	11.26454	0.26263	8.987712	30.43086	-12.4604	42.89129	18.39688
2429.035	4.793103	11.21175	0.26066	9.080668	29.153	-13.8599	43.01286	17.34101
2662.339	3.584615	10.71813	0.259567	8.442961	27.81257	-13.4797	41.29229	17.99148
3024.132	4.030769	10.462	0.243544	8.942156	30.22729	-12.7301	42.95743	19.63534
2942.86	3.567164	10.37463	0.245893	8.69951	29.838	-12.3537	42.19171	15.74591
2983.579	3.666667	10.66051	0.247424	9.000824	30.092	-12.994	43.086	19.47402
2974.456	4.030769	10.95567	0.250968	8.95459	30.55286	-13.1007	43.65357	19.55171
2676.135	4.927273	11.26454	0.26263	8.987712	30.43086	-12.4604	42.89129	18.39688
3205.848	4.79661	9.997283	0.249281	8.916534	31.59814	-8.50629	40.10443	22.22416
2676.135	4.927273	11.26454	0.26263	8.987712	30.43086	-12.4604	42.89129	18.39688

Table S2 - Anonymized database

2718.884	3.921875	8.442255	0.208246	8.998847	28.63929	-11.9006	40.53986	14.11862
2799.826	4.542373	11.07159	0.249311	9.105087	30.57157	-13.8371	44.40871	17.4494
2662.339	3.584615	10.71813	0.259567	8.442961	27.81257	-13.4797	41.29229	17.99148
2974.456	4.030769	10.95567	0.250968	8.95459	30.55286	-13.1007	43.65357	19.55171
3097.084	4.844828	10.92389	0.266723	8.862637	31.75329	-9.20271	40.956	22.2623
2974.456	4.030769	10.95567	0.250968	8.95459	30.55286	-13.1007	43.65357	19.55171
2799.826	4.542373	11.07159	0.249311	9.105087	30.57157	-13.8371	44.40871	17.4494
2687.626	4.542373	10.94315	0.247359	9.105289	29.877	-14.363	44.24	17.0018
3087.363	4.862069	10.02532	0.248886	9.004414	31.52586	-8.75486	40.28071	21.9568
2676.135	4.927273	11.26454	0.26263	8.987712	30.43086	-12.4604	42.89129	18.39688
2518.368	4.459016	11.51837	0.258096	9.220036	29.27157	-15.3567	44.62829	17.88371
3087.363	4.862069	10.02532	0.248886	9.004414	31.52586	-8.75486	40.28071	21.9568
2974.456	4.030769	10.95567	0.250968	8.95459	30.55286	-13.1007	43.65357	19.55171
2429.035	4.793103	11.21175	0.26066	9.080668	29.153	-13.8599	43.01286	17.34101
3113.026	4.793103	10.04865	0.249842	8.974093	31.278	-8.942	40.22	21.90496
2799.826	4.542373	11.07159	0.249311	9.105087	30.57157	-13.8371	44.40871	17.4494
3097.084	4.844828	10.92389	0.266723	8.862637	31.75329	-9.20271	40.956	22.2623
3087.363	4.862069	10.02532	0.248886	9.004414	31.52586	-8.75486	40.28071	21.9568
3087.363	4.862069	10.02532	0.248886	9.004414	31.52586	-8.75486	40.28071	21.9568
2957.386	4.435484	10.50273	0.259459	8.880752	30.82643	-9.65286	40.47929	21.32689
2646.493	4.689655	11.33282	0.264203	8.937867	30.49671	-12.3977	42.89443	18.23403
2240.972	4.5	10.24482	0.238973	9.470442	27.75557	-15.1146	42.87014	16.65014
2699.456	4.15873	11.4184	0.255565	9.0218	30.08986	-14.5891	44.679	17.1172
3140.023	3.939394	10.33348	0.241358	8.912543	30.57343	-12.2404	42.81386	21.6844
2799.826	4.542373	11.07159	0.249311	9.105087	30.57157	-13.8371	44.40871	17.4494
2869.708	4.387097	10.01899	0.246341	8.666644	29.66714	-11.0041	40.67129	19.24168
2676.135	4.927273	11.26454	0.26263	8.987712	30.43086	-12.4604	42.89129	18.39688
2661.64	4.655172	10.23391	0.241802	9.129539	29.50586	-12.8177	42.32357	10.81254
3186.837	4.896552	9.49847	0.242855	8.842822	31.02329	-8.08843	39.11171	22.09674
2957.386	4.435484	10.50273	0.259459	8.880752	30.82643	-9.65286	40.47929	21.32689
2240.972	4.5	10.24482	0.238973	9.470442	27.75557	-15.1146	42.87014	16.65014
2662.339	3.584615	10.71813	0.259567	8.442961	27.81257	-13.4797	41.29229	17.99148
3097.084	4.844828	10.92389	0.266723	8.862637	31.75329	-9.20271	40.956	22.2623
2799.826	4.542373	11.07159	0.249311	9.105087	30.57157	-13.8371	44.40871	17.4494
3097.084	4.844828	10.92389	0.266723	8.862637	31.75329	-9.20271	40.956	22.2623
3040.462	4.864407	10.83357	0.264768	8.930403	31.33086	-9.58643	40.91729	21.62754
2798.628	4.55	10.7441	0.257734	8.935147	30.38557	-11.3011	41.68671	20.63604
2661.64	4.655172	10.23391	0.241802	9.129539	29.50586	-12.8177	42.32357	10.81254
2807.883	4.190476	10.83214	0.246376	9.035814	29.79829	-14.1676	43.96586	15.21182
3097.084	4.844828	10.92389	0.266723	8.862637	31.75329	-9.20271	40.956	22.2623
2974.456	4.030769	10.95567	0.250968	8.95459	30.55286	-13.1007	43.65357	19.55171
3161.85	4.779661	10.63573	0.262117	8.883855	31.75043	-8.82586	40.57629	22.06796
3040.462	4.864407	10.83357	0.264768	8.930403	31.33086	-9.58643	40.91729	21.62754
3097.084	4.844828	10.92389	0.266723	8.862637	31.75329	-9.20271	40.956	22.2623
3087.363	4.862069	10.02532	0.248886	9.004414	31.52586	-8.75486	40.28071	21.9568
2687.626	4.542373	10.94315	0.247359	9.105289	29.877	-14.363	44.24	17.0018
3087.363	4.862069	10.02532	0.248886	9.004414	31.52586	-8.75486	40.28071	21.9568
3118.855	4.046875	10.26652	0.241974	8.910968	30.34643	-12.0817	42.42814	21.63227
2798.628	4.55	10.7441	0.257734	8.935147	30.38557	-11.3011	41.68671	20.63604
2662.339	3.584615	10.71813	0.259567	8.442961	27.81257	-13.4797	41.29229	17.99148

Table S2 - Anonymized database

3205.848	4.79661	9.997283	0.249281	8.916534	31.59814	-8.50629	40.10443	22.22416
3161.85	4.779661	10.63573	0.262117	8.883855	31.75043	-8.82586	40.57629	22.06796
2999.858	4.16129	10.43253	0.242742	8.842099	29.89586	-13.082	42.97786	19.40637
3097.084	4.844828	10.92389	0.266723	8.862637	31.75329	-9.20271	40.956	22.2623
2240.972	4.5	10.24482	0.238973	9.470442	27.75557	-15.1146	42.87014	16.65014
3077.479	4.516129	10.7899	0.264113	8.651458	31.12286	-9.73043	40.85329	21.44169
2974.456	4.030769	10.95567	0.250968	8.95459	30.55286	-13.1007	43.65357	19.55171
2829.6	3.555556	11.10361	0.256655	8.846986	30.21014	-13.0526	43.26271	18.90434
2999.858	4.16129	10.43253	0.242742	8.842099	29.89586	-13.082	42.97786	19.40637
3087.363	4.862069	10.02532	0.248886	9.004414	31.52586	-8.75486	40.28071	21.9568
2799.826	4.542373	11.07159	0.249311	9.105087	30.57157	-13.8371	44.40871	17.4494
3118.855	4.046875	10.26652	0.241974	8.910968	30.34643	-12.0817	42.42814	21.63227
2718.884	3.921875	8.442255	0.208246	8.998847	28.63929	-11.9006	40.53986	14.11862
2662.542	4.344262	11.35504	0.253968	9.015101	30.02743	-14.683	44.71043	16.94557
3176.007	4.982456	10.08337	0.250595	8.931754	31.66286	-8.57486	40.23771	22.02507
2240.972	4.5	10.24482	0.238973	9.470442	27.75557	-15.1146	42.87014	16.65014
2932.246	4.689655	11.01525	0.262471	8.700931	30.75229	-11.2153	41.96757	21.1399
2240.972	4.5	10.24482	0.238973	9.470442	27.75557	-15.1146	42.87014	16.65014
2989.158	4.442623	10.31513	0.252065	8.9269	30.95129	-9.97129	40.92257	21.3243
3024.132	4.030769	10.462	0.243544	8.942156	30.22729	-12.7301	42.95743	19.63534
2957.386	4.435484	10.50273	0.259459	8.880752	30.82643	-9.65286	40.47929	21.32689
2953.894	3.642857	11.23202	0.258294	8.934624	30.589	-12.8964	43.48543	19.47527
2967.535	3.779412	10.5232	0.244535	9.00546	29.92871	-13.1049	43.03357	19.39375
2498.522	4.533333	10.88513	0.254832	8.928289	29.48414	-13.2309	42.715	19.13995
3087.363	4.862069	10.02532	0.248886	9.004414	31.52586	-8.75486	40.28071	21.9568
2989.158	4.442623	10.31513	0.252065	8.9269	30.95129	-9.97129	40.92257	21.3243
2957.386	4.435484	10.50273	0.259459	8.880752	30.82643	-9.65286	40.47929	21.32689
2662.339	3.584615	10.71813	0.259567	8.442961	27.81257	-13.4797	41.29229	17.99148
3097.084	4.844828	10.92389	0.266723	8.862637	31.75329	-9.20271	40.956	22.2623
2974.456	4.030769	10.95567	0.250968	8.95459	30.55286	-13.1007	43.65357	19.55171
3077.569	4.5	10.78333	0.264992	8.648788	31.03186	-9.66114	40.693	21.40941
2798.628	4.55	10.7441	0.257734	8.935147	30.38557	-11.3011	41.68671	20.63604
3077.479	4.516129	10.7899	0.264113	8.651458	31.12286	-9.73043	40.85329	21.44169
2418.186	5.074074	10.82791	0.252969	9.258108	28.88971	-13.9136	42.80329	9.588137
3040.462	4.864407	10.83357	0.264768	8.930403	31.33086	-9.58643	40.91729	21.62754
3077.479	4.516129	10.7899	0.264113	8.651458	31.12286	-9.73043	40.85329	21.44169
2967.535	3.779412	10.5232	0.244535	9.00546	29.92871	-13.1049	43.03357	19.39375
3077.479	4.516129	10.7899	0.264113	8.651458	31.12286	-9.73043	40.85329	21.44169
3087.363	4.862069	10.02532	0.248886	9.004414	31.52586	-8.75486	40.28071	21.9568
2676.135	4.927273	11.26454	0.26263	8.987712	30.43086	-12.4604	42.89129	18.39688
2676.135	4.927273	11.26454	0.26263	8.987712	30.43086	-12.4604	42.89129	18.39688
2676.135	4.927273	11.26454	0.26263	8.987712	30.43086	-12.4604	42.89129	18.39688
2240.972	4.5	10.24482	0.238973	9.470442	27.75557	-15.1146	42.87014	16.65014
2662.339	3.584615	10.71813	0.259567	8.442961	27.81257	-13.4797	41.29229	17.99148
2959.483	4.745763	10.91957	0.268321	8.862077	31.116	-9.58	40.696	21.3915
3087.363	4.862069	10.02532	0.248886	9.004414	31.52586	-8.75486	40.28071	21.9568
3205.848	4.79661	9.997283	0.249281	8.916534	31.59814	-8.50629	40.10443	22.22416
3140.023	3.939394	10.33348	0.241358	8.912543	30.57343	-12.2404	42.81386	21.6844
2974.456	4.030769	10.95567	0.250968	8.95459	30.55286	-13.1007	43.65357	19.55171
2661.64	4.655172	10.23391	0.241802	9.129539	29.50586	-12.8177	42.32357	10.81254

Table S2 - Anonymized database

3087.363	4.862069	10.02532	0.248886	9.004414	31.52586	-8.75486	40.28071	21.9568
2662.339	3.584615	10.71813	0.259567	8.442961	27.81257	-13.4797	41.29229	17.99148
3087.363	4.862069	10.02532	0.248886	9.004414	31.52586	-8.75486	40.28071	21.9568
2999.858	4.16129	10.43253	0.242742	8.842099	29.89586	-13.082	42.97786	19.40637
3077.479	4.516129	10.7899	0.264113	8.651458	31.12286	-9.73043	40.85329	21.44169
2967.535	3.779412	10.5232	0.244535	9.00546	29.92871	-13.1049	43.03357	19.39375
3111.843	4.847458	11.07463	0.276246	8.774996	31.43486	-8.65486	40.08971	22.16168
2718.884	3.921875	8.442255	0.208246	8.998847	28.63929	-11.9006	40.53986	14.11862
2718.884	3.921875	8.442255	0.208246	8.998847	28.63929	-11.9006	40.53986	14.11862
2967.535	3.779412	10.5232	0.244535	9.00546	29.92871	-13.1049	43.03357	19.39375
2676.135	4.927273	11.26454	0.26263	8.987712	30.43086	-12.4604	42.89129	18.39688
3146.548	3.910448	10.54269	0.246233	8.918875	30.84171	-11.9743	42.816	20.15043
2798.628	4.55	10.7441	0.257734	8.935147	30.38557	-11.3011	41.68671	20.63604
2661.64	4.655172	10.23391	0.241802	9.129539	29.50586	-12.8177	42.32357	10.81254
2357.397	3.742424	10.59493	0.248061	8.643704	27.30029	-15.4107	42.711	16.92964
3087.363	4.862069	10.02532	0.248886	9.004414	31.52586	-8.75486	40.28071	21.9568
2974.456	4.030769	10.95567	0.250968	8.95459	30.55286	-13.1007	43.65357	19.55171
2933.048	4.349206	10.49677	0.259555	8.92375	30.85714	-9.58429	40.44143	21.28388
2799.826	4.542373	11.07159	0.249311	9.105087	30.57157	-13.8371	44.40871	17.4494
2957.386	4.435484	10.50273	0.259459	8.880752	30.82643	-9.65286	40.47929	21.32689
2676.135	4.927273	11.26454	0.26263	8.987712	30.43086	-12.4604	42.89129	18.39688
3097.084	4.844828	10.92389	0.266723	8.862637	31.75329	-9.20271	40.956	22.2623
2953.894	3.642857	11.23202	0.258294	8.934624	30.589	-12.8964	43.48543	19.47527
3097.084	4.844828	10.92389	0.266723	8.862637	31.75329	-9.20271	40.956	22.2623
3118.855	4.046875	10.26652	0.241974	8.910968	30.34643	-12.0817	42.42814	21.63227
2687.626	4.542373	10.94315	0.247359	9.105289	29.877	-14.363	44.24	17.0018
3087.363	4.862069	10.02532	0.248886	9.004414	31.52586	-8.75486	40.28071	21.9568
3040.462	4.864407	10.83357	0.264768	8.930403	31.33086	-9.58643	40.91729	21.62754
3097.084	4.844828	10.92389	0.266723	8.862637	31.75329	-9.20271	40.956	22.2623
2967.535	3.779412	10.5232	0.244535	9.00546	29.92871	-13.1049	43.03357	19.39375
2676.135	4.927273	11.26454	0.26263	8.987712	30.43086	-12.4604	42.89129	18.39688
2942.86	3.567164	10.37463	0.245893	8.69951	29.838	-12.3537	42.19171	15.74591
3097.084	4.844828	10.92389	0.266723	8.862637	31.75329	-9.20271	40.956	22.2623
3087.363	4.862069	10.02532	0.248886	9.004414	31.52586	-8.75486	40.28071	21.9568
3087.363	4.862069	10.02532	0.248886	9.004414	31.52586	-8.75486	40.28071	21.9568
2418.186	5.074074	10.82791	0.252969	9.258108	28.88971	-13.9136	42.80329	9.588137
2240.972	4.5	10.24482	0.238973	9.470442	27.75557	-15.1146	42.87014	16.65014
3087.363	4.862069	10.02532	0.248886	9.004414	31.52586	-8.75486	40.28071	21.9568
2669.999	4.174603	11.01735	0.247661	9.074549	29.75129	-14.7344	44.48571	17.00764
3097.084	4.844828	10.92389	0.266723	8.862637	31.75329	-9.20271	40.956	22.2623
3085.205	4.7	10.54885	0.261208	8.905989	31.41229	-8.97257	40.38486	21.88501
2957.386	4.435484	10.50273	0.259459	8.880752	30.82643	-9.65286	40.47929	21.32689
2957.386	4.435484	10.50273	0.259459	8.880752	30.82643	-9.65286	40.47929	21.32689
2662.339	3.584615	10.71813	0.259567	8.442961	27.81257	-13.4797	41.29229	17.99148
3087.363	4.862069	10.02532	0.248886	9.004414	31.52586	-8.75486	40.28071	21.9568
2662.339	3.584615	10.71813	0.259567	8.442961	27.81257	-13.4797	41.29229	17.99148
2967.535	3.779412	10.5232	0.244535	9.00546	29.92871	-13.1049	43.03357	19.39375
2829.6	3.555556	11.10361	0.256655	8.846986	30.21014	-13.0526	43.26271	18.90434
2662.339	3.584615	10.71813	0.259567	8.442961	27.81257	-13.4797	41.29229	17.99148
3087.363	4.862069	10.02532	0.248886	9.004414	31.52586	-8.75486	40.28071	21.9568

Table S2 - Anonymized database

3097.084	4.844828	10.92389	0.266723	8.862637	31.75329	-9.20271	40.956	22.2623
2967.535	3.779412	10.5232	0.244535	9.00546	29.92871	-13.1049	43.03357	19.39375
3146.548	3.910448	10.54269	0.246233	8.918875	30.84171	-11.9743	42.816	20.15043
2661.64	4.655172	10.23391	0.241802	9.129539	29.50586	-12.8177	42.32357	10.81254
3097.084	4.844828	10.92389	0.266723	8.862637	31.75329	-9.20271	40.956	22.2623
3097.084	4.844828	10.92389	0.266723	8.862637	31.75329	-9.20271	40.956	22.2623
2807.883	4.190476	10.83214	0.246376	9.035814	29.79829	-14.1676	43.96586	15.21182
2999.858	4.16129	10.43253	0.242742	8.842099	29.89586	-13.082	42.97786	19.40637
2798.628	4.55	10.7441	0.257734	8.935147	30.38557	-11.3011	41.68671	20.63604
3146.548	3.910448	10.54269	0.246233	8.918875	30.84171	-11.9743	42.816	20.15043
3077.479	4.516129	10.7899	0.264113	8.651458	31.12286	-9.73043	40.85329	21.44169
2957.386	4.435484	10.50273	0.259459	8.880752	30.82643	-9.65286	40.47929	21.32689
2570.41	4.095238	10.31579	0.235489	9.217671	29.51943	-14.2864	43.80586	16.54059
3024.132	4.030769	10.462	0.243544	8.942156	30.22729	-12.7301	42.95743	19.63534
3040.462	4.864407	10.83357	0.264768	8.930403	31.33086	-9.58643	40.91729	21.62754
2518.368	4.459016	11.51837	0.258096	9.220036	29.27157	-15.3567	44.62829	17.88371
2699.456	4.15873	11.4184	0.255565	9.0218	30.08986	-14.5891	44.679	17.1172
2942.86	3.567164	10.37463	0.245893	8.69951	29.838	-12.3537	42.19171	15.74591
3097.084	4.844828	10.92389	0.266723	8.862637	31.75329	-9.20271	40.956	22.2623
2953.704	3.619718	10.85687	0.250288	8.957851	30.26029	-13.1173	43.37757	19.42741
2807.883	4.190476	10.83214	0.246376	9.035814	29.79829	-14.1676	43.96586	15.21182
2953.894	3.642857	11.23202	0.258294	8.934624	30.589	-12.8964	43.48543	19.47527
2718.884	3.921875	8.442255	0.208246	8.998847	28.63929	-11.9006	40.53986	14.11862
3097.084	4.844828	10.92389	0.266723	8.862637	31.75329	-9.20271	40.956	22.2623
2676.135	4.927273	11.26454	0.26263	8.987712	30.43086	-12.4604	42.89129	18.39688
2669.999	4.174603	11.01735	0.247661	9.074549	29.75129	-14.7344	44.48571	17.00764
3118.855	4.046875	10.26652	0.241974	8.910968	30.34643	-12.0817	42.42814	21.63227
3077.479	4.516129	10.7899	0.264113	8.651458	31.12286	-9.73043	40.85329	21.44169
2957.386	4.435484	10.50273	0.259459	8.880752	30.82643	-9.65286	40.47929	21.32689
2807.883	4.190476	10.83214	0.246376	9.035814	29.79829	-14.1676	43.96586	15.21182
2807.883	4.190476	10.83214	0.246376	9.035814	29.79829	-14.1676	43.96586	15.21182
2798.628	4.55	10.7441	0.257734	8.935147	30.38557	-11.3011	41.68671	20.63604
2967.535	3.779412	10.5232	0.244535	9.00546	29.92871	-13.1049	43.03357	19.39375
2676.135	4.927273	11.26454	0.26263	8.987712	30.43086	-12.4604	42.89129	18.39688
2699.456	4.15873	11.4184	0.255565	9.0218	30.08986	-14.5891	44.679	17.1172
3087.363	4.862069	10.02532	0.248886	9.004414	31.52586	-8.75486	40.28071	21.9568
2974.456	4.030769	10.95567	0.250968	8.95459	30.55286	-13.1007	43.65357	19.55171
2799.826	4.542373	11.07159	0.249311	9.105087	30.57157	-13.8371	44.40871	17.4494
2807.883	4.190476	10.83214	0.246376	9.035814	29.79829	-14.1676	43.96586	15.21182
3077.479	4.516129	10.7899	0.264113	8.651458	31.12286	-9.73043	40.85329	21.44169
3040.462	4.864407	10.83357	0.264768	8.930403	31.33086	-9.58643	40.91729	21.62754
3087.363	4.862069	10.02532	0.248886	9.004414	31.52586	-8.75486	40.28071	21.9568
2942.86	3.567164	10.37463	0.245893	8.69951	29.838	-12.3537	42.19171	15.74591
2676.135	4.927273	11.26454	0.26263	8.987712	30.43086	-12.4604	42.89129	18.39688
2687.626	4.542373	10.94315	0.247359	9.105289	29.877	-14.363	44.24	17.0018
3087.363	4.862069	10.02532	0.248886	9.004414	31.52586	-8.75486	40.28071	21.9568
2676.135	4.927273	11.26454	0.26263	8.987712	30.43086	-12.4604	42.89129	18.39688
3097.084	4.844828	10.92389	0.266723	8.862637	31.75329	-9.20271	40.956	22.2623
2676.135	4.927273	11.26454	0.26263	8.987712	30.43086	-12.4604	42.89129	18.39688
2676.135	4.927273	11.26454	0.26263	8.987712	30.43086	-12.4604	42.89129	18.39688

Table S2 - Anonymized database

3024.132	4.030769	10.462	0.243544	8.942156	30.22729	-12.7301	42.95743	19.63534
2799.826	4.542373	11.07159	0.249311	9.105087	30.57157	-13.8371	44.40871	17.4494
2662.339	3.584615	10.71813	0.259567	8.442961	27.81257	-13.4797	41.29229	17.99148
2798.628	4.55	10.7441	0.257734	8.935147	30.38557	-11.3011	41.68671	20.63604
3077.479	4.516129	10.7899	0.264113	8.651458	31.12286	-9.73043	40.85329	21.44169
3087.363	4.862069	10.02532	0.248886	9.004414	31.52586	-8.75486	40.28071	21.9568
2429.035	4.793103	11.21175	0.26066	9.080668	29.153	-13.8599	43.01286	17.34101
3024.132	4.030769	10.462	0.243544	8.942156	30.22729	-12.7301	42.95743	19.63534
2799.826	4.542373	11.07159	0.249311	9.105087	30.57157	-13.8371	44.40871	17.4494
3159.857	4.419355	10.32602	0.255375	8.661225	31.25843	-9.17629	40.43471	21.77787
3173.256	4.862069	10.46421	0.257957	8.889374	31.798	-8.76771	40.56571	22.57521
3087.363	4.862069	10.02532	0.248886	9.004414	31.52586	-8.75486	40.28071	21.9568
2676.135	4.927273	11.26454	0.26263	8.987712	30.43086	-12.4604	42.89129	18.39688
2662.339	3.584615	10.71813	0.259567	8.442961	27.81257	-13.4797	41.29229	17.99148
3038.727	4.728814	9.828841	0.247999	8.850262	30.594	-9.03857	39.63257	21.49053
3097.084	4.844828	10.92389	0.266723	8.862637	31.75329	-9.20271	40.956	22.2623
2799.826	4.542373	11.07159	0.249311	9.105087	30.57157	-13.8371	44.40871	17.4494
3097.084	4.844828	10.92389	0.266723	8.862637	31.75329	-9.20271	40.956	22.2623
3077.479	4.516129	10.7899	0.264113	8.651458	31.12286	-9.73043	40.85329	21.44169
2676.135	4.927273	11.26454	0.26263	8.987712	30.43086	-12.4604	42.89129	18.39688
2676.135	4.927273	11.26454	0.26263	8.987712	30.43086	-12.4604	42.89129	18.39688
3097.084	4.844828	10.92389	0.266723	8.862637	31.75329	-9.20271	40.956	22.2623
2957.386	4.435484	10.50273	0.259459	8.880752	30.82643	-9.65286	40.47929	21.32689
2957.386	4.435484	10.50273	0.259459	8.880752	30.82643	-9.65286	40.47929	21.32689
2953.704	3.619718	10.85687	0.250288	8.957851	30.26029	-13.1173	43.37757	19.42741
2807.883	4.190476	10.83214	0.246376	9.035814	29.79829	-14.1676	43.96586	15.21182
3097.084	4.844828	10.92389	0.266723	8.862637	31.75329	-9.20271	40.956	22.2623
2661.64	4.655172	10.23391	0.241802	9.129539	29.50586	-12.8177	42.32357	10.81254
3077.479	4.516129	10.7899	0.264113	8.651458	31.12286	-9.73043	40.85329	21.44169
2687.626	4.542373	10.94315	0.247359	9.105289	29.877	-14.363	44.24	17.0018
3146.548	3.910448	10.54269	0.246233	8.918875	30.84171	-11.9743	42.816	20.15043
2699.456	4.15873	11.4184	0.255565	9.0218	30.08986	-14.5891	44.679	17.1172
2718.884	3.921875	8.442255	0.208246	8.998847	28.63929	-11.9006	40.53986	14.11862
3024.132	4.030769	10.462	0.243544	8.942156	30.22729	-12.7301	42.95743	19.63534
2661.64	4.655172	10.23391	0.241802	9.129539	29.50586	-12.8177	42.32357	10.81254
3143.601	3.848485	9.72933	0.204933	9.861557	32.02443	-15.4513	47.47571	23.28599
2513.247	3.088608	9.5855	0.203461	10.44502	28.597	-18.5153	47.11229	13.03295
3049.273	4.063492	9.502195	0.20322	9.824579	31.45443	-15.3037	46.75814	21.94865
2833.384	3.666667	10.54802	0.218217	10.18309	31.266	-17.0713	48.33729	21.86225
2955.56	4.147541	9.553184	0.195655	10.03982	31.40329	-17.4234	48.82671	21.63962
2645.201	3.569444	10.45767	0.208788	10.33064	29.44329	-20.6443	50.08757	13.41331
3079.435	3.342466	9.668865	0.194996	10.36921	31.41586	-18.1691	49.585	23.25553
2703.508	3.637681	10.27859	0.206883	10.33822	30.76129	-18.9217	49.683	20.56579
3124.07	3.848485	9.289995	0.197856	9.90706	31.66843	-15.2849	46.95329	22.29269
2971.976	3.507042	9.839538	0.196129	10.30251	30.57086	-19.5979	50.16871	11.47576
2729.098	3.833333	10.26425	0.20757	10.15239	31.23957	-18.2101	49.44971	20.62871
2334.449	3.444444	9.741253	0.198649	10.44967	28.95714	-20.0804	49.03757	20.41729
2818.002	3.118421	10.37036	0.198005	10.45229	30.28257	-22.0917	52.37429	22.04617
2923.503	3.148649	10.07393	0.197836	10.34481	30.52557	-20.395	50.92057	22.45725
2816.375	4.225806	10.11058	0.201421	10.25443	31.65743	-18.5389	50.19629	21.09371

Table S2 - Anonymized database

3355.584	3.823529	8.960777	0.194525	9.82302	32.09971	-13.9653	46.065	23.33846
2720.62	3.924242	9.211519	0.193151	10.20257	30.18943	-17.5013	47.69071	20.48316
3066.458	4.095238	10.02045	0.20307	10.07719	31.792	-17.5527	49.34471	22.81733
3039.765	4.16129	9.341959	0.20026	9.829737	31.31429	-15.3349	46.64914	21.90146
2955.821	4.140625	9.83131	0.204391	9.894741	31.40057	-16.6999	48.10043	21.73634
2832.254	3.757576	10.66365	0.212504	10.19024	32.00971	-18.1711	50.18086	21.19691
2997.424	3.865672	9.59628	0.200211	9.834272	31.40686	-16.524	47.93086	21.7804
3043.4	4.666667	8.338459	0.183626	9.777416	30.71686	-14.6931	45.41	21.82645
3088.452	4.095238	9.72039	0.204079	9.84008	31.868	-15.7624	47.63043	23.05503
2969.13	3.876923	9.648569	0.198923	10.03029	31.83686	-16.6673	48.50414	21.68879
2560.472	3.720588	8.935824	0.184992	10.23391	28.98629	-19.3176	48.30386	19.71297
2922.614	3.236111	10.04527	0.198393	10.28965	30.32329	-20.31	50.63329	21.06594
2206.389	3.434211	9.759786	0.202076	10.26453	27.81157	-20.4861	48.29771	18.98945
3088.305	4.35	9.927816	0.204093	10.03794	32.08886	-16.5547	48.64357	22.1844
2969.13	3.876923	9.648569	0.198923	10.03029	31.83686	-16.6673	48.50414	21.68879
3071.319	3.118421	10.67653	0.210396	10.22518	31.22514	-19.5197	50.74486	19.21593
3043.4	4.666667	8.338459	0.183626	9.777416	30.71686	-14.6931	45.41	21.82645
2729.098	3.833333	10.26425	0.20757	10.15239	31.23957	-18.2101	49.44971	20.62871
2833.384	3.666667	10.54802	0.218217	10.18309	31.266	-17.0713	48.33729	21.86225
2860.531	3.444444	9.393533	0.200529	10.19238	30.72586	-16.118	46.84386	22.39519
3101.577	4.0625	9.365044	0.20081	9.821972	31.23814	-15.3983	46.63643	22.05755
3089.078	3.850746	10.15646	0.208547	9.921092	32.163	-16.5381	48.70114	23.13301
2550.962	3.486111	9.757453	0.199574	10.23631	30.00514	-18.8861	48.89129	20.20426
3080.584	4.131148	9.272258	0.191442	10.11107	32.01786	-16.416	48.43386	23.07747
3036.646	4.245902	9.330536	0.193925	10.15762	31.97314	-16.141	48.11414	22.95326
2828.368	3.80303	9.689591	0.202829	10.269	31.03414	-16.738	47.77214	22.14646
2703.508	3.637681	10.27859	0.206883	10.33822	30.76129	-18.9217	49.683	20.56579
3326.608	3.619718	8.961124	0.193185	9.86121	32.33814	-14.0481	46.38629	13.45416
3124.07	3.848485	9.289995	0.197856	9.90706	31.66843	-15.2849	46.95329	22.29269
2345.926	3.266667	9.0665	0.186425	10.52026	28.78571	-19.8477	48.63343	20.50251
3043.4	4.666667	8.338459	0.183626	9.777416	30.71686	-14.6931	45.41	21.82645
2703.508	3.637681	10.27859	0.206883	10.33822	30.76129	-18.9217	49.683	20.56579
2345.926	3.266667	9.0665	0.186425	10.52026	28.78571	-19.8477	48.63343	20.50251
3043.4	4.666667	8.338459	0.183626	9.777416	30.71686	-14.6931	45.41	21.82645
2729.098	3.833333	10.26425	0.20757	10.15239	31.23957	-18.2101	49.44971	20.62871
2578.469	3.108108	8.448527	0.179642	10.28123	27.71686	-19.313	47.02986	19.43304
2969.13	3.876923	9.648569	0.198923	10.03029	31.83686	-16.6673	48.50414	21.68879
2345.926	3.266667	9.0665	0.186425	10.52026	28.78571	-19.8477	48.63343	20.50251
3088.305	4.35	9.927816	0.204093	10.03794	32.08886	-16.5547	48.64357	22.1844
2715.243	3.597222	10.52134	0.209668	10.40458	29.67014	-20.5107	50.18086	13.72431
2951.18	3.053333	10.2714	0.201403	10.33048	30.78543	-20.2139	50.99929	21.25676
2746.081	3.081081	9.047838	0.191149	10.30719	28.64843	-18.6854	47.33386	10.59751
3036.646	4.245902	9.330536	0.193925	10.15762	31.97314	-16.141	48.11414	22.95326
2720.62	3.924242	9.211519	0.193151	10.20257	30.18943	-17.5013	47.69071	20.48316
2971.976	3.507042	9.839538	0.196129	10.30251	30.57086	-19.5979	50.16871	11.47576
2729.098	3.833333	10.26425	0.20757	10.15239	31.23957	-18.2101	49.44971	20.62871
2870.028	3.830769	9.322591	0.191365	10.07967	31.28729	-17.429	48.71629	22.80193
3043.4	4.666667	8.338459	0.183626	9.777416	30.71686	-14.6931	45.41	21.82645
2774.309	3.521739	9.703014	0.202362	9.963909	30.53629	-17.4124	47.94871	20.75152
3043.4	4.666667	8.338459	0.183626	9.777416	30.71686	-14.6931	45.41	21.82645

Table S2 - Anonymized database

2676.259	3.272727	10.39109	0.21017	10.40598	29.52929	-19.9121	49.44143	13.31791
2720.62	3.924242	9.211519	0.193151	10.20257	30.18943	-17.5013	47.69071	20.48316
2818.002	3.118421	10.37036	0.198005	10.45229	30.28257	-22.0917	52.37429	22.04617
2833.384	3.666667	10.54802	0.218217	10.18309	31.266	-17.0713	48.33729	21.86225
3124.07	3.848485	9.289995	0.197856	9.90706	31.66843	-15.2849	46.95329	22.29269
2626.119	3.089744	9.693341	0.202767	10.41368	29.196	-18.6094	47.80543	13.31518
2720.62	3.924242	9.211519	0.193151	10.20257	30.18943	-17.5013	47.69071	20.48316
2786.842	3.459459	10.20735	0.2062	10.50743	30.02157	-19.4807	49.50229	13.98523
2832.254	3.757576	10.66365	0.212504	10.19024	32.00971	-18.1711	50.18086	21.19691
2438.76	3.666667	10.27292	0.207676	10.36404	29.74414	-19.722	49.46614	20.88198
3067.424	3.80597	9.968374	0.208515	9.825926	31.84486	-15.9617	47.80657	22.07466
2971.976	3.507042	9.839538	0.196129	10.30251	30.57086	-19.5979	50.16871	11.47576
2844.379	3.457143	9.777613	0.196765	10.34554	29.874	-19.8179	49.69186	17.44609
2662.224	3.742424	9.530261	0.19745	10.28178	29.99014	-18.2766	48.26671	20.23103
3043.4	4.666667	8.338459	0.183626	9.777416	30.71686	-14.6931	45.41	21.82645
2513.247	3.088608	9.5855	0.203461	10.44502	28.597	-18.5153	47.11229	13.03295
2971.976	3.507042	9.839538	0.196129	10.30251	30.57086	-19.5979	50.16871	11.47576
3071.319	3.118421	10.67653	0.210396	10.22518	31.22514	-19.5197	50.74486	19.21593
2969.13	3.876923	9.648569	0.198923	10.03029	31.83686	-16.6673	48.50414	21.68879
2774.309	3.521739	9.703014	0.202362	9.963909	30.53629	-17.4124	47.94871	20.75152
3047.869	3.56338	9.76008	0.198343	10.34188	30.90171	-18.3063	49.208	11.8681
3123.806	4.09375	9.686827	0.205102	9.774654	31.555	-15.6743	47.22929	23.08931
2606.346	3.632353	10.4301	0.211153	9.995482	31.43443	-17.9614	49.39586	19.88637
2774.309	3.521739	9.703014	0.202362	9.963909	30.53629	-17.4124	47.94871	20.75152
3043.4	4.666667	8.338459	0.183626	9.777416	30.71686	-14.6931	45.41	21.82645
3052.728	4.09375	10.09171	0.206352	9.916827	32.14229	-16.7631	48.90543	22.94353
2578.469	3.108108	8.448527	0.179642	10.28123	27.71686	-19.313	47.02986	19.43304
3043.4	4.666667	8.338459	0.183626	9.777416	30.71686	-14.6931	45.41	21.82645
3161.414	3.818182	9.904945	0.208156	9.826251	32.09357	-15.4906	47.58414	23.34493
2969.13	3.876923	9.648569	0.198923	10.03029	31.83686	-16.6673	48.50414	21.68879
2720.62	3.924242	9.211519	0.193151	10.20257	30.18943	-17.5013	47.69071	20.48316
3101.577	4.0625	9.365044	0.20081	9.821972	31.23814	-15.3983	46.63643	22.05755
3071.319	3.118421	10.67653	0.210396	10.22518	31.22514	-19.5197	50.74486	19.21593
2940.962	3.907692	9.768635	0.205861	9.845118	31.60629	-15.8463	47.45257	21.61192
2720.62	3.924242	9.211519	0.193151	10.20257	30.18943	-17.5013	47.69071	20.48316
3146.277	4.163934	8.829821	0.191257	9.872304	31.41214	-14.7553	46.16743	22.35059
3036.646	4.245902	9.330536	0.193925	10.15762	31.97314	-16.141	48.11414	22.95326
2720.62	3.924242	9.211519	0.193151	10.20257	30.18943	-17.5013	47.69071	20.48316
3052.728	4.09375	10.09171	0.206352	9.916827	32.14229	-16.7631	48.90543	22.94353
2560.472	3.720588	8.935824	0.184992	10.23391	28.98629	-19.3176	48.30386	19.71297
2925.869	3.550725	9.70975	0.195793	10.40932	30.23286	-19.3591	49.592	22.56089
3146.171	4.129032	9.04708	0.188025	10.1896	32.14929	-15.9671	48.11643	23.42611
2997.424	3.865672	9.59628	0.200211	9.834272	31.40686	-16.524	47.93086	21.7804
2720.62	3.924242	9.211519	0.193151	10.20257	30.18943	-17.5013	47.69071	20.48316
3360.511	3.676471	9.281052	0.197939	9.89741	32.93729	-13.9511	46.88843	23.31316
2715.243	3.597222	10.52134	0.209668	10.40458	29.67014	-20.5107	50.18086	13.72431
3124.07	3.848485	9.289995	0.197856	9.90706	31.66843	-15.2849	46.95329	22.29269
3011.265	4.078125	10.22993	0.208462	9.95724	31.96543	-17.1079	49.07329	22.03953
3043.4	4.666667	8.338459	0.183626	9.777416	30.71686	-14.6931	45.41	21.82645
3052.728	4.09375	10.09171	0.206352	9.916827	32.14229	-16.7631	48.90543	22.94353

Table S2 - Anonymized database

2917.31	3.408451	9.877948	0.198858	10.37929	30.34514	-19.3283	49.67343	17.77062
3516.29	3.577465	8.945646	0.195036	9.882489	32.96786	-12.8987	45.86657	14.22615
2923.503	3.148649	10.07393	0.197836	10.34481	30.52557	-20.395	50.92057	22.45725
3025.46	4.03125	9.863706	0.207216	9.835521	31.501	-16.1	47.601	22.77145
3025.46	4.03125	9.863706	0.207216	9.835521	31.501	-16.1	47.601	22.77145
2844.379	3.457143	9.777613	0.196765	10.34554	29.874	-19.8179	49.69186	17.44609
2396.535	3.337838	10.65104	0.212531	10.44231	28.519	-21.5961	50.11514	19.19645
2617.909	3.608696	10.25447	0.209609	10.15016	30.47357	-18.4484	48.922	21.03759
2774.309	3.521739	9.703014	0.202362	9.963909	30.53629	-17.4124	47.94871	20.75152
3009.203	3.628571	9.71153	0.197405	10.3599	30.71229	-18.4837	49.196	11.6938
3089.078	3.850746	10.15646	0.208547	9.921092	32.163	-16.5381	48.70114	23.13301
3066.458	4.095238	10.02045	0.20307	10.07719	31.792	-17.5527	49.34471	22.81733
3516.29	3.577465	8.945646	0.195036	9.882489	32.96786	-12.8987	45.86657	14.22615
2729.098	3.833333	10.26425	0.20757	10.15239	31.23957	-18.2101	49.44971	20.62871
2610.051	3.213333	9.584712	0.202486	10.53074	29.096	-18.2391	47.33514	15.18559
3079.435	3.342466	9.668865	0.194996	10.36921	31.41586	-18.1691	49.585	23.25553
2513.247	3.088608	9.5855	0.203461	10.44502	28.597	-18.5153	47.11229	13.03295
2774.309	3.521739	9.703014	0.202362	9.963909	30.53629	-17.4124	47.94871	20.75152
3025.46	4.03125	9.863706	0.207216	9.835521	31.501	-16.1	47.601	22.77145
3011.265	4.078125	10.22993	0.208462	9.95724	31.96543	-17.1079	49.07329	22.03953
2703.508	3.637681	10.27859	0.206883	10.33822	30.76129	-18.9217	49.683	20.56579
2774.309	3.521739	9.703014	0.202362	9.963909	30.53629	-17.4124	47.94871	20.75152
2987.64	3.585714	9.621104	0.194823	10.3977	30.63357	-18.7501	49.38371	22.88703
2860.531	3.444444	9.393533	0.200529	10.19238	30.72586	-16.118	46.84386	22.39519
2623.688	3.380282	10.20546	0.207272	10.0447	31.07271	-18.1644	49.23714	20.00774
2818.002	3.118421	10.37036	0.198005	10.45229	30.28257	-22.0917	52.37429	22.04617
3157.675	4.131148	9.31247	0.192054	10.1149	32.12743	-16.3613	48.48871	22.48246
3096.618	4.063492	9.568456	0.202182	9.796947	31.35657	-15.9694	47.326	22.03666
2550.962	3.486111	9.757453	0.199574	10.23631	30.00514	-18.8861	48.89129	20.20426
3132.089	4.15873	9.632898	0.203246	10.0056	32.07814	-15.3171	47.39529	22.36384
2969.13	3.876923	9.648569	0.198923	10.03029	31.83686	-16.6673	48.50414	21.68879
3329.211	3.823529	8.944313	0.193079	9.856359	32.189	-14.1356	46.32457	13.50888
3079.162	4.241935	10.34929	0.208999	9.971054	32.49586	-17.0226	49.51843	23.08395
2578.469	3.108108	8.448527	0.179642	10.28123	27.71686	-19.313	47.02986	19.43304
2720.62	3.924242	9.211519	0.193151	10.20257	30.18943	-17.5013	47.69071	20.48316
2560.472	3.720588	8.935824	0.184992	10.23391	28.98629	-19.3176	48.30386	19.71297
3041.622	3.066667	10.70262	0.210772	10.22507	30.71586	-20.0623	50.77814	19.18052
2206.389	3.434211	9.759786	0.202076	10.26453	27.81157	-20.4861	48.29771	18.98945
2922.614	3.236111	10.04527	0.198393	10.28965	30.32329	-20.31	50.63329	21.06594
3326.608	3.619718	8.961124	0.193185	9.86121	32.33814	-14.0481	46.38629	13.45416
3047.869	3.56338	9.76008	0.198343	10.34188	30.90171	-18.3063	49.208	11.8681
2990.374	3.9375	10.12846	0.208523	10.01187	32.73057	-15.8417	48.57229	21.84768
2971.976	3.507042	9.839538	0.196129	10.30251	30.57086	-19.5979	50.16871	11.47576
3039.765	4.16129	9.341959	0.20026	9.829737	31.31429	-15.3349	46.64914	21.90146
2593.717	3.441176	9.290489	0.195748	10.15841	28.16857	-19.2929	47.46143	19.6807
2438.76	3.666667	10.27292	0.207676	10.36404	29.74414	-19.722	49.46614	20.88198
2951.18	3.053333	10.2714	0.201403	10.33048	30.78543	-20.2139	50.99929	21.25676
3016.139	3.478873	9.383418	0.190313	10.37424	30.54371	-18.7614	49.30514	21.4313
3039.765	4.16129	9.341959	0.20026	9.829737	31.31429	-15.3349	46.64914	21.90146
2206.389	3.434211	9.759786	0.202076	10.26453	27.81157	-20.4861	48.29771	18.98945

Table S2 - Anonymized database

3041.622	3.066667	10.70262	0.210772	10.22507	30.71586	-20.0623	50.77814	19.18052
2463.622	3.150685	9.611519	0.200193	10.07934	27.71114	-20.3001	48.01129	18.82339
2345.926	3.266667	9.0665	0.186425	10.52026	28.78571	-19.8477	48.63343	20.50251
2990.374	3.9375	10.12846	0.208523	10.01187	32.73057	-15.8417	48.57229	21.84768
2786.908	3.492958	9.800266	0.210128	10.2229	30.51757	-16.122	46.63957	22.05495
2774.309	3.521739	9.703014	0.202362	9.963909	30.53629	-17.4124	47.94871	20.75152
2345.926	3.266667	9.0665	0.186425	10.52026	28.78571	-19.8477	48.63343	20.50251
3023.486	3.273973	9.743431	0.194456	10.34775	30.96843	-19.1377	50.10614	21.54044
2951.18	3.053333	10.2714	0.201403	10.33048	30.78543	-20.2139	50.99929	21.25676
2345.926	3.266667	9.0665	0.186425	10.52026	28.78571	-19.8477	48.63343	20.50251
3047.869	3.56338	9.76008	0.198343	10.34188	30.90171	-18.3063	49.208	11.8681
2720.62	3.924242	9.211519	0.193151	10.20257	30.18943	-17.5013	47.69071	20.48316
2997.424	3.865672	9.59628	0.200211	9.834272	31.40686	-16.524	47.93086	21.7804
3036.521	4.098361	9.444434	0.194661	10.04042	31.90829	-16.609	48.51729	21.97524
2513.247	3.088608	9.5855	0.203461	10.44502	28.597	-18.5153	47.11229	13.03295
3043.4	4.666667	8.338459	0.183626	9.777416	30.71686	-14.6931	45.41	21.82645
2550.962	3.486111	9.757453	0.199574	10.23631	30.00514	-18.8861	48.89129	20.20426
2940.962	3.907692	9.768635	0.205861	9.845118	31.60629	-15.8463	47.45257	21.61192
2550.962	3.486111	9.757453	0.199574	10.23631	30.00514	-18.8861	48.89129	20.20426
3101.577	4.0625	9.365044	0.20081	9.821972	31.23814	-15.3983	46.63643	22.05755
3016.139	3.478873	9.383418	0.190313	10.37424	30.54371	-18.7614	49.30514	21.4313
3101.577	4.0625	9.365044	0.20081	9.821972	31.23814	-15.3983	46.63643	22.05755
3171.111	4.047619	8.83475	0.184868	10.20737	32.19429	-15.5951	47.78943	23.54588
2355.08	3.507042	11.32059	0.223415	10.31362	29.91371	-20.7569	50.67057	18.80301
2912.877	3.148649	10.111	0.198425	10.32885	30.51443	-20.4419	50.95629	22.40084
2513.247	3.088608	9.5855	0.203461	10.44502	28.597	-18.5153	47.11229	13.03295
2824.231	3.892308	10.20289	0.211236	9.986661	31.64171	-16.6593	48.301	21.143
3043.4	4.666667	8.338459	0.183626	9.777416	30.71686	-14.6931	45.41	21.82645
2992.128	3.380282	10.1615	0.200691	10.28589	30.84043	-19.7921	50.63257	11.59315
2645.201	3.569444	10.45767	0.208788	10.33064	29.44329	-20.6443	50.08757	13.41331
3146.171	4.129032	9.04708	0.188025	10.1896	32.14929	-15.9671	48.11643	23.42611
2438.76	3.666667	10.27292	0.207676	10.36404	29.74414	-19.722	49.46614	20.88198
3432.799	3.642857	8.921	0.191537	9.942755	33.01786	-13.558	46.57586	13.77671
3222.86	4.288136	8.841277	0.182143	10.18231	32.28086	-16.2594	48.54029	23.67093
2676.259	3.272727	10.39109	0.21017	10.40598	29.52929	-19.9121	49.44143	13.31791
2818.002	3.118421	10.37036	0.198005	10.45229	30.28257	-22.0917	52.37429	22.04617
3020.883	4.112903	9.512231	0.202034	9.840979	31.40357	-15.6789	47.08243	21.8846
2513.247	3.088608	9.5855	0.203461	10.44502	28.597	-18.5153	47.11229	13.03295
2832.254	3.757576	10.66365	0.212504	10.19024	32.00971	-18.1711	50.18086	21.19691
2720.62	3.924242	9.211519	0.193151	10.20257	30.18943	-17.5013	47.69071	20.48316
2396.535	3.337838	10.65104	0.212531	10.44231	28.519	-21.5961	50.11514	19.19645
3157.675	4.131148	9.31247	0.192054	10.1149	32.12743	-16.3613	48.48871	22.48246
3079.162	4.241935	10.34929	0.208999	9.971054	32.49586	-17.0226	49.51843	23.08395
3124.07	3.848485	9.289995	0.197856	9.90706	31.66843	-15.2849	46.95329	22.29269
2925.869	3.550725	9.70975	0.195793	10.40932	30.23286	-19.3591	49.592	22.56089
3047.869	3.56338	9.76008	0.198343	10.34188	30.90171	-18.3063	49.208	11.8681
2438.76	3.666667	10.27292	0.207676	10.36404	29.74414	-19.722	49.46614	20.88198
2355.08	3.507042	11.32059	0.223415	10.31362	29.91371	-20.7569	50.67057	18.80301
2952.344	3.424658	9.764739	0.196614	10.43063	30.56929	-19.0951	49.66443	11.56935
2860.531	3.444444	9.393533	0.200529	10.19238	30.72586	-16.118	46.84386	22.39519

Table S2 - Anonymized database

2818.002	3.118421	10.37036	0.198005	10.45229	30.28257	-22.0917	52.37429	22.04617
2979.006	3.514286	9.7355	0.197383	10.40052	30.39414	-18.9287	49.32286	22.82912
3169.654	3.619718	9.142538	0.196486	9.755498	31.73257	-14.7977	46.53029	12.84766
3052.366	4.129032	9.698566	0.200726	10.05568	31.85843	-16.459	48.31743	22.07512
3007.729	3.923077	9.609654	0.200674	9.936124	32.10071	-15.7863	47.887	21.88538
3052.728	4.09375	10.09171	0.206352	9.916827	32.14229	-16.7631	48.90543	22.94353
3101.577	4.0625	9.365044	0.20081	9.821972	31.23814	-15.3983	46.63643	22.05755
2676.259	3.272727	10.39109	0.21017	10.40598	29.52929	-19.9121	49.44143	13.31791
2987.64	3.585714	9.621104	0.194823	10.3977	30.63357	-18.7501	49.38371	22.88703
3063.674	3.892308	9.501245	0.202792	9.763144	31.00114	-15.851	46.85214	21.86049
3109.165	4.092308	9.821316	0.205086	9.853863	31.83357	-16.0553	47.88886	23.22146
2719.538	3.80597	10.14394	0.204419	10.16886	31.16057	-18.4626	49.62314	20.62244
2355.08	3.507042	11.32059	0.223415	10.31362	29.91371	-20.7569	50.67057	18.80301
3124.07	3.848485	9.289995	0.197856	9.90706	31.66843	-15.2849	46.95329	22.29269
3171.111	4.047619	8.83475	0.184868	10.20737	32.19429	-15.5951	47.78943	23.54588
2917.31	3.408451	9.877948	0.198858	10.37929	30.34514	-19.3283	49.67343	17.77062
2513.247	3.088608	9.5855	0.203461	10.44502	28.597	-18.5153	47.11229	13.03295
3009.203	3.628571	9.71153	0.197405	10.3599	30.71229	-18.4837	49.196	11.6938
3184.869	4.079365	9.347069	0.202388	9.779507	31.50871	-14.6751	46.18386	23.39989
2206.389	3.434211	9.759786	0.202076	10.26453	27.81157	-20.4861	48.29771	18.98945
3088.452	4.095238	9.72039	0.204079	9.84008	31.868	-15.7624	47.63043	23.05503
2940.962	3.907692	9.768635	0.205861	9.845118	31.60629	-15.8463	47.45257	21.61192
3011.265	4.078125	10.22993	0.208462	9.95724	31.96543	-17.1079	49.07329	22.03953
3042.166	3.681818	9.684217	0.200545	9.978877	31.73586	-16.5537	48.28957	21.85101
2989.027	3.361111	9.706876	0.196767	10.39114	30.61429	-18.7176	49.33186	22.89026
2720.62	3.924242	9.211519	0.193151	10.20257	30.18943	-17.5013	47.69071	20.48316
2637.779	3.210526	9.619613	0.201082	10.43597	29.08286	-18.7563	47.83914	13.39855
3071.319	3.118421	10.67653	0.210396	10.22518	31.22514	-19.5197	50.74486	19.21593
2844.379	3.457143	9.777613	0.196765	10.34554	29.874	-19.8179	49.69186	17.44609
2971.976	3.507042	9.839538	0.196129	10.30251	30.57086	-19.5979	50.16871	11.47576
3025.46	4.03125	9.863706	0.207216	9.835521	31.501	-16.1	47.601	22.77145
3036.521	4.098361	9.444434	0.194661	10.04042	31.90829	-16.609	48.51729	21.97524
2818.002	3.118421	10.37036	0.198005	10.45229	30.28257	-22.0917	52.37429	22.04617
2593.717	3.441176	9.290489	0.195748	10.15841	28.16857	-19.2929	47.46143	19.6807
2940.962	3.907692	9.768635	0.205861	9.845118	31.60629	-15.8463	47.45257	21.61192
3020.883	4.112903	9.512231	0.202034	9.840979	31.40357	-15.6789	47.08243	21.8846
2396.535	3.337838	10.65104	0.212531	10.44231	28.519	-21.5961	50.11514	19.19645
2720.62	3.924242	9.211519	0.193151	10.20257	30.18943	-17.5013	47.69071	20.48316
3020.883	4.112903	9.512231	0.202034	9.840979	31.40357	-15.6789	47.08243	21.8846
2355.08	3.507042	11.32059	0.223415	10.31362	29.91371	-20.7569	50.67057	18.80301
2206.389	3.434211	9.759786	0.202076	10.26453	27.81157	-20.4861	48.29771	18.98945
3011.265	4.078125	10.22993	0.208462	9.95724	31.96543	-17.1079	49.07329	22.03953
3039.765	4.16129	9.341959	0.20026	9.829737	31.31429	-15.3349	46.64914	21.90146
3023.486	3.273973	9.743431	0.194456	10.34775	30.96843	-19.1377	50.10614	21.54044
3157.675	4.131148	9.31247	0.192054	10.1149	32.12743	-16.3613	48.48871	22.48246
3132.089	4.15873	9.632898	0.203246	10.0056	32.07814	-15.3171	47.39529	22.36384
3123.806	4.09375	9.686827	0.205102	9.774654	31.555	-15.6743	47.22929	23.08931
2926.277	3.8	10.21629	0.214888	10.05581	30.92871	-16.6137	47.54243	21.35853
2206.389	3.434211	9.759786	0.202076	10.26453	27.81157	-20.4861	48.29771	18.98945
3124.07	3.848485	9.289995	0.197856	9.90706	31.66843	-15.2849	46.95329	22.29269

Table S2 - Anonymized database

3011.265	4.078125	10.22993	0.208462	9.95724	31.96543	-17.1079	49.07329	22.03953
2506.682	3.637681	8.851797	0.185123	10.22822	28.75129	-19.0644	47.81571	19.48459
2606.346	3.632353	10.4301	0.211153	9.995482	31.43443	-17.9614	49.39586	19.88637
2438.76	3.666667	10.27292	0.207676	10.36404	29.74414	-19.722	49.46614	20.88198
3160.261	4.109375	9.709338	0.204196	9.885808	32.01771	-15.5313	47.549	21.68932
3043.4	4.666667	8.338459	0.183626	9.777416	30.71686	-14.6931	45.41	21.82645
2774.309	3.521739	9.703014	0.202362	9.963909	30.53629	-17.4124	47.94871	20.75152
3043.4	4.666667	8.338459	0.183626	9.777416	30.71686	-14.6931	45.41	21.82645
3096.618	4.063492	9.568456	0.202182	9.796947	31.35657	-15.9694	47.326	22.03666
2598.688	3.438356	9.616374	0.201647	10.44363	29.066	-18.6231	47.68914	13.24516
3052.728	4.09375	10.09171	0.206352	9.916827	32.14229	-16.7631	48.90543	22.94353
3036.521	4.098361	9.444434	0.194661	10.04042	31.90829	-16.609	48.51729	21.97524
3160.261	4.109375	9.709338	0.204196	9.885808	32.01771	-15.5313	47.549	21.68932
3071.319	3.118421	10.67653	0.210396	10.22518	31.22514	-19.5197	50.74486	19.21593
3124.07	3.848485	9.289995	0.197856	9.90706	31.66843	-15.2849	46.95329	22.29269
2513.247	3.088608	9.5855	0.203461	10.44502	28.597	-18.5153	47.11229	13.03295
2774.309	3.521739	9.703014	0.202362	9.963909	30.53629	-17.4124	47.94871	20.75152
2720.62	3.924242	9.211519	0.193151	10.20257	30.18943	-17.5013	47.69071	20.48316
2617.909	3.608696	10.25447	0.209609	10.15016	30.47357	-18.4484	48.922	21.03759
2940.962	3.907692	9.768635	0.205861	9.845118	31.60629	-15.8463	47.45257	21.61192
2987.64	3.585714	9.621104	0.194823	10.3977	30.63357	-18.7501	49.38371	22.88703
3011.265	4.078125	10.22993	0.208462	9.95724	31.96543	-17.1079	49.07329	22.03953
3011.265	4.078125	10.22993	0.208462	9.95724	31.96543	-17.1079	49.07329	22.03953
3011.265	4.078125	10.22993	0.208462	9.95724	31.96543	-17.1079	49.07329	22.03953
3071.319	3.118421	10.67653	0.210396	10.22518	31.22514	-19.5197	50.74486	19.21593
2637.797	3.647059	10.26389	0.209853	10.07175	30.58243	-18.3276	48.91	20.10075
3146.277	4.163934	8.829821	0.191257	9.872304	31.41214	-14.7553	46.16743	22.35059
3066.458	4.095238	10.02045	0.20307	10.07719	31.792	-17.5527	49.34471	22.81733
3326.608	3.619718	8.961124	0.193185	9.86121	32.33814	-14.0481	46.38629	13.45416
2720.62	3.924242	9.211519	0.193151	10.20257	30.18943	-17.5013	47.69071	20.48316
3052.728	4.09375	10.09171	0.206352	9.916827	32.14229	-16.7631	48.90543	22.94353
3036.646	4.245902	9.330536	0.193925	10.15762	31.97314	-16.141	48.11414	22.95326
3096.618	4.063492	9.568456	0.202182	9.796947	31.35657	-15.9694	47.326	22.03666
2833.384	3.666667	10.54802	0.218217	10.18309	31.266	-17.0713	48.33729	21.86225
2355.08	3.507042	11.32059	0.223415	10.31362	29.91371	-20.7569	50.67057	18.80301
3043.4	4.666667	8.338459	0.183626	9.777416	30.71686	-14.6931	45.41	21.82645
2833.384	3.666667	10.54802	0.218217	10.18309	31.266	-17.0713	48.33729	21.86225
3173.402	3.921875	9.196533	0.198058	9.808554	31.55871	-14.8749	46.43357	22.51121
3079.435	3.342466	9.668865	0.194996	10.36921	31.41586	-18.1691	49.585	23.25553
2484.856	3.637681	10.41304	0.208482	10.37992	29.147	-20.8	49.947	19.61129
3096.618	4.063492	9.568456	0.202182	9.796947	31.35657	-15.9694	47.326	22.03666
3043.4	4.666667	8.338459	0.183626	9.777416	30.71686	-14.6931	45.41	21.82645
2987.64	3.585714	9.621104	0.194823	10.3977	30.63357	-18.7501	49.38371	22.88703
2206.389	3.434211	9.759786	0.202076	10.26453	27.81157	-20.4861	48.29771	18.98945
3129.049	4.2	9.489887	0.194885	10.12341	32.04429	-16.6506	48.69486	23.24579
2833.384	3.666667	10.54802	0.218217	10.18309	31.266	-17.0713	48.33729	21.86225
2729.098	3.833333	10.26425	0.20757	10.15239	31.23957	-18.2101	49.44971	20.62871
2746.081	3.081081	9.047838	0.191149	10.30719	28.64843	-18.6854	47.33386	10.59751
3088.305	4.35	9.927816	0.204093	10.03794	32.08886	-16.5547	48.64357	22.1844
3036.521	4.098361	9.444434	0.194661	10.04042	31.90829	-16.609	48.51729	21.97524

Table S2 - Anonymized database

2940.962	3.907692	9.768635	0.205861	9.845118	31.60629	-15.8463	47.45257	21.61192
3009.203	3.628571	9.71153	0.197405	10.3599	30.71229	-18.4837	49.196	11.6938
3157.675	4.131148	9.31247	0.192054	10.1149	32.12743	-16.3613	48.48871	22.48246
2355.08	3.507042	11.32059	0.223415	10.31362	29.91371	-20.7569	50.67057	18.80301
3123.806	4.09375	9.686827	0.205102	9.774654	31.555	-15.6743	47.22929	23.08931
3132.831	3.742424	9.895681	0.207888	9.981043	31.94986	-15.6511	47.601	22.23615
3088.452	4.095238	9.72039	0.204079	9.84008	31.868	-15.7624	47.63043	23.05503
2703.508	3.637681	10.27859	0.206883	10.33822	30.76129	-18.9217	49.683	20.56579
3065.358	3.764706	9.782505	0.206515	9.84485	31.603	-15.7666	47.36957	22.95677
3030.293	3.450704	9.858533	0.198719	10.33327	30.87457	-18.7359	49.61043	11.79528
2940.962	3.907692	9.768635	0.205861	9.845118	31.60629	-15.8463	47.45257	21.61192
3146.277	4.163934	8.829821	0.191257	9.872304	31.41214	-14.7553	46.16743	22.35059
3232.691	4.16129	9.788929	0.208078	9.755859	32.24229	-14.8023	47.04457	24.2802
3043.4	4.666667	8.338459	0.183626	9.777416	30.71686	-14.6931	45.41	21.82645
2720.62	3.924242	9.211519	0.193151	10.20257	30.18943	-17.5013	47.69071	20.48316
3253.218	4.274194	9.606536	0.202983	9.875097	32.23571	-15.091	47.32671	13.2132
2821.627	3.535211	9.850964	0.207337	10.20544	30.81671	-16.6951	47.51186	21.74653
3146.171	4.129032	9.04708	0.188025	10.1896	32.14929	-15.9671	48.11643	23.42611
3052.366	4.129032	9.698566	0.200726	10.05568	31.85843	-16.459	48.31743	22.07512
2729.098	3.833333	10.26425	0.20757	10.15239	31.23957	-18.2101	49.44971	20.62871
3232.691	4.16129	9.788929	0.208078	9.755859	32.24229	-14.8023	47.04457	24.2802
2513.247	3.088608	9.5855	0.203461	10.44502	28.597	-18.5153	47.11229	13.03295
2718.073	3.12	9.238385	0.192095	10.2745	28.56386	-19.529	48.09286	20.09524
3049.273	4.063492	9.502195	0.20322	9.824579	31.45443	-15.3037	46.75814	21.94865
2720.62	3.924242	9.211519	0.193151	10.20257	30.18943	-17.5013	47.69071	20.48316
3047.869	3.56338	9.76008	0.198343	10.34188	30.90171	-18.3063	49.208	11.8681
2623.688	3.380282	10.20546	0.207272	10.0447	31.07271	-18.1644	49.23714	20.00774
2463.622	3.150685	9.611519	0.200193	10.07934	27.71114	-20.3001	48.01129	18.82339
2992.128	3.380282	10.1615	0.200691	10.28589	30.84043	-19.7921	50.63257	11.59315
3079.435	3.342466	9.668865	0.194996	10.36921	31.41586	-18.1691	49.585	23.25553
2774.309	3.521739	9.703014	0.202362	9.963909	30.53629	-17.4124	47.94871	20.75152
2940.962	3.907692	9.768635	0.205861	9.845118	31.60629	-15.8463	47.45257	21.61192
3080.584	4.131148	9.272258	0.191442	10.11107	32.01786	-16.416	48.43386	23.07747
3345.531	3.791045	9.12561	0.193741	9.919523	32.98914	-14.113	47.10214	13.41591
2774.309	3.521739	9.703014	0.202362	9.963909	30.53629	-17.4124	47.94871	20.75152
2718.073	3.12	9.238385	0.192095	10.2745	28.56386	-19.529	48.09286	20.09524
3036.521	4.098361	9.444434	0.194661	10.04042	31.90829	-16.609	48.51729	21.97524
3063.674	3.892308	9.501245	0.202792	9.763144	31.00114	-15.851	46.85214	21.86049
3169.654	3.619718	9.142538	0.196486	9.755498	31.73257	-14.7977	46.53029	12.84766
2940.962	3.907692	9.768635	0.205861	9.845118	31.60629	-15.8463	47.45257	21.61192
3043.4	4.666667	8.338459	0.183626	9.777416	30.71686	-14.6931	45.41	21.82645
2997.424	3.865672	9.59628	0.200211	9.834272	31.40686	-16.524	47.93086	21.7804
2720.62	3.924242	9.211519	0.193151	10.20257	30.18943	-17.5013	47.69071	20.48316
2637.797	3.647059	10.26389	0.209853	10.07175	30.58243	-18.3276	48.91	20.10075
2206.389	3.434211	9.759786	0.202076	10.26453	27.81157	-20.4861	48.29771	18.98945
3039.765	4.16129	9.341959	0.20026	9.829737	31.31429	-15.3349	46.64914	21.90146
2951.18	3.053333	10.2714	0.201403	10.33048	30.78543	-20.2139	50.99929	21.25676
2617.909	3.608696	10.25447	0.209609	10.15016	30.47357	-18.4484	48.922	21.03759
3041.622	3.066667	10.70262	0.210772	10.22507	30.71586	-20.0623	50.77814	19.18052
3016.139	3.478873	9.383418	0.190313	10.37424	30.54371	-18.7614	49.30514	21.4313

Table S2 - Anonymized database

2720.62	3.924242	9.211519	0.193151	10.20257	30.18943	-17.5013	47.69071	20.48316
3339.664	4.174603	9.129423	0.19655	9.850078	32.232	-14.2164	46.44843	13.57277
3096.618	4.063492	9.568456	0.202182	9.796947	31.35657	-15.9694	47.326	22.03666
3080.584	4.131148	9.272258	0.191442	10.11107	32.01786	-16.416	48.43386	23.07747
2578.469	3.108108	8.448527	0.179642	10.28123	27.71686	-19.313	47.02986	19.43304
3146.277	4.163934	8.829821	0.191257	9.872304	31.41214	-14.7553	46.16743	22.35059
3043.4	4.666667	8.338459	0.183626	9.777416	30.71686	-14.6931	45.41	21.82645
2554.498	3.731343	10.29718	0.213109	10.02711	30.34971	-17.9691	48.31886	19.85525
3043.4	4.666667	8.338459	0.183626	9.777416	30.71686	-14.6931	45.41	21.82645
2971.976	3.507042	9.839538	0.196129	10.30251	30.57086	-19.5979	50.16871	11.47576
2751.918	3.746269	10.9468	0.226119	9.881999	31.41943	-16.9921	48.41157	20.7932
2955.56	4.147541	9.553184	0.195655	10.03982	31.40329	-17.4234	48.82671	21.63962
2816.375	4.225806	10.11058	0.201421	10.25443	31.65743	-18.5389	50.19629	21.09371
2715.243	3.597222	10.52134	0.209668	10.40458	29.67014	-20.5107	50.18086	13.72431
2774.309	3.521739	9.703014	0.202362	9.963909	30.53629	-17.4124	47.94871	20.75152
2550.962	3.486111	9.757453	0.199574	10.23631	30.00514	-18.8861	48.89129	20.20426
3036.646	4.245902	9.330536	0.193925	10.15762	31.97314	-16.141	48.11414	22.95326
2786.842	3.459459	10.20735	0.2062	10.50743	30.02157	-19.4807	49.50229	13.98523
2593.717	3.441176	9.290489	0.195748	10.15841	28.16857	-19.2929	47.46143	19.6807
3146.277	4.163934	8.829821	0.191257	9.872304	31.41214	-14.7553	46.16743	22.35059
3101.577	4.0625	9.365044	0.20081	9.821972	31.23814	-15.3983	46.63643	22.05755
3043.4	4.666667	8.338459	0.183626	9.777416	30.71686	-14.6931	45.41	21.82645
3036.646	4.245902	9.330536	0.193925	10.15762	31.97314	-16.141	48.11414	22.95326
3124.07	3.848485	9.289995	0.197856	9.90706	31.66843	-15.2849	46.95329	22.29269
3143.601	3.848485	9.72933	0.204933	9.861557	32.02443	-15.4513	47.47571	23.28599
2438.76	3.666667	10.27292	0.207676	10.36404	29.74414	-19.722	49.46614	20.88198
3011.265	4.078125	10.22993	0.208462	9.95724	31.96543	-17.1079	49.07329	22.03953
2719.538	3.80597	10.14394	0.204419	10.16886	31.16057	-18.4626	49.62314	20.62244
2833.384	3.666667	10.54802	0.218217	10.18309	31.266	-17.0713	48.33729	21.86225
3109.165	4.092308	9.821316	0.205086	9.853863	31.83357	-16.0553	47.88886	23.22146
2484.856	3.637681	10.41304	0.208482	10.37992	29.147	-20.8	49.947	19.61129
3025.46	4.03125	9.863706	0.207216	9.835521	31.501	-16.1	47.601	22.77145
3052.366	4.129032	9.698566	0.200726	10.05568	31.85843	-16.459	48.31743	22.07512
2626.119	3.089744	9.693341	0.202767	10.41368	29.196	-18.6094	47.80543	13.31518
2844.379	3.457143	9.777613	0.196765	10.34554	29.874	-19.8179	49.69186	17.44609
2206.389	3.434211	9.759786	0.202076	10.26453	27.81157	-20.4861	48.29771	18.98945
3080.584	4.131148	9.272258	0.191442	10.11107	32.01786	-16.416	48.43386	23.07747
2463.622	3.150685	9.611519	0.200193	10.07934	27.71114	-20.3001	48.01129	18.82339
2513.247	3.088608	9.5855	0.203461	10.44502	28.597	-18.5153	47.11229	13.03295
2593.717	3.441176	9.290489	0.195748	10.15841	28.16857	-19.2929	47.46143	19.6807
2598.688	3.438356	9.616374	0.201647	10.44363	29.066	-18.6231	47.68914	13.24516
3079.435	3.342466	9.668865	0.194996	10.36921	31.41586	-18.1691	49.585	23.25553
2992.128	3.380282	10.1615	0.200691	10.28589	30.84043	-19.7921	50.63257	11.59315
2617.909	3.608696	10.25447	0.209609	10.15016	30.47357	-18.4484	48.922	21.03759
2971.976	3.507042	9.839538	0.196129	10.30251	30.57086	-19.5979	50.16871	11.47576
2720.62	3.924242	9.211519	0.193151	10.20257	30.18943	-17.5013	47.69071	20.48316
2961.49	3.833333	9.770547	0.203823	9.890176	31.73357	-16.2029	47.93643	21.72685
2720.62	3.924242	9.211519	0.193151	10.20257	30.18943	-17.5013	47.69071	20.48316
3011.265	4.078125	10.22993	0.208462	9.95724	31.96543	-17.1079	49.07329	22.03953
2206.389	3.434211	9.759786	0.202076	10.26453	27.81157	-20.4861	48.29771	18.98945

Table S2 - Anonymized database

3146.277	4.163934	8.829821	0.191257	9.872304	31.41214	-14.7553	46.16743	22.35059
2729.098	3.833333	10.26425	0.20757	10.15239	31.23957	-18.2101	49.44971	20.62871
2844.379	3.457143	9.777613	0.196765	10.34554	29.874	-19.8179	49.69186	17.44609
3047.869	3.56338	9.76008	0.198343	10.34188	30.90171	-18.3063	49.208	11.8681
2560.472	3.720588	8.935824	0.184992	10.23391	28.98629	-19.3176	48.30386	19.71297
2720.62	3.924242	9.211519	0.193151	10.20257	30.18943	-17.5013	47.69071	20.48316
3052.728	4.09375	10.09171	0.206352	9.916827	32.14229	-16.7631	48.90543	22.94353
3036.646	4.245902	9.330536	0.193925	10.15762	31.97314	-16.141	48.11414	22.95326
3158.928	4.125	9.289349	0.195812	9.996184	31.92857	-15.5116	47.44014	22.46529
3067.424	3.80597	9.968374	0.208515	9.825926	31.84486	-15.9617	47.80657	22.07466
2513.247	3.088608	9.5855	0.203461	10.44502	28.597	-18.5153	47.11229	13.03295
2513.247	3.088608	9.5855	0.203461	10.44502	28.597	-18.5153	47.11229	13.03295
2662.224	3.742424	9.530261	0.19745	10.28178	29.99014	-18.2766	48.26671	20.23103
2720.62	3.924242	9.211519	0.193151	10.20257	30.18943	-17.5013	47.69071	20.48316
2729.098	3.833333	10.26425	0.20757	10.15239	31.23957	-18.2101	49.44971	20.62871
2689.903	3.217949	9.952698	0.20402	10.44442	29.31957	-19.4633	48.78286	13.32329
3079.435	3.342466	9.668865	0.194996	10.36921	31.41586	-18.1691	49.585	23.25553
2818.002	3.118421	10.37036	0.198005	10.45229	30.28257	-22.0917	52.37429	22.04617
2513.247	3.088608	9.5855	0.203461	10.44502	28.597	-18.5153	47.11229	13.03295
2206.389	3.434211	9.759786	0.202076	10.26453	27.81157	-20.4861	48.29771	18.98945
2979.006	3.514286	9.7355	0.197383	10.40052	30.39414	-18.9287	49.32286	22.82912
2719.538	3.80597	10.14394	0.204419	10.16886	31.16057	-18.4626	49.62314	20.62244
3355.584	3.823529	8.960777	0.194525	9.82302	32.09971	-13.9653	46.065	23.33846
3157.675	4.131148	9.31247	0.192054	10.1149	32.12743	-16.3613	48.48871	22.48246
2334.449	3.444444	9.741253	0.198649	10.44967	28.95714	-20.0804	49.03757	20.41729
3009.203	3.628571	9.71153	0.197405	10.3599	30.71229	-18.4837	49.196	11.6938
3041.622	3.066667	10.70262	0.210772	10.22507	30.71586	-20.0623	50.77814	19.18052
2926.277	3.8	10.21629	0.214888	10.05581	30.92871	-16.6137	47.54243	21.35853
3025.46	4.03125	9.863706	0.207216	9.835521	31.501	-16.1	47.601	22.77145
2818.002	3.118421	10.37036	0.198005	10.45229	30.28257	-22.0917	52.37429	22.04617
3089.078	3.850746	10.15646	0.208547	9.921092	32.163	-16.5381	48.70114	23.13301
2969.13	3.876923	9.648569	0.198923	10.03029	31.83686	-16.6673	48.50414	21.68879
3016.139	3.478873	9.383418	0.190313	10.37424	30.54371	-18.7614	49.30514	21.4313
3345.531	3.791045	9.12561	0.193741	9.919523	32.98914	-14.113	47.10214	13.41591
2560.472	3.720588	8.935824	0.184992	10.23391	28.98629	-19.3176	48.30386	19.71297
3036.521	4.098361	9.444434	0.194661	10.04042	31.90829	-16.609	48.51729	21.97524
2438.76	3.666667	10.27292	0.207676	10.36404	29.74414	-19.722	49.46614	20.88198
2626.119	3.089744	9.693341	0.202767	10.41368	29.196	-18.6094	47.80543	13.31518
2345.926	3.266667	9.0665	0.186425	10.52026	28.78571	-19.8477	48.63343	20.50251
2513.247	3.088608	9.5855	0.203461	10.44502	28.597	-18.5153	47.11229	13.03295
2940.962	3.907692	9.768635	0.205861	9.845118	31.60629	-15.8463	47.45257	21.61192
3146.277	4.163934	8.829821	0.191257	9.872304	31.41214	-14.7553	46.16743	22.35059
2355.08	3.507042	11.32059	0.223415	10.31362	29.91371	-20.7569	50.67057	18.80301
3047.869	3.56338	9.76008	0.198343	10.34188	30.90171	-18.3063	49.208	11.8681
2951.18	3.053333	10.2714	0.201403	10.33048	30.78543	-20.2139	50.99929	21.25676
3071.319	3.118421	10.67653	0.210396	10.22518	31.22514	-19.5197	50.74486	19.21593
3063.674	3.892308	9.501245	0.202792	9.763144	31.00114	-15.851	46.85214	21.86049
3160.261	4.109375	9.709338	0.204196	9.885808	32.01771	-15.5313	47.549	21.68932
2637.797	3.647059	10.26389	0.209853	10.07175	30.58243	-18.3276	48.91	20.10075
3088.305	4.35	9.927816	0.204093	10.03794	32.08886	-16.5547	48.64357	22.1844

Table S2 - Anonymized database

3161.414	3.818182	9.904945	0.208156	9.826251	32.09357	-15.4906	47.58414	23.34493
2940.962	3.907692	9.768635	0.205861	9.845118	31.60629	-15.8463	47.45257	21.61192
2578.469	3.108108	8.448527	0.179642	10.28123	27.71686	-19.313	47.02986	19.43304
3009.203	3.628571	9.71153	0.197405	10.3599	30.71229	-18.4837	49.196	11.6938
3011.265	4.078125	10.22993	0.208462	9.95724	31.96543	-17.1079	49.07329	22.03953
3047.869	3.56338	9.76008	0.198343	10.34188	30.90171	-18.3063	49.208	11.8681
2940.962	3.907692	9.768635	0.205861	9.845118	31.60629	-15.8463	47.45257	21.61192
2987.64	3.585714	9.621104	0.194823	10.3977	30.63357	-18.7501	49.38371	22.88703
2513.247	3.088608	9.5855	0.203461	10.44502	28.597	-18.5153	47.11229	13.03295
2593.717	3.441176	9.290489	0.195748	10.15841	28.16857	-19.2929	47.46143	19.6807
2997.424	3.865672	9.59628	0.200211	9.834272	31.40686	-16.524	47.93086	21.7804
3036.646	4.245902	9.330536	0.193925	10.15762	31.97314	-16.141	48.11414	22.95326
2396.535	3.337838	10.65104	0.212531	10.44231	28.519	-21.5961	50.11514	19.19645
2438.76	3.666667	10.27292	0.207676	10.36404	29.74414	-19.722	49.46614	20.88198
2833.384	3.666667	10.54802	0.218217	10.18309	31.266	-17.0713	48.33729	21.86225
3036.521	4.098361	9.444434	0.194661	10.04042	31.90829	-16.609	48.51729	21.97524
2939.377	3.541667	9.598382	0.194586	10.42902	30.412	-18.9151	49.32714	11.47931
3345.531	3.791045	9.12561	0.193741	9.919523	32.98914	-14.113	47.10214	13.41591
2550.962	3.486111	9.757453	0.199574	10.23631	30.00514	-18.8861	48.89129	20.20426
2345.926	3.266667	9.0665	0.186425	10.52026	28.78571	-19.8477	48.63343	20.50251
2729.098	3.833333	10.26425	0.20757	10.15239	31.23957	-18.2101	49.44971	20.62871
3016.139	3.478873	9.383418	0.190313	10.37424	30.54371	-18.7614	49.30514	21.4313
3036.521	4.098361	9.444434	0.194661	10.04042	31.90829	-16.609	48.51729	21.97524
2610.051	3.213333	9.584712	0.202486	10.53074	29.096	-18.2391	47.33514	15.18559
2786.908	3.492958	9.800266	0.210128	10.2229	30.51757	-16.122	46.63957	22.05495
2720.62	3.924242	9.211519	0.193151	10.20257	30.18943	-17.5013	47.69071	20.48316
2940.962	3.907692	9.768635	0.205861	9.845118	31.60629	-15.8463	47.45257	21.61192
3052.728	4.09375	10.09171	0.206352	9.916827	32.14229	-16.7631	48.90543	22.94353
3067.424	3.80597	9.968374	0.208515	9.825926	31.84486	-15.9617	47.80657	22.07466
3052.366	4.129032	9.698566	0.200726	10.05568	31.85843	-16.459	48.31743	22.07512
2513.247	3.088608	9.5855	0.203461	10.44502	28.597	-18.5153	47.11229	13.03295
2345.926	3.266667	9.0665	0.186425	10.52026	28.78571	-19.8477	48.63343	20.50251
2969.13	3.876923	9.648569	0.198923	10.03029	31.83686	-16.6673	48.50414	21.68879
2334.449	3.444444	9.741253	0.198649	10.44967	28.95714	-20.0804	49.03757	20.41729
2355.08	3.507042	11.32059	0.223415	10.31362	29.91371	-20.7569	50.67057	18.80301
3123.806	4.09375	9.686827	0.205102	9.774654	31.555	-15.6743	47.22929	23.08931
3080.584	4.131148	9.272258	0.191442	10.11107	32.01786	-16.416	48.43386	23.07747
3173.402	3.921875	9.196533	0.198058	9.808554	31.55871	-14.8749	46.43357	22.51121
2940.962	3.907692	9.768635	0.205861	9.845118	31.60629	-15.8463	47.45257	21.61192
2606.346	3.632353	10.4301	0.211153	9.995482	31.43443	-17.9614	49.39586	19.88637
2355.08	3.507042	11.32059	0.223415	10.31362	29.91371	-20.7569	50.67057	18.80301
2206.389	3.434211	9.759786	0.202076	10.26453	27.81157	-20.4861	48.29771	18.98945
2578.469	3.108108	8.448527	0.179642	10.28123	27.71686	-19.313	47.02986	19.43304
3345.531	3.791045	9.12561	0.193741	9.919523	32.98914	-14.113	47.10214	13.41591
2484.856	3.637681	10.41304	0.208482	10.37992	29.147	-20.8	49.947	19.61129
3109.165	4.092308	9.821316	0.205086	9.853863	31.83357	-16.0553	47.88886	23.22146
3516.29	3.577465	8.945646	0.195036	9.882489	32.96786	-12.8987	45.86657	14.22615
3345.531	3.791045	9.12561	0.193741	9.919523	32.98914	-14.113	47.10214	13.41591
2751.918	3.746269	10.9468	0.226119	9.881999	31.41943	-16.9921	48.41157	20.7932
3124.07	3.848485	9.289995	0.197856	9.90706	31.66843	-15.2849	46.95329	22.29269

Table S2 - Anonymized database

2955.56	4.147541	9.553184	0.195655	10.03982	31.40329	-17.4234	48.82671	21.63962
2626.119	3.089744	9.693341	0.202767	10.41368	29.196	-18.6094	47.80543	13.31518
2720.62	3.924242	9.211519	0.193151	10.20257	30.18943	-17.5013	47.69071	20.48316
2912.877	3.148649	10.111	0.198425	10.32885	30.51443	-20.4419	50.95629	22.40084
2926.277	3.8	10.21629	0.214888	10.05581	30.92871	-16.6137	47.54243	21.35853
3253.218	4.274194	9.606536	0.202983	9.875097	32.23571	-15.091	47.32671	13.2132
2818.002	3.118421	10.37036	0.198005	10.45229	30.28257	-22.0917	52.37429	22.04617
2951.18	3.053333	10.2714	0.201403	10.33048	30.78543	-20.2139	50.99929	21.25676
3132.089	4.15873	9.632898	0.203246	10.0056	32.07814	-15.3171	47.39529	22.36384
3041.622	3.066667	10.70262	0.210772	10.22507	30.71586	-20.0623	50.77814	19.18052
3007.729	3.923077	9.609654	0.200674	9.936124	32.10071	-15.7863	47.887	21.88538
3132.089	4.15873	9.632898	0.203246	10.0056	32.07814	-15.3171	47.39529	22.36384
2550.962	3.486111	9.757453	0.199574	10.23631	30.00514	-18.8861	48.89129	20.20426
2832.254	3.757576	10.66365	0.212504	10.19024	32.00971	-18.1711	50.18086	21.19691
2718.073	3.12	9.238385	0.192095	10.2745	28.56386	-19.529	48.09286	20.09524
2818.002	3.118421	10.37036	0.198005	10.45229	30.28257	-22.0917	52.37429	22.04617
3009.203	3.628571	9.71153	0.197405	10.3599	30.71229	-18.4837	49.196	11.6938
2990.374	3.9375	10.12846	0.208523	10.01187	32.73057	-15.8417	48.57229	21.84768
3047.869	3.56338	9.76008	0.198343	10.34188	30.90171	-18.3063	49.208	11.8681
3043.4	4.666667	8.338459	0.183626	9.777416	30.71686	-14.6931	45.41	21.82645
3132.089	4.15873	9.632898	0.203246	10.0056	32.07814	-15.3171	47.39529	22.36384
2746.081	3.081081	9.047838	0.191149	10.30719	28.64843	-18.6854	47.33386	10.59751
2912.877	3.148649	10.111	0.198425	10.32885	30.51443	-20.4419	50.95629	22.40084
3157.675	4.131148	9.31247	0.192054	10.1149	32.12743	-16.3613	48.48871	22.48246
2578.469	3.108108	8.448527	0.179642	10.28123	27.71686	-19.313	47.02986	19.43304
2844.379	3.457143	9.777613	0.196765	10.34554	29.874	-19.8179	49.69186	17.44609
2617.909	3.608696	10.25447	0.209609	10.15016	30.47357	-18.4484	48.922	21.03759
3345.531	3.791045	9.12561	0.193741	9.919523	32.98914	-14.113	47.10214	13.41591
2774.309	3.521739	9.703014	0.202362	9.963909	30.53629	-17.4124	47.94871	20.75152
3143.601	3.848485	9.72933	0.204933	9.861557	32.02443	-15.4513	47.47571	23.28599

Table S2 - Anonymized database

bioc09	bioc12	bioc16	bioc17	bioc18	bioc19	elevation	slope	solar_rad
9.044802	991.827	354.167	182.75	290.509	238.406	393.3734	0.883189	1757.257
9.044802	991.827	354.167	182.75	290.509	238.406	393.3734	0.883189	1757.257
12.52371	993.896	366.841	158.388	303.399	233.035	326.726	4.709216	1576.021
12.52371	993.896	366.841	158.388	303.399	233.035	326.726	4.709216	1576.021
12.52371	993.896	366.841	158.388	303.399	233.035	326.726	4.709216	1576.021
12.52371	993.896	366.841	158.388	303.399	233.035	326.726	4.709216	1576.021
12.52371	993.896	366.841	158.388	303.399	233.035	326.726	4.709216	1576.021
12.52371	993.896	366.841	158.388	303.399	233.035	326.726	4.709216	1576.021
12.52371	993.896	366.841	158.388	303.399	233.035	326.726	4.709216	1576.021
9.354027	957.175	340.137	181.652	277.985	229.463	288.2634	7.966951	2209.513
13.84265	1240.789	517.558	149.909	474.696	246.821	261.3956	2.788267	1863.578
12.20807	946.878	318.087	174.998	274.794	229.236	281.3162	5.652831	2044.658
12.20807	946.878	318.087	174.998	274.794	229.236	281.3162	5.652831	2044.658
12.20807	946.878	318.087	174.998	274.794	229.236	281.3162	5.652831	2044.658
12.20807	946.878	318.087	174.998	274.794	229.236	281.3162	5.652831	2044.658
0.479769	1017.669	352.698	182.124	285.505	245.116	351.0301	7.706647	1808.782
4.91122	1063.63	371.22	193.627	282.856	269.534	289.6766	7.741191	1246.598
1.375121	1021.311	345.988	189.06	237.048	251.852	342.6181	4.375702	1469.899
1.375121	1021.311	345.988	189.06	237.048	251.852	342.6181	4.375702	1469.899
4.000544	1011.503	351.963	179.538	281.873	241.433	371.2694	8.848426	2283.271
4.000544	1011.503	351.963	179.538	281.873	241.433	371.2694	8.848426	2283.271
4.000544	1011.503	351.963	179.538	281.873	241.433	371.2694	8.848426	2283.271
4.000544	1011.503	351.963	179.538	281.873	241.433	371.2694	8.848426	2283.271
4.000544	1011.503	351.963	179.538	281.873	241.433	371.2694	8.848426	2283.271
4.000544	1011.503	351.963	179.538	281.873	241.433	371.2694	8.848426	2283.271
4.000544	1011.503	351.963	179.538	281.873	241.433	371.2694	8.848426	2283.271
15.12292	998.573	362.943	154.869	227.313	280.001	65.75416	1.331257	1836.56
15.12292	998.573	362.943	154.869	227.313	280.001	65.75416	1.331257	1836.56
7.56167	1085.974	370.378	169.387	288.858	317.829	300.4833	2.304261	1756.669
15.58609	1000.009	361.433	179.124	201.378	253.201	67.61459	3.770963	2000.362
15.58609	1000.009	361.433	179.124	201.378	253.201	67.61459	3.770963	2000.362
15.58609	1000.009	361.433	179.124	201.378	253.201	67.61459	3.770963	2000.362
-0.98983	956.144	346.759	160.414	239.894	218.472	333.6983	3.161077	1300.838
-0.98983	956.144	346.759	160.414	239.894	218.472	333.6983	3.161077	1300.838
14.63104	838.498	327.419	138.423	194.725	283.686	207.2583	1.315532	1774.275
-1.80355	982.029	384.009	170.345	232.275	215.746	464.0417	2.215279	1849.836
-1.80355	982.029	384.009	170.345	232.275	215.746	464.0417	2.215279	1849.836
-1.80355	982.029	384.009	170.345	232.275	215.746	464.0417	2.215279	1849.836
-1.80355	982.029	384.009	170.345	232.275	215.746	464.0417	2.215279	1849.836
-1.80355	982.029	384.009	170.345	232.275	215.746	464.0417	2.215279	1849.836
14.11634	987.686	347.181	135.313	280.523	287.051	278.7108	2.373454	1633.689
15.75438	900.163	331.78	120.801	249.553	271.399	201.7106	1.461046	1938.337
12.51116	1099.962	394.828	165.691	348.036	290.794	302.7306	0.714524	1821.651
12.51116	1099.962	394.828	165.691	348.036	290.794	302.7306	0.714524	1821.651
16.90351	986.811	363.566	161.582	264.788	242.427	353.8546	1.464825	1792.748
16.90351	986.811	363.566	161.582	264.788	242.427	353.8546	1.464825	1792.748
16.90351	986.811	363.566	161.582	264.788	242.427	353.8546	1.464825	1792.748
16.90351	986.811	363.566	161.582	264.788	242.427	353.8546	1.464825	1792.748
16.90351	986.811	363.566	161.582	264.788	242.427	353.8546	1.464825	1792.748

Table S2 - Anonymized database

12.52371	993.896	366.841	158.388	303.399	233.035	324.8262	4.430806	1452.51
12.52371	993.896	366.841	158.388	303.399	233.035	324.8262	4.430806	1452.51
12.52371	993.896	366.841	158.388	303.399	233.035	324.8262	4.430806	1452.51
12.52371	993.896	366.841	158.388	303.399	233.035	324.8262	4.430806	1452.51
12.52371	993.896	366.841	158.388	303.399	233.035	324.8262	4.430806	1452.51
12.52371	993.896	366.841	158.388	303.399	233.035	324.8262	4.430806	1452.51
12.52371	993.896	366.841	158.388	303.399	233.035	324.8262	4.430806	1452.51
14.14814	987.4	387.621	161.22	214.5	325.319	138.9436	4.177484	1768.744
7.11078	1049.088	353.943	146.009	284.948	296.027	202.8772	0.626459	1847.405
7.11078	1049.088	353.943	146.009	284.948	296.027	202.8772	0.626459	1847.405
7.11078	1049.088	353.943	146.009	284.948	296.027	202.8772	0.626459	1847.405
10.92335	976.525	356.957	166.786	307.865	215.414	383.0372	3.69426	1981.62
10.92335	976.525	356.957	166.786	307.865	215.414	383.0372	3.69426	1981.62
10.92335	976.525	356.957	166.786	307.865	215.414	383.0372	3.69426	1981.62
10.92335	976.525	356.957	166.786	307.865	215.414	383.0372	3.69426	1981.62
7.473742	1066.62	398.524	135.652	371.894	273.251	110.2755	2.379869	1729.49
7.473742	1066.62	398.524	135.652	371.894	273.251	110.2755	2.379869	1729.49
4.441451	1255.458	456.667	206.111	380.475	301.287	346.7785	9.205475	1602.376
14.77926	1071.804	368.332	158.335	315.379	308.669	216.4959	3.566722	2008.679
14.77926	1071.804	368.332	158.335	315.379	308.669	216.4959	3.566722	2008.679
8.854786	1061.025	387.175	189.077	311.413	246.257	334.6038	2.363094	1913.333
8.854786	1061.025	387.175	189.077	311.413	246.257	334.6038	2.363094	1913.333
4.12744	1143.212	368.326	233.217	302.355	303.292	332.479	1.43155	1747.347
4.12744	1143.212	368.326	233.217	302.355	303.292	332.479	1.43155	1747.347
4.12744	1143.212	368.326	233.217	302.355	303.292	332.479	1.43155	1747.347
4.12744	1143.212	368.326	233.217	302.355	303.292	332.479	1.43155	1747.347
5.010225	1305.261	546.741	196.548	523.579	271.212	354.8399	6.927933	2376.013
-1.34088	925.463	302.666	166.731	241.107	214.488	502.9605	5.291672	1632.742
-1.34088	925.463	302.666	166.731	241.107	214.488	502.9605	5.291672	1632.742
-1.34088	925.463	302.666	166.731	241.107	214.488	502.9605	5.291672	1632.742
-1.34088	925.463	302.666	166.731	241.107	214.488	502.9605	5.291672	1632.742
-1.34088	925.463	302.666	166.731	241.107	214.488	502.9605	5.291672	1632.742
-1.34088	925.463	302.666	166.731	241.107	214.488	502.9605	5.291672	1632.742
-1.34088	925.463	302.666	166.731	241.107	214.488	502.9605	5.291672	1632.742
-1.34088	925.463	302.666	166.731	241.107	214.488	502.9605	5.291672	1632.742
2.915978	1181.614	463.026	199.043	344.474	266.835	560.2236	2.186021	2000.388
2.915978	1181.614	463.026	199.043	344.474	266.835	560.2236	2.186021	2000.388
-0.1286	1094.69	356.775	194.064	288.347	257.338	427.4386	0.903677	1672.949
-0.1286	1094.69	356.775	194.064	288.347	257.338	427.4386	0.903677	1672.949
15.44095	969.157	366.644	144.347	243.381	276.543	61.45306	4.601897	1495.971
-0.16976	1173.229	429.033	154.596	419.01	170.398	324.8262	4.430806	1452.51
-0.16976	1173.229	429.033	154.596	419.01	170.398	324.8262	4.430806	1452.51
-0.16976	1173.229	429.033	154.596	419.01	170.398	324.8262	4.430806	1452.51
-0.16976	1173.229	429.033	154.596	419.01	170.398	324.8262	4.430806	1452.51
-0.16976	1173.229	429.033	154.596	419.01	170.398	324.8262	4.430806	1452.51
-0.16976	1173.229	429.033	154.596	419.01	170.398	324.8262	4.430806	1452.51
-0.78801	1101.295	387.341	150.397	374.596	161.97	487.1186	2.469619	1975.047
0.621198	1219.512	468.387	202.814	393.063	202.814	311.9337	7.613102	2064.278
0.621198	1219.512	468.387	202.814	393.063	202.814	311.9337	7.613102	2064.278
0.621198	1219.512	468.387	202.814	393.063	202.814	311.9337	7.613102	2064.278

Table S2 - Anonymized database

0.621198	1219.512	468.387	202.814	393.063	202.814	311.9337	7.613102	2064.278
0.621198	1219.512	468.387	202.814	393.063	202.814	311.9337	7.613102	2064.278
0.621198	1219.512	468.387	202.814	393.063	202.814	311.9337	7.613102	2064.278
0.621198	1219.512	468.387	202.814	393.063	202.814	311.9337	7.613102	2064.278
0.771571	1197.382	458.126	182.4	415.096	182.4	326.7982	10.80945	1379.297
0.771571	1197.382	458.126	182.4	415.096	182.4	326.7982	10.80945	1379.297
0.771571	1197.382	458.126	182.4	415.096	182.4	326.7982	10.80945	1379.297
-0.17186	1074.032	360.456	138.771	343.129	138.771	175.2454	2.117011	1948.305
-0.17186	1074.032	360.456	138.771	343.129	138.771	175.2454	2.117011	1948.305
18.51983	1182.28	468.97	149.281	389.263	228.039	311.9507	4.48972	1438.063
18.51983	1182.28	468.97	149.281	389.263	228.039	311.9507	4.48972	1438.063
18.51983	1182.28	468.97	149.281	389.263	228.039	311.9507	4.48972	1438.063
0.851703	1355.105	492.748	223.249	415.345	223.249	344.9812	0.403961	1862.702
0.851703	1355.105	492.748	223.249	415.345	223.249	344.9812	0.403961	1862.702
0.437352	1192.216	424.53	193.203	358.705	193.203	346.348	3.354737	2021.434
0.437352	1192.216	424.53	193.203	358.705	193.203	346.348	3.354737	2021.434
0.437352	1192.216	424.53	193.203	358.705	193.203	346.348	3.354737	2021.434
19.98208	1282.251	428.942	199.648	282.135	301.821	261.3956	2.788267	1863.578
19.98208	1282.251	428.942	199.648	282.135	301.821	261.3956	2.788267	1863.578
1.222846	1081.288	481.4	110.162	440.515	110.162	138.7071	2.50683	1815.574
1.222846	1081.288	481.4	110.162	440.515	110.162	138.7071	2.50683	1815.574
1.222846	1081.288	481.4	110.162	440.515	110.162	138.7071	2.50683	1815.574
1.222846	1081.288	481.4	110.162	440.515	110.162	138.7071	2.50683	1815.574
1.222846	1081.288	481.4	110.162	440.515	110.162	138.7071	2.50683	1815.574
1.222846	1081.288	481.4	110.162	440.515	110.162	138.7071	2.50683	1815.574
1.222846	1081.288	481.4	110.162	440.515	110.162	138.7071	2.50683	1815.574
-1.68034	1121.098	416.359	203.313	357.376	203.313	444.7566	3.02755	1895.277
18.31974	1205.787	450.19	167.266	347.842	213.155	342.0194	5.998885	1432.308
1.34567	1036.298	416.576	125.763	351.163	125.763	98.39349	4.764332	1521.834
1.34567	1036.298	416.576	125.763	351.163	125.763	98.39349	4.764332	1521.834
1.34567	1036.298	416.576	125.763	351.163	125.763	98.39349	4.764332	1521.834
1.34567	1036.298	416.576	125.763	351.163	125.763	98.39349	4.764332	1521.834
1.34567	1036.298	416.576	125.763	351.163	125.763	98.39349	4.764332	1521.834
1.620286	1046.792	412.833	141.234	346.007	141.234	82.30245	4.124621	1880.85
1.620286	1046.792	412.833	141.234	346.007	141.234	82.30245	4.124621	1880.85
0.520489	1214.764	431.146	202.207	358.102	202.207	314.7635	8.712111	2402.854
0.520489	1214.764	431.146	202.207	358.102	202.207	314.7635	8.712111	2402.854
0.520489	1214.764	431.146	202.207	358.102	202.207	314.7635	8.712111	2402.854
-0.31168	1068.237	390.363	151.73	337.921	151.73	349.5407	3.111368	1662.415
10.40388	1050.159	366.522	140.553	358.447	160.446	257.2712	12.69096	821.2842
0.816011	1260.301	478.654	184.986	452.367	184.986	311.8525	9.304274	2253.092
1.28983	1198.578	501.065	158.017	491.325	158.017	83.46487	4.532743	1482.57
18.06966	1187.626	460.407	120.673	370.06	252.737	353.7827	1.677322	1845.688
18.06966	1187.626	460.407	120.673	370.06	252.737	353.7827	1.677322	1845.688
18.06966	1187.626	460.407	120.673	370.06	252.737	353.7827	1.677322	1845.688
18.06966	1187.626	460.407	120.673	370.06	252.737	353.7827	1.677322	1845.688
18.06966	1187.626	460.407	120.673	370.06	252.737	353.7827	1.677322	1845.688
1.193769	1313.203	540.484	182.942	510.422	182.942	353.818	9.800788	2414.645
1.584154	1113.703	482.577	139.954	429.346	139.954	256.7606	3.657492	1909.23
1.584154	1113.703	482.577	139.954	429.346	139.954	256.7606	3.657492	1909.23

Table S2 - Anonymized database

1.14756	1229.533	480.647	158.43	449.146	158.43	320.661	3.05985	1967.903
0.278016	1231.077	450.28	214.251	388.43	214.251	350.8546	4.056374	2002.568
1.379049	1054.364	415.073	148.135	389.473	148.135	106.7864	2.553069	1817.755
1.379049	1054.364	415.073	148.135	389.473	148.135	106.7864	2.553069	1817.755
0.715363	1169.376	440.42	164.323	432.232	164.323	214.8781	5.50378	1491.958
16.66705	1638.333	620.346	275.441	543.848	283.835	581.6044	5.521491	1480.118
16.66705	1638.333	620.346	275.441	543.848	283.835	581.6044	5.521491	1480.118
16.66705	1638.333	620.346	275.441	543.848	283.835	581.6044	5.521491	1480.118
1.154319	1069.139	395.328	155.942	377.332	155.942	99.84711	3.078826	1649.392
1.154319	1069.139	395.328	155.942	377.332	155.942	99.84711	3.078826	1649.392
17.83516	1263.249	455.287	161.919	339.602	231.219	368.9091	0.768818	1715.272
17.83516	1263.249	455.287	161.919	339.602	231.219	368.9091	0.768818	1715.272
1.288907	1101.384	521.317	112.221	487.129	112.221	134.9252	5.741443	1696.724
8.643956	1438.028	548.298	229.766	513.314	249.796	663.8906	3.372374	2187.516
0.913973	1173.967	450.496	167.028	431.8	167.028	252.7432	7.030299	2220.283
0.913973	1173.967	450.496	167.028	431.8	167.028	252.7432	7.030299	2220.283
0.913973	1173.967	450.496	167.028	431.8	167.028	252.7432	7.030299	2220.283
0.913973	1173.967	450.496	167.028	431.8	167.028	252.7432	7.030299	2220.283
0.913973	1173.967	450.496	167.028	431.8	167.028	252.7432	7.030299	2220.283
0.913973	1173.967	450.496	167.028	431.8	167.028	252.7432	7.030299	2220.283
0.913973	1173.967	450.496	167.028	431.8	167.028	252.7432	7.030299	2220.283
0.913973	1173.967	450.496	167.028	431.8	167.028	252.7432	7.030299	2220.283
1.197742	1080.637	418.909	149.214	375.353	149.214	220.2656	6.243832	2323.249
1.689896	977.137	384.824	146.945	333.587	146.945	106.5691	4.151762	1547.465
1.689896	977.137	384.824	146.945	333.587	146.945	106.5691	4.151762	1547.465
1.689896	977.137	384.824	146.945	333.587	146.945	106.5691	4.151762	1547.465
1.689896	977.137	384.824	146.945	333.587	146.945	106.5691	4.151762	1547.465
10.41302	1107.858	399.833	144.454	360.669	180.515	442.1107	8.673082	1871.338
10.41302	1107.858	399.833	144.454	360.669	180.515	442.1107	8.673082	1871.338
10.41302	1107.858	399.833	144.454	360.669	180.515	442.1107	8.673082	1871.338
10.41302	1107.858	399.833	144.454	360.669	180.515	442.1107	8.673082	1871.338
17.96497	1217.182	430.052	155.312	302.152	227.337	330.8731	0.217345	1789.407
17.96497	1217.182	430.052	155.312	302.152	227.337	330.8731	0.217345	1789.407
1.542247	1064.831	411.141	151.292	388.665	151.292	75.6599	0.628604	1800.512
1.542247	1064.831	411.141	151.292	388.665	151.292	75.6599	0.628604	1800.512
1.601962	1072.172	436.875	137.997	384.836	137.997	218.33	14.04217	963.5971
1.601962	1072.172	436.875	137.997	384.836	137.997	218.33	14.04217	963.5971
1.601962	1072.172	436.875	137.997	384.836	137.997	218.33	14.04217	963.5971
1.601962	1072.172	436.875	137.997	384.836	137.997	218.33	14.04217	963.5971
-1.4519	1086.461	453.6	123.568	373.129	150.863	340.6455	9.196588	2278.729
-1.4519	1086.461	453.6	123.568	373.129	150.863	340.6455	9.196588	2278.729
-1.4519	1086.461	453.6	123.568	373.129	150.863	340.6455	9.196588	2278.729
0.520451	1256.469	464.044	198.199	401.5	198.199	341.0001	4.046354	2112.621
0.082527	1290.431	569.334	129.365	564.797	129.365	222.9399	2.51953	2007.912
0.082527	1290.431	569.334	129.365	564.797	129.365	222.9399	2.51953	2007.912
0.082527	1290.431	569.334	129.365	564.797	129.365	222.9399	2.51953	2007.912
0.082527	1290.431	569.334	129.365	564.797	129.365	222.9399	2.51953	2007.912
10.15296	1559.359	468.632	281.171	378.371	345.994	358.2456	1.694529	1918.018
12.0029	1615.592	586.466	280.469	478.007	402.27	118.0156	12.89384	1712.243
3.334753	1820.554	648.36	310.079	606.007	375.933	69.08638	7.388803	1751.463

Table S2 - Anonymized database

3.674995	1796.955	636.808	315.92	562.572	388.759	51.4521	2.942134	1946.844
20.29861	1505.293	539.816	263.28	415.787	304.279	381.3399	16.77052	2831.876
11.25932	1786.514	679.295	296.975	564.399	398.125	181.7777	3.736843	1920.678
3.453907	1686.738	545.082	331.226	437.584	402.128	119.4134	0.113889	1846.314
4.866038	1797.357	640.779	337.3	454.125	442.061	34.27692	1.662563	1977.799
8.429346	1519.842	463.864	279.723	375.305	336.653	365.6075	12.60653	1907.473
8.484687	1566.905	478.142	281.648	399.884	348.375	344.1273	8.791059	2300.894
-0.31727	1895.87	656.12	306.828	585.782	315.603	362.4643	6.86713	1628.494
-0.31727	1895.87	656.12	306.828	585.782	315.603	362.4643	6.86713	1628.494
2.233962	1737.543	673.737	283.023	600.086	358.252	270.7938	28.19601	3349.484
2.233962	1737.543	673.737	283.023	600.086	358.252	270.7938	28.19601	3349.484
10.12959	1638.506	522.081	270.148	522.081	357.382	313.4054	11.75299	1418.11
-0.1882	1614.638	664.352	245.667	619.388	295.482	380.8375	4.070759	1519.568
2.621714	1723.602	662.333	291.47	629.761	353.159	208.766	6.265687	1832.687
2.621714	1723.602	662.333	291.47	629.761	353.159	208.766	6.265687	1832.687
7.75183	1744.123	686.263	290.613	624.057	366.899	96.70383	4.908966	1559.2
2.793099	1680.525	640.494	253.498	580.599	350.649	214.722	3.836843	1629.528
3.38656	1671.116	534.958	296.157	388.283	341.02	305.1122	1.098116	1929.252
3.702681	1741.133	640.872	271.713	560.119	436.985	100.9892	8.350395	2369.271
0.446736	2007.881	732.491	340.365	659.08	457.163	311.8601	0.561861	1807.235
2.98006	1712.541	647.985	294.939	584.4	364.177	122.264	6.164512	2312.941
3.170423	1667.742	687.202	246.772	575.913	321.377	125.6447	1.431671	1880.36
12.67234	1722.314	565.458	330.413	405.641	440.329	54.76521	5.173323	1836.214
11.38651	1863.632	727.8	297.391	628.303	411.921	119.6027	1.839919	1925.151
3.202247	1743.849	671.584	293.122	576.912	397.75	146.1161	0.880539	1819.605
3.204835	1834.523	698.969	287.86	616.796	385.032	106.5939	5.842232	1397.98
3.204835	1834.523	698.969	287.86	616.796	385.032	106.5939	5.842232	1397.98
-2.55621	1415.638	491.994	259.568	371.713	269.072	398.2895	1.172895	1606.425
3.186462	1749.97	683.881	258.535	628.119	343.95	174.5617	0.468577	1840.082
3.186462	1749.97	683.881	258.535	628.119	343.95	174.5617	0.468577	1840.082
-2.46074	1373.009	422.337	249.857	365.846	249.857	396.1142	1.802708	1720.296
-2.46074	1373.009	422.337	249.857	365.846	249.857	396.1142	1.802708	1720.296
-1.24902	1802.949	615.544	299.283	536.595	299.283	367.5201	5.641701	2122.695
4.306681	1669.886	546.443	290.398	461.013	447.037	28.46955	0.679182	1846.094
3.654016	1808.103	609.406	326.03	600.122	405.004	69.53417	3.856232	1601.942
1.36717	1631.658	686.213	240.917	561.508	319.077	161.0517	3.782925	1557.11
2.728368	1578.184	496.347	288.66	353.428	329.864	410.7733	5.040017	1900.799
2.728368	1578.184	496.347	288.66	353.428	329.864	410.7733	5.040017	1900.799
2.728368	1578.184	496.347	288.66	353.428	329.864	410.7733	5.040017	1900.799
2.728368	1578.184	496.347	288.66	353.428	329.864	410.7733	5.040017	1900.799
0.537049	1707.55	650.433	286.152	619.503	305.673	365.2663	8.606683	2248.623
0.537049	1707.55	650.433	286.152	619.503	305.673	365.2663	8.606683	2248.623
2.99339	1736.133	670.84	291.367	566.046	393.127	178.8689	3.350492	1871.85
3.09322	1645.044	511.449	294.596	395.47	329.005	311.3914	3.386594	1645.39
-1.55404	1655.204	707.488	235.803	692.561	285.481	489.7062	3.59755	1702.217
10.09323	1722.892	566.342	274.754	566.342	353.188	272.5588	7.12005	2301.675
10.09323	1722.892	566.342	274.754	566.342	353.188	272.5588	7.12005	2301.675
-0.41771	1550.417	578.046	232.753	534.165	284.639	200.8394	0.870624	920.6934
9.714725	2126.397	975.95	282.783	975.95	390.961	197.695	5.642335	2223.441
9.714725	2126.397	975.95	282.783	975.95	390.961	197.695	5.642335	2223.441

Table S2 - Anonymized database

9.714725	2126.397	975.95	282.783	975.95	390.961	197.695	5.642335	2223.441
-2.13416	1758.907	557.63	287.94	533.806	287.94	578.7245	15.7686	1843.488
0.940978	1742.635	716.934	292.411	568.432	345.087	306.2375	0.923596	1703.135
1.108027	1624.28	625.868	268.403	625.868	274.827	369.4496	3.147714	1759.718
1.108027	1624.28	625.868	268.403	625.868	274.827	369.4496	3.147714	1759.718
1.108027	1624.28	625.868	268.403	625.868	274.827	369.4496	3.147714	1759.718
3.361176	1757.985	700.142	264.277	591.576	417.158	130.5054	1.396612	1963.494
11.64161	1855.697	761.546	284.142	679.693	395.206	119.5475	4.079675	1512.646
11.64161	1855.697	761.546	284.142	679.693	395.206	119.5475	4.079675	1512.646
11.64161	1855.697	761.546	284.142	679.693	395.206	119.5475	4.079675	1512.646
1.068066	1644.372	676.736	255.604	540.952	328.193	187.6075	5.691956	1343.706
1.068066	1644.372	676.736	255.604	540.952	328.193	187.6075	5.691956	1343.706
1.495863	1907.282	690.445	334.487	517.025	436.375	148.2937	1.099489	1699.062
3.165027	1786.812	722.962	244.536	616.051	363.824	176.3364	1.888108	1919.367
3.165027	1786.812	722.962	244.536	616.051	363.824	176.3364	1.888108	1919.367
2.045709	1716.124	701.845	242.792	614.784	306.751	288.6531	13.5198	1038.656
2.045709	1716.124	701.845	242.792	614.784	306.751	288.6531	13.5198	1038.656
1.517253	1619.106	647.824	248.946	647.824	267.039	330.4612	5.314943	2050.618
1.517253	1619.106	647.824	248.946	647.824	267.039	330.4612	5.314943	2050.618
12.39011	1694.763	534.732	335.899	398.526	429.408	83.90097	2.566	2002.437
12.39011	1694.763	534.732	335.899	398.526	429.408	83.90097	2.566	2002.437
12.39011	1694.763	534.732	335.899	398.526	429.408	83.90097	2.566	2002.437
2.923692	1750.604	725.927	281.575	644.04	339.092	108.9301	0.270718	1853.959
2.923692	1750.604	725.927	281.575	644.04	339.092	108.9301	0.270718	1853.959
-2.32548	1444.681	448.344	270.455	395.367	270.455	330.781	0.599844	1782.226
-2.32548	1444.681	448.344	270.455	395.367	270.455	330.781	0.599844	1782.226
2.309527	1759.552	665.196	297.839	608.559	382.53	231.4455	5.869617	2064.344
2.309527	1759.552	665.196	297.839	608.559	382.53	231.4455	5.869617	2064.344
-0.98347	1482.361	509.542	255.363	437.681	289.916	443.7068	24.45277	9.84658
2.658973	1857.823	804.853	282.122	769.688	368.375	163.24	0.627206	1907.193
-2.0658	1737.211	552.653	308.083	492.523	308.083	559.32	6.887537	1658.008
-2.0658	1737.211	552.653	308.083	492.523	308.083	559.32	6.887537	1658.008
2.775	1706.642	622.925	269.626	594.823	367.238	151.6085	3.044361	1695.477
2.775	1706.642	622.925	269.626	594.823	367.238	151.6085	3.044361	1695.477
9.765038	1726.196	579.005	315.421	579.005	318.305	367.2032	9.372241	1854.878
9.765038	1726.196	579.005	315.421	579.005	318.305	367.2032	9.372241	1854.878
9.765038	1726.196	579.005	315.421	579.005	318.305	367.2032	9.372241	1854.878
9.765038	1726.196	579.005	315.421	579.005	318.305	367.2032	9.372241	1854.878
2.92322	1869.494	701.625	331.039	646.369	378.381	118.1115	1.501855	1862.66
2.739203	1760.081	718.746	280.373	654.203	347.181	162.8161	1.964231	1741.297
-0.03574	1728.667	592.558	281.159	459.295	297.488	321.0894	7.302571	1338.034
3.270945	1667.127	534.067	293.901	424.25	335.156	334.5434	9.641643	1425.432
3.270945	1667.127	534.067	293.901	424.25	335.156	334.5434	9.641643	1425.432
2.861005	1876.506	770.783	302.223	712.595	376.309	131.5466	7.098525	1302.395
2.861005	1876.506	770.783	302.223	712.595	376.309	131.5466	7.098525	1302.395
-2.66538	1472.877	466.147	269.666	425.297	269.666	385.9416	16.94996	446.4642
-2.66538	1472.877	466.147	269.666	425.297	269.666	385.9416	16.94996	446.4642
-2.66538	1472.877	466.147	269.666	425.297	269.666	385.9416	16.94996	446.4642
3.254396	1940.243	834.343	252.329	723.289	400.305	297.4856	4.78008	2179.6
3.254396	1940.243	834.343	252.329	723.289	400.305	297.4856	4.78008	2179.6

Table S2 - Anonymized database

-1.80219	1829.322	673.068	270.259	598.688	295.698	425.881	16.28463	2612.849
-1.80219	1829.322	673.068	270.259	598.688	295.698	425.881	16.28463	2612.849
3.191758	1749.745	600.434	315.678	581.92	364.259	71.10771	0.571329	1809.155
3.191758	1749.745	600.434	315.678	581.92	364.259	71.10771	0.571329	1809.155
3.191758	1749.745	600.434	315.678	581.92	364.259	71.10771	0.571329	1809.155
3.191758	1749.745	600.434	315.678	581.92	364.259	71.10771	0.571329	1809.155
3.673863	1689.434	533.232	297.784	533.232	348.849	353.818	9.800788	2414.645
3.03844	1928.127	868.941	263.871	768.761	345.561	115.4449	3.68327	1941.253
3.03844	1928.127	868.941	263.871	768.761	345.561	115.4449	3.68327	1941.253
3.019214	1815.915	658.626	332.458	596.949	373.76	107.388	1.836803	1754.523
3.019214	1815.915	658.626	332.458	596.949	373.76	107.388	1.836803	1754.523
3.019214	1815.915	658.626	332.458	596.949	373.76	107.388	1.836803	1754.523
3.019214	1815.915	658.626	332.458	596.949	373.76	107.388	1.836803	1754.523
11.56054	1731.735	653	289.524	551.408	392.258	133.692	0.928783	1841.605
3.019214	1815.915	658.626	332.458	596.949	373.76	62.67819	6.543175	1760.891
3.019214	1815.915	658.626	332.458	596.949	373.76	62.67819	6.543175	1760.891
7.99656	1601.338	515.562	279.154	513.384	337.386	388.4849	0.546904	1860.972
7.99656	1601.338	515.562	279.154	513.384	337.386	388.4849	0.546904	1860.972
0.693659	1641.955	674.39	234.111	617.163	294.82	211.2159	8.984717	1305.867
0.693659	1641.955	674.39	234.111	617.163	294.82	211.2159	8.984717	1305.867
0.693659	1641.955	674.39	234.111	617.163	294.82	211.2159	8.984717	1305.867
0.057346	1641.624	688.454	233.49	652.575	304.641	423.5338	15.46822	1331.014
-2.73457	1690.79	575.959	263.325	529.174	263.325	602.6755	10.18564	2336.094
3.269582	1761.512	673.087	252.466	580.927	351.081	186.2722	14.29552	2568.046
3.269582	1761.512	673.087	252.466	580.927	351.081	186.2722	14.29552	2568.046
10.22724	1567.866	501.406	274.268	463.839	342.907	344.3909	7.572275	1738.915
10.22724	1567.866	501.406	274.268	463.839	342.907	344.3909	7.572275	1738.915
10.22724	1567.866	501.406	274.268	463.839	342.907	344.3909	7.572275	1738.915
0.655505	1798.683	851.344	229.906	796.802	302.237	230.9364	4.226843	2008.737
0.655505	1798.683	851.344	229.906	796.802	302.237	230.9364	4.226843	2008.737
0.655505	1798.683	851.344	229.906	796.802	302.237	230.9364	4.226843	2008.737
0.655505	1798.683	851.344	229.906	796.802	302.237	230.9364	4.226843	2008.737
12.79818	1775.993	583.92	319.59	490.019	439.791	91.46474	1.612379	1995.601
12.79818	1775.993	583.92	319.59	490.019	439.791	91.46474	1.612379	1995.601
12.79818	1775.993	583.92	319.59	490.019	439.791	91.46474	1.612379	1995.601
7.493527	1732.353	675.805	294.739	618.215	369.921	105.9056	4.341443	1633.646
7.493527	1732.353	675.805	294.739	618.215	369.921	105.9056	4.341443	1633.646
7.493527	1732.353	675.805	294.739	618.215	369.921	105.9056	4.341443	1633.646
3.539126	1680.951	554.388	288.569	403.414	299.121	317.2497	11.81392	959.2725
3.539126	1680.951	554.388	288.569	403.414	299.121	317.2497	11.81392	959.2725
3.539126	1680.951	554.388	288.569	403.414	299.121	317.2497	11.81392	959.2725
10.2139	1949.674	754.498	311.599	738.447	399.291	126.3232	4.08183	1849.596
10.2139	1949.674	754.498	311.599	738.447	399.291	126.3232	4.08183	1849.596
10.11392	1595.562	514.394	277.919	514.394	340.584	327.824	10.42236	2086.291
10.11392	1595.562	514.394	277.919	514.394	340.584	327.824	10.42236	2086.291
10.11392	1595.562	514.394	277.919	514.394	340.584	327.824	10.42236	2086.291
10.11392	1595.562	514.394	277.919	514.394	340.584	327.824	10.42236	2086.291
10.11392	1595.562	514.394	277.919	514.394	340.584	327.824	10.42236	2086.291
1.527967	1593.369	653.504	239.989	652.947	263.645	222.4361	2.733181	1472.043
1.527967	1593.369	653.504	239.989	652.947	263.645	222.4361	2.733181	1472.043

Table S2 - Anonymized database

2.515495	1766.287	753.767	272.141	674.837	333.336	147.7344	2.213931	1994.002
2.515495	1766.287	753.767	272.141	674.837	333.336	147.7344	2.213931	1994.002
2.515495	1766.287	753.767	272.141	674.837	333.336	147.7344	2.213931	1994.002
2.994984	1636.069	511.474	292.264	370.066	332.285	363.2772	6.386463	2184.072
2.994984	1636.069	511.474	292.264	370.066	332.285	363.2772	6.386463	2184.072
2.994984	1636.069	511.474	292.264	370.066	332.285	363.2772	6.386463	2184.072
-0.8633	1752.065	718.955	260.487	570.262	362.782	302.8407	5.463532	1223.193
-0.8633	1752.065	718.955	260.487	570.262	362.782	302.8407	5.463532	1223.193
3.082264	1841.716	765.463	299.75	657.881	389.15	187.7079	2.024981	2018.113
3.082264	1841.716	765.463	299.75	657.881	389.15	187.7079	2.024981	2018.113
3.082264	1841.716	765.463	299.75	657.881	389.15	187.7079	2.024981	2018.113
3.082264	1841.716	765.463	299.75	657.881	389.15	187.7079	2.024981	2018.113
3.082264	1841.716	765.463	299.75	657.881	389.15	187.7079	2.024981	2018.113
3.082264	1841.716	765.463	299.75	657.881	389.15	187.7079	2.024981	2018.113
-0.9291	1498.215	648.835	172.608	624.047	236.998	318.5292	13.84482	1524.968
-0.9291	1498.215	648.835	172.608	624.047	236.998	318.5292	13.84482	1524.968
-0.9291	1498.215	648.835	172.608	624.047	236.998	318.5292	13.84482	1524.968
-0.9291	1498.215	648.835	172.608	624.047	236.998	318.5292	13.84482	1524.968
2.67878	1857.64	776.54	295.668	720.464	376.505	139.6398	0.460311	1805.588
2.67878	1857.64	776.54	295.668	720.464	376.505	139.6398	0.460311	1805.588
2.67878	1857.64	776.54	295.668	720.464	376.505	139.6398	0.460311	1805.588
2.67878	1857.64	776.54	295.668	720.464	376.505	139.6398	0.460311	1805.588
9.674176	1677.047	747.362	233.006	715.846	287.275	500.629	1.819382	1815.314
9.674176	1677.047	747.362	233.006	715.846	287.275	500.629	1.819382	1815.314
9.674176	1677.047	747.362	233.006	715.846	287.275	500.629	1.819382	1815.314
1.165121	1758.55	773.716	260.25	620.897	324.493	240.9818	2.35066	1953.851
1.165121	1758.55	773.716	260.25	620.897	324.493	240.9818	2.35066	1953.851
1.165121	1758.55	773.716	260.25	620.897	324.493	240.9818	2.35066	1953.851
1.165121	1758.55	773.716	260.25	620.897	324.493	240.9818	2.35066	1953.851
1.165121	1758.55	773.716	260.25	620.897	324.493	240.9818	2.35066	1953.851
-3.14471	1338.751	410.37	265.597	404.62	271.411	482.474	3.426512	1754.312
-3.14471	1338.751	410.37	265.597	404.62	271.411	482.474	3.426512	1754.312
-3.14471	1338.751	410.37	265.597	404.62	271.411	482.474	3.426512	1754.312
-3.14471	1338.751	410.37	265.597	404.62	271.411	482.474	3.426512	1754.312
3.284632	1766.656	726.812	251.065	605.233	355.336	142.9463	2.576109	1703.397
3.284632	1766.656	726.812	251.065	605.233	355.336	142.9463	2.576109	1703.397
3.284632	1766.656	726.812	251.065	605.233	355.336	142.9463	2.576109	1703.397
3.284632	1766.656	726.812	251.065	605.233	355.336	142.9463	2.576109	1703.397
10.3962	1656.738	540.026	292.239	499.881	349.794	288.6407	7.765498	2007.697
10.3962	1656.738	540.026	292.239	499.881	349.794	288.6407	7.765498	2007.697
10.3962	1656.738	540.026	292.239	499.881	349.794	288.6407	7.765498	2007.697
3.224808	1725.185	734.304	267.716	605.522	351.814	118.4804	4.569775	1687.026
3.224808	1725.185	734.304	267.716	605.522	351.814	118.4804	4.569775	1687.026
3.224808	1725.185	734.304	267.716	605.522	351.814	118.4804	4.569775	1687.026
2.844868	1735.468	596.5	332.033	537.819	364.987	99.84711	3.078826	1649.392
2.844868	1735.468	596.5	332.033	537.819	364.987	99.84711	3.078826	1649.392
2.844868	1735.468	596.5	332.033	537.819	364.987	99.84711	3.078826	1649.392
-1.72355	1358.596	581.878	172.581	523.931	235.866	693.117	3.449864	1538.647
-1.72355	1358.596	581.878	172.581	523.931	235.866	693.117	3.449864	1538.647
-1.72355	1358.596	581.878	172.581	523.931	235.866	693.117	3.449864	1538.647

Table S2 - Anonymized database

-1.72355	1358.596	581.878	172.581	523.931	235.866	693.117	3.449864	1538.647
9.255143	1650.811	552.766	309.093	552.766	317.585	384.1146	8.195766	2390.127
9.255143	1650.811	552.766	309.093	552.766	317.585	384.1146	8.195766	2390.127
9.255143	1650.811	552.766	309.093	552.766	317.585	384.1146	8.195766	2390.127
9.255143	1650.811	552.766	309.093	552.766	317.585	384.1146	8.195766	2390.127
9.255143	1650.811	552.766	309.093	552.766	317.585	384.1146	8.195766	2390.127
9.255143	1650.811	552.766	309.093	552.766	317.585	384.1146	8.195766	2390.127
1.599203	1874.653	773.233	277.26	704.633	364.567	427.536	23.76717	3358.25
1.599203	1874.653	773.233	277.26	704.633	364.567	427.536	23.76717	3358.25
1.599203	1874.653	773.233	277.26	704.633	364.567	427.536	23.76717	3358.25
1.599203	1874.653	773.233	277.26	704.633	364.567	427.536	23.76717	3358.25
11.32852	1866.229	735.493	293.782	613.172	407.25	250.0854	6.824211	2129.41
11.32852	1866.229	735.493	293.782	613.172	407.25	250.0854	6.824211	2129.41
11.32852	1866.229	735.493	293.782	613.172	407.25	250.0854	6.824211	2129.41
11.32852	1866.229	735.493	293.782	613.172	407.25	250.0854	6.824211	2129.41
11.32852	1866.229	735.493	293.782	613.172	407.25	250.0854	6.824211	2129.41
11.32852	1866.229	735.493	293.782	613.172	407.25	250.0854	6.824211	2129.41
11.32852	1866.229	735.493	293.782	613.172	407.25	250.0854	6.824211	2129.41
11.32852	1866.229	735.493	293.782	613.172	407.25	250.0854	6.824211	2129.41
11.32852	1866.229	735.493	293.782	613.172	407.25	250.0854	6.824211	2129.41
11.32852	1866.229	735.493	293.782	613.172	407.25	250.0854	6.824211	2129.41
3.078527	1709.982	751.458	244.056	643.587	367.179	191.9448	3.036096	2134.077
3.078527	1709.982	751.458	244.056	643.587	367.179	191.9448	3.036096	2134.077
3.078527	1709.982	751.458	244.056	643.587	367.179	191.9448	3.036096	2134.077
3.078527	1709.982	751.458	244.056	643.587	367.179	191.9448	3.036096	2134.077
0.977159	1594.237	664.744	241.42	613.571	280.445	344.4803	2.282428	1975.805
0.977159	1594.237	664.744	241.42	613.571	280.445	344.4803	2.282428	1975.805
0.977159	1594.237	664.744	241.42	613.571	280.445	344.4803	2.282428	1975.805
0.977159	1594.237	664.744	241.42	613.571	280.445	344.4803	2.282428	1975.805
0.977159	1594.237	664.744	241.42	613.571	280.445	344.4803	2.282428	1975.805
0.977159	1594.237	664.744	241.42	613.571	280.445	344.4803	2.282428	1975.805
0.977159	1594.237	664.744	241.42	613.571	280.445	344.4803	2.282428	1975.805
0.977159	1594.237	664.744	241.42	613.571	280.445	344.4803	2.282428	1975.805
0.977159	1594.237	664.744	241.42	613.571	280.445	344.4803	2.282428	1975.805
4.000544	1011.503	351.963	179.538	281.873	241.433	371.2694	8.848426	2283.271
12.52371	993.896	366.841	158.388	303.399	233.035	324.8262	4.430806	1452.51
16.90351	986.811	363.566	161.582	264.788	242.427	353.8546	1.464825	1792.748
14.68604	944.988	330.019	134.217	209.285	286.834	132.9971	1.029028	1796.389
15.58609	1000.009	361.433	179.124	201.378	253.201	67.61459	3.770963	2000.362
12.20807	946.878	318.087	174.998	274.794	229.236	281.3162	5.652831	2044.658
16.90351	986.811	363.566	161.582	264.788	242.427	353.8546	1.464825	1792.748
7.473742	1066.62	398.524	135.652	371.894	273.251	110.2755	2.379869	1729.49
12.52371	993.896	366.841	158.388	303.399	233.035	326.726	4.709216	1576.021
4.91122	1063.63	371.22	193.627	282.856	269.534	289.6766	7.741191	1246.598
-0.98983	956.144	346.759	160.414	239.894	218.472	333.6983	3.161077	1300.838
12.52371	993.896	366.841	158.388	303.399	233.035	326.726	4.709216	1576.021
12.52371	993.896	366.841	158.388	303.399	233.035	326.726	4.709216	1576.021
-1.80355	982.029	384.009	170.345	232.275	215.746	464.0417	2.215279	1849.836
12.51116	1099.962	394.828	165.691	348.036	290.794	302.7306	0.714524	1821.651
13.81095	1034.957	372.781	154.518	273.019	309.967	216.8499	8.778607	2428.288

Table S2 - Anonymized database

12.52371	993.896	366.841	158.388	303.399	233.035	326.726	4.709216	1576.021
9.044802	991.827	354.167	182.75	290.509	238.406	393.3734	0.883189	1757.257
12.52371	993.896	366.841	158.388	303.399	233.035	324.8262	4.430806	1452.51
0.222209	1018.41	362.801	162.232	301.794	226.826	330.1108	1.448045	1837.309
14.25296	913.857	345.469	137.96	189.659	287.484	60.08836	2.996304	1851.407
13.84265	1240.789	517.558	149.909	474.696	246.821	261.3956	2.788267	1863.578
12.20807	946.878	318.087	174.998	274.794	229.236	281.3162	5.652831	2044.658
12.52371	993.896	366.841	158.388	303.399	233.035	324.8262	4.430806	1452.51
12.20807	946.878	318.087	174.998	274.794	229.236	337.5405	3.893426	1645.461
2.915978	1181.614	463.026	199.043	344.474	266.835	560.2236	2.186021	2000.388
2.915978	1181.614	463.026	199.043	344.474	266.835	560.2236	2.186021	2000.388
12.20807	946.878	318.087	174.998	274.794	229.236	281.3162	5.652831	2044.658
8.854786	1061.025	387.175	189.077	311.413	246.257	334.6038	2.363094	1913.333
13.81095	1034.957	372.781	154.518	273.019	309.967	216.8499	8.778607	2428.288
16.90351	986.811	363.566	161.582	264.788	242.427	353.8546	1.464825	1792.748
12.52371	993.896	366.841	158.388	303.399	233.035	326.726	4.709216	1576.021
14.19208	966.958	367.081	135.801	233.848	293.741	126.8244	3.309033	1579.56
6.909423	992.342	340.909	154.232	245.555	285.881	184.0726	2.807042	1659.809
4.441451	1255.458	456.667	206.111	380.475	301.287	346.7785	9.205475	1602.376
7.770912	1003.114	339.764	159.097	249.623	287.605	191.539	8.983807	1815.853
9.044802	991.827	354.167	182.75	290.509	238.406	393.3734	0.883189	1757.257
8.854786	1061.025	387.175	189.077	311.413	246.257	334.6038	2.363094	1913.333
0.498819	1041.527	361.072	186.248	309.375	247.21	344.0219	8.271492	2272.567
4.000544	1011.503	351.963	179.538	281.873	241.433	371.2694	8.848426	2283.271
14.11634	987.686	347.181	135.313	280.523	287.051	278.7108	2.373454	1633.689
14.25296	913.857	345.469	137.96	189.659	287.484	60.08836	2.996304	1851.407
12.52371	993.896	366.841	158.388	303.399	233.035	324.8262	4.430806	1452.51
7.770912	1003.114	339.764	159.097	249.623	287.605	191.539	8.983807	1815.853
4.12744	1143.212	368.326	233.217	302.355	303.292	332.479	1.43155	1747.347
13.84265	1240.789	517.558	149.909	474.696	246.821	261.3956	2.788267	1863.578
12.52371	993.896	366.841	158.388	303.399	233.035	326.726	4.709216	1576.021
12.51116	1099.962	394.828	165.691	348.036	290.794	302.7306	0.714524	1821.651
0.479769	1017.669	352.698	182.124	285.505	245.116	351.0301	7.706647	1808.782
7.49811	1020.529	349.305	144.313	305.725	275.767	129.0689	5.067988	2231.996
15.44095	969.157	366.644	144.347	243.381	276.543	61.45306	4.601897	1495.971
12.51116	1099.962	394.828	165.691	348.036	290.794	302.7306	0.714524	1821.651
1.375121	1021.311	345.988	189.06	237.048	251.852	342.6181	4.375702	1469.899
8.854786	1061.025	387.175	189.077	311.413	246.257	334.6038	2.363094	1913.333
16.90351	986.811	363.566	161.582	264.788	242.427	353.8546	1.464825	1792.748
12.52371	993.896	366.841	158.388	303.399	233.035	324.8262	4.430806	1452.51
14.53205	1109.398	381.222	185.432	255.372	311.253	90.55988	3.017958	1646.286
6.501401	701.054	230.791	121.731	167.889	212.075	192.8504	1.106623	1910.482
4.12744	1143.212	368.326	233.217	302.355	303.292	332.479	1.43155	1747.347
15.44095	969.157	366.644	144.347	243.381	276.543	61.45306	4.601897	1495.971
-1.34088	925.463	302.666	166.731	241.107	214.488	502.9605	5.291672	1632.742
12.52371	993.896	366.841	158.388	303.399	233.035	326.726	4.709216	1576.021
13.87799	934.095	354.856	143.822	199.265	294.851	136.4366	3.643151	1641.586
10.92335	976.525	356.957	166.786	307.865	215.414	383.0372	3.69426	1981.62
-1.80355	982.029	384.009	170.345	232.275	215.746	464.0417	2.215279	1849.836
15.75438	900.163	331.78	120.801	249.553	271.399	201.7106	1.461046	1938.337

Table S2 - Anonymized database

13.81095	1034.957	372.781	154.518	273.019	309.967	216.8499	8.778607	2428.288
7.49811	1020.529	349.305	144.313	305.725	275.767	129.0689	5.067988	2231.996
12.52371	993.896	366.841	158.388	303.399	233.035	326.726	4.709216	1576.021
10.92335	976.525	356.957	166.786	307.865	215.414	383.0372	3.69426	1981.62
16.90351	986.811	363.566	161.582	264.788	242.427	353.8546	1.464825	1792.748
-0.1286	1094.69	356.775	194.064	288.347	257.338	427.4386	0.903677	1672.949
-1.80355	982.029	384.009	170.345	232.275	215.746	464.0417	2.215279	1849.836
12.52371	993.896	366.841	158.388	303.399	233.035	326.726	4.709216	1576.021
12.52371	993.896	366.841	158.388	303.399	233.035	326.726	4.709216	1576.021
0.479769	1017.669	352.698	182.124	285.505	245.116	351.0301	7.706647	1808.782
13.84265	1240.789	517.558	149.909	474.696	246.821	261.3956	2.788267	1863.578
-1.80355	982.029	384.009	170.345	232.275	215.746	464.0417	2.215279	1849.836
14.14814	987.4	387.621	161.22	214.5	325.319	138.9436	4.177484	1768.744
4.12744	1143.212	368.326	233.217	302.355	303.292	332.479	1.43155	1747.347
13.84265	1240.789	517.558	149.909	474.696	246.821	261.3956	2.788267	1863.578
0.479769	1017.669	352.698	182.124	285.505	245.116	351.0301	7.706647	1808.782
14.14814	987.4	387.621	161.22	214.5	325.319	138.9436	4.177484	1768.744
0.479769	1017.669	352.698	182.124	285.505	245.116	351.0301	7.706647	1808.782
12.52371	993.896	366.841	158.388	303.399	233.035	326.726	4.709216	1576.021
-1.34088	925.463	302.666	166.731	241.107	214.488	502.9605	5.291672	1632.742
12.52371	993.896	366.841	158.388	303.399	233.035	324.8262	4.430806	1452.51
-1.80355	982.029	384.009	170.345	232.275	215.746	464.0417	2.215279	1849.836
15.75438	900.163	331.78	120.801	249.553	271.399	201.7106	1.461046	1938.337
4.12744	1143.212	368.326	233.217	302.355	303.292	332.479	1.43155	1747.347
12.52371	993.896	366.841	158.388	303.399	233.035	324.8262	4.430806	1452.51
14.53205	1109.398	381.222	185.432	255.372	311.253	90.55988	3.017958	1646.286
14.63104	838.498	327.419	138.423	194.725	283.686	207.2583	1.315532	1774.275
15.44095	969.157	366.644	144.347	243.381	276.543	61.45306	4.601897	1495.971
-1.80355	982.029	384.009	170.345	232.275	215.746	464.0417	2.215279	1849.836
-1.80355	982.029	384.009	170.345	232.275	215.746	464.0417	2.215279	1849.836
15.75438	900.163	331.78	120.801	249.553	271.399	201.7106	1.461046	1938.337
12.51116	1099.962	394.828	165.691	348.036	290.794	302.7306	0.714524	1821.651
15.12292	998.573	362.943	154.869	227.313	280.001	65.75416	1.331257	1836.56
13.87799	934.095	354.856	143.822	199.265	294.851	136.4366	3.643151	1641.586
-1.80355	982.029	384.009	170.345	232.275	215.746	464.0417	2.215279	1849.836
12.20807	946.878	318.087	174.998	274.794	229.236	337.5405	3.893426	1645.461
14.30245	999.755	345.534	169.656	210.583	290.658	119.721	3.06824	1631.99
-1.34088	925.463	302.666	166.731	241.107	214.488	502.9605	5.291672	1632.742
1.623912	1157.452	440.171	189.349	381.165	251.905	300.6322	3.048275	2061.277
12.51116	1099.962	394.828	165.691	348.036	290.794	302.7306	0.714524	1821.651
-1.80355	982.029	384.009	170.345	232.275	215.746	464.0417	2.215279	1849.836
7.11078	1049.088	353.943	146.009	284.948	296.027	202.8772	0.626459	1847.405
0.479769	1017.669	352.698	182.124	285.505	245.116	351.0301	7.706647	1808.782
12.51116	1099.962	394.828	165.691	348.036	290.794	302.7306	0.714524	1821.651
4.000544	1011.503	351.963	179.538	281.873	241.433	371.2694	8.848426	2283.271
7.11078	1049.088	353.943	146.009	284.948	296.027	202.8772	0.626459	1847.405
14.26695	975.32	347.324	129.172	244.574	273.307	94.82304	0.619173	1813.049
16.90351	986.811	363.566	161.582	264.788	242.427	353.8546	1.464825	1792.748
13.84265	1240.789	517.558	149.909	474.696	246.821	261.3956	2.788267	1863.578
12.20807	946.878	318.087	174.998	274.794	229.236	281.3162	5.652831	2044.658

Table S2 - Anonymized database

14.26695	975.32	347.324	129.172	244.574	273.307	94.82304	0.619173	1813.049
15.75438	900.163	331.78	120.801	249.553	271.399	201.7106	1.461046	1938.337
15.44095	969.157	366.644	144.347	243.381	276.543	61.45306	4.601897	1495.971
-1.34088	925.463	302.666	166.731	241.107	214.488	502.9605	5.291672	1632.742
12.52371	993.896	366.841	158.388	303.399	233.035	326.726	4.709216	1576.021
12.52371	993.896	366.841	158.388	303.399	233.035	326.726	4.709216	1576.021
3.715302	835.73	299.191	144.341	227.131	219.342	371.2189	6.547291	2148.801
12.51116	1099.962	394.828	165.691	348.036	290.794	302.7306	0.714524	1821.651
10.92335	976.525	356.957	166.786	307.865	215.414	383.0372	3.69426	1981.62
9.044802	991.827	354.167	182.75	290.509	238.406	393.3734	0.883189	1757.257
7.854473	1014.61	335.897	184.492	209.822	274.534	123.7461	2.737207	1736.878
12.52371	993.896	366.841	158.388	303.399	233.035	324.8262	4.430806	1452.51
9.044802	991.827	354.167	182.75	290.509	238.406	393.3734	0.883189	1757.257
4.000544	1011.503	351.963	179.538	281.873	241.433	371.2694	8.848426	2283.271
9.044802	991.827	354.167	182.75	290.509	238.406	393.3734	0.883189	1757.257
16.90351	986.811	363.566	161.582	264.788	242.427	353.8546	1.464825	1792.748
14.63104	838.498	327.419	138.423	194.725	283.686	207.2583	1.315532	1774.275
7.11078	1049.088	353.943	146.009	284.948	296.027	202.8772	0.626459	1847.405
4.000544	1011.503	351.963	179.538	281.873	241.433	371.2694	8.848426	2283.271
2.915978	1181.614	463.026	199.043	344.474	266.835	560.2236	2.186021	2000.388
14.25296	913.857	345.469	137.96	189.659	287.484	60.08836	2.996304	1851.407
12.52371	993.896	366.841	158.388	303.399	233.035	324.8262	4.430806	1452.51
-1.34088	925.463	302.666	166.731	241.107	214.488	502.9605	5.291672	1632.742
7.11078	1049.088	353.943	146.009	284.948	296.027	202.8772	0.626459	1847.405
14.53205	1109.398	381.222	185.432	255.372	311.253	90.55988	3.017958	1646.286
4.000544	1011.503	351.963	179.538	281.873	241.433	371.2694	8.848426	2283.271
10.92335	976.525	356.957	166.786	307.865	215.414	383.0372	3.69426	1981.62
14.76842	1009.45	358.954	157.905	225.445	283.931	111.7041	1.756512	1916.818
-1.80355	982.029	384.009	170.345	232.275	215.746	464.0417	2.215279	1849.836
8.854786	1061.025	387.175	189.077	311.413	246.257	334.6038	2.363094	1913.333
12.52371	993.896	366.841	158.388	303.399	233.035	324.8262	4.430806	1452.51
14.53205	1109.398	381.222	185.432	255.372	311.253	90.55988	3.017958	1646.286
12.52371	993.896	366.841	158.388	303.399	233.035	326.726	4.709216	1576.021
4.12744	1143.212	368.326	233.217	302.355	303.292	332.479	1.43155	1747.347
15.75438	900.163	331.78	120.801	249.553	271.399	201.7106	1.461046	1938.337
12.51116	1099.962	394.828	165.691	348.036	290.794	302.7306	0.714524	1821.651
12.52371	993.896	366.841	158.388	303.399	233.035	326.726	4.709216	1576.021
6.99744	1068.364	377.113	166.177	273.454	299.788	135.5694	8.309216	1940.668
2.915978	1181.614	463.026	199.043	344.474	266.835	560.2236	2.186021	2000.388
-0.98983	956.144	346.759	160.414	239.894	218.472	333.6983	3.161077	1300.838
14.25296	913.857	345.469	137.96	189.659	287.484	60.08836	2.996304	1851.407
10.92335	976.525	356.957	166.786	307.865	215.414	383.0372	3.69426	1981.62
7.56167	1085.974	370.378	169.387	288.858	317.829	300.4833	2.304261	1756.669
12.52371	993.896	366.841	158.388	303.399	233.035	324.8262	4.430806	1452.51
4.000544	1011.503	351.963	179.538	281.873	241.433	371.2694	8.848426	2283.271
3.715302	835.73	299.191	144.341	227.131	219.342	371.2189	6.547291	2148.801
0.479769	1017.669	352.698	182.124	285.505	245.116	351.0301	7.706647	1808.782
-0.98983	956.144	346.759	160.414	239.894	218.472	333.6983	3.161077	1300.838
-1.80355	982.029	384.009	170.345	232.275	215.746	464.0417	2.215279	1849.836
13.84265	1240.789	517.558	149.909	474.696	246.821	261.3956	2.788267	1863.578

Table S2 - Anonymized database

3.715302	835.73	299.191	144.341	227.131	219.342	371.2189	6.547291	2148.801
14.26695	975.32	347.324	129.172	244.574	273.307	94.82304	0.619173	1813.049
4.000544	1011.503	351.963	179.538	281.873	241.433	371.2694	8.848426	2283.271
12.52371	993.896	366.841	158.388	303.399	233.035	326.726	4.709216	1576.021
8.854786	1061.025	387.175	189.077	311.413	246.257	334.6038	2.363094	1913.333
14.25296	913.857	345.469	137.96	189.659	287.484	60.08836	2.996304	1851.407
13.43378	1174.369	424.168	207.11	349.535	265.892	399.359	3.519502	1809.91
12.52371	993.896	366.841	158.388	303.399	233.035	324.8262	4.430806	1452.51
14.53205	1109.398	381.222	185.432	255.372	311.253	90.55988	3.017958	1646.286
12.52371	993.896	366.841	158.388	303.399	233.035	326.726	4.709216	1576.021
15.75438	900.163	331.78	120.801	249.553	271.399	201.7106	1.461046	1938.337
2.915978	1181.614	463.026	199.043	344.474	266.835	560.2236	2.186021	2000.388
15.58609	1000.009	361.433	179.124	201.378	253.201	67.61459	3.770963	2000.362
15.12292	998.573	362.943	154.869	227.313	280.001	65.75416	1.331257	1836.56
8.854786	1061.025	387.175	189.077	311.413	246.257	334.6038	2.363094	1913.333
13.84265	1240.789	517.558	149.909	474.696	246.821	261.3956	2.788267	1863.578
3.715302	835.73	299.191	144.341	227.131	219.342	371.2189	6.547291	2148.801
12.52371	993.896	366.841	158.388	303.399	233.035	326.726	4.709216	1576.021
14.14814	987.4	387.621	161.22	214.5	325.319	138.9436	4.177484	1768.744
10.92335	976.525	356.957	166.786	307.865	215.414	383.0372	3.69426	1981.62
14.14814	987.4	387.621	161.22	214.5	325.319	138.9436	4.177484	1768.744
1.375121	1021.311	345.988	189.06	237.048	251.852	342.6181	4.375702	1469.899
12.51116	1099.962	394.828	165.691	348.036	290.794	302.7306	0.714524	1821.651
14.76842	1009.45	358.954	157.905	225.445	283.931	111.7041	1.756512	1916.818
0.479769	1017.669	352.698	182.124	285.505	245.116	351.0301	7.706647	1808.782
0.498819	1041.527	361.072	186.248	309.375	247.21	344.0219	8.271492	2272.567
8.854786	1061.025	387.175	189.077	311.413	246.257	334.6038	2.363094	1913.333
4.12744	1143.212	368.326	233.217	302.355	303.292	332.479	1.43155	1747.347
6.909423	992.342	340.909	154.232	245.555	285.881	184.0726	2.807042	1659.809
12.52371	993.896	366.841	158.388	303.399	233.035	324.8262	4.430806	1452.51
10.92335	976.525	356.957	166.786	307.865	215.414	383.0372	3.69426	1981.62
14.25296	913.857	345.469	137.96	189.659	287.484	60.08836	2.996304	1851.407
14.68604	944.988	330.019	134.217	209.285	286.834	132.9971	1.029028	1796.389
7.56167	1085.974	370.378	169.387	288.858	317.829	300.4833	2.304261	1756.669
12.51116	1099.962	394.828	165.691	348.036	290.794	302.7306	0.714524	1821.651
2.915978	1181.614	463.026	199.043	344.474	266.835	560.2236	2.186021	2000.388
-1.34088	925.463	302.666	166.731	241.107	214.488	502.9605	5.291672	1632.742
9.354027	957.175	340.137	181.652	277.985	229.463	288.2634	7.966951	2209.513
-1.34088	925.463	302.666	166.731	241.107	214.488	502.9605	5.291672	1632.742
14.32436	957.012	345.225	160.053	205.484	283.088	73.95937	4.403274	1673.257
-0.16976	1173.229	429.033	154.596	419.01	170.398	324.8262	4.430806	1452.51
-1.24171	1063.862	365.344	157.394	355.954	171.554	358.2814	3.191571	1939.405
16.66705	1638.333	620.346	275.441	543.848	283.835	581.6044	5.521491	1480.118
0.771571	1197.382	458.126	182.4	415.096	182.4	326.7982	10.80945	1379.297
0.851703	1355.105	492.748	223.249	415.345	223.249	344.9812	0.403961	1862.702
0.520451	1256.469	464.044	198.199	401.5	198.199	341.0001	4.046354	2112.621
0.621198	1219.512	468.387	202.814	393.063	202.814	311.9337	7.613102	2064.278
-0.16976	1173.229	429.033	154.596	419.01	170.398	324.8262	4.430806	1452.51
1.689896	977.137	384.824	146.945	333.587	146.945	106.5691	4.151762	1547.465
-0.16976	1173.229	429.033	154.596	419.01	170.398	324.8262	4.430806	1452.51

Table S2 - Anonymized database

19.98208	1282.251	428.942	199.648	282.135	301.821	261.3956	2.788267	1863.578
18.51983	1182.28	468.97	149.281	389.263	228.039	311.9507	4.48972	1438.063
16.66705	1638.333	620.346	275.441	543.848	283.835	581.6044	5.521491	1480.118
0.621198	1219.512	468.387	202.814	393.063	202.814	311.9337	7.613102	2064.278
1.34567	1036.298	416.576	125.763	351.163	125.763	98.39349	4.764332	1521.834
0.621198	1219.512	468.387	202.814	393.063	202.814	311.9337	7.613102	2064.278
18.51983	1182.28	468.97	149.281	389.263	228.039	311.9507	4.48972	1438.063
18.06966	1187.626	460.407	120.673	370.06	252.737	353.7827	1.677322	1845.688
1.222846	1081.288	481.4	110.162	440.515	110.162	138.7071	2.50683	1815.574
-0.16976	1173.229	429.033	154.596	419.01	170.398	324.8262	4.430806	1452.51
-1.68034	1121.098	416.359	203.313	357.376	203.313	444.7566	3.02755	1895.277
1.222846	1081.288	481.4	110.162	440.515	110.162	138.7071	2.50683	1815.574
0.621198	1219.512	468.387	202.814	393.063	202.814	311.9337	7.613102	2064.278
-1.24171	1063.862	365.344	157.394	355.954	171.554	358.2814	3.191571	1939.405
1.266747	1132.892	504.388	117.166	469.63	117.166	130.4095	12.40068	2461.734
18.51983	1182.28	468.97	149.281	389.263	228.039	311.9507	4.48972	1438.063
1.34567	1036.298	416.576	125.763	351.163	125.763	98.39349	4.764332	1521.834
1.222846	1081.288	481.4	110.162	440.515	110.162	138.7071	2.50683	1815.574
1.222846	1081.288	481.4	110.162	440.515	110.162	138.7071	2.50683	1815.574
0.913973	1173.967	450.496	167.028	431.8	167.028	252.7432	7.030299	2220.283
10.40388	1050.159	366.522	140.553	358.447	160.446	257.2712	12.69096	821.2842
6.802302	1272.427	502.081	162.056	449.659	195.962	499.9418	1.755741	1653.932
17.96497	1217.182	430.052	155.312	302.152	227.337	330.8731	0.217345	1789.407
1.250885	1300.403	531.24	183.097	512.437	183.097	332.2233	16.70643	1434.77
18.51983	1182.28	468.97	149.281	389.263	228.039	311.9507	4.48972	1438.063
0.924357	1193.517	454.643	160.198	433.523	160.198	269.1555	0.307515	1886.006
-0.16976	1173.229	429.033	154.596	419.01	170.398	324.8262	4.430806	1452.51
10.41302	1107.858	399.833	144.454	360.669	180.515	442.1107	8.673082	1871.338
1.748725	1014.328	389.7	141.235	333.39	141.235	185.4104	10.88632	2682.429
0.913973	1173.967	450.496	167.028	431.8	167.028	252.7432	7.030299	2220.283
6.802302	1272.427	502.081	162.056	449.659	195.962	499.9418	1.755741	1653.932
16.66705	1638.333	620.346	275.441	543.848	283.835	581.6044	5.521491	1480.118
1.34567	1036.298	416.576	125.763	351.163	125.763	98.39349	4.764332	1521.834
18.51983	1182.28	468.97	149.281	389.263	228.039	311.9507	4.48972	1438.063
1.34567	1036.298	416.576	125.763	351.163	125.763	98.39349	4.764332	1521.834
1.154319	1069.139	395.328	155.942	377.332	155.942	99.84711	3.078826	1649.392
0.082527	1290.431	569.334	129.365	564.797	129.365	222.9399	2.51953	2007.912
10.41302	1107.858	399.833	144.454	360.669	180.515	442.1107	8.673082	1871.338
18.31974	1205.787	450.19	167.266	347.842	213.155	342.0194	5.998885	1432.308
1.34567	1036.298	416.576	125.763	351.163	125.763	98.39349	4.764332	1521.834
0.621198	1219.512	468.387	202.814	393.063	202.814	311.9337	7.613102	2064.278
1.591879	1120.093	442.314	150.672	406.007	150.672	78.42129	6.451437	1501.143
1.154319	1069.139	395.328	155.942	377.332	155.942	99.84711	3.078826	1649.392
1.34567	1036.298	416.576	125.763	351.163	125.763	98.39349	4.764332	1521.834
1.222846	1081.288	481.4	110.162	440.515	110.162	138.7071	2.50683	1815.574
18.06966	1187.626	460.407	120.673	370.06	252.737	353.7827	1.677322	1845.688
1.222846	1081.288	481.4	110.162	440.515	110.162	138.7071	2.50683	1815.574
1.193769	1313.203	540.484	182.942	510.422	182.942	353.818	9.800788	2414.645
0.082527	1290.431	569.334	129.365	564.797	129.365	222.9399	2.51953	2007.912
16.66705	1638.333	620.346	275.441	543.848	283.835	581.6044	5.521491	1480.118

Table S2 - Anonymized database

1.689896	977.137	384.824	146.945	333.587	146.945	106.5691	4.151762	1547.465
1.591879	1120.093	442.314	150.672	406.007	150.672	78.42129	6.451437	1501.143
0.816011	1260.301	478.654	184.986	452.367	184.986	311.8525	9.304274	2253.092
1.34567	1036.298	416.576	125.763	351.163	125.763	98.39349	4.764332	1521.834
6.802302	1272.427	502.081	162.056	449.659	195.962	499.9418	1.755741	1653.932
1.601962	1072.172	436.875	137.997	384.836	137.997	218.33	14.04217	963.5971
0.621198	1219.512	468.387	202.814	393.063	202.814	311.9337	7.613102	2064.278
0.278016	1231.077	450.28	214.251	388.43	214.251	350.8546	4.056374	2002.568
0.816011	1260.301	478.654	184.986	452.367	184.986	311.8525	9.304274	2253.092
1.222846	1081.288	481.4	110.162	440.515	110.162	138.7071	2.50683	1815.574
18.51983	1182.28	468.97	149.281	389.263	228.039	311.9507	4.48972	1438.063
1.193769	1313.203	540.484	182.942	510.422	182.942	353.818	9.800788	2414.645
19.98208	1282.251	428.942	199.648	282.135	301.821	261.3956	2.788267	1863.578
17.83516	1263.249	455.287	161.919	339.602	231.219	368.9091	0.768818	1715.272
2.199714	1096.915	414.401	166.252	391.109	169.133	90.27238	2.59097	1700.188
6.802302	1272.427	502.081	162.056	449.659	195.962	499.9418	1.755741	1653.932
1.14756	1229.533	480.647	158.43	449.146	158.43	320.661	3.05985	1967.903
6.802302	1272.427	502.081	162.056	449.659	195.962	499.9418	1.755741	1653.932
0.951566	1240.925	465.009	181.022	445.045	181.022	270.6296	0.168178	1811.263
0.771571	1197.382	458.126	182.4	415.096	182.4	326.7982	10.80945	1379.297
0.913973	1173.967	450.496	167.028	431.8	167.028	252.7432	7.030299	2220.283
0.542005	1264.835	458.168	192.243	422.977	192.243	344.0219	8.271492	2272.567
0.437352	1192.216	424.53	193.203	358.705	193.203	369.287	5.148047	1885.585
-0.78801	1101.295	387.341	150.397	374.596	161.97	487.1186	2.469619	1975.047
1.222846	1081.288	481.4	110.162	440.515	110.162	138.7071	2.50683	1815.574
0.951566	1240.925	465.009	181.022	445.045	181.022	270.6296	0.168178	1811.263
0.913973	1173.967	450.496	167.028	431.8	167.028	252.7432	7.030299	2220.283
16.66705	1638.333	620.346	275.441	543.848	283.835	581.6044	5.521491	1480.118
1.34567	1036.298	416.576	125.763	351.163	125.763	98.39349	4.764332	1521.834
0.621198	1219.512	468.387	202.814	393.063	202.814	311.9337	7.613102	2064.278
1.584154	1113.703	482.577	139.954	429.346	139.954	256.7606	3.657492	1909.23
0.082527	1290.431	569.334	129.365	564.797	129.365	222.9399	2.51953	2007.912
1.601962	1072.172	436.875	137.997	384.836	137.997	218.33	14.04217	963.5971
-1.4519	1086.461	453.6	123.568	373.129	150.863	340.6455	9.196588	2278.729
1.154319	1069.139	395.328	155.942	377.332	155.942	99.84711	3.078826	1649.392
1.601962	1072.172	436.875	137.997	384.836	137.997	218.33	14.04217	963.5971
0.437352	1192.216	424.53	193.203	358.705	193.203	346.348	3.354737	2021.434
1.601962	1072.172	436.875	137.997	384.836	137.997	218.33	14.04217	963.5971
1.222846	1081.288	481.4	110.162	440.515	110.162	138.7071	2.50683	1815.574
-0.16976	1173.229	429.033	154.596	419.01	170.398	324.8262	4.430806	1452.51
-0.16976	1173.229	429.033	154.596	419.01	170.398	324.8262	4.430806	1452.51
-0.16976	1173.229	429.033	154.596	419.01	170.398	324.8262	4.430806	1452.51
6.802302	1272.427	502.081	162.056	449.659	195.962	499.9418	1.755741	1653.932
16.66705	1638.333	620.346	275.441	543.848	283.835	581.6044	5.521491	1480.118
0.914132	1169.055	429.019	168.008	424.65	168.008	169.8027	1.355794	1924.805
1.222846	1081.288	481.4	110.162	440.515	110.162	138.7071	2.50683	1815.574
1.689896	977.137	384.824	146.945	333.587	146.945	106.5691	4.151762	1547.465
1.250885	1300.403	531.24	183.097	512.437	183.097	332.2233	16.70643	1434.77
0.621198	1219.512	468.387	202.814	393.063	202.814	311.9337	7.613102	2064.278
10.41302	1107.858	399.833	144.454	360.669	180.515	442.1107	8.673082	1871.338

Table S2 - Anonymized database

1.222846	1081.288	481.4	110.162	440.515	110.162	138.7071	2.50683	1815.574
16.66705	1638.333	620.346	275.441	543.848	283.835	581.6044	5.521491	1480.118
1.222846	1081.288	481.4	110.162	440.515	110.162	138.7071	2.50683	1815.574
0.816011	1260.301	478.654	184.986	452.367	184.986	311.8525	9.304274	2253.092
1.601962	1072.172	436.875	137.997	384.836	137.997	218.33	14.04217	963.5971
0.437352	1192.216	424.53	193.203	358.705	193.203	346.348	3.354737	2021.434
1.603604	1039.627	370.788	151.062	360.051	151.062	118.0156	12.89384	1712.243
19.98208	1282.251	428.942	199.648	282.135	301.821	261.3956	2.788267	1863.578
19.98208	1282.251	428.942	199.648	282.135	301.821	261.3956	2.788267	1863.578
0.437352	1192.216	424.53	193.203	358.705	193.203	346.348	3.354737	2021.434
-0.16976	1173.229	429.033	154.596	419.01	170.398	324.8262	4.430806	1452.51
1.309764	1202.427	471.813	175.725	450.776	175.725	302.5654	5.50175	1736.775
0.082527	1290.431	569.334	129.365	564.797	129.365	222.9399	2.51953	2007.912
10.41302	1107.858	399.833	144.454	360.669	180.515	442.1107	8.673082	1871.338
8.643956	1438.028	548.298	229.766	513.314	249.796	663.8906	3.372374	2187.516
1.222846	1081.288	481.4	110.162	440.515	110.162	138.7071	2.50683	1815.574
0.621198	1219.512	468.387	202.814	393.063	202.814	311.9337	7.613102	2064.278
0.715363	1169.376	440.42	164.323	432.232	164.323	214.8781	5.50378	1491.958
18.51983	1182.28	468.97	149.281	389.263	228.039	311.9507	4.48972	1438.063
0.913973	1173.967	450.496	167.028	431.8	167.028	252.7432	7.030299	2220.283
-0.16976	1173.229	429.033	154.596	419.01	170.398	324.8262	4.430806	1452.51
1.34567	1036.298	416.576	125.763	351.163	125.763	98.39349	4.764332	1521.834
0.542005	1264.835	458.168	192.243	422.977	192.243	344.0219	8.271492	2272.567
1.34567	1036.298	416.576	125.763	351.163	125.763	98.39349	4.764332	1521.834
1.193769	1313.203	540.484	182.942	510.422	182.942	353.818	9.800788	2414.645
18.06966	1187.626	460.407	120.673	370.06	252.737	353.7827	1.677322	1845.688
1.222846	1081.288	481.4	110.162	440.515	110.162	138.7071	2.50683	1815.574
1.154319	1069.139	395.328	155.942	377.332	155.942	99.84711	3.078826	1649.392
1.34567	1036.298	416.576	125.763	351.163	125.763	98.39349	4.764332	1521.834
0.437352	1192.216	424.53	193.203	358.705	193.203	346.348	3.354737	2021.434
-0.16976	1173.229	429.033	154.596	419.01	170.398	324.8262	4.430806	1452.51
0.851703	1355.105	492.748	223.249	415.345	223.249	344.9812	0.403961	1862.702
1.34567	1036.298	416.576	125.763	351.163	125.763	98.39349	4.764332	1521.834
1.222846	1081.288	481.4	110.162	440.515	110.162	138.7071	2.50683	1815.574
1.222846	1081.288	481.4	110.162	440.515	110.162	138.7071	2.50683	1815.574
-1.4519	1086.461	453.6	123.568	373.129	150.863	340.6455	9.196588	2278.729
6.802302	1272.427	502.081	162.056	449.659	195.962	499.9418	1.755741	1653.932
1.222846	1081.288	481.4	110.162	440.515	110.162	138.7071	2.50683	1815.574
17.84441	1261.902	481.286	151.452	365.826	231.318	417.374	1.331445	1795.906
1.34567	1036.298	416.576	125.763	351.163	125.763	98.39349	4.764332	1521.834
1.28983	1198.578	501.065	158.017	491.325	158.017	83.46487	4.532743	1482.57
0.913973	1173.967	450.496	167.028	431.8	167.028	252.7432	7.030299	2220.283
0.913973	1173.967	450.496	167.028	431.8	167.028	252.7432	7.030299	2220.283
16.66705	1638.333	620.346	275.441	543.848	283.835	581.6044	5.521491	1480.118
1.222846	1081.288	481.4	110.162	440.515	110.162	138.7071	2.50683	1815.574
16.66705	1638.333	620.346	275.441	543.848	283.835	581.6044	5.521491	1480.118
0.437352	1192.216	424.53	193.203	358.705	193.203	346.348	3.354737	2021.434
0.278016	1231.077	450.28	214.251	388.43	214.251	350.8546	4.056374	2002.568
16.66705	1638.333	620.346	275.441	543.848	283.835	581.6044	5.521491	1480.118
1.222846	1081.288	481.4	110.162	440.515	110.162	138.7071	2.50683	1815.574

Table S2 - Anonymized database

1.34567	1036.298	416.576	125.763	351.163	125.763	98.39349	4.764332	1521.834
0.437352	1192.216	424.53	193.203	358.705	193.203	346.348	3.354737	2021.434
1.309764	1202.427	471.813	175.725	450.776	175.725	302.5654	5.50175	1736.775
10.41302	1107.858	399.833	144.454	360.669	180.515	442.1107	8.673082	1871.338
1.34567	1036.298	416.576	125.763	351.163	125.763	98.39349	4.764332	1521.834
1.34567	1036.298	416.576	125.763	351.163	125.763	98.39349	4.764332	1521.834
18.31974	1205.787	450.19	167.266	347.842	213.155	342.0194	5.998885	1432.308
0.816011	1260.301	478.654	184.986	452.367	184.986	311.8525	9.304274	2253.092
0.082527	1290.431	569.334	129.365	564.797	129.365	222.9399	2.51953	2007.912
1.309764	1202.427	471.813	175.725	450.776	175.725	302.5654	5.50175	1736.775
1.601962	1072.172	436.875	137.997	384.836	137.997	218.33	14.04217	963.5971
0.913973	1173.967	450.496	167.028	431.8	167.028	252.7432	7.030299	2220.283
17.71124	1371.437	527.992	195.869	453.861	282.068	376.0003	5.043976	1765.235
0.771571	1197.382	458.126	182.4	415.096	182.4	326.7982	10.80945	1379.297
1.154319	1069.139	395.328	155.942	377.332	155.942	99.84711	3.078826	1649.392
-1.68034	1121.098	416.359	203.313	357.376	203.313	444.7566	3.02755	1895.277
17.96497	1217.182	430.052	155.312	302.152	227.337	330.8731	0.217345	1789.407
0.851703	1355.105	492.748	223.249	415.345	223.249	344.9812	0.403961	1862.702
1.34567	1036.298	416.576	125.763	351.163	125.763	98.39349	4.764332	1521.834
0.520489	1214.764	431.146	202.207	358.102	202.207	314.7635	8.712111	2402.854
18.31974	1205.787	450.19	167.266	347.842	213.155	342.0194	5.998885	1432.308
0.542005	1264.835	458.168	192.243	422.977	192.243	344.0219	8.271492	2272.567
19.98208	1282.251	428.942	199.648	282.135	301.821	261.3956	2.788267	1863.578
1.34567	1036.298	416.576	125.763	351.163	125.763	98.39349	4.764332	1521.834
-0.16976	1173.229	429.033	154.596	419.01	170.398	324.8262	4.430806	1452.51
17.84441	1261.902	481.286	151.452	365.826	231.318	417.374	1.331445	1795.906
1.193769	1313.203	540.484	182.942	510.422	182.942	353.818	9.800788	2414.645
1.601962	1072.172	436.875	137.997	384.836	137.997	218.33	14.04217	963.5971
0.913973	1173.967	450.496	167.028	431.8	167.028	252.7432	7.030299	2220.283
18.31974	1205.787	450.19	167.266	347.842	213.155	342.0194	5.998885	1432.308
18.31974	1205.787	450.19	167.266	347.842	213.155	342.0194	5.998885	1432.308
0.082527	1290.431	569.334	129.365	564.797	129.365	222.9399	2.51953	2007.912
0.437352	1192.216	424.53	193.203	358.705	193.203	369.287	5.148047	1885.585
-0.16976	1173.229	429.033	154.596	419.01	170.398	324.8262	4.430806	1452.51
17.96497	1217.182	430.052	155.312	302.152	227.337	330.8731	0.217345	1789.407
1.222846	1081.288	481.4	110.162	440.515	110.162	138.7071	2.50683	1815.574
0.621198	1219.512	468.387	202.814	393.063	202.814	311.9337	7.613102	2064.278
18.51983	1182.28	468.97	149.281	389.263	228.039	311.9507	4.48972	1438.063
18.31974	1205.787	450.19	167.266	347.842	213.155	342.0194	5.998885	1432.308
1.601962	1072.172	436.875	137.997	384.836	137.997	218.33	14.04217	963.5971
1.154319	1069.139	395.328	155.942	377.332	155.942	99.84711	3.078826	1649.392
1.222846	1081.288	481.4	110.162	440.515	110.162	138.7071	2.50683	1815.574
0.851703	1355.105	492.748	223.249	415.345	223.249	344.9812	0.403961	1862.702
-0.16976	1173.229	429.033	154.596	419.01	170.398	324.8262	4.430806	1452.51
18.06966	1187.626	460.407	120.673	370.06	252.737	353.7827	1.677322	1845.688
1.222846	1081.288	481.4	110.162	440.515	110.162	138.7071	2.50683	1815.574
-0.16976	1173.229	429.033	154.596	419.01	170.398	324.8262	4.430806	1452.51
1.34567	1036.298	416.576	125.763	351.163	125.763	98.39349	4.764332	1521.834
-0.16976	1173.229	429.033	154.596	419.01	170.398	324.8262	4.430806	1452.51
-0.16976	1173.229	429.033	154.596	419.01	170.398	324.8262	4.430806	1452.51

Table S2 - Anonymized database

0.771571	1197.382	458.126	182.4	415.096	182.4	326.7982	10.80945	1379.297
18.51983	1182.28	468.97	149.281	389.263	228.039	311.9507	4.48972	1438.063
16.66705	1638.333	620.346	275.441	543.848	283.835	581.6044	5.521491	1480.118
0.082527	1290.431	569.334	129.365	564.797	129.365	222.9399	2.51953	2007.912
1.601962	1072.172	436.875	137.997	384.836	137.997	218.33	14.04217	963.5971
1.222846	1081.288	481.4	110.162	440.515	110.162	138.7071	2.50683	1815.574
-1.24171	1063.862	365.344	157.394	355.954	171.554	358.2814	3.191571	1939.405
0.771571	1197.382	458.126	182.4	415.096	182.4	326.7982	10.80945	1379.297
18.51983	1182.28	468.97	149.281	389.263	228.039	311.9507	4.48972	1438.063
1.918511	1053.9	391.572	139.722	345.5	139.722	259.744	7.931207	1889.531
1.620286	1046.792	412.833	141.234	346.007	141.234	82.30245	4.124621	1880.85
1.222846	1081.288	481.4	110.162	440.515	110.162	138.7071	2.50683	1815.574
-0.16976	1173.229	429.033	154.596	419.01	170.398	324.8262	4.430806	1452.51
16.66705	1638.333	620.346	275.441	543.848	283.835	581.6044	5.521491	1480.118
1.197742	1080.637	418.909	149.214	375.353	149.214	220.2656	6.243832	2323.249
1.34567	1036.298	416.576	125.763	351.163	125.763	98.39349	4.764332	1521.834
18.51983	1182.28	468.97	149.281	389.263	228.039	311.9507	4.48972	1438.063
1.34567	1036.298	416.576	125.763	351.163	125.763	98.39349	4.764332	1521.834
1.601962	1072.172	436.875	137.997	384.836	137.997	218.33	14.04217	963.5971
-0.16976	1173.229	429.033	154.596	419.01	170.398	324.8262	4.430806	1452.51
-0.16976	1173.229	429.033	154.596	419.01	170.398	324.8262	4.430806	1452.51
1.34567	1036.298	416.576	125.763	351.163	125.763	98.39349	4.764332	1521.834
0.913973	1173.967	450.496	167.028	431.8	167.028	252.7432	7.030299	2220.283
0.913973	1173.967	450.496	167.028	431.8	167.028	252.7432	7.030299	2220.283
0.520489	1214.764	431.146	202.207	358.102	202.207	314.7635	8.712111	2402.854
18.31974	1205.787	450.19	167.266	347.842	213.155	342.0194	5.998885	1432.308
1.34567	1036.298	416.576	125.763	351.163	125.763	98.39349	4.764332	1521.834
10.41302	1107.858	399.833	144.454	360.669	180.515	442.1107	8.673082	1871.338
1.601962	1072.172	436.875	137.997	384.836	137.997	218.33	14.04217	963.5971
18.06966	1187.626	460.407	120.673	370.06	252.737	353.7827	1.677322	1845.688
1.309764	1202.427	471.813	175.725	450.776	175.725	302.5654	5.50175	1736.775
17.96497	1217.182	430.052	155.312	302.152	227.337	330.8731	0.217345	1789.407
19.98208	1282.251	428.942	199.648	282.135	301.821	261.3956	2.788267	1863.578
0.771571	1197.382	458.126	182.4	415.096	182.4	326.7982	10.80945	1379.297
10.41302	1107.858	399.833	144.454	360.669	180.515	442.1107	8.673082	1871.338
12.07976	1683.682	592.625	305.189	507.767	402.879	92.29993	9.324022	1126.678
-3.14471	1338.751	410.37	265.597	404.62	271.411	482.474	3.426512	1754.312
11.77305	1798.059	729.666	298.193	624.238	390.883	158.5528	2.500618	2028.029
1.527967	1593.369	653.504	239.989	652.947	263.645	222.4361	2.733181	1472.043
2.309527	1759.552	665.196	297.839	608.559	382.53	231.4455	5.869617	2064.344
1.980445	1420.531	451.953	284.108	376.634	292.057	415.1788	7.663112	2302.833
3.673863	1689.434	533.232	297.784	533.232	348.849	353.818	9.800788	2414.645
0.693659	1641.955	674.39	234.111	617.163	294.82	211.2159	8.984717	1305.867
3.224808	1725.185	734.304	267.716	605.522	351.814	118.4804	4.569775	1687.026
2.994984	1636.069	511.474	292.264	370.066	332.285	363.2772	6.386463	2184.072
1.165121	1758.55	773.716	260.25	620.897	324.493	240.9818	2.35066	1953.851
-1.55404	1655.204	707.488	235.803	692.561	285.481	489.7062	3.59755	1702.217
9.255143	1650.811	552.766	309.093	552.766	317.585	384.1146	8.195766	2390.127
9.765038	1726.196	579.005	315.421	579.005	318.305	367.2032	9.372241	1854.878
1.36717	1631.658	686.213	240.917	561.508	319.077	161.0517	3.782925	1557.11

Table S2 - Anonymized database

4.306681	1669.886	546.443	290.398	461.013	447.037	28.46955	0.679182	1846.094
0.977159	1594.237	664.744	241.42	613.571	280.445	344.4803	2.282428	1975.805
2.775	1706.642	622.925	269.626	594.823	367.238	151.6085	3.044361	1695.477
3.082264	1841.716	765.463	299.75	657.881	389.15	187.7079	2.024981	2018.113
2.602055	1673.899	547.323	330.145	481.046	374.802	161.7074	3.475744	1856.403
1.495863	1907.282	690.445	334.487	517.025	436.375	148.2937	1.099489	1699.062
2.92322	1869.494	701.625	331.039	646.369	378.381	118.1115	1.501855	1862.66
3.078527	1709.982	751.458	244.056	643.587	367.179	191.9448	3.036096	2134.077
3.312335	1748.048	605.137	330.629	521.998	401.257	121.9577	2.032796	1891.805
10.2139	1949.674	754.498	311.599	738.447	399.291	126.3232	4.08183	1849.596
0.089544	1624.298	659.248	248.595	629.329	291.427	367.653	6.092455	2268.084
-1.24902	1802.949	615.544	299.283	536.595	299.283	367.5201	5.641701	2122.695
-1.72355	1358.596	581.878	172.581	523.931	235.866	693.117	3.449864	1538.647
2.861005	1876.506	770.783	302.223	712.595	376.309	131.5466	7.098525	1302.395
10.2139	1949.674	754.498	311.599	738.447	399.291	126.3232	4.08183	1849.596
3.539126	1680.951	554.388	288.569	403.414	299.121	317.2497	11.81392	959.2725
3.078527	1709.982	751.458	244.056	643.587	367.179	191.9448	3.036096	2134.077
1.165121	1758.55	773.716	260.25	620.897	324.493	240.9818	2.35066	1953.851
1.527967	1593.369	653.504	239.989	652.947	263.645	222.4361	2.733181	1472.043
1.517253	1619.106	647.824	248.946	647.824	267.039	330.4612	5.314943	2050.618
3.269582	1761.512	673.087	252.466	580.927	351.081	186.2722	14.29552	2568.046
3.191758	1749.745	600.434	315.678	581.92	364.259	71.10771	0.571329	1809.155
0.057346	1641.624	688.454	233.49	652.575	304.641	423.5338	15.46822	1331.014
2.739203	1760.081	718.746	280.373	654.203	347.181	162.8161	1.964231	1741.297
2.515495	1766.287	753.767	272.141	674.837	333.336	147.7344	2.213931	1994.002
1.314016	1682.612	746.082	207.825	675.088	320.78	172.1728	4.648338	2040.351
0.693659	1641.955	674.39	234.111	617.163	294.82	211.2159	8.984717	1305.867
12.69676	1795.836	596.635	330.645	435.48	447.855	87.46478	2.164512	1745.672
3.224808	1725.185	734.304	267.716	605.522	351.814	118.4804	4.569775	1687.026
9.674176	1677.047	747.362	233.006	715.846	287.275	500.629	1.819382	1815.314
3.078527	1709.982	751.458	244.056	643.587	367.179	191.9448	3.036096	2134.077
0.693659	1641.955	674.39	234.111	617.163	294.82	211.2159	8.984717	1305.867
9.674176	1677.047	747.362	233.006	715.846	287.275	500.629	1.819382	1815.314
3.078527	1709.982	751.458	244.056	643.587	367.179	191.9448	3.036096	2134.077
1.165121	1758.55	773.716	260.25	620.897	324.493	240.9818	2.35066	1953.851
-2.73457	1690.79	575.959	263.325	529.174	263.325	602.6755	10.18564	2336.094
10.2139	1949.674	754.498	311.599	738.447	399.291	126.3232	4.08183	1849.596
9.674176	1677.047	747.362	233.006	715.846	287.275	500.629	1.819382	1815.314
2.861005	1876.506	770.783	302.223	712.595	376.309	131.5466	7.098525	1302.395
-2.32548	1444.681	448.344	270.455	395.367	270.455	330.781	0.599844	1782.226
-0.54135	1745.066	603.212	306.222	572.405	308.373	294.5679	3.569889	2092.734
-2.0658	1737.211	552.653	308.083	492.523	308.083	559.32	6.887537	1658.008
2.515495	1766.287	753.767	272.141	674.837	333.336	147.7344	2.213931	1994.002
0.977159	1594.237	664.744	241.42	613.571	280.445	344.4803	2.282428	1975.805
2.994984	1636.069	511.474	292.264	370.066	332.285	363.2772	6.386463	2184.072
1.165121	1758.55	773.716	260.25	620.897	324.493	240.9818	2.35066	1953.851
9.714725	2126.397	975.95	282.783	975.95	390.961	197.695	5.642335	2223.441
3.078527	1709.982	751.458	244.056	643.587	367.179	191.9448	3.036096	2134.077
1.599203	1874.653	773.233	277.26	704.633	364.567	427.536	23.76717	3358.25
3.078527	1709.982	751.458	244.056	643.587	367.179	191.9448	3.036096	2134.077

Table S2 - Anonymized database

-2.48813	1431.669	416.868	267.291	405.63	267.291	379.0812	2.998592	1846.541
0.977159	1594.237	664.744	241.42	613.571	280.445	344.4803	2.282428	1975.805
9.255143	1650.811	552.766	309.093	552.766	317.585	384.1146	8.195766	2390.127
1.527967	1593.369	653.504	239.989	652.947	263.645	222.4361	2.733181	1472.043
3.224808	1725.185	734.304	267.716	605.522	351.814	118.4804	4.569775	1687.026
-2.66538	1472.877	466.147	269.666	425.297	269.666	385.9416	16.94996	446.4642
0.977159	1594.237	664.744	241.42	613.571	280.445	344.4803	2.282428	1975.805
-2.15702	1375.056	425.211	233.701	379.362	233.701	347.2733	4.467479	2075.471
1.495863	1907.282	690.445	334.487	517.025	436.375	148.2937	1.099489	1699.062
-0.9291	1498.215	648.835	172.608	624.047	236.998	318.5292	13.84482	1524.968
3.261434	1879.834	690.801	311.902	627.259	388.914	65.92776	4.091606	1768.229
2.994984	1636.069	511.474	292.264	370.066	332.285	363.2772	6.386463	2184.072
7.99656	1601.338	515.562	279.154	513.384	337.386	388.4849	0.546904	1860.972
0.537049	1707.55	650.433	286.152	619.503	305.673	365.2663	8.606683	2248.623
3.078527	1709.982	751.458	244.056	643.587	367.179	191.9448	3.036096	2134.077
-3.14471	1338.751	410.37	265.597	404.62	271.411	482.474	3.426512	1754.312
2.994984	1636.069	511.474	292.264	370.066	332.285	363.2772	6.386463	2184.072
3.539126	1680.951	554.388	288.569	403.414	299.121	317.2497	11.81392	959.2725
10.2139	1949.674	754.498	311.599	738.447	399.291	126.3232	4.08183	1849.596
1.599203	1874.653	773.233	277.26	704.633	364.567	427.536	23.76717	3358.25
10.3962	1656.738	540.026	292.239	499.881	349.794	288.6407	7.765498	2007.697
3.361176	1757.985	700.142	264.277	591.576	417.158	130.5054	1.396612	1963.494
0.940978	1742.635	716.934	292.411	568.432	345.087	306.2375	0.923596	1703.135
1.599203	1874.653	773.233	277.26	704.633	364.567	427.536	23.76717	3358.25
3.078527	1709.982	751.458	244.056	643.587	367.179	191.9448	3.036096	2134.077
3.019214	1815.915	658.626	332.458	596.949	373.76	107.388	1.836803	1754.523
-2.73457	1690.79	575.959	263.325	529.174	263.325	602.6755	10.18564	2336.094
3.078527	1709.982	751.458	244.056	643.587	367.179	191.9448	3.036096	2134.077
3.654016	1808.103	609.406	326.03	600.122	405.004	69.53417	3.856232	1601.942
10.2139	1949.674	754.498	311.599	738.447	399.291	126.3232	4.08183	1849.596
0.977159	1594.237	664.744	241.42	613.571	280.445	344.4803	2.282428	1975.805
3.269582	1761.512	673.087	252.466	580.927	351.081	186.2722	14.29552	2568.046
3.539126	1680.951	554.388	288.569	403.414	299.121	317.2497	11.81392	959.2725
11.32852	1866.229	735.493	293.782	613.172	407.25	250.0854	6.824211	2129.41
0.977159	1594.237	664.744	241.42	613.571	280.445	344.4803	2.282428	1975.805
3.284632	1766.656	726.812	251.065	605.233	355.336	142.9463	2.576109	1703.397
2.515495	1766.287	753.767	272.141	674.837	333.336	147.7344	2.213931	1994.002
0.977159	1594.237	664.744	241.42	613.571	280.445	344.4803	2.282428	1975.805
3.019214	1815.915	658.626	332.458	596.949	373.76	107.388	1.836803	1754.523
0.089544	1624.298	659.248	248.595	629.329	291.427	363.0986	3.370361	2047.03
9.827434	1638.509	525.536	266.242	525.536	358.351	378.9761	11.98303	2443.236
2.923692	1750.604	725.927	281.575	644.04	339.092	108.9301	0.270718	1853.959
2.92322	1869.494	701.625	331.039	646.369	378.381	118.1115	1.501855	1862.66
0.977159	1594.237	664.744	241.42	613.571	280.445	344.4803	2.282428	1975.805
12.86876	1716.21	581.786	322.215	480.047	430.242	85.72495	2.663779	1867.87
-2.32548	1444.681	448.344	270.455	395.367	270.455	330.781	0.599844	1782.226
3.224808	1725.185	734.304	267.716	605.522	351.814	118.4804	4.569775	1687.026
2.844868	1735.468	596.5	332.033	537.819	364.987	99.84711	3.078826	1649.392
3.078527	1709.982	751.458	244.056	643.587	367.179	191.9448	3.036096	2134.077
3.019214	1815.915	658.626	332.458	596.949	373.76	107.388	1.836803	1754.523

Table S2 - Anonymized database

9.759945	1571.422	502.145	278.226	468.865	346.317	338.9043	2.973717	1697.062
13.3792	1700.819	607.024	323.455	425.48	432.876	9.546456	0.136922	1877.606
9.765038	1726.196	579.005	315.421	579.005	318.305	367.2032	9.372241	1854.878
11.56054	1731.735	653	289.524	551.408	392.258	133.692	0.928783	1841.605
11.56054	1731.735	653	289.524	551.408	392.258	133.692	0.928783	1841.605
7.99656	1601.338	515.562	279.154	513.384	337.386	388.4849	0.546904	1860.972
-0.98347	1482.361	509.542	255.363	437.681	289.916	443.7068	24.45277	9.84658
0.655505	1798.683	851.344	229.906	796.802	302.237	230.9364	4.226843	2008.737
1.599203	1874.653	773.233	277.26	704.633	364.567	427.536	23.76717	3358.25
10.22724	1567.866	501.406	274.268	463.839	342.907	344.3909	7.572275	1738.915
3.191758	1749.745	600.434	315.678	581.92	364.259	71.10771	0.571329	1809.155
2.775	1706.642	622.925	269.626	594.823	367.238	151.6085	3.044361	1695.477
13.3792	1700.819	607.024	323.455	425.48	432.876	9.546456	0.136922	1877.606
1.165121	1758.55	773.716	260.25	620.897	324.493	240.9818	2.35066	1953.851
-2.55621	1415.638	491.994	259.568	371.713	269.072	398.2895	1.172895	1606.425
3.673863	1689.434	533.232	297.784	533.232	348.849	353.818	9.800788	2414.645
-3.14471	1338.751	410.37	265.597	404.62	271.411	482.474	3.426512	1754.312
1.599203	1874.653	773.233	277.26	704.633	364.567	427.536	23.76717	3358.25
11.56054	1731.735	653	289.524	551.408	392.258	133.692	0.928783	1841.605
2.844868	1735.468	596.5	332.033	537.819	364.987	99.84711	3.078826	1649.392
0.693659	1641.955	674.39	234.111	617.163	294.82	211.2159	8.984717	1305.867
1.599203	1874.653	773.233	277.26	704.633	364.567	427.536	23.76717	3358.25
10.11392	1595.562	514.394	277.919	514.394	340.584	327.824	10.42236	2086.291
1.517253	1619.106	647.824	248.946	647.824	267.039	330.4612	5.314943	2050.618
0.893077	1718.605	712.982	287.465	568.043	350.714	345.0285	2.841131	1883.004
9.255143	1650.811	552.766	309.093	552.766	317.585	384.1146	8.195766	2390.127
7.493527	1732.353	675.805	294.739	618.215	369.921	105.9056	4.341443	1633.646
3.254396	1940.243	834.343	252.329	723.289	400.305	297.4856	4.78008	2179.6
0.057346	1641.624	688.454	233.49	652.575	304.641	423.5338	15.46822	1331.014
3.03844	1928.127	868.941	263.871	768.761	345.561	115.4449	3.68327	1941.253
10.2139	1949.674	754.498	311.599	738.447	399.291	126.3232	4.08183	1849.596
12.66181	1742.803	571.654	332.656	408.076	442.982	68.98592	0.954197	1861.581
3.034577	1756.874	629.997	322.861	564.549	354.739	45.35895	9.336276	1008.816
-2.73457	1690.79	575.959	263.325	529.174	263.325	602.6755	10.18564	2336.094
0.977159	1594.237	664.744	241.42	613.571	280.445	344.4803	2.282428	1975.805
0.089544	1624.298	659.248	248.595	629.329	291.427	363.0986	3.370361	2047.03
-0.03574	1728.667	592.558	281.159	459.295	297.488	321.0894	7.302571	1338.034
-1.72355	1358.596	581.878	172.581	523.931	235.866	693.117	3.449864	1538.647
-1.24902	1802.949	615.544	299.283	536.595	299.283	367.5201	5.641701	2122.695
12.69676	1795.836	596.635	330.645	435.48	447.855	87.46478	2.164512	1745.672
10.3962	1656.738	540.026	292.239	499.881	349.794	288.6407	7.765498	2007.697
11.40095	1777.769	639.072	310.748	491.146	400.351	109.0332	0.997501	1804.575
2.994984	1636.069	511.474	292.264	370.066	332.285	363.2772	6.386463	2184.072
3.082264	1841.716	765.463	299.75	657.881	389.15	187.7079	2.024981	2018.113
-1.80219	1829.322	673.068	270.259	598.688	295.698	425.881	16.28463	2612.849
-0.9291	1498.215	648.835	172.608	624.047	236.998	318.5292	13.84482	1524.968
-0.54135	1745.066	603.212	306.222	572.405	308.373	294.5679	3.569889	2092.734
3.270945	1667.127	534.067	293.901	424.25	335.156	334.5434	9.641643	1425.432
3.082264	1841.716	765.463	299.75	657.881	389.15	187.7079	2.024981	2018.113
-1.72355	1358.596	581.878	172.581	523.931	235.866	693.117	3.449864	1538.647

Table S2 - Anonymized database

-0.03574	1728.667	592.558	281.159	459.295	297.488	321.0894	7.302571	1338.034
-2.42617	1767.578	569.778	308.643	515.791	309.057	662.647	3.772194	2229.433
9.674176	1677.047	747.362	233.006	715.846	287.275	500.629	1.819382	1815.314
11.40095	1777.769	639.072	310.748	491.146	400.351	109.0332	0.997501	1804.575
1.108027	1624.28	625.868	268.403	625.868	274.827	369.4496	3.147714	1759.718
1.599203	1874.653	773.233	277.26	704.633	364.567	427.536	23.76717	3358.25
9.674176	1677.047	747.362	233.006	715.846	287.275	500.629	1.819382	1815.314
3.501115	1770.707	617.138	289.172	563.194	308.652	322.9681	4.351334	1582.188
-0.54135	1745.066	603.212	306.222	572.405	308.373	294.5679	3.569889	2092.734
9.674176	1677.047	747.362	233.006	715.846	287.275	500.629	1.819382	1815.314
10.3962	1656.738	540.026	292.239	499.881	349.794	288.6407	7.765498	2007.697
0.977159	1594.237	664.744	241.42	613.571	280.445	344.4803	2.282428	1975.805
2.92322	1869.494	701.625	331.039	646.369	378.381	118.1115	1.501855	1862.66
2.67878	1857.64	776.54	295.668	720.464	376.505	139.6398	0.460311	1805.588
-3.14471	1338.751	410.37	265.597	404.62	271.411	482.474	3.426512	1754.312
3.078527	1709.982	751.458	244.056	643.587	367.179	191.9448	3.036096	2134.077
0.057346	1641.624	688.454	233.49	652.575	304.641	423.5338	15.46822	1331.014
11.32852	1866.229	735.493	293.782	613.172	407.25	250.0854	6.824211	2129.41
0.057346	1641.624	688.454	233.49	652.575	304.641	423.5338	15.46822	1331.014
3.269582	1761.512	673.087	252.466	580.927	351.081	186.2722	14.29552	2568.046
3.270945	1667.127	534.067	293.901	424.25	335.156	334.5434	9.641643	1425.432
3.269582	1761.512	673.087	252.466	580.927	351.081	186.2722	14.29552	2568.046
2.966132	1738.862	720.904	276.289	636.563	334.732	153.333	4.875383	2212.881
-0.8633	1752.065	718.955	260.487	570.262	362.782	302.8407	5.463532	1223.193
9.712044	1732.498	585.667	311.317	585.667	315.814	368.1078	3.085942	1998.603
-3.14471	1338.751	410.37	265.597	404.62	271.411	482.474	3.426512	1754.312
1.778709	1995.365	772.682	326.285	701.46	438.144	177.847	2.773011	1576.339
3.078527	1709.982	751.458	244.056	643.587	367.179	191.9448	3.036096	2134.077
3.09322	1645.044	511.449	294.596	395.47	329.005	311.3914	3.386594	1645.39
1.980445	1420.531	451.953	284.108	376.634	292.057	415.1788	7.663112	2302.833
2.923692	1750.604	725.927	281.575	644.04	339.092	108.9301	0.270718	1853.959
-0.9291	1498.215	648.835	172.608	624.047	236.998	318.5292	13.84482	1524.968
13.06402	1796.584	616.989	330.372	463.305	444.109	67.57873	4.980977	1515.009
7.75183	1744.123	686.263	290.613	624.057	366.899	96.70383	4.908966	1559.2
-2.48813	1431.669	416.868	267.291	405.63	267.291	379.0812	2.998592	1846.541
9.255143	1650.811	552.766	309.093	552.766	317.585	384.1146	8.195766	2390.127
2.99339	1736.133	670.84	291.367	566.046	393.127	178.8689	3.350492	1871.85
-3.14471	1338.751	410.37	265.597	404.62	271.411	482.474	3.426512	1754.312
1.495863	1907.282	690.445	334.487	517.025	436.375	148.2937	1.099489	1699.062
0.977159	1594.237	664.744	241.42	613.571	280.445	344.4803	2.282428	1975.805
-0.98347	1482.361	509.542	255.363	437.681	289.916	443.7068	24.45277	9.84658
7.493527	1732.353	675.805	294.739	618.215	369.921	105.9056	4.341443	1633.646
3.034577	1756.874	629.997	322.861	564.549	354.739	45.35895	9.336276	1008.816
3.224808	1725.185	734.304	267.716	605.522	351.814	118.4804	4.569775	1687.026
9.827434	1638.509	525.536	266.242	525.536	358.351	378.9761	11.98303	2443.236
10.3962	1656.738	540.026	292.239	499.881	349.794	288.6407	7.765498	2007.697
-0.9291	1498.215	648.835	172.608	624.047	236.998	318.5292	13.84482	1524.968
-0.8633	1752.065	718.955	260.487	570.262	362.782	302.8407	5.463532	1223.193
8.484687	1566.905	478.142	281.648	399.884	348.375	344.1273	8.791059	2300.894
1.517253	1619.106	647.824	248.946	647.824	267.039	330.4612	5.314943	2050.618

Table S2 - Anonymized database

9.255143	1650.811	552.766	309.093	552.766	317.585	384.1146	8.195766	2390.127
10.09323	1722.892	566.342	274.754	566.342	353.188	272.5588	7.12005	2301.675
12.19463	1690.914	540.209	315.174	420.553	427.2	117.4134	4.922527	1843.187
2.658973	1857.823	804.853	282.122	769.688	368.375	163.24	0.627206	1907.193
11.64161	1855.697	761.546	284.142	679.693	395.206	119.5475	4.079675	1512.646
3.019214	1815.915	658.626	332.458	596.949	373.76	62.67819	6.543175	1760.891
3.269582	1761.512	673.087	252.466	580.927	351.081	186.2722	14.29552	2568.046
-2.48813	1431.669	416.868	267.291	405.63	267.291	379.0812	2.998592	1846.541
10.11392	1595.562	514.394	277.919	514.394	340.584	327.824	10.42236	2086.291
3.165027	1786.812	722.962	244.536	616.051	363.824	176.3364	1.888108	1919.367
3.366198	1708.992	522.547	336.275	466.573	407.951	98.54894	0.803851	1818.95
1.068066	1644.372	676.736	255.604	540.952	328.193	187.6075	5.691956	1343.706
-0.8633	1752.065	718.955	260.487	570.262	362.782	302.8407	5.463532	1223.193
3.224808	1725.185	734.304	267.716	605.522	351.814	118.4804	4.569775	1687.026
2.966132	1738.862	720.904	276.289	636.563	334.732	153.333	4.875383	2212.881
9.759945	1571.422	502.145	278.226	468.865	346.317	338.9043	2.973717	1697.062
-3.14471	1338.751	410.37	265.597	404.62	271.411	482.474	3.426512	1754.312
10.22724	1567.866	501.406	274.268	463.839	342.907	344.3909	7.572275	1738.915
3.702681	1741.133	640.872	271.713	560.119	436.985	100.9892	8.350395	2369.271
-1.72355	1358.596	581.878	172.581	523.931	235.866	693.117	3.449864	1538.647
3.312335	1748.048	605.137	330.629	521.998	401.257	121.9577	2.032796	1891.805
11.32852	1866.229	735.493	293.782	613.172	407.25	250.0854	6.824211	2129.41
2.844868	1735.468	596.5	332.033	537.819	364.987	99.84711	3.078826	1649.392
2.793099	1680.525	640.494	253.498	580.599	350.649	214.722	3.836843	1629.528
10.12959	1638.506	522.081	270.148	522.081	357.382	313.4054	11.75299	1418.11
0.977159	1594.237	664.744	241.42	613.571	280.445	344.4803	2.282428	1975.805
-2.64582	1464.559	458.745	257.359	414.643	257.359	411.3077	1.967308	1620.276
3.539126	1680.951	554.388	288.569	403.414	299.121	317.2497	11.81392	959.2725
7.99656	1601.338	515.562	279.154	513.384	337.386	388.4849	0.546904	1860.972
2.994984	1636.069	511.474	292.264	370.066	332.285	363.2772	6.386463	2184.072
11.56054	1731.735	653	289.524	551.408	392.258	133.692	0.928783	1841.605
2.67878	1857.64	776.54	295.668	720.464	376.505	139.6398	0.460311	1805.588
9.255143	1650.811	552.766	309.093	552.766	317.585	384.1146	8.195766	2390.127
-1.80219	1829.322	673.068	270.259	598.688	295.698	425.881	16.28463	2612.849
11.32852	1866.229	735.493	293.782	613.172	407.25	250.0854	6.824211	2129.41
2.99339	1736.133	670.84	291.367	566.046	393.127	178.8689	3.350492	1871.85
-0.98347	1482.361	509.542	255.363	437.681	289.916	443.7068	24.45277	9.84658
0.977159	1594.237	664.744	241.42	613.571	280.445	344.4803	2.282428	1975.805
2.99339	1736.133	670.84	291.367	566.046	393.127	178.8689	3.350492	1871.85
-0.8633	1752.065	718.955	260.487	570.262	362.782	302.8407	5.463532	1223.193
-1.72355	1358.596	581.878	172.581	523.931	235.866	693.117	3.449864	1538.647
2.844868	1735.468	596.5	332.033	537.819	364.987	99.84711	3.078826	1649.392
3.082264	1841.716	765.463	299.75	657.881	389.15	187.7079	2.024981	2018.113
3.501115	1770.707	617.138	289.172	563.194	308.652	322.9681	4.351334	1582.188
7.493527	1732.353	675.805	294.739	618.215	369.921	105.9056	4.341443	1633.646
3.03844	1928.127	868.941	263.871	768.761	345.561	115.4449	3.68327	1941.253
3.361176	1757.985	700.142	264.277	591.576	417.158	130.5054	1.396612	1963.494
2.045709	1716.124	701.845	242.792	614.784	306.751	288.6531	13.5198	1038.656
-1.72355	1358.596	581.878	172.581	523.931	235.866	693.117	3.449864	1538.647
3.224808	1725.185	734.304	267.716	605.522	351.814	118.4804	4.569775	1687.026

Table S2 - Anonymized database

2.844868	1735.468	596.5	332.033	537.819	364.987	99.84711	3.078826	1649.392
-0.1882	1614.638	664.352	245.667	619.388	295.482	380.8375	4.070759	1519.568
0.940978	1742.635	716.934	292.411	568.432	345.087	306.2375	0.923596	1703.135
-0.9291	1498.215	648.835	172.608	624.047	236.998	318.5292	13.84482	1524.968
3.494104	1713.474	552.081	335.551	431.305	415.616	110.0355	1.325417	1893.37
3.078527	1709.982	751.458	244.056	643.587	367.179	191.9448	3.036096	2134.077
1.599203	1874.653	773.233	277.26	704.633	364.567	427.536	23.76717	3358.25
3.078527	1709.982	751.458	244.056	643.587	367.179	191.9448	3.036096	2134.077
3.254396	1940.243	834.343	252.329	723.289	400.305	297.4856	4.78008	2179.6
-2.80929	1344.204	397.487	267.195	338.895	267.195	409.062	9.06532	2102.892
3.019214	1815.915	658.626	332.458	596.949	373.76	62.67819	6.543175	1760.891
2.67878	1857.64	776.54	295.668	720.464	376.505	139.6398	0.460311	1805.588
3.494104	1713.474	552.081	335.551	431.305	415.616	110.0355	1.325417	1893.37
3.539126	1680.951	554.388	288.569	403.414	299.121	317.2497	11.81392	959.2725
3.224808	1725.185	734.304	267.716	605.522	351.814	118.4804	4.569775	1687.026
-3.14471	1338.751	410.37	265.597	404.62	271.411	482.474	3.426512	1754.312
1.599203	1874.653	773.233	277.26	704.633	364.567	427.536	23.76717	3358.25
0.977159	1594.237	664.744	241.42	613.571	280.445	344.4803	2.282428	1975.805
0.655505	1798.683	851.344	229.906	796.802	302.237	230.9364	4.226843	2008.737
11.32852	1866.229	735.493	293.782	613.172	407.25	250.0854	6.824211	2129.41
10.11392	1595.562	514.394	277.919	514.394	340.584	327.824	10.42236	2086.291
2.844868	1735.468	596.5	332.033	537.819	364.987	99.84711	3.078826	1649.392
2.844868	1735.468	596.5	332.033	537.819	364.987	99.84711	3.078826	1649.392
2.844868	1735.468	596.5	332.033	537.819	364.987	99.84711	3.078826	1649.392
3.539126	1680.951	554.388	288.569	403.414	299.121	317.2497	11.81392	959.2725
0.914473	1762.075	769.288	249.294	630.181	304.338	195.607	12.72143	1135.702
3.284632	1766.656	726.812	251.065	605.233	355.336	142.9463	2.576109	1703.397
2.775	1706.642	622.925	269.626	594.823	367.238	151.6085	3.044361	1695.477
12.69676	1795.836	596.635	330.645	435.48	447.855	87.46478	2.164512	1745.672
0.977159	1594.237	664.744	241.42	613.571	280.445	344.4803	2.282428	1975.805
3.019214	1815.915	658.626	332.458	596.949	373.76	62.67819	6.543175	1760.891
2.515495	1766.287	753.767	272.141	674.837	333.336	147.7344	2.213931	1994.002
3.254396	1940.243	834.343	252.329	723.289	400.305	297.4856	4.78008	2179.6
1.527967	1593.369	653.504	239.989	652.947	263.645	222.4361	2.733181	1472.043
-0.8633	1752.065	718.955	260.487	570.262	362.782	302.8407	5.463532	1223.193
3.078527	1709.982	751.458	244.056	643.587	367.179	191.9448	3.036096	2134.077
1.527967	1593.369	653.504	239.989	652.947	263.645	222.4361	2.733181	1472.043
3.402511	1801.875	765.158	242.219	652.101	377.846	184.527	4.803395	1531.5
3.673863	1689.434	533.232	297.784	533.232	348.849	353.818	9.800788	2414.645
-0.41771	1550.417	578.046	232.753	534.165	284.639	200.8394	0.870624	920.6934
3.254396	1940.243	834.343	252.329	723.289	400.305	297.4856	4.78008	2179.6
3.078527	1709.982	751.458	244.056	643.587	367.179	191.9448	3.036096	2134.077
10.11392	1595.562	514.394	277.919	514.394	340.584	327.824	10.42236	2086.291
-1.72355	1358.596	581.878	172.581	523.931	235.866	693.117	3.449864	1538.647
2.98006	1712.541	647.985	294.939	584.4	364.177	122.264	6.164512	2312.941
1.527967	1593.369	653.504	239.989	652.947	263.645	222.4361	2.733181	1472.043
1.165121	1758.55	773.716	260.25	620.897	324.493	240.9818	2.35066	1953.851
-2.0658	1737.211	552.653	308.083	492.523	308.083	559.32	6.887537	1658.008
2.861005	1876.506	770.783	302.223	712.595	376.309	131.5466	7.098525	1302.395
2.67878	1857.64	776.54	295.668	720.464	376.505	139.6398	0.460311	1805.588

Table S2 - Anonymized database

11.32852	1866.229	735.493	293.782	613.172	407.25	250.0854	6.824211	2129.41
10.22724	1567.866	501.406	274.268	463.839	342.907	344.3909	7.572275	1738.915
7.493527	1732.353	675.805	294.739	618.215	369.921	105.9056	4.341443	1633.646
-0.8633	1752.065	718.955	260.487	570.262	362.782	302.8407	5.463532	1223.193
3.361176	1757.985	700.142	264.277	591.576	417.158	130.5054	1.396612	1963.494
3.186462	1749.97	683.881	258.535	628.119	343.95	174.5617	0.468577	1840.082
3.312335	1748.048	605.137	330.629	521.998	401.257	121.9577	2.032796	1891.805
0.693659	1641.955	674.39	234.111	617.163	294.82	211.2159	8.984717	1305.867
3.202247	1743.849	671.584	293.122	576.912	397.75	146.1161	0.880539	1819.605
3.38656	1671.116	534.958	296.157	388.283	341.02	305.1122	1.098116	1929.252
11.32852	1866.229	735.493	293.782	613.172	407.25	250.0854	6.824211	2129.41
3.284632	1766.656	726.812	251.065	605.233	355.336	142.9463	2.576109	1703.397
4.061687	1767.573	553.444	337.394	553.444	410.382	52.20359	5.166937	1447.064
3.078527	1709.982	751.458	244.056	643.587	367.179	191.9448	3.036096	2134.077
0.977159	1594.237	664.744	241.42	613.571	280.445	344.4803	2.282428	1975.805
12.39011	1694.763	534.732	335.899	398.526	429.408	83.90097	2.566	2002.437
1.369231	1606.278	649.244	245.354	642.337	269.913	301.849	15.21554	2029.688
2.923692	1750.604	725.927	281.575	644.04	339.092	108.9301	0.270718	1853.959
2.658973	1857.823	804.853	282.122	769.688	368.375	163.24	0.627206	1907.193
1.165121	1758.55	773.716	260.25	620.897	324.493	240.9818	2.35066	1953.851
4.061687	1767.573	553.444	337.394	553.444	410.382	52.20359	5.166937	1447.064
-3.14471	1338.751	410.37	265.597	404.62	271.411	482.474	3.426512	1754.312
-2.13416	1758.907	557.63	287.94	533.806	287.94	578.7245	15.7686	1843.488
11.77305	1798.059	729.666	298.193	624.238	390.883	158.5528	2.500618	2028.029
0.977159	1594.237	664.744	241.42	613.571	280.445	344.4803	2.282428	1975.805
10.3962	1656.738	540.026	292.239	499.881	349.794	288.6407	7.765498	2007.697
0.893077	1718.605	712.982	287.465	568.043	350.714	345.0285	2.841131	1883.004
-2.42617	1767.578	569.778	308.643	515.791	309.057	662.647	3.772194	2229.433
3.09322	1645.044	511.449	294.596	395.47	329.005	311.3914	3.386594	1645.39
3.673863	1689.434	533.232	297.784	533.232	348.849	353.818	9.800788	2414.645
1.599203	1874.653	773.233	277.26	704.633	364.567	427.536	23.76717	3358.25
11.32852	1866.229	735.493	293.782	613.172	407.25	250.0854	6.824211	2129.41
2.739203	1760.081	718.746	280.373	654.203	347.181	162.8161	1.964231	1741.297
12.79818	1775.993	583.92	319.59	490.019	439.791	91.46474	1.612379	1995.601
1.599203	1874.653	773.233	277.26	704.633	364.567	427.536	23.76717	3358.25
-2.13416	1758.907	557.63	287.94	533.806	287.94	578.7245	15.7686	1843.488
2.67878	1857.64	776.54	295.668	720.464	376.505	139.6398	0.460311	1805.588
3.165027	1786.812	722.962	244.536	616.051	363.824	176.3364	1.888108	1919.367
12.19463	1690.914	540.209	315.174	420.553	427.2	117.4134	4.922527	1843.187
11.32852	1866.229	735.493	293.782	613.172	407.25	250.0854	6.824211	2129.41
3.078527	1709.982	751.458	244.056	643.587	367.179	191.9448	3.036096	2134.077
2.92322	1869.494	701.625	331.039	646.369	378.381	118.1115	1.501855	1862.66
0.977159	1594.237	664.744	241.42	613.571	280.445	344.4803	2.282428	1975.805
0.914473	1762.075	769.288	249.294	630.181	304.338	195.607	12.72143	1135.702
-1.72355	1358.596	581.878	172.581	523.931	235.866	693.117	3.449864	1538.647
3.082264	1841.716	765.463	299.75	657.881	389.15	187.7079	2.024981	2018.113
-0.54135	1745.066	603.212	306.222	572.405	308.373	294.5679	3.569889	2092.734
0.655505	1798.683	851.344	229.906	796.802	302.237	230.9364	4.226843	2008.737
-0.03574	1728.667	592.558	281.159	459.295	297.488	321.0894	7.302571	1338.034
3.270945	1667.127	534.067	293.901	424.25	335.156	334.5434	9.641643	1425.432

Table S2 - Anonymized database

0.977159	1594.237	664.744	241.42	613.571	280.445	344.4803	2.282428	1975.805
12.67234	1722.314	565.458	330.413	405.641	440.329	54.76521	5.173323	1836.214
3.254396	1940.243	834.343	252.329	723.289	400.305	297.4856	4.78008	2179.6
2.739203	1760.081	718.746	280.373	654.203	347.181	162.8161	1.964231	1741.297
-2.73457	1690.79	575.959	263.325	529.174	263.325	602.6755	10.18564	2336.094
3.284632	1766.656	726.812	251.065	605.233	355.336	142.9463	2.576109	1703.397
3.078527	1709.982	751.458	244.056	643.587	367.179	191.9448	3.036096	2134.077
0.446736	2007.881	732.491	340.365	659.08	457.163	311.8601	0.561861	1807.235
3.078527	1709.982	751.458	244.056	643.587	367.179	191.9448	3.036096	2134.077
2.994984	1636.069	511.474	292.264	370.066	332.285	363.2772	6.386463	2184.072
10.61508	1932.18	718.592	325.626	676.148	436.192	188.1355	5.151401	2020.188
2.309527	1759.552	665.196	297.839	608.559	382.53	231.4455	5.869617	2064.344
1.36717	1631.658	686.213	240.917	561.508	319.077	161.0517	3.782925	1557.11
-2.32548	1444.681	448.344	270.455	395.367	270.455	330.781	0.599844	1782.226
1.599203	1874.653	773.233	277.26	704.633	364.567	427.536	23.76717	3358.25
0.057346	1641.624	688.454	233.49	652.575	304.641	423.5338	15.46822	1331.014
2.515495	1766.287	753.767	272.141	674.837	333.336	147.7344	2.213931	1994.002
-2.15702	1375.056	425.211	233.701	379.362	233.701	347.2733	4.467479	2075.471
-1.80219	1829.322	673.068	270.259	598.688	295.698	425.881	16.28463	2612.849
3.284632	1766.656	726.812	251.065	605.233	355.336	142.9463	2.576109	1703.397
3.269582	1761.512	673.087	252.466	580.927	351.081	186.2722	14.29552	2568.046
3.078527	1709.982	751.458	244.056	643.587	367.179	191.9448	3.036096	2134.077
2.515495	1766.287	753.767	272.141	674.837	333.336	147.7344	2.213931	1994.002
3.224808	1725.185	734.304	267.716	605.522	351.814	118.4804	4.569775	1687.026
12.07976	1683.682	592.625	305.189	507.767	402.879	92.29993	9.324022	1126.678
-0.9291	1498.215	648.835	172.608	624.047	236.998	318.5292	13.84482	1524.968
2.844868	1735.468	596.5	332.033	537.819	364.987	99.84711	3.078826	1649.392
1.068066	1644.372	676.736	255.604	540.952	328.193	187.6075	5.691956	1343.706
1.527967	1593.369	653.504	239.989	652.947	263.645	222.4361	2.733181	1472.043
3.366198	1708.992	522.547	336.275	466.573	407.951	98.54894	0.803851	1818.95
-0.41771	1550.417	578.046	232.753	534.165	284.639	200.8394	0.870624	920.6934
11.56054	1731.735	653	289.524	551.408	392.258	133.692	0.928783	1841.605
2.658973	1857.823	804.853	282.122	769.688	368.375	163.24	0.627206	1907.193
-2.66538	1472.877	466.147	269.666	425.297	269.666	385.9416	16.94996	446.4642
7.99656	1601.338	515.562	279.154	513.384	337.386	388.4849	0.546904	1860.972
-1.72355	1358.596	581.878	172.581	523.931	235.866	693.117	3.449864	1538.647
2.739203	1760.081	718.746	280.373	654.203	347.181	162.8161	1.964231	1741.297
-2.42617	1767.578	569.778	308.643	515.791	309.057	662.647	3.772194	2229.433
-3.14471	1338.751	410.37	265.597	404.62	271.411	482.474	3.426512	1754.312
-1.80219	1829.322	673.068	270.259	598.688	295.698	425.881	16.28463	2612.849
-2.80929	1344.204	397.487	267.195	338.895	267.195	409.062	9.06532	2102.892
3.673863	1689.434	533.232	297.784	533.232	348.849	353.818	9.800788	2414.645
3.09322	1645.044	511.449	294.596	395.47	329.005	311.3914	3.386594	1645.39
0.655505	1798.683	851.344	229.906	796.802	302.237	230.9364	4.226843	2008.737
2.994984	1636.069	511.474	292.264	370.066	332.285	363.2772	6.386463	2184.072
0.977159	1594.237	664.744	241.42	613.571	280.445	344.4803	2.282428	1975.805
11.38651	1863.632	727.8	297.391	628.303	411.921	119.6027	1.839919	1925.151
0.977159	1594.237	664.744	241.42	613.571	280.445	344.4803	2.282428	1975.805
2.844868	1735.468	596.5	332.033	537.819	364.987	99.84711	3.078826	1649.392
-1.72355	1358.596	581.878	172.581	523.931	235.866	693.117	3.449864	1538.647

Table S2 - Anonymized database

3.284632	1766.656	726.812	251.065	605.233	355.336	142.9463	2.576109	1703.397
1.165121	1758.55	773.716	260.25	620.897	324.493	240.9818	2.35066	1953.851
7.99656	1601.338	515.562	279.154	513.384	337.386	388.4849	0.546904	1860.972
10.3962	1656.738	540.026	292.239	499.881	349.794	288.6407	7.765498	2007.697
0.089544	1624.298	659.248	248.595	629.329	291.427	363.0986	3.370361	2047.03
0.977159	1594.237	664.744	241.42	613.571	280.445	344.4803	2.282428	1975.805
3.019214	1815.915	658.626	332.458	596.949	373.76	62.67819	6.543175	1760.891
2.515495	1766.287	753.767	272.141	674.837	333.336	147.7344	2.213931	1994.002
3.170423	1667.742	687.202	246.772	575.913	321.377	125.6447	1.431671	1880.36
3.261434	1879.834	690.801	311.902	627.259	388.914	65.92776	4.091606	1768.229
-3.14471	1338.751	410.37	265.597	404.62	271.411	482.474	3.426512	1754.312
-3.14471	1338.751	410.37	265.597	404.62	271.411	482.474	3.426512	1754.312
0.537049	1707.55	650.433	286.152	619.503	305.673	365.2663	8.606683	2248.623
0.977159	1594.237	664.744	241.42	613.571	280.445	344.4803	2.282428	1975.805
1.165121	1758.55	773.716	260.25	620.897	324.493	240.9818	2.35066	1953.851
-2.46074	1373.009	422.337	249.857	365.846	249.857	396.1142	1.802708	1720.296
3.673863	1689.434	533.232	297.784	533.232	348.849	353.818	9.800788	2414.645
9.255143	1650.811	552.766	309.093	552.766	317.585	384.1146	8.195766	2390.127
-3.14471	1338.751	410.37	265.597	404.62	271.411	482.474	3.426512	1754.312
-1.72355	1358.596	581.878	172.581	523.931	235.866	693.117	3.449864	1538.647
10.09323	1722.892	566.342	274.754	566.342	353.188	272.5588	7.12005	2301.675
1.068066	1644.372	676.736	255.604	540.952	328.193	187.6075	5.691956	1343.706
4.306681	1669.886	546.443	290.398	461.013	447.037	28.46955	0.679182	1846.094
7.493527	1732.353	675.805	294.739	618.215	369.921	105.9056	4.341443	1633.646
-1.55404	1655.204	707.488	235.803	692.561	285.481	489.7062	3.59755	1702.217
10.22724	1567.866	501.406	274.268	463.839	342.907	344.3909	7.572275	1738.915
-0.03574	1728.667	592.558	281.159	459.295	297.488	321.0894	7.302571	1338.034
2.045709	1716.124	701.845	242.792	614.784	306.751	288.6531	13.5198	1038.656
11.56054	1731.735	653	289.524	551.408	392.258	133.692	0.928783	1841.605
9.255143	1650.811	552.766	309.093	552.766	317.585	384.1146	8.195766	2390.127
3.191758	1749.745	600.434	315.678	581.92	364.259	71.10771	0.571329	1809.155
10.2139	1949.674	754.498	311.599	738.447	399.291	126.3232	4.08183	1849.596
3.270945	1667.127	534.067	293.901	424.25	335.156	334.5434	9.641643	1425.432
12.79818	1775.993	583.92	319.59	490.019	439.791	91.46474	1.612379	1995.601
0.089544	1624.298	659.248	248.595	629.329	291.427	367.653	6.092455	2268.084
2.67878	1857.64	776.54	295.668	720.464	376.505	139.6398	0.460311	1805.588
-0.9291	1498.215	648.835	172.608	624.047	236.998	318.5292	13.84482	1524.968
-2.66538	1472.877	466.147	269.666	425.297	269.666	385.9416	16.94996	446.4642
9.674176	1677.047	747.362	233.006	715.846	287.275	500.629	1.819382	1815.314
-3.14471	1338.751	410.37	265.597	404.62	271.411	482.474	3.426512	1754.312
11.32852	1866.229	735.493	293.782	613.172	407.25	250.0854	6.824211	2129.41
3.284632	1766.656	726.812	251.065	605.233	355.336	142.9463	2.576109	1703.397
-0.8633	1752.065	718.955	260.487	570.262	362.782	302.8407	5.463532	1223.193
10.3962	1656.738	540.026	292.239	499.881	349.794	288.6407	7.765498	2007.697
-0.54135	1745.066	603.212	306.222	572.405	308.373	294.5679	3.569889	2092.734
3.539126	1680.951	554.388	288.569	403.414	299.121	317.2497	11.81392	959.2725
3.165027	1786.812	722.962	244.536	616.051	363.824	176.3364	1.888108	1919.367
3.494104	1713.474	552.081	335.551	431.305	415.616	110.0355	1.325417	1893.37
0.914473	1762.075	769.288	249.294	630.181	304.338	195.607	12.72143	1135.702
2.861005	1876.506	770.783	302.223	712.595	376.309	131.5466	7.098525	1302.395

Table S2 - Anonymized database

3.654016	1808.103	609.406	326.03	600.122	405.004	69.53417	3.856232	1601.942
11.32852	1866.229	735.493	293.782	613.172	407.25	250.0854	6.824211	2129.41
-2.73457	1690.79	575.959	263.325	529.174	263.325	602.6755	10.18564	2336.094
10.22724	1567.866	501.406	274.268	463.839	342.907	344.3909	7.572275	1738.915
2.844868	1735.468	596.5	332.033	537.819	364.987	99.84711	3.078826	1649.392
10.3962	1656.738	540.026	292.239	499.881	349.794	288.6407	7.765498	2007.697
11.32852	1866.229	735.493	293.782	613.172	407.25	250.0854	6.824211	2129.41
10.11392	1595.562	514.394	277.919	514.394	340.584	327.824	10.42236	2086.291
-3.14471	1338.751	410.37	265.597	404.62	271.411	482.474	3.426512	1754.312
-1.80219	1829.322	673.068	270.259	598.688	295.698	425.881	16.28463	2612.849
2.92322	1869.494	701.625	331.039	646.369	378.381	118.1115	1.501855	1862.66
2.515495	1766.287	753.767	272.141	674.837	333.336	147.7344	2.213931	1994.002
-0.98347	1482.361	509.542	255.363	437.681	289.916	443.7068	24.45277	9.84658
-0.9291	1498.215	648.835	172.608	624.047	236.998	318.5292	13.84482	1524.968
1.527967	1593.369	653.504	239.989	652.947	263.645	222.4361	2.733181	1472.043
2.67878	1857.64	776.54	295.668	720.464	376.505	139.6398	0.460311	1805.588
8.429346	1519.842	463.864	279.723	375.305	336.653	365.6075	12.60653	1907.473
12.79818	1775.993	583.92	319.59	490.019	439.791	91.46474	1.612379	1995.601
0.057346	1641.624	688.454	233.49	652.575	304.641	423.5338	15.46822	1331.014
9.674176	1677.047	747.362	233.006	715.846	287.275	500.629	1.819382	1815.314
1.165121	1758.55	773.716	260.25	620.897	324.493	240.9818	2.35066	1953.851
3.270945	1667.127	534.067	293.901	424.25	335.156	345.6854	12.32941	1844.389
2.67878	1857.64	776.54	295.668	720.464	376.505	139.6398	0.460311	1805.588
-2.55621	1415.638	491.994	259.568	371.713	269.072	398.2895	1.172895	1606.425
1.108027	1624.28	625.868	268.403	625.868	274.827	369.4496	3.147714	1759.718
0.977159	1594.237	664.744	241.42	613.571	280.445	344.4803	2.282428	1975.805
11.32852	1866.229	735.493	293.782	613.172	407.25	250.0854	6.824211	2129.41
3.019214	1815.915	658.626	332.458	596.949	373.76	62.67819	6.543175	1760.891
3.261434	1879.834	690.801	311.902	627.259	388.914	65.92776	4.091606	1768.229
2.658973	1857.823	804.853	282.122	769.688	368.375	163.24	0.627206	1907.193
-3.14471	1338.751	410.37	265.597	404.62	271.411	482.474	3.426512	1754.312
9.674176	1677.047	747.362	233.006	715.846	287.275	500.629	1.819382	1815.314
10.2139	1949.674	754.498	311.599	738.447	399.291	126.3232	4.08183	1849.596
-1.55404	1655.204	707.488	235.803	692.561	285.481	489.7062	3.59755	1702.217
-0.8633	1752.065	718.955	260.487	570.262	362.782	302.8407	5.463532	1223.193
3.361176	1757.985	700.142	264.277	591.576	417.158	130.5054	1.396612	1963.494
2.739203	1760.081	718.746	280.373	654.203	347.181	162.8161	1.964231	1741.297
3.402511	1801.875	765.158	242.219	652.101	377.846	184.527	4.803395	1531.5
11.32852	1866.229	735.493	293.782	613.172	407.25	250.0854	6.824211	2129.41
0.940978	1742.635	716.934	292.411	568.432	345.087	306.2375	0.923596	1703.135
-0.8633	1752.065	718.955	260.487	570.262	362.782	302.8407	5.463532	1223.193
-1.72355	1358.596	581.878	172.581	523.931	235.866	693.117	3.449864	1538.647
-2.73457	1690.79	575.959	263.325	529.174	263.325	602.6755	10.18564	2336.094
12.79818	1775.993	583.92	319.59	490.019	439.791	91.46474	1.612379	1995.601
-0.41771	1550.417	578.046	232.753	534.165	284.639	200.8394	0.870624	920.6934
3.366198	1708.992	522.547	336.275	466.573	407.951	98.54894	0.803851	1818.95
13.3792	1700.819	607.024	323.455	425.48	432.876	9.546456	0.136922	1877.606
12.79818	1775.993	583.92	319.59	490.019	439.791	91.46474	1.612379	1995.601
10.61508	1932.18	718.592	325.626	676.148	436.192	188.1355	5.151401	2020.188
3.224808	1725.185	734.304	267.716	605.522	351.814	118.4804	4.569775	1687.026

Table S2 - Anonymized database

2.309527	1759.552	665.196	297.839	608.559	382.53	231.4455	5.869617	2064.344
-2.66538	1472.877	466.147	269.666	425.297	269.666	385.9416	16.94996	446.4642
0.977159	1594.237	664.744	241.42	613.571	280.445	344.4803	2.282428	1975.805
9.712044	1732.498	585.667	311.317	585.667	315.814	368.1078	3.085942	1998.603
2.045709	1716.124	701.845	242.792	614.784	306.751	288.6531	13.5198	1038.656
12.39011	1694.763	534.732	335.899	398.526	429.408	83.90097	2.566	2002.437
9.255143	1650.811	552.766	309.093	552.766	317.585	384.1146	8.195766	2390.127
-0.54135	1745.066	603.212	306.222	572.405	308.373	294.5679	3.569889	2092.734
3.03844	1928.127	868.941	263.871	768.761	345.561	115.4449	3.68327	1941.253
-0.03574	1728.667	592.558	281.159	459.295	297.488	321.0894	7.302571	1338.034
11.64161	1855.697	761.546	284.142	679.693	395.206	119.5475	4.079675	1512.646
3.03844	1928.127	868.941	263.871	768.761	345.561	115.4449	3.68327	1941.253
0.057346	1641.624	688.454	233.49	652.575	304.641	423.5338	15.46822	1331.014
1.495863	1907.282	690.445	334.487	517.025	436.375	148.2937	1.099489	1699.062
-2.13416	1758.907	557.63	287.94	533.806	287.94	578.7245	15.7686	1843.488
9.255143	1650.811	552.766	309.093	552.766	317.585	384.1146	8.195766	2390.127
10.22724	1567.866	501.406	274.268	463.839	342.907	344.3909	7.572275	1738.915
11.40095	1777.769	639.072	310.748	491.146	400.351	109.0332	0.997501	1804.575
10.3962	1656.738	540.026	292.239	499.881	349.794	288.6407	7.765498	2007.697
3.078527	1709.982	751.458	244.056	643.587	367.179	191.9448	3.036096	2134.077
3.03844	1928.127	868.941	263.871	768.761	345.561	115.4449	3.68327	1941.253
-2.0658	1737.211	552.653	308.083	492.523	308.083	559.32	6.887537	1658.008
9.712044	1732.498	585.667	311.317	585.667	315.814	368.1078	3.085942	1998.603
7.493527	1732.353	675.805	294.739	618.215	369.921	105.9056	4.341443	1633.646
-2.73457	1690.79	575.959	263.325	529.174	263.325	602.6755	10.18564	2336.094
7.99656	1601.338	515.562	279.154	513.384	337.386	388.4849	0.546904	1860.972
0.655505	1798.683	851.344	229.906	796.802	302.237	230.9364	4.226843	2008.737
12.79818	1775.993	583.92	319.59	490.019	439.791	91.46474	1.612379	1995.601
1.599203	1874.653	773.233	277.26	704.633	364.567	427.536	23.76717	3358.25
12.07976	1683.682	592.625	305.189	507.767	402.879	92.29993	9.324022	1126.678

Table S2 - Anonymized database

pcurv	tcurv	twi	EW	NS	ITL	FRI	exp_yrs	col_nov
5.80E-07	-0.00012	10.36644	0.996778	-0.08021	0.002177	0.390538	Over 10 ye	7
5.80E-07	-0.00012	10.36644	0.996778	-0.08021	0.002177	0.390538	Over 10 ye	7
-0.00062	0.002157	6.203242	0.859966	0.510351	0.005228	0.388217	2 to 5 year	25
-0.00062	0.002157	6.203242	0.859966	0.510351	0.005228	0.388217	2 to 5 year	25
-0.00062	0.002157	6.203242	0.859966	0.510351	0.005228	0.388217	2 to 5 year	25
-0.00062	0.002157	6.203242	0.859966	0.510351	0.005228	0.388217	2 to 5 year	25
-0.00062	0.002157	6.203242	0.859966	0.510351	0.005228	0.388217	2 to 5 year	25
-0.00062	0.002157	6.203242	0.859966	0.510351	0.005228	0.388217	2 to 5 year	25
-0.00062	0.002157	6.203242	0.859966	0.510351	0.005228	0.388217	2 to 5 year	25
0.002836	-0.00084	7.157466	-0.5676	-0.8233	0.001367	0.396477	Over 10 ye	2
-0.00064	-0.00037	8.810884	0.159075	-0.98727	0.017465	0.406714	2 to 5 year	8
0.002679	0.000302	7.512269	0.558958	-0.8292	0.002882	0.388022	2 to 5 year	8
0.002679	0.000302	7.512269	0.558958	-0.8292	0.002882	0.388022	2 to 5 year	8
0.002679	0.000302	7.512269	0.558958	-0.8292	0.002882	0.388022	2 to 5 year	8
0.002679	0.000302	7.512269	0.558958	-0.8292	0.002882	0.388022	2 to 5 year	8
0.002881	0.000422	5.561703	0.998678	0.051401	0.000468	0.416233	5 to 10 yea	8
0.000858	-0.00035	6.141755	-0.20772	0.978188	0.004976	0.409187	2 to 5 year	2
-0.00011	0.000304	7.088681	-0.39936	0.916795	6.15E-05	0.413402	5 to 10 yea	4
-0.00011	0.000304	7.088681	-0.39936	0.916795	6.15E-05	0.413402	5 to 10 yea	4
-0.00069	-0.00114	8.010088	0.5936	-0.80476	0.003718	0.39511	Over 10 ye	15
-0.00069	-0.00114	8.010088	0.5936	-0.80476	0.003718	0.39511	Over 10 ye	15
-0.00069	-0.00114	8.010088	0.5936	-0.80476	0.003718	0.39511	Over 10 ye	15
-0.00069	-0.00114	8.010088	0.5936	-0.80476	0.003718	0.39511	Over 10 ye	15
-0.00069	-0.00114	8.010088	0.5936	-0.80476	0.003718	0.39511	Over 10 ye	15
-0.00069	-0.00114	8.010088	0.5936	-0.80476	0.003718	0.39511	Over 10 ye	15
-0.00069	-0.00114	8.010088	0.5936	-0.80476	0.003718	0.39511	Over 10 ye	15
-0.00099	-0.00155	15.27797	-0.98557	0.169284	0.000484	0.429518	Over 10 ye	4
-0.00099	-0.00155	15.27797	-0.98557	0.169284	0.000484	0.429518	Over 10 ye	4
6.76E-05	0.000252	8.373786	-0.47368	0.880697	0.067381	0.314345	2 to 5 year	3
-0.00032	-0.00022	7.557192	0.915366	-0.40262	0.00028	0.423083	Over 10 ye	5
-0.00032	-0.00022	7.557192	0.915366	-0.40262	0.00028	0.423083	Over 10 ye	5
-0.00032	-0.00022	7.557192	0.915366	-0.40262	0.00028	0.423083	Over 10 ye	5
-0.00024	-0.00318	12.51608	-0.20785	0.978162	0.000148	0.401935	Over 10 ye	5
-0.00024	-0.00318	12.51608	-0.20785	0.978162	0.000148	0.401935	Over 10 ye	5
9.08E-05	0.00013	7.812431	0.274433	0.961606	0.093409	0.287989	2 to 5 year	3
0.000253	2.23E-05	9.858754	0.137937	-0.99044	0.003718	0.399419	2 to 5 year	16
0.000253	2.23E-05	9.858754	0.137937	-0.99044	0.003718	0.399419	2 to 5 year	16
0.000253	2.23E-05	9.858754	0.137937	-0.99044	0.003718	0.399419	2 to 5 year	16
0.000253	2.23E-05	9.858754	0.137937	-0.99044	0.003718	0.399419	2 to 5 year	16
0.000253	2.23E-05	9.858754	0.137937	-0.99044	0.003718	0.399419	2 to 5 year	16
0.001051	0.00104	6.225508	0.022104	0.999756	0.005495	0.401035	2 to 5 year	2
-0.00124	0.000302	9.16566	-0.97215	-0.23437	0.075947	0.300423	2 to 5 year	7
-0.00091	0.000146	11.52142	0.881388	-0.47239	0.021245	0.375289	5 to 10 yea	12
-0.00091	0.000146	11.52142	0.881388	-0.47239	0.021245	0.375289	5 to 10 yea	12
-0.00133	5.61E-05	8.335838	-0.93209	-0.36222	0.036897	0.350057	5 to 10 yea	12
-0.00133	5.61E-05	8.335838	-0.93209	-0.36222	0.036897	0.350057	5 to 10 yea	12
-0.00133	5.61E-05	8.335838	-0.93209	-0.36222	0.036897	0.350057	5 to 10 yea	12
-0.00133	5.61E-05	8.335838	-0.93209	-0.36222	0.036897	0.350057	5 to 10 yea	12
-0.00133	5.61E-05	8.335838	-0.93209	-0.36222	0.036897	0.350057	5 to 10 yea	12

Table S2 - Anonymized database

-0.0004	0.000957	6.641533	0.442236	0.896899	0.005228	0.388217	2 to 5 year	20
-0.0004	0.000957	6.641533	0.442236	0.896899	0.005228	0.388217	2 to 5 year	20
-0.0004	0.000957	6.641533	0.442236	0.896899	0.005228	0.388217	2 to 5 year	20
-0.0004	0.000957	6.641533	0.442236	0.896899	0.005228	0.388217	2 to 5 year	20
-0.0004	0.000957	6.641533	0.442236	0.896899	0.005228	0.388217	2 to 5 year	20
-0.0004	0.000957	6.641533	0.442236	0.896899	0.005228	0.388217	2 to 5 year	20
-0.0004	0.000957	6.641533	0.442236	0.896899	0.005228	0.388217	2 to 5 year	20
-0.00019	0.000372	7.982374	0.981559	0.191161	0.011815	0.379323	5 to 10 yea	5
-8.50E-05	0.000397	7.945985	0.140679	0.990055	0.033448	0.351633	5 to 10 yea	7
-8.50E-05	0.000397	7.945985	0.140679	0.990055	0.033448	0.351633	5 to 10 yea	7
-8.50E-05	0.000397	7.945985	0.140679	0.990055	0.033448	0.351633	5 to 10 yea	7
0.00045	0.001567	6.19803	0.377834	-0.92587	0.001788	0.397825	Over 10 ye	11
0.00045	0.001567	6.19803	0.377834	-0.92587	0.001788	0.397825	Over 10 ye	11
0.00045	0.001567	6.19803	0.377834	-0.92587	0.001788	0.397825	Over 10 ye	11
0.00045	0.001567	6.19803	0.377834	-0.92587	0.001788	0.397825	Over 10 ye	11
-4.84E-05	0.000152	7.918477	-0.79487	0.606782	0.003819	0.382867	2 to 5 year	3
-4.84E-05	0.000152	7.918477	-0.79487	0.606782	0.003819	0.382867	2 to 5 year	3
0.000122	0.000508	6.87462	-0.92133	0.38879	0.004402	0.396521	2 to 5 year	2
0.000743	-0.00013	7.701545	0.940046	-0.34105	0.091966	0.278508	2 to 5 year	2
0.000743	-0.00013	7.701545	0.940046	-0.34105	0.091966	0.278508	2 to 5 year	2
0.000181	0.001625	6.488424	-0.71545	-0.69866	0.008097	0.387316	1 to 2 year	9
0.000181	0.001625	6.488424	-0.71545	-0.69866	0.008097	0.387316	1 to 2 year	9
0.001139	0.000826	8.643519	0.917286	0.398228	3.06E-05	0.404539	2 to 5 year	10
0.001139	0.000826	8.643519	0.917286	0.398228	3.06E-05	0.404539	2 to 5 year	10
0.001139	0.000826	8.643519	0.917286	0.398228	3.06E-05	0.404539	2 to 5 year	10
0.001139	0.000826	8.643519	0.917286	0.398228	3.06E-05	0.404539	2 to 5 year	10
0.000352	0.000302	6.929667	-0.10627	-0.99434	0.003855	0.402067	2 to 5 year	1
-0.0002	0.001013	6.953256	0.983781	0.179375	0.002878	0.393284	5 to 10 yea	15
-0.0002	0.001013	6.953256	0.983781	0.179375	0.002878	0.393284	5 to 10 yea	15
-0.0002	0.001013	6.953256	0.983781	0.179375	0.002878	0.393284	5 to 10 yea	15
-0.0002	0.001013	6.953256	0.983781	0.179375	0.002878	0.393284	5 to 10 yea	15
-0.0002	0.001013	6.953256	0.983781	0.179375	0.002878	0.393284	5 to 10 yea	15
-0.0002	0.001013	6.953256	0.983781	0.179375	0.002878	0.393284	5 to 10 yea	15
-0.0002	0.001013	6.953256	0.983781	0.179375	0.002878	0.393284	5 to 10 yea	15
-0.0002	0.001013	6.953256	0.983781	0.179375	0.002878	0.393284	5 to 10 yea	15
-0.00041	-0.00111	11.81479	-0.34196	-0.93972	0.02326	0.372225	5 to 10 yea	8
-0.00041	-0.00111	11.81479	-0.34196	-0.93972	0.02326	0.372225	5 to 10 yea	8
5.87E-05	-0.00011	9.120533	-0.22296	0.974829	0.037514	0.339528	2 to 5 year	3
5.87E-05	-0.00011	9.120533	-0.22296	0.974829	0.037514	0.339528	2 to 5 year	3
-3.60E-05	-8.79E-05	8.242848	-0.22782	0.973704	0.0006	0.432857	2 to 5 year	5
-0.0004	0.000957	6.641533	0.442236	0.896899	0.005228	0.388217	5 to 10 yea	26
-0.0004	0.000957	6.641533	0.442236	0.896899	0.005228	0.388217	5 to 10 yea	26
-0.0004	0.000957	6.641533	0.442236	0.896899	0.005228	0.388217	5 to 10 yea	26
-0.0004	0.000957	6.641533	0.442236	0.896899	0.005228	0.388217	5 to 10 yea	26
-0.0004	0.000957	6.641533	0.442236	0.896899	0.005228	0.388217	5 to 10 yea	26
-0.0004	0.000957	6.641533	0.442236	0.896899	0.005228	0.388217	5 to 10 yea	26
0.000452	-0.00043	8.325767	-0.38681	-0.92216	0.000667	0.38089	Less than 1	2
0.002697	0.002325	5.800778	0.905334	-0.4247	0.001936	0.399948	Over 10 ye	17
0.002697	0.002325	5.800778	0.905334	-0.4247	0.001936	0.399948	Over 10 ye	17
0.002697	0.002325	5.800778	0.905334	-0.4247	0.001936	0.399948	Over 10 ye	17

Table S2 - Anonymized database

0.002697	0.002325	5.800778	0.905334	-0.4247	0.001936	0.399948	Over 10 ye	17
0.002697	0.002325	5.800778	0.905334	-0.4247	0.001936	0.399948	Over 10 ye	17
0.002697	0.002325	5.800778	0.905334	-0.4247	0.001936	0.399948	Over 10 ye	17
0.002697	0.002325	5.800778	0.905334	-0.4247	0.001936	0.399948	Over 10 ye	17
0.000891	0.00392	5.304569	0.842642	0.538474	4.06E-05	0.399017	2 to 5 year	9
0.000891	0.00392	5.304569	0.842642	0.538474	4.06E-05	0.399017	2 to 5 year	9
0.000891	0.00392	5.304569	0.842642	0.538474	4.06E-05	0.399017	2 to 5 year	9
-0.00031	0.000173	8.677309	0.540681	-0.84123	0.024952	0.366882	1 to 2 year	2
-0.00031	0.000173	8.677309	0.540681	-0.84123	0.024952	0.366882	1 to 2 year	2
-0.00068	2.72E-05	8.409824	0.47124	0.882005	0.039537	0.326279	2 to 5 year	14
-0.00068	2.72E-05	8.409824	0.47124	0.882005	0.039537	0.326279	2 to 5 year	14
-0.00068	2.72E-05	8.409824	0.47124	0.882005	0.039537	0.326279	2 to 5 year	14
-0.0004	0.000669	7.70624	-0.99708	0.076361	0.019945	0.383244	2 to 5 year	6
-0.0004	0.000669	7.70624	-0.99708	0.076361	0.019945	0.383244	2 to 5 year	6
-7.18E-05	-1.56E-05	7.911486	-0.3367	-0.94161	0.000646	0.422779	5 to 10 yea	9
-7.18E-05	-1.56E-05	7.911486	-0.3367	-0.94161	0.000646	0.422779	5 to 10 yea	9
-7.18E-05	-1.56E-05	7.911486	-0.3367	-0.94161	0.000646	0.422779	5 to 10 yea	9
-0.00064	-0.00037	8.810884	0.159075	-0.98727	0.017465	0.406714	5 to 10 yea	8
-0.00064	-0.00037	8.810884	0.159075	-0.98727	0.017465	0.406714	5 to 10 yea	8
0.000443	0.00158	6.237093	-0.99049	0.137557	0.001588	0.360391	Over 10 ye	31
0.000443	0.00158	6.237093	-0.99049	0.137557	0.001588	0.360391	Over 10 ye	31
0.000443	0.00158	6.237093	-0.99049	0.137557	0.001588	0.360391	Over 10 ye	31
0.000443	0.00158	6.237093	-0.99049	0.137557	0.001588	0.360391	Over 10 ye	31
0.000443	0.00158	6.237093	-0.99049	0.137557	0.001588	0.360391	Over 10 ye	31
0.000443	0.00158	6.237093	-0.99049	0.137557	0.001588	0.360391	Over 10 ye	31
0.000443	0.00158	6.237093	-0.99049	0.137557	0.001588	0.360391	Over 10 ye	31
0.000722	-0.00228	10.3242	-0.90898	-0.41683	0.002737	0.389425	Less than 1	3
0.001296	-0.00068	8.44785	0.663142	0.748494	0.009909	0.387572	2 to 5 year	8
-0.00052	-0.00071	8.980148	0.447706	0.894181	0.08958	0.278293	Over 10 ye	28
-0.00052	-0.00071	8.980148	0.447706	0.894181	0.08958	0.278293	Over 10 ye	28
-0.00052	-0.00071	8.980148	0.447706	0.894181	0.08958	0.278293	Over 10 ye	28
-0.00052	-0.00071	8.980148	0.447706	0.894181	0.08958	0.278293	Over 10 ye	28
-0.00052	-0.00071	8.980148	0.447706	0.894181	0.08958	0.278293	Over 10 ye	28
0.000333	0.000143	7.315743	0.993855	-0.11069	0.055491	0.279209	2 to 5 year	3
0.000333	0.000143	7.315743	0.993855	-0.11069	0.055491	0.279209	2 to 5 year	3
-0.00547	-0.00471	9.736187	-0.29985	-0.95399	0.005529	0.407736	5 to 10 yea	5
-0.00547	-0.00471	9.736187	-0.29985	-0.95399	0.005529	0.407736	5 to 10 yea	5
-0.00547	-0.00471	9.736187	-0.29985	-0.95399	0.005529	0.407736	5 to 10 yea	5
0.000556	0.000357	6.866586	-0.81302	0.582238	0.022762	0.38422	2 to 5 year	1
-0.00158	-0.00072	6.94064	-0.32886	0.94438	0.024372	0.374732	Less than 1	2
-0.00089	-0.00035	7.0788	-0.71109	-0.7031	0.003488	0.403009	2 to 5 year	5
-0.00032	0.00039	7.819598	-0.16045	0.987043	0.013231	0.403303	2 to 5 year	2
-0.00111	-0.00027	10.13836	0.715309	-0.69881	0.036897	0.350057	Over 10 ye	10
-0.00111	-0.00027	10.13836	0.715309	-0.69881	0.036897	0.350057	Over 10 ye	10
-0.00111	-0.00027	10.13836	0.715309	-0.69881	0.036897	0.350057	Over 10 ye	10
-0.00111	-0.00027	10.13836	0.715309	-0.69881	0.036897	0.350057	Over 10 ye	10
-0.00111	-0.00027	10.13836	0.715309	-0.69881	0.036897	0.350057	Over 10 ye	10
0.001105	0.000415	5.860742	-0.63306	-0.7741	0.000107	0.404435	5 to 10 yea	5
-0.00093	0.000257	8.140813	0.99914	-0.04147	0.048512	0.34648	2 to 5 year	3
-0.00093	0.000257	8.140813	0.99914	-0.04147	0.048512	0.34648	2 to 5 year	3

Table S2 - Anonymized database

-0.00154	-0.00157	9.944622	-0.94558	-0.32538	0.008966	0.390204	1 to 2 year	2
0.000452	0.000842	6.690324	-0.85015	-0.52654	0.032563	0.348585	1 to 2 year	3
0.001133	0.000899	6.576385	0.988113	0.153732	0.002441	0.427285	1 to 2 year	2
0.001133	0.000899	6.576385	0.988113	0.153732	0.002441	0.427285	1 to 2 year	2
0.001576	0.000457	6.224496	0.630085	0.776526	0.044078	0.371762	1 to 2 year	2
0.001339	-0.00023	7.543291	0.125484	0.992096	0.005514	0.398555	5 to 10 yea	15
0.001339	-0.00023	7.543291	0.125484	0.992096	0.005514	0.398555	5 to 10 yea	15
0.001339	-0.00023	7.543291	0.125484	0.992096	0.005514	0.398555	5 to 10 yea	15
0.00033	0.000413	7.68102	0.622847	0.782343	0.01425	0.411578	Over 10 ye	8
0.00033	0.000413	7.68102	0.622847	0.782343	0.01425	0.411578	Over 10 ye	8
0.000131	-0.00029	9.292435	-0.10971	0.993964	0.037466	0.339227	Over 10 ye	3
0.000131	-0.00029	9.292435	-0.10971	0.993964	0.037466	0.339227	Over 10 ye	3
0.000413	-0.00087	6.962271	0.945246	0.326358	0.001523	0.379229	1 to 2 year	1
-0.00026	0.000462	8.163028	0.284725	-0.95861	0.024897	0.373615	1 to 2 year	2
0.000135	-0.00132	8.828544	0.684457	-0.72905	0.043763	0.373741	2 to 5 year	19
0.000135	-0.00132	8.828544	0.684457	-0.72905	0.043763	0.373741	2 to 5 year	19
0.000135	-0.00132	8.828544	0.684457	-0.72905	0.043763	0.373741	2 to 5 year	19
0.000135	-0.00132	8.828544	0.684457	-0.72905	0.043763	0.373741	2 to 5 year	19
0.000135	-0.00132	8.828544	0.684457	-0.72905	0.043763	0.373741	2 to 5 year	19
0.000135	-0.00132	8.828544	0.684457	-0.72905	0.043763	0.373741	2 to 5 year	19
0.000135	-0.00132	8.828544	0.684457	-0.72905	0.043763	0.373741	2 to 5 year	19
0.000135	-0.00132	8.828544	0.684457	-0.72905	0.043763	0.373741	2 to 5 year	19
-0.00027	0.00029	7.187155	0.29133	-0.95662	0.027795	0.37041	Less than 1	2
-0.00013	2.74E-05	7.615221	-0.22678	0.973947	0.015185	0.373612	5 to 10 yea	7
-0.00013	2.74E-05	7.615221	-0.22678	0.973947	0.015185	0.373612	5 to 10 yea	7
-0.00013	2.74E-05	7.615221	-0.22678	0.973947	0.015185	0.373612	5 to 10 yea	7
-0.00013	2.74E-05	7.615221	-0.22678	0.973947	0.015185	0.373612	5 to 10 yea	7
-0.0017	-0.00024	7.246581	-0.94994	-0.31242	0.001859	0.392238	Over 10 ye	11
-0.0017	-0.00024	7.246581	-0.94994	-0.31242	0.001859	0.392238	Over 10 ye	11
-0.0017	-0.00024	7.246581	-0.94994	-0.31242	0.001859	0.392238	Over 10 ye	11
-0.0017	-0.00024	7.246581	-0.94994	-0.31242	0.001859	0.392238	Over 10 ye	11
0.000406	0.00014	7.523566	0.726085	-0.6876	0.034812	0.339728	2 to 5 year	6
0.000406	0.00014	7.523566	0.726085	-0.6876	0.034812	0.339728	2 to 5 year	6
-0.00023	0.000335	8.117977	-0.3198	0.947486	0.002843	0.430213	2 to 5 year	2
-0.00023	0.000335	8.117977	-0.3198	0.947486	0.002843	0.430213	2 to 5 year	2
-0.00163	0.000282	6.320958	-0.53334	0.845902	0.065325	0.32561	2 to 5 year	15
-0.00163	0.000282	6.320958	-0.53334	0.845902	0.065325	0.32561	2 to 5 year	15
-0.00163	0.000282	6.320958	-0.53334	0.845902	0.065325	0.32561	2 to 5 year	15
-0.00163	0.000282	6.320958	-0.53334	0.845902	0.065325	0.32561	2 to 5 year	15
-0.00066	0.000356	7.34828	0.48702	-0.87339	0.004815	0.391996	2 to 5 year	5
-0.00066	0.000356	7.34828	0.48702	-0.87339	0.004815	0.391996	2 to 5 year	5
-0.00066	0.000356	7.34828	0.48702	-0.87339	0.004815	0.391996	2 to 5 year	5
-0.00037	8.18E-05	9.069148	-0.38439	-0.92317	0.000216	0.430965	1 to 2 year	2
-8.74E-05	0.000909	7.239906	0.181161	-0.98345	0.033777	0.366614	2 to 5 year	11
-8.74E-05	0.000909	7.239906	0.181161	-0.98345	0.033777	0.366614	2 to 5 year	11
-8.74E-05	0.000909	7.239906	0.181161	-0.98345	0.033777	0.366614	2 to 5 year	11
-8.74E-05	0.000909	7.239906	0.181161	-0.98345	0.033777	0.366614	2 to 5 year	11
0.001516	0.001423	5.770835	-0.80301	-0.59597	8.47E-05	0.403413	1 to 2 year	1
2.60E-05	0.000763	5.972576	0.988796	0.149274	0.012535	0.405665	2 to 5 year	1
-0.00035	0.000564	6.56746	-0.9893	0.145864	0.008895	0.411329	Less than 1	1

Table S2 - Anonymized database

-0.00087	-0.00074	8.42246	0.919444	-0.39322	0.000542	0.421315	2 to 5 year	1
0.000277	-0.00146	6.302177	-0.33404	-0.94256	0.024641	0.373095	2 to 5 year	1
0.001349	0.000827	6.187137	0.943099	-0.33251	0.021619	0.385329	1 to 2 year	1
-5.00E-06	0.000193	8.795108	-0.62124	-0.78362	0.004689	0.411139	Less than 1	1
0.000228	-0.0001	8.286763	-0.53674	-0.84375	7.60E-06	0.371811	1 to 2 year	1
0.002791	-0.00029	5.59797	0.994875	-0.10112	0.000414	0.426809	2 to 5 year	2
-0.00597	-0.00256	9.492289	0.701878	-0.7123	0.000216	0.430965	2 to 5 year	2
-0.00081	-0.00139	7.623704	-0.89808	0.439828	0.026385	0.372594	1 to 2 year	2
-0.00081	-0.00139	7.623704	-0.89808	0.439828	0.026385	0.372594	1 to 2 year	2
0.000526	-0.00148	5.582147	0.572154	-0.82015	0.025125	0.351967	1 to 2 year	2
0.000526	-0.00148	5.582147	0.572154	-0.82015	0.025125	0.351967	1 to 2 year	2
-0.00243	-0.00465	7.259941	0.896445	0.443156	6.97E-05	0.422515	2 to 5 year	2
-0.00108	0.000143	9.864983	-0.52344	0.852061	0.018665	0.380921	2 to 5 year	2
0.000249	0.000448	6.982077	0.999605	0.028089	0.010763	0.38082	1 to 2 year	2
0.000249	0.000448	6.982077	0.999605	0.028089	0.010763	0.38082	1 to 2 year	2
0.000918	0.000957	7.354772	0.678338	0.73475	0.014464	0.321976	Less than 1	2
-0.00103	-0.0005	9.234109	-0.53885	0.842403	0.077446	0.322683	2 to 5 year	2
-0.00017	-8.30E-05	10.68071	-0.56871	-0.82254	0.00063	0.42541	5 to 10 yea	2
-0.0005	0.00166	6.079113	-0.63624	-0.77149	0.009163	0.405865	1 to 2 year	2
0.000199	-0.00019	8.966571	-0.91646	-0.40012	0.004935	0.377711	5 to 10 yea	2
-0.00237	0.000735	6.755063	0.091776	-0.99578	0.017132	0.354799	2 to 5 year	2
0.00018	-0.00015	9.943292	0.995409	-0.09571	0.010153	0.375219	2 to 5 year	2
0.001771	0.001576	6.105651	-0.99955	0.029905	0.000843	0.430229	Over 10 ye	2
0.000971	0.00096	6.294988	0.703294	-0.7109	0.039269	0.364845	5 to 10 yea	2
0.000425	0.000244	7.423181	-0.58327	0.81228	0.006118	0.419368	1 to 2 year	2
0.000162	0.001492	7.414687	0.046305	0.998927	0.002787	0.412379	2 to 5 year	2
0.000162	0.001492	7.414687	0.046305	0.998927	0.002787	0.412379	2 to 5 year	2
-0.00048	-0.00042	10.2381	-0.54978	0.835311	0.021104	0.374728	2 to 5 year	3
-0.00102	-0.00061	11.12156	0.96399	0.265938	0.050741	0.34748	1 to 2 year	3
-0.00102	-0.00061	11.12156	0.96399	0.265938	0.050741	0.34748	1 to 2 year	3
0.000476	-0.0004	9.722341	0.966119	0.258098	0.036207	0.34751	5 to 10 yea	3
0.000476	-0.0004	9.722341	0.966119	0.258098	0.036207	0.34751	5 to 10 yea	3
0.001039	0	7.701547	0.757136	-0.65326	0.030647	0.366727	5 to 10 yea	3
3.00E-06	-0.00061	12.34398	0.695892	0.718146	3.18E-05	0.406127	2 to 5 year	3
-0.00061	-0.00019	8.427426	-0.53394	0.845524	0.000184	0.415632	2 to 5 year	3
-0.00324	-9.00E-04	12.5425	-0.65231	0.757956	0.052029	0.311606	2 to 5 year	3
0.001345	0.002955	5.291983	-0.99946	-0.03292	0.001659	0.403552	2 to 5 year	4
0.001345	0.002955	5.291983	-0.99946	-0.03292	0.001659	0.403552	2 to 5 year	4
0.001345	0.002955	5.291983	-0.99946	-0.03292	0.001659	0.403552	2 to 5 year	4
0.001345	0.002955	5.291983	-0.99946	-0.03292	0.001659	0.403552	2 to 5 year	4
-0.00043	0.000222	6.91947	-0.59886	-0.80085	0.003814	0.39002	2 to 5 year	4
-0.00043	0.000222	6.91947	-0.59886	-0.80085	0.003814	0.39002	2 to 5 year	4
0.000486	0.000696	7.21388	-0.99999	0.004943	0.003964	0.413122	5 to 10 yea	4
0.001133	0.000546	6.975701	-0.65862	0.752475	0.004293	0.399878	5 to 10 yea	4
0.002444	-0.00025	6.068515	-0.99992	-0.01246	0.00288	0.395734	2 to 5 year	4
-0.00031	0.000414	6.752109	-0.48257	-0.87585	0.000372	0.396149	2 to 5 year	4
-0.00031	0.000414	6.752109	-0.48257	-0.87585	0.000372	0.396149	2 to 5 year	4
0.000106	0.000435	10.30726	-0.07783	0.996966	0.000332	0.395629	5 to 10 yea	4
-0.00087	-0.00049	8.240852	0.281379	-0.9596	0.004232	0.388678	1 to 2 year	4
-0.00087	-0.00049	8.240852	0.281379	-0.9596	0.004232	0.388678	1 to 2 year	4

Table S2 - Anonymized database

-0.00087	-0.00049	8.240852	0.281379	-0.9596	0.004232	0.388678	1 to 2 year	4
0.000332	0.001614	5.844646	0.999888	0.014955	0.022942	0.378116	Over 10 ye	4
-0.00018	0.000166	9.809126	-0.98811	-0.15374	0.017034	0.383016	2 to 5 year	4
-0.00091	-0.00011	7.181785	0.96959	0.244737	0.003353	0.400542	2 to 5 year	5
-0.00091	-0.00011	7.181785	0.96959	0.244737	0.003353	0.400542	2 to 5 year	5
-0.00091	-0.00011	7.181785	0.96959	0.244737	0.003353	0.400542	2 to 5 year	5
-0.00013	0.000117	9.363936	-0.85537	-0.51802	0.068087	0.336834	2 to 5 year	5
0.000384	0.000594	7.174697	-0.35256	0.935788	0.042672	0.359447	2 to 5 year	5
0.000384	0.000594	7.174697	-0.35256	0.935788	0.042672	0.359447	2 to 5 year	5
0.000384	0.000594	7.174697	-0.35256	0.935788	0.042672	0.359447	2 to 5 year	5
-4.00E-05	0.000166	6.602221	-0.24178	0.97033	0.042414	0.328016	2 to 5 year	5
-4.00E-05	0.000166	6.602221	-0.24178	0.97033	0.042414	0.328016	2 to 5 year	5
-0.00181	-3.80E-05	11.36418	-0.63714	0.770751	0.001584	0.411519	5 to 10 yea	5
-0.00028	-0.00062	10.31837	-0.92986	-0.3679	0.062812	0.32094	2 to 5 year	5
-0.00028	-0.00062	10.31837	-0.92986	-0.3679	0.062812	0.32094	2 to 5 year	5
0.002301	0.003581	5.215646	0.603308	0.797508	0.01096	0.389974	1 to 2 year	5
0.002301	0.003581	5.215646	0.603308	0.797508	0.01096	0.389974	1 to 2 year	5
0.00364	0.001644	5.660378	-0.83004	-0.55771	0.062955	0.326097	1 to 2 year	5
0.00364	0.001644	5.660378	-0.83004	-0.55771	0.062955	0.326097	1 to 2 year	5
8.50E-05	-0.00011	8.182613	0.65839	-0.75268	0.00036	0.432406	2 to 5 year	5
8.50E-05	-0.00011	8.182613	0.65839	-0.75268	0.00036	0.432406	2 to 5 year	5
8.50E-05	-0.00011	8.182613	0.65839	-0.75268	0.00036	0.432406	2 to 5 year	5
-0.00014	0.000165	10.64129	0.875599	-0.48304	0.003819	0.382867	2 to 5 year	5
-0.00014	0.000165	10.64129	0.875599	-0.48304	0.003819	0.382867	2 to 5 year	5
0.00096	9.00E-05	6.846452	0.995135	-0.09852	0.035441	0.339374	2 to 5 year	5
0.00096	9.00E-05	6.846452	0.995135	-0.09852	0.035441	0.339374	2 to 5 year	5
0.001131	0.00063	7.54068	0.908182	-0.41858	0.339062	0.343287	5 to 10 yea	5
0.001131	0.00063	7.54068	0.908182	-0.41858	0.339062	0.343287	5 to 10 yea	5
0.001447	0.000749	5.42723	0.013815	0.999905	0.000165	0.401891	Over 10 ye	5
-0.00011	-0.00023	10.92632	0.31408	-0.9494	0.064053	0.313069	Over 10 ye	5
-0.00048	-0.00042	8.983812	-0.95159	0.307365	0.001385	0.40293	2 to 5 year	5
-0.00048	-0.00042	8.983812	-0.95159	0.307365	0.001385	0.40293	2 to 5 year	5
0.000585	-9.20E-05	7.951361	0.67058	0.741837	0.039557	0.340828	2 to 5 year	5
0.000585	-9.20E-05	7.951361	0.67058	0.741837	0.039557	0.340828	2 to 5 year	5
-0.002	-0.0005	7.043015	-0.99784	-0.06576	0.002133	0.399649	2 to 5 year	6
-0.002	-0.0005	7.043015	-0.99784	-0.06576	0.002133	0.399649	2 to 5 year	6
-0.002	-0.0005	7.043015	-0.99784	-0.06576	0.002133	0.399649	2 to 5 year	6
-0.002	-0.0005	7.043015	-0.99784	-0.06576	0.002133	0.399649	2 to 5 year	6
2.80E-05	0.000158	7.935713	0.986909	-0.16128	0.01295	0.403363	5 to 10 yea	6
-3.40E-05	-0.00087	9.206155	-0.75518	0.65552	0.021835	0.387149	5 to 10 yea	6
-0.00126	0.000191	7.233391	0.378898	0.925438	0.008101	0.393838	1 to 2 year	6
-0.00249	-0.00183	7.791164	0.833364	0.552724	0.000654	0.433276	5 to 10 yea	6
-0.00249	-0.00183	7.791164	0.833364	0.552724	0.000654	0.433276	5 to 10 yea	6
-0.00037	0.000395	7.243243	-0.01524	0.999884	0.037988	0.325697	2 to 5 year	6
-0.00037	0.000395	7.243243	-0.01524	0.999884	0.037988	0.325697	2 to 5 year	6
0.002139	-0.00178	6.95804	-0.13969	0.990196	0.009714	0.385306	1 to 2 year	7
0.002139	-0.00178	6.95804	-0.13969	0.990196	0.009714	0.385306	1 to 2 year	7
0.002139	-0.00178	6.95804	-0.13969	0.990196	0.009714	0.385306	1 to 2 year	7
0.000545	-0.00015	7.788049	0.711975	-0.70221	0.079883	0.293708	2 to 5 year	7
0.000545	-0.00015	7.788049	0.711975	-0.70221	0.079883	0.293708	2 to 5 year	7

Table S2 - Anonymized database

0.004227	0.002337	5.757184	-0.80577	-0.59222	0.000278	0.402242	Over 10 ye	7
0.004227	0.002337	5.757184	-0.80577	-0.59222	0.000278	0.402242	Over 10 ye	7
0.000112	0.000241	7.972994	0.449417	0.893322	0.002402	0.419275	2 to 5 year	7
0.000112	0.000241	7.972994	0.449417	0.893322	0.002402	0.419275	2 to 5 year	7
0.000112	0.000241	7.972994	0.449417	0.893322	0.002402	0.419275	2 to 5 year	7
0.000112	0.000241	7.972994	0.449417	0.893322	0.002402	0.419275	2 to 5 year	7
0.001105	0.000415	5.860742	-0.63306	-0.7741	0.000107	0.404435	5 to 10 yea	7
-0.00063	-6.20E-05	8.821591	-0.96326	-0.26858	0.050648	0.295265	2 to 5 year	7
-0.00063	-6.20E-05	8.821591	-0.96326	-0.26858	0.050648	0.295265	2 to 5 year	7
0.000179	-0.00019	9.676757	0.818019	0.575191	0.014334	0.395231	Over 10 ye	7
0.000179	-0.00019	9.676757	0.818019	0.575191	0.014334	0.395231	Over 10 ye	7
0.000179	-0.00019	9.676757	0.818019	0.575191	0.014334	0.395231	Over 10 ye	7
0.000179	-0.00019	9.676757	0.818019	0.575191	0.014334	0.395231	Over 10 ye	7
-0.0006	0.001251	7.111355	-0.90249	0.430702	0.002676	0.421914	5 to 10 yea	7
0.000708	0.000582	7.037695	0.990504	0.137486	0.014558	0.400028	Over 10 ye	7
0.000708	0.000582	7.037695	0.990504	0.137486	0.014558	0.400028	Over 10 ye	7
-0.00029	-6.10E-05	10.92574	0.009512	-0.99995	0.040339	0.342214	2 to 5 year	8
-0.00029	-6.10E-05	10.92574	0.009512	-0.99995	0.040339	0.342214	2 to 5 year	8
-0.00149	-0.00148	8.976081	-0.75992	0.650019	0.009797	0.373252	2 to 5 year	8
-0.00149	-0.00148	8.976081	-0.75992	0.650019	0.009797	0.373252	2 to 5 year	8
-0.00149	-0.00148	8.976081	-0.75992	0.650019	0.009797	0.373252	2 to 5 year	8
0.003221	0.001054	5.788663	0.94556	0.325447	0.005833	0.395439	2 to 5 year	8
0.002565	0.000252	7.01588	-0.81028	-0.58604	0.008547	0.386603	5 to 10 yea	8
-0.00125	-0.00089	6.507705	0.751696	-0.65951	0.059097	0.317329	2 to 5 year	8
-0.00125	-0.00089	6.507705	0.751696	-0.65951	0.059097	0.317329	2 to 5 year	8
0.000404	-0.00101	6.550444	-0.98501	0.172514	0.000392	0.415925	5 to 10 yea	9
0.000404	-0.00101	6.550444	-0.98501	0.172514	0.000392	0.415925	5 to 10 yea	9
0.000404	-0.00101	6.550444	-0.98501	0.172514	0.000392	0.415925	5 to 10 yea	9
0.000265	-0.00173	8.467335	0.624563	-0.78097	0.032531	0.341907	2 to 5 year	9
0.000265	-0.00173	8.467335	0.624563	-0.78097	0.032531	0.341907	2 to 5 year	9
0.000265	-0.00173	8.467335	0.624563	-0.78097	0.032531	0.341907	2 to 5 year	9
0.000265	-0.00173	8.467335	0.624563	-0.78097	0.032531	0.341907	2 to 5 year	9
2.20E-05	-0.00026	8.60884	0.084634	-0.99641	1.55E-05	0.411413	2 to 5 year	9
2.20E-05	-0.00026	8.60884	0.084634	-0.99641	1.55E-05	0.411413	2 to 5 year	9
2.20E-05	-0.00026	8.60884	0.084634	-0.99641	1.55E-05	0.411413	2 to 5 year	9
0.000705	0.000384	6.483116	-0.75141	0.659836	0.016717	0.333957	Over 10 ye	9
0.000705	0.000384	6.483116	-0.75141	0.659836	0.016717	0.333957	Over 10 ye	9
0.000705	0.000384	6.483116	-0.75141	0.659836	0.016717	0.333957	Over 10 ye	9
0.002295	0.00551	4.841191	-0.19558	0.980687	0.006205	0.387621	5 to 10 yea	10
0.002295	0.00551	4.841191	-0.19558	0.980687	0.006205	0.387621	5 to 10 yea	10
0.002295	0.00551	4.841191	-0.19558	0.980687	0.006205	0.387621	5 to 10 yea	10
-0.00351	-0.00493	9.698227	0.9886	-0.15057	0.05117	0.320754	5 to 10 yea	10
-0.00351	-0.00493	9.698227	0.9886	-0.15057	0.05117	0.320754	5 to 10 yea	10
0.00096	0.005301	5.114022	0.944001	-0.32994	4.06E-05	0.399017	5 to 10 yea	10
0.00096	0.005301	5.114022	0.944001	-0.32994	4.06E-05	0.399017	5 to 10 yea	10
0.00096	0.005301	5.114022	0.944001	-0.32994	4.06E-05	0.399017	5 to 10 yea	10
0.00096	0.005301	5.114022	0.944001	-0.32994	4.06E-05	0.399017	5 to 10 yea	10
0.00096	0.005301	5.114022	0.944001	-0.32994	4.06E-05	0.399017	5 to 10 yea	10
-0.00019	0.000252	9.080857	-0.15098	0.988537	0.023393	0.371974	Over 10 ye	10
-0.00019	0.000252	9.080857	-0.15098	0.988537	0.023393	0.371974	Over 10 ye	10

Table S2 - Anonymized database

3.30E-05	0.000103	7.984262	0.497598	-0.86741	0.004843	0.391127	5 to 10 yea	11
3.30E-05	0.000103	7.984262	0.497598	-0.86741	0.004843	0.391127	5 to 10 yea	11
3.30E-05	0.000103	7.984262	0.497598	-0.86741	0.004843	0.391127	5 to 10 yea	11
-0.00037	0.000636	6.427949	-0.7724	-0.63513	0.003948	0.402743	Over 10 ye	11
-0.00037	0.000636	6.427949	-0.7724	-0.63513	0.003948	0.402743	Over 10 ye	11
-0.00037	0.000636	6.427949	-0.7724	-0.63513	0.003948	0.402743	Over 10 ye	11
0.000382	-0.00059	12.14705	0.290701	0.956814	0.001242	0.395773	1 to 2 year	11
0.000382	-0.00059	12.14705	0.290701	0.956814	0.001242	0.395773	1 to 2 year	11
0.000373	0.000481	7.152483	-0.42747	-0.90403	0.016893	0.387055	2 to 5 year	11
0.000373	0.000481	7.152483	-0.42747	-0.90403	0.016893	0.387055	2 to 5 year	11
0.000373	0.000481	7.152483	-0.42747	-0.90403	0.016893	0.387055	2 to 5 year	11
0.000373	0.000481	7.152483	-0.42747	-0.90403	0.016893	0.387055	2 to 5 year	11
0.000373	0.000481	7.152483	-0.42747	-0.90403	0.016893	0.387055	2 to 5 year	11
0.000373	0.000481	7.152483	-0.42747	-0.90403	0.016893	0.387055	2 to 5 year	11
3.70E-05	0.000344	6.792089	-0.99023	0.139451	0.012965	0.387951	2 to 5 year	12
3.70E-05	0.000344	6.792089	-0.99023	0.139451	0.012965	0.387951	2 to 5 year	12
3.70E-05	0.000344	6.792089	-0.99023	0.139451	0.012965	0.387951	2 to 5 year	12
3.70E-05	0.000344	6.792089	-0.99023	0.139451	0.012965	0.387951	2 to 5 year	12
-7.20E-05	-4.70E-05	11.12294	0.04965	0.998767	0.103332	0.286539	Over 10 ye	12
-7.20E-05	-4.70E-05	11.12294	0.04965	0.998767	0.103332	0.286539	Over 10 ye	12
-7.20E-05	-4.70E-05	11.12294	0.04965	0.998767	0.103332	0.286539	Over 10 ye	12
-7.20E-05	-4.70E-05	11.12294	0.04965	0.998767	0.103332	0.286539	Over 10 ye	12
0.000125	0.000106	8.09158	-0.47147	-0.88188	0.001293	0.395439	5 to 10 yea	12
0.000125	0.000106	8.09158	-0.47147	-0.88188	0.001293	0.395439	5 to 10 yea	12
0.000125	0.000106	8.09158	-0.47147	-0.88188	0.001293	0.395439	5 to 10 yea	12
-3.90E-05	-0.00066	10.38832	-0.13929	-0.99025	0.046155	0.323061	Over 10 ye	15
-3.90E-05	-0.00066	10.38832	-0.13929	-0.99025	0.046155	0.323061	Over 10 ye	15
-3.90E-05	-0.00066	10.38832	-0.13929	-0.99025	0.046155	0.323061	Over 10 ye	15
-3.90E-05	-0.00066	10.38832	-0.13929	-0.99025	0.046155	0.323061	Over 10 ye	15
-3.90E-05	-0.00066	10.38832	-0.13929	-0.99025	0.046155	0.323061	Over 10 ye	15
-0.00045	-0.0004	9.139278	-0.97586	-0.21837	0.009431	0.390315	2 to 5 year	20
-0.00045	-0.0004	9.139278	-0.97586	-0.21837	0.009431	0.390315	2 to 5 year	20
-0.00045	-0.0004	9.139278	-0.97586	-0.21837	0.009431	0.390315	2 to 5 year	20
-0.00045	-0.0004	9.139278	-0.97586	-0.21837	0.009431	0.390315	2 to 5 year	20
-0.00018	0.000577	7.314416	0.452999	0.891511	0.078283	0.307638	5 to 10 yea	11
-0.00018	0.000577	7.314416	0.452999	0.891511	0.078283	0.307638	5 to 10 yea	11
-0.00018	0.000577	7.314416	0.452999	0.891511	0.078283	0.307638	5 to 10 yea	11
-0.00018	0.000577	7.314416	0.452999	0.891511	0.078283	0.307638	5 to 10 yea	11
-0.00354	-0.0001	7.882977	0.95887	-0.28384	0.000103	0.423653	1 to 2 year	12
-0.00354	-0.0001	7.882977	0.95887	-0.28384	0.000103	0.423653	1 to 2 year	12
-0.00354	-0.0001	7.882977	0.95887	-0.28384	0.000103	0.423653	1 to 2 year	12
-0.00023	0.000294	7.396209	-0.85928	0.511503	0.102343	0.287067	5 to 10 yea	13
-0.00023	0.000294	7.396209	-0.85928	0.511503	0.102343	0.287067	5 to 10 yea	13
-0.00023	0.000294	7.396209	-0.85928	0.511503	0.102343	0.287067	5 to 10 yea	13
0.00033	0.000413	7.68102	0.622847	0.782343	0.01425	0.411578	Over 10 ye	14
0.00033	0.000413	7.68102	0.622847	0.782343	0.01425	0.411578	Over 10 ye	14
0.00033	0.000413	7.68102	0.622847	0.782343	0.01425	0.411578	Over 10 ye	14
-0.00028	0.000453	6.854851	0.797983	0.60268	0.009312	0.395292	2 to 5 year	16
-0.00028	0.000453	6.854851	0.797983	0.60268	0.009312	0.395292	2 to 5 year	16
-0.00028	0.000453	6.854851	0.797983	0.60268	0.009312	0.395292	2 to 5 year	16

Table S2 - Anonymized database

-0.00028	0.000453	6.854851	0.797983	0.60268	0.009312	0.395292	2 to 5 year	16
-0.00054	-0.00053	7.974645	0.37949	-0.9252	0.003664	0.395188	Over 10 ye	16
-0.00054	-0.00053	7.974645	0.37949	-0.9252	0.003664	0.395188	Over 10 ye	16
-0.00054	-0.00053	7.974645	0.37949	-0.9252	0.003664	0.395188	Over 10 ye	16
-0.00054	-0.00053	7.974645	0.37949	-0.9252	0.003664	0.395188	Over 10 ye	16
-0.00054	-0.00053	7.974645	0.37949	-0.9252	0.003664	0.395188	Over 10 ye	16
-0.00054	-0.00053	7.974645	0.37949	-0.9252	0.003664	0.395188	Over 10 ye	16
-0.00317	-0.00758	8.285575	0.381979	-0.92417	0.008867	0.385166	Over 10 ye	17
-0.00317	-0.00758	8.285575	0.381979	-0.92417	0.008867	0.385166	Over 10 ye	17
-0.00317	-0.00758	8.285575	0.381979	-0.92417	0.008867	0.385166	Over 10 ye	17
-0.00317	-0.00758	8.285575	0.381979	-0.92417	0.008867	0.385166	Over 10 ye	17
-0.00108	-0.00118	9.110424	0.81459	-0.58004	0.043763	0.373741	2 to 5 year	24
-0.00108	-0.00118	9.110424	0.81459	-0.58004	0.043763	0.373741	2 to 5 year	24
-0.00108	-0.00118	9.110424	0.81459	-0.58004	0.043763	0.373741	2 to 5 year	24
-0.00108	-0.00118	9.110424	0.81459	-0.58004	0.043763	0.373741	2 to 5 year	24
-0.00108	-0.00118	9.110424	0.81459	-0.58004	0.043763	0.373741	2 to 5 year	24
-0.00108	-0.00118	9.110424	0.81459	-0.58004	0.043763	0.373741	2 to 5 year	24
-0.00108	-0.00118	9.110424	0.81459	-0.58004	0.043763	0.373741	2 to 5 year	24
-0.00108	-0.00118	9.110424	0.81459	-0.58004	0.043763	0.373741	2 to 5 year	24
-0.00108	-0.00118	9.110424	0.81459	-0.58004	0.043763	0.373741	2 to 5 year	24
-0.00108	-0.00118	9.110424	0.81459	-0.58004	0.043763	0.373741	2 to 5 year	24
0.000928	0.000315	7.07586	-0.01795	-0.99984	0.047869	0.34643	5 to 10 yea	26
0.000928	0.000315	7.07586	-0.01795	-0.99984	0.047869	0.34643	5 to 10 yea	26
0.000928	0.000315	7.07586	-0.01795	-0.99984	0.047869	0.34643	5 to 10 yea	26
0.000928	0.000315	7.07586	-0.01795	-0.99984	0.047869	0.34643	5 to 10 yea	26
-0.00069	-0.00049	8.958983	0.132123	-0.99123	0.044799	0.359558	Over 10 ye	34
-0.00069	-0.00049	8.958983	0.132123	-0.99123	0.044799	0.359558	Over 10 ye	34
-0.00069	-0.00049	8.958983	0.132123	-0.99123	0.044799	0.359558	Over 10 ye	34
-0.00069	-0.00049	8.958983	0.132123	-0.99123	0.044799	0.359558	Over 10 ye	34
-0.00069	-0.00049	8.958983	0.132123	-0.99123	0.044799	0.359558	Over 10 ye	34
-0.00069	-0.00049	8.958983	0.132123	-0.99123	0.044799	0.359558	Over 10 ye	34
-0.00069	-0.00049	8.958983	0.132123	-0.99123	0.044799	0.359558	Over 10 ye	34
-0.00069	-0.00049	8.958983	0.132123	-0.99123	0.044799	0.359558	Over 10 ye	34
-0.00069	-0.00049	8.958983	0.132123	-0.99123	0.044799	0.359558	Over 10 ye	34
-0.00069	-0.00114	8.010088	0.5936	-0.80476	0.003718	0.39511	Over 10 ye	15
-0.0004	0.000957	6.641533	0.442236	0.896899	0.005228	0.388217	2 to 5 year	20
-0.00133	5.61E-05	8.335838	-0.93209	-0.36222	0.036897	0.350057	5 to 10 yea	12
0.000137	-0.00059	9.942574	0.071324	0.997453	0.021988	0.380222	5 to 10 yea	2
-0.00032	-0.00022	7.557192	0.915366	-0.40262	0.00028	0.423083	Over 10 ye	5
0.002679	0.000302	7.512269	0.558958	-0.8292	0.002882	0.388022	2 to 5 year	8
-0.00133	5.61E-05	8.335838	-0.93209	-0.36222	0.036897	0.350057	5 to 10 yea	12
-4.84E-05	0.000152	7.918477	-0.79487	0.606782	0.003819	0.382867	2 to 5 year	3
-0.00062	0.002157	6.203242	0.859966	0.510351	0.005228	0.388217	2 to 5 year	25
0.000858	-0.00035	6.141755	-0.20772	0.978188	0.004976	0.409187	2 to 5 year	2
-0.00024	-0.00318	12.51608	-0.20785	0.978162	0.000148	0.401935	Over 10 ye	5
-0.00062	0.002157	6.203242	0.859966	0.510351	0.005228	0.388217	2 to 5 year	25
-0.00062	0.002157	6.203242	0.859966	0.510351	0.005228	0.388217	2 to 5 year	25
0.000253	2.23E-05	9.858754	0.137937	-0.99044	0.003718	0.399419	2 to 5 year	16
-0.00091	0.000146	11.52142	0.881388	-0.47239	0.021245	0.375289	5 to 10 yea	12
-0.00266	-0.00119	7.505739	0.449955	-0.89305	0.019117	0.376947	2 to 5 year	3

Table S2 - Anonymized database

-0.00062	0.002157	6.203242	0.859966	0.510351	0.005228	0.388217	2 to 5 year	25
5.80E-07	-0.00012	10.36644	0.996778	-0.08021	0.002177	0.390538	Over 10 ye	7
-0.0004	0.000957	6.641533	0.442236	0.896899	0.005228	0.388217	2 to 5 year	20
0.000332	-0.00016	8.166043	0.84661	-0.53221	0.035441	0.339374	Less than 1	1
-0.00014	-2.45E-05	8.419302	-0.99468	-0.10297	0.006977	0.395521	Over 10 ye	6
-0.00064	-0.00037	8.810884	0.159075	-0.98727	0.017465	0.406714	2 to 5 year	8
0.002679	0.000302	7.512269	0.558958	-0.8292	0.002882	0.388022	2 to 5 year	8
-0.0004	0.000957	6.641533	0.442236	0.896899	0.005228	0.388217	2 to 5 year	20
0.000193	0.000456	7.759312	0.949024	0.315205	0.001225	0.387706	Over 10 ye	2
-0.00041	-0.00111	11.81479	-0.34196	-0.93972	0.02326	0.372225	5 to 10 yea	8
-0.00041	-0.00111	11.81479	-0.34196	-0.93972	0.02326	0.372225	5 to 10 yea	8
0.002679	0.000302	7.512269	0.558958	-0.8292	0.002882	0.388022	2 to 5 year	8
0.000181	0.001625	6.488424	-0.71545	-0.69866	0.008097	0.387316	1 to 2 year	9
-0.00266	-0.00119	7.505739	0.449955	-0.89305	0.019117	0.376947	2 to 5 year	3
-0.00133	5.61E-05	8.335838	-0.93209	-0.36222	0.036897	0.350057	5 to 10 yea	12
-0.00062	0.002157	6.203242	0.859966	0.510351	0.005228	0.388217	2 to 5 year	25
0.000693	0.000295	7.874253	-0.26448	0.964392	0.029475	0.379598	1 to 2 year	1
0.004048	7.36E-05	5.468233	-0.63031	0.776346	0.088383	0.284658	1 to 2 year	2
0.000122	0.000508	6.87462	-0.92133	0.38879	0.004402	0.396521	2 to 5 year	2
-0.00131	-0.00066	7.994784	-0.99629	0.086062	0.063094	0.314198	Less than 1	2
5.80E-07	-0.00012	10.36644	0.996778	-0.08021	0.002177	0.390538	Over 10 ye	7
0.000181	0.001625	6.488424	-0.71545	-0.69866	0.008097	0.387316	1 to 2 year	9
-0.00138	-0.00091	7.085806	-0.68539	-0.72818	0.000877	0.41725	1 to 2 year	2
-0.00069	-0.00114	8.010088	0.5936	-0.80476	0.003718	0.39511	Over 10 ye	15
0.001051	0.00104	6.225508	0.022104	0.999756	0.005495	0.401035	2 to 5 year	2
-0.00014	-2.45E-05	8.419302	-0.99468	-0.10297	0.006977	0.395521	Over 10 ye	6
-0.0004	0.000957	6.641533	0.442236	0.896899	0.005228	0.388217	2 to 5 year	20
-0.00131	-0.00066	7.994784	-0.99629	0.086062	0.063094	0.314198	Less than 1	2
0.001139	0.000826	8.643519	0.917286	0.398228	3.06E-05	0.404539	2 to 5 year	10
-0.00064	-0.00037	8.810884	0.159075	-0.98727	0.017465	0.406714	2 to 5 year	8
-0.00062	0.002157	6.203242	0.859966	0.510351	0.005228	0.388217	2 to 5 year	25
-0.00091	0.000146	11.52142	0.881388	-0.47239	0.021245	0.375289	5 to 10 yea	12
0.002881	0.000422	5.561703	0.998678	0.051401	0.000468	0.416233	5 to 10 yea	8
0.001387	0.002705	5.654704	0.266069	-0.96395	0.017398	0.354443	Less than 1	2
-3.60E-05	-8.79E-05	8.242848	-0.22782	0.973704	0.0006	0.432857	2 to 5 year	5
-0.00091	0.000146	11.52142	0.881388	-0.47239	0.021245	0.375289	5 to 10 yea	12
-0.00011	0.000304	7.088681	-0.39936	0.916795	6.15E-05	0.413402	5 to 10 yea	4
0.000181	0.001625	6.488424	-0.71545	-0.69866	0.008097	0.387316	1 to 2 year	9
-0.00133	5.61E-05	8.335838	-0.93209	-0.36222	0.036897	0.350057	5 to 10 yea	12
-0.0004	0.000957	6.641533	0.442236	0.896899	0.005228	0.388217	2 to 5 year	20
-0.00037	0.001131	7.66582	-0.27043	0.962741	0.010208	0.415073	5 to 10 yea	5
0.000212	-0.00112	11.00994	-0.21897	-0.97573	0.004866	0.386107	5 to 10 yea	1
0.001139	0.000826	8.643519	0.917286	0.398228	3.06E-05	0.404539	2 to 5 year	10
-3.60E-05	-8.79E-05	8.242848	-0.22782	0.973704	0.0006	0.432857	2 to 5 year	5
-0.0002	0.001013	6.953256	0.983781	0.179375	0.002878	0.393284	5 to 10 yea	15
-0.00062	0.002157	6.203242	0.859966	0.510351	0.005228	0.388217	2 to 5 year	25
0.000181	-0.00068	9.671714	-0.74085	0.67167	0.002675	0.401647	1 to 2 year	2
0.00045	0.001567	6.19803	0.377834	-0.92587	0.001788	0.397825	Over 10 ye	11
0.000253	2.23E-05	9.858754	0.137937	-0.99044	0.003718	0.399419	2 to 5 year	16
-0.00124	0.000302	9.16566	-0.97215	-0.23437	0.075947	0.300423	2 to 5 year	7

Table S2 - Anonymized database

-0.00266	-0.00119	7.505739	0.449955	-0.89305	0.019117	0.376947	2 to 5 year	3
0.001387	0.002705	5.654704	0.266069	-0.96395	0.017398	0.354443	Less than 1	2
-0.00062	0.002157	6.203242	0.859966	0.510351	0.005228	0.388217	2 to 5 year	25
0.00045	0.001567	6.19803	0.377834	-0.92587	0.001788	0.397825	Over 10 ye	11
-0.00133	5.61E-05	8.335838	-0.93209	-0.36222	0.036897	0.350057	5 to 10 yea	12
5.87E-05	-0.00011	9.120533	-0.22296	0.974829	0.037514	0.339528	2 to 5 year	3
0.000253	2.23E-05	9.858754	0.137937	-0.99044	0.003718	0.399419	2 to 5 year	16
-0.00062	0.002157	6.203242	0.859966	0.510351	0.005228	0.388217	2 to 5 year	25
-0.00062	0.002157	6.203242	0.859966	0.510351	0.005228	0.388217	2 to 5 year	25
0.002881	0.000422	5.561703	0.998678	0.051401	0.000468	0.416233	5 to 10 yea	8
-0.00064	-0.00037	8.810884	0.159075	-0.98727	0.017465	0.406714	2 to 5 year	8
0.000253	2.23E-05	9.858754	0.137937	-0.99044	0.003718	0.399419	2 to 5 year	16
-0.00019	0.000372	7.982374	0.981559	0.191161	0.011815	0.379323	5 to 10 yea	5
0.001139	0.000826	8.643519	0.917286	0.398228	3.06E-05	0.404539	2 to 5 year	10
-0.00064	-0.00037	8.810884	0.159075	-0.98727	0.017465	0.406714	2 to 5 year	8
0.002881	0.000422	5.561703	0.998678	0.051401	0.000468	0.416233	5 to 10 yea	8
-0.00019	0.000372	7.982374	0.981559	0.191161	0.011815	0.379323	5 to 10 yea	5
0.002881	0.000422	5.561703	0.998678	0.051401	0.000468	0.416233	5 to 10 yea	8
-0.00062	0.002157	6.203242	0.859966	0.510351	0.005228	0.388217	2 to 5 year	25
-0.0002	0.001013	6.953256	0.983781	0.179375	0.002878	0.393284	5 to 10 yea	15
-0.0004	0.000957	6.641533	0.442236	0.896899	0.005228	0.388217	2 to 5 year	20
0.000253	2.23E-05	9.858754	0.137937	-0.99044	0.003718	0.399419	2 to 5 year	16
-0.00124	0.000302	9.16566	-0.97215	-0.23437	0.075947	0.300423	2 to 5 year	7
0.001139	0.000826	8.643519	0.917286	0.398228	3.06E-05	0.404539	2 to 5 year	10
-0.0004	0.000957	6.641533	0.442236	0.896899	0.005228	0.388217	2 to 5 year	20
-0.00037	0.001131	7.66582	-0.27043	0.962741	0.010208	0.415073	5 to 10 yea	5
9.08E-05	0.00013	7.812431	0.274433	0.961606	0.093409	0.287989	2 to 5 year	3
-3.60E-05	-8.79E-05	8.242848	-0.22782	0.973704	0.0006	0.432857	2 to 5 year	5
0.000253	2.23E-05	9.858754	0.137937	-0.99044	0.003718	0.399419	2 to 5 year	16
0.000253	2.23E-05	9.858754	0.137937	-0.99044	0.003718	0.399419	2 to 5 year	16
-0.00124	0.000302	9.16566	-0.97215	-0.23437	0.075947	0.300423	2 to 5 year	7
-0.00091	0.000146	11.52142	0.881388	-0.47239	0.021245	0.375289	5 to 10 yea	12
-0.00099	-0.00155	15.27797	-0.98557	0.169284	0.000484	0.429518	Over 10 ye	4
0.000181	-0.00068	9.671714	-0.74085	0.67167	0.002675	0.401647	1 to 2 year	2
0.000253	2.23E-05	9.858754	0.137937	-0.99044	0.003718	0.399419	2 to 5 year	16
0.000193	0.000456	7.759312	0.949024	0.315205	0.001225	0.387706	Over 10 ye	2
0.00074	-2.47E-05	7.907915	0.246254	0.969205	0.000527	0.425985	1 to 2 year	1
-0.0002	0.001013	6.953256	0.983781	0.179375	0.002878	0.393284	5 to 10 yea	15
0.00092	0.000939	6.476176	-0.28295	-0.95913	0.000145	0.407446	1 to 2 year	1
-0.00091	0.000146	11.52142	0.881388	-0.47239	0.021245	0.375289	5 to 10 yea	12
0.000253	2.23E-05	9.858754	0.137937	-0.99044	0.003718	0.399419	2 to 5 year	16
-8.50E-05	0.000397	7.945985	0.140679	0.990055	0.033448	0.351633	5 to 10 yea	7
0.002881	0.000422	5.561703	0.998678	0.051401	0.000468	0.416233	5 to 10 yea	8
-0.00091	0.000146	11.52142	0.881388	-0.47239	0.021245	0.375289	5 to 10 yea	12
-0.00069	-0.00114	8.010088	0.5936	-0.80476	0.003718	0.39511	Over 10 ye	15
-8.50E-05	0.000397	7.945985	0.140679	0.990055	0.033448	0.351633	5 to 10 yea	7
0.000718	-1.35E-05	7.385127	-0.73872	0.67401	0.005736	0.423036	2 to 5 year	3
-0.00133	5.61E-05	8.335838	-0.93209	-0.36222	0.036897	0.350057	5 to 10 yea	12
-0.00064	-0.00037	8.810884	0.159075	-0.98727	0.017465	0.406714	2 to 5 year	8
0.002679	0.000302	7.512269	0.558958	-0.8292	0.002882	0.388022	2 to 5 year	8

Table S2 - Anonymized database

0.000718	-1.35E-05	7.385127	-0.73872	0.67401	0.005736	0.423036	2 to 5 year	3
-0.00124	0.000302	9.16566	-0.97215	-0.23437	0.075947	0.300423	2 to 5 year	7
-3.60E-05	-8.79E-05	8.242848	-0.22782	0.973704	0.0006	0.432857	2 to 5 year	5
-0.0002	0.001013	6.953256	0.983781	0.179375	0.002878	0.393284	5 to 10 yea	15
-0.00062	0.002157	6.203242	0.859966	0.510351	0.005228	0.388217	2 to 5 year	25
-0.00062	0.002157	6.203242	0.859966	0.510351	0.005228	0.388217	2 to 5 year	25
-0.00142	0.000344	7.128039	0.597078	-0.80218	0.047731	0.353481	2 to 5 year	4
-0.00091	0.000146	11.52142	0.881388	-0.47239	0.021245	0.375289	5 to 10 yea	12
0.00045	0.001567	6.19803	0.377834	-0.92587	0.001788	0.397825	Over 10 ye	11
5.80E-07	-0.00012	10.36644	0.996778	-0.08021	0.002177	0.390538	Over 10 ye	7
9.37E-05	-0.00029	9.465427	-0.75018	0.661232	0.007897	0.414037	2 to 5 year	1
-0.0004	0.000957	6.641533	0.442236	0.896899	0.005228	0.388217	2 to 5 year	20
5.80E-07	-0.00012	10.36644	0.996778	-0.08021	0.002177	0.390538	Over 10 ye	7
-0.00069	-0.00114	8.010088	0.5936	-0.80476	0.003718	0.39511	Over 10 ye	15
5.80E-07	-0.00012	10.36644	0.996778	-0.08021	0.002177	0.390538	Over 10 ye	7
-0.00133	5.61E-05	8.335838	-0.93209	-0.36222	0.036897	0.350057	5 to 10 yea	12
9.08E-05	0.00013	7.812431	0.274433	0.961606	0.093409	0.287989	2 to 5 year	3
-8.50E-05	0.000397	7.945985	0.140679	0.990055	0.033448	0.351633	5 to 10 yea	7
-0.00069	-0.00114	8.010088	0.5936	-0.80476	0.003718	0.39511	Over 10 ye	15
-0.00041	-0.00111	11.81479	-0.34196	-0.93972	0.02326	0.372225	5 to 10 yea	8
-0.00014	-2.45E-05	8.419302	-0.99468	-0.10297	0.006977	0.395521	Over 10 ye	6
-0.0004	0.000957	6.641533	0.442236	0.896899	0.005228	0.388217	2 to 5 year	20
-0.0002	0.001013	6.953256	0.983781	0.179375	0.002878	0.393284	5 to 10 yea	15
-8.50E-05	0.000397	7.945985	0.140679	0.990055	0.033448	0.351633	5 to 10 yea	7
-0.00037	0.001131	7.66582	-0.27043	0.962741	0.010208	0.415073	5 to 10 yea	5
-0.00069	-0.00114	8.010088	0.5936	-0.80476	0.003718	0.39511	Over 10 ye	15
0.00045	0.001567	6.19803	0.377834	-0.92587	0.001788	0.397825	Over 10 ye	11
-3.39E-05	0.000811	7.181637	0.934402	-0.35622	6.14E-05	0.420754	1 to 2 year	2
0.000253	2.23E-05	9.858754	0.137937	-0.99044	0.003718	0.399419	2 to 5 year	16
0.000181	0.001625	6.488424	-0.71545	-0.69866	0.008097	0.387316	1 to 2 year	9
-0.0004	0.000957	6.641533	0.442236	0.896899	0.005228	0.388217	2 to 5 year	20
-0.00037	0.001131	7.66582	-0.27043	0.962741	0.010208	0.415073	5 to 10 yea	5
-0.00062	0.002157	6.203242	0.859966	0.510351	0.005228	0.388217	2 to 5 year	25
0.001139	0.000826	8.643519	0.917286	0.398228	3.06E-05	0.404539	2 to 5 year	10
-0.00124	0.000302	9.16566	-0.97215	-0.23437	0.075947	0.300423	2 to 5 year	7
-0.00091	0.000146	11.52142	0.881388	-0.47239	0.021245	0.375289	5 to 10 yea	12
-0.00062	0.002157	6.203242	0.859966	0.510351	0.005228	0.388217	2 to 5 year	25
0.00095	-0.00083	6.641782	0.979936	-0.19931	0.052453	0.331113	2 to 5 year	1
-0.00041	-0.00111	11.81479	-0.34196	-0.93972	0.02326	0.372225	5 to 10 yea	8
-0.00024	-0.00318	12.51608	-0.20785	0.978162	0.000148	0.401935	Over 10 ye	5
-0.00014	-2.45E-05	8.419302	-0.99468	-0.10297	0.006977	0.395521	Over 10 ye	6
0.00045	0.001567	6.19803	0.377834	-0.92587	0.001788	0.397825	Over 10 ye	11
6.76E-05	0.000252	8.373786	-0.47368	0.880697	0.067381	0.314345	2 to 5 year	3
-0.0004	0.000957	6.641533	0.442236	0.896899	0.005228	0.388217	2 to 5 year	20
-0.00069	-0.00114	8.010088	0.5936	-0.80476	0.003718	0.39511	Over 10 ye	15
-0.00142	0.000344	7.128039	0.597078	-0.80218	0.047731	0.353481	2 to 5 year	4
0.002881	0.000422	5.561703	0.998678	0.051401	0.000468	0.416233	5 to 10 yea	8
-0.00024	-0.00318	12.51608	-0.20785	0.978162	0.000148	0.401935	Over 10 ye	5
0.000253	2.23E-05	9.858754	0.137937	-0.99044	0.003718	0.399419	2 to 5 year	16
-0.00064	-0.00037	8.810884	0.159075	-0.98727	0.017465	0.406714	2 to 5 year	8

Table S2 - Anonymized database

-0.00142	0.000344	7.128039	0.597078	-0.80218	0.047731	0.353481	2 to 5 year	4
0.000718	-1.35E-05	7.385127	-0.73872	0.67401	0.005736	0.423036	2 to 5 year	3
-0.00069	-0.00114	8.010088	0.5936	-0.80476	0.003718	0.39511	Over 10 ye	15
-0.00062	0.002157	6.203242	0.859966	0.510351	0.005228	0.388217	2 to 5 year	25
0.000181	0.001625	6.488424	-0.71545	-0.69866	0.008097	0.387316	1 to 2 year	9
-0.00014	-2.45E-05	8.419302	-0.99468	-0.10297	0.006977	0.395521	Over 10 ye	6
-0.00039	0.000536	7.561653	0.939061	-0.34375	0.017287	0.36359	2 to 5 year	1
-0.0004	0.000957	6.641533	0.442236	0.896899	0.005228	0.388217	2 to 5 year	20
-0.00037	0.001131	7.66582	-0.27043	0.962741	0.010208	0.415073	5 to 10 yea	5
-0.00062	0.002157	6.203242	0.859966	0.510351	0.005228	0.388217	2 to 5 year	25
-0.00124	0.000302	9.16566	-0.97215	-0.23437	0.075947	0.300423	2 to 5 year	7
-0.00041	-0.00111	11.81479	-0.34196	-0.93972	0.02326	0.372225	5 to 10 yea	8
-0.00032	-0.00022	7.557192	0.915366	-0.40262	0.00028	0.423083	Over 10 ye	5
-0.00099	-0.00155	15.27797	-0.98557	0.169284	0.000484	0.429518	Over 10 ye	4
0.000181	0.001625	6.488424	-0.71545	-0.69866	0.008097	0.387316	1 to 2 year	9
-0.00064	-0.00037	8.810884	0.159075	-0.98727	0.017465	0.406714	2 to 5 year	8
-0.00142	0.000344	7.128039	0.597078	-0.80218	0.047731	0.353481	2 to 5 year	4
-0.00062	0.002157	6.203242	0.859966	0.510351	0.005228	0.388217	2 to 5 year	25
-0.00019	0.000372	7.982374	0.981559	0.191161	0.011815	0.379323	5 to 10 yea	5
0.00045	0.001567	6.19803	0.377834	-0.92587	0.001788	0.397825	Over 10 ye	11
-0.00019	0.000372	7.982374	0.981559	0.191161	0.011815	0.379323	5 to 10 yea	5
-0.00011	0.000304	7.088681	-0.39936	0.916795	6.15E-05	0.413402	5 to 10 yea	4
-0.00091	0.000146	11.52142	0.881388	-0.47239	0.021245	0.375289	5 to 10 yea	12
-3.39E-05	0.000811	7.181637	0.934402	-0.35622	6.14E-05	0.420754	1 to 2 year	2
0.002881	0.000422	5.561703	0.998678	0.051401	0.000468	0.416233	5 to 10 yea	8
-0.00138	-0.00091	7.085806	-0.68539	-0.72818	0.000877	0.41725	1 to 2 year	2
0.000181	0.001625	6.488424	-0.71545	-0.69866	0.008097	0.387316	1 to 2 year	9
0.001139	0.000826	8.643519	0.917286	0.398228	3.06E-05	0.404539	2 to 5 year	10
0.004048	7.36E-05	5.468233	-0.63031	0.776346	0.088383	0.284658	1 to 2 year	2
-0.0004	0.000957	6.641533	0.442236	0.896899	0.005228	0.388217	2 to 5 year	20
0.00045	0.001567	6.19803	0.377834	-0.92587	0.001788	0.397825	Over 10 ye	11
-0.00014	-2.45E-05	8.419302	-0.99468	-0.10297	0.006977	0.395521	Over 10 ye	6
0.000137	-0.00059	9.942574	0.071324	0.997453	0.021988	0.380222	5 to 10 yea	2
6.76E-05	0.000252	8.373786	-0.47368	0.880697	0.067381	0.314345	2 to 5 year	3
-0.00091	0.000146	11.52142	0.881388	-0.47239	0.021245	0.375289	5 to 10 yea	12
-0.00041	-0.00111	11.81479	-0.34196	-0.93972	0.02326	0.372225	5 to 10 yea	8
-0.0002	0.001013	6.953256	0.983781	0.179375	0.002878	0.393284	5 to 10 yea	15
0.002836	-0.00084	7.157466	-0.5676	-0.8233	0.001367	0.396477	Over 10 ye	2
-0.0002	0.001013	6.953256	0.983781	0.179375	0.002878	0.393284	5 to 10 yea	15
0.000961	0.000191	7.336547	0.876566	0.481282	0.011134	0.405673	1 to 2 year	1
-0.0004	0.000957	6.641533	0.442236	0.896899	0.005228	0.388217	5 to 10 yea	26
-0.00048	0.00019	9.078908	0.467368	-0.88406	0.00014	0.39744	2 to 5 year	3
0.001339	-0.00023	7.543291	0.125484	0.992096	0.005514	0.398555	5 to 10 yea	15
0.000891	0.00392	5.304569	0.842642	0.538474	4.06E-05	0.399017	2 to 5 year	9
-0.0004	0.000669	7.70624	-0.99708	0.076361	0.019945	0.383244	2 to 5 year	6
-0.00037	8.18E-05	9.069148	-0.38439	-0.92317	0.000216	0.430965	1 to 2 year	2
0.002697	0.002325	5.800778	0.905334	-0.4247	0.001936	0.399948	Over 10 ye	17
-0.0004	0.000957	6.641533	0.442236	0.896899	0.005228	0.388217	5 to 10 yea	26
-0.00013	2.74E-05	7.615221	-0.22678	0.973947	0.015185	0.373612	5 to 10 yea	7
-0.0004	0.000957	6.641533	0.442236	0.896899	0.005228	0.388217	5 to 10 yea	26

Table S2 - Anonymized database

-0.00064	-0.00037	8.810884	0.159075	-0.98727	0.017465	0.406714	5 to 10 yea	8
-0.00068	2.72E-05	8.409824	0.47124	0.882005	0.039537	0.326279	2 to 5 year	14
0.001339	-0.00023	7.543291	0.125484	0.992096	0.005514	0.398555	5 to 10 yea	15
0.002697	0.002325	5.800778	0.905334	-0.4247	0.001936	0.399948	Over 10 ye	17
-0.00052	-0.00071	8.980148	0.447706	0.894181	0.08958	0.278293	Over 10 ye	28
0.002697	0.002325	5.800778	0.905334	-0.4247	0.001936	0.399948	Over 10 ye	17
-0.00068	2.72E-05	8.409824	0.47124	0.882005	0.039537	0.326279	2 to 5 year	14
-0.00111	-0.00027	10.13836	0.715309	-0.69881	0.036897	0.350057	Over 10 ye	10
0.000443	0.00158	6.237093	-0.99049	0.137557	0.001588	0.360391	Over 10 ye	31
-0.0004	0.000957	6.641533	0.442236	0.896899	0.005228	0.388217	5 to 10 yea	26
0.000722	-0.00228	10.3242	-0.90898	-0.41683	0.002737	0.389425	Less than 1	3
0.000443	0.00158	6.237093	-0.99049	0.137557	0.001588	0.360391	Over 10 ye	31
0.002697	0.002325	5.800778	0.905334	-0.4247	0.001936	0.399948	Over 10 ye	17
-0.00048	0.00019	9.078908	0.467368	-0.88406	0.00014	0.39744	2 to 5 year	3
0.002443	0.009064	4.495414	-0.72388	-0.68993	0.023465	0.383007	1 to 2 year	1
-0.00068	2.72E-05	8.409824	0.47124	0.882005	0.039537	0.326279	2 to 5 year	14
-0.00052	-0.00071	8.980148	0.447706	0.894181	0.08958	0.278293	Over 10 ye	28
0.000443	0.00158	6.237093	-0.99049	0.137557	0.001588	0.360391	Over 10 ye	31
0.000443	0.00158	6.237093	-0.99049	0.137557	0.001588	0.360391	Over 10 ye	31
0.000135	-0.00132	8.828544	0.684457	-0.72905	0.043763	0.373741	2 to 5 year	19
-0.00158	-0.00072	6.94064	-0.32886	0.94438	0.024372	0.374732	Less than 1	2
0.001036	-4.26E-06	6.939217	0.941752	0.336307	0.001393	0.393455	5 to 10 yea	7
0.000406	0.00014	7.523566	0.726085	-0.6876	0.034812	0.339728	2 to 5 year	6
-0.001	0.000891	6.066459	-0.95174	0.30692	0.000202	0.386658	2 to 5 year	2
-0.00068	2.72E-05	8.409824	0.47124	0.882005	0.039537	0.326279	2 to 5 year	14
0.000244	-9.30E-06	8.093132	0.954265	0.298962	0.021249	0.365716	Less than 1	1
-0.0004	0.000957	6.641533	0.442236	0.896899	0.005228	0.388217	5 to 10 yea	26
-0.0017	-0.00024	7.246581	-0.94994	-0.31242	0.001859	0.392238	Over 10 ye	11
-0.00395	-0.00151	8.142526	-0.16303	-0.98662	0.072606	0.311889	2 to 5 year	1
0.000135	-0.00132	8.828544	0.684457	-0.72905	0.043763	0.373741	2 to 5 year	19
0.001036	-4.26E-06	6.939217	0.941752	0.336307	0.001393	0.393455	5 to 10 yea	7
0.001339	-0.00023	7.543291	0.125484	0.992096	0.005514	0.398555	5 to 10 yea	15
-0.00052	-0.00071	8.980148	0.447706	0.894181	0.08958	0.278293	Over 10 ye	28
-0.00068	2.72E-05	8.409824	0.47124	0.882005	0.039537	0.326279	2 to 5 year	14
-0.00052	-0.00071	8.980148	0.447706	0.894181	0.08958	0.278293	Over 10 ye	28
0.00033	0.000413	7.68102	0.622847	0.782343	0.01425	0.411578	Over 10 ye	8
-8.74E-05	0.000909	7.239906	0.181161	-0.98345	0.033777	0.366614	2 to 5 year	11
-0.0017	-0.00024	7.246581	-0.94994	-0.31242	0.001859	0.392238	Over 10 ye	11
0.001296	-0.00068	8.44785	0.663142	0.748494	0.009909	0.387572	2 to 5 year	8
-0.00052	-0.00071	8.980148	0.447706	0.894181	0.08958	0.278293	Over 10 ye	28
0.002697	0.002325	5.800778	0.905334	-0.4247	0.001936	0.399948	Over 10 ye	17
-0.00082	-6.62E-05	7.554388	-0.74691	0.664929	0.012798	0.397134	1 to 2 year	2
0.00033	0.000413	7.68102	0.622847	0.782343	0.01425	0.411578	Over 10 ye	8
-0.00052	-0.00071	8.980148	0.447706	0.894181	0.08958	0.278293	Over 10 ye	28
0.000443	0.00158	6.237093	-0.99049	0.137557	0.001588	0.360391	Over 10 ye	31
-0.00111	-0.00027	10.13836	0.715309	-0.69881	0.036897	0.350057	Over 10 ye	10
0.000443	0.00158	6.237093	-0.99049	0.137557	0.001588	0.360391	Over 10 ye	31
0.001105	0.000415	5.860742	-0.63306	-0.7741	0.000107	0.404435	5 to 10 yea	5
-8.74E-05	0.000909	7.239906	0.181161	-0.98345	0.033777	0.366614	2 to 5 year	11
0.001339	-0.00023	7.543291	0.125484	0.992096	0.005514	0.398555	5 to 10 yea	15

Table S2 - Anonymized database

-0.00013	2.74E-05	7.615221	-0.22678	0.973947	0.015185	0.373612	5 to 10 yea	7
-0.00082	-6.62E-05	7.554388	-0.74691	0.664929	0.012798	0.397134	1 to 2 year	2
-0.00089	-0.00035	7.0788	-0.71109	-0.7031	0.003488	0.403009	2 to 5 year	5
-0.00052	-0.00071	8.980148	0.447706	0.894181	0.08958	0.278293	Over 10 ye	28
0.001036	-4.26E-06	6.939217	0.941752	0.336307	0.001393	0.393455	5 to 10 yea	7
-0.00163	0.000282	6.320958	-0.53334	0.845902	0.065325	0.32561	2 to 5 year	15
0.002697	0.002325	5.800778	0.905334	-0.4247	0.001936	0.399948	Over 10 ye	17
0.000452	0.000842	6.690324	-0.85015	-0.52654	0.032563	0.348585	1 to 2 year	3
-0.00089	-0.00035	7.0788	-0.71109	-0.7031	0.003488	0.403009	2 to 5 year	5
0.000443	0.00158	6.237093	-0.99049	0.137557	0.001588	0.360391	Over 10 ye	31
-0.00068	2.72E-05	8.409824	0.47124	0.882005	0.039537	0.326279	2 to 5 year	14
0.001105	0.000415	5.860742	-0.63306	-0.7741	0.000107	0.404435	5 to 10 yea	5
-0.00064	-0.00037	8.810884	0.159075	-0.98727	0.017465	0.406714	5 to 10 yea	8
0.000131	-0.00029	9.292435	-0.10971	0.993964	0.037466	0.339227	Over 10 ye	3
0.000258	0.000345	7.474024	0.731652	0.681678	0.008124	0.414248	Less than 1	1
0.001036	-4.26E-06	6.939217	0.941752	0.336307	0.001393	0.393455	5 to 10 yea	7
-0.00154	-0.00157	9.944622	-0.94558	-0.32538	0.008966	0.390204	1 to 2 year	2
0.001036	-4.26E-06	6.939217	0.941752	0.336307	0.001393	0.393455	5 to 10 yea	7
0.001353	0.000184	6.482762	-0.35969	0.933073	0.020249	0.388149	Over 10 ye	2
0.000891	0.00392	5.304569	0.842642	0.538474	4.06E-05	0.399017	2 to 5 year	9
0.000135	-0.00132	8.828544	0.684457	-0.72905	0.043763	0.373741	2 to 5 year	19
-0.00138	-0.00091	7.085806	-0.68539	-0.72818	0.000877	0.41725	1 to 2 year	3
0.004535	-0.00052	5.5207	0.989703	-0.14314	0.000434	0.426494	2 to 5 year	2
0.000452	-0.00043	8.325767	-0.38681	-0.92216	0.000667	0.38089	Less than 1	2
0.000443	0.00158	6.237093	-0.99049	0.137557	0.001588	0.360391	Over 10 ye	31
0.001353	0.000184	6.482762	-0.35969	0.933073	0.020249	0.388149	Over 10 ye	2
0.000135	-0.00132	8.828544	0.684457	-0.72905	0.043763	0.373741	2 to 5 year	19
0.001339	-0.00023	7.543291	0.125484	0.992096	0.005514	0.398555	5 to 10 yea	15
-0.00052	-0.00071	8.980148	0.447706	0.894181	0.08958	0.278293	Over 10 ye	28
0.002697	0.002325	5.800778	0.905334	-0.4247	0.001936	0.399948	Over 10 ye	17
-0.00093	0.000257	8.140813	0.99914	-0.04147	0.048512	0.34648	2 to 5 year	3
-8.74E-05	0.000909	7.239906	0.181161	-0.98345	0.033777	0.366614	2 to 5 year	11
-0.00163	0.000282	6.320958	-0.53334	0.845902	0.065325	0.32561	2 to 5 year	15
-0.00066	0.000356	7.34828	0.48702	-0.87339	0.004815	0.391996	2 to 5 year	5
0.00033	0.000413	7.68102	0.622847	0.782343	0.01425	0.411578	Over 10 ye	8
-0.00163	0.000282	6.320958	-0.53334	0.845902	0.065325	0.32561	2 to 5 year	15
-7.18E-05	-1.56E-05	7.911486	-0.3367	-0.94161	0.000646	0.422779	5 to 10 yea	9
-0.00163	0.000282	6.320958	-0.53334	0.845902	0.065325	0.32561	2 to 5 year	15
0.000443	0.00158	6.237093	-0.99049	0.137557	0.001588	0.360391	Over 10 ye	31
-0.0004	0.000957	6.641533	0.442236	0.896899	0.005228	0.388217	5 to 10 yea	26
-0.0004	0.000957	6.641533	0.442236	0.896899	0.005228	0.388217	5 to 10 yea	26
-0.0004	0.000957	6.641533	0.442236	0.896899	0.005228	0.388217	5 to 10 yea	26
0.001036	-4.26E-06	6.939217	0.941752	0.336307	0.001393	0.393455	5 to 10 yea	7
0.001339	-0.00023	7.543291	0.125484	0.992096	0.005514	0.398555	5 to 10 yea	15
0.000123	0.000242	9.155404	0.103996	-0.99458	0.013909	0.384906	2 to 5 year	1
0.000443	0.00158	6.237093	-0.99049	0.137557	0.001588	0.360391	Over 10 ye	31
-0.00013	2.74E-05	7.615221	-0.22678	0.973947	0.015185	0.373612	5 to 10 yea	7
-0.001	0.000891	6.066459	-0.95174	0.30692	0.000202	0.386658	2 to 5 year	2
0.002697	0.002325	5.800778	0.905334	-0.4247	0.001936	0.399948	Over 10 ye	17
-0.0017	-0.00024	7.246581	-0.94994	-0.31242	0.001859	0.392238	Over 10 ye	11

Table S2 - Anonymized database

0.000443	0.00158	6.237093	-0.99049	0.137557	0.001588	0.360391	Over 10 ye	31
0.001339	-0.00023	7.543291	0.125484	0.992096	0.005514	0.398555	5 to 10 yea	15
0.000443	0.00158	6.237093	-0.99049	0.137557	0.001588	0.360391	Over 10 ye	31
-0.00089	-0.00035	7.0788	-0.71109	-0.7031	0.003488	0.403009	2 to 5 year	5
-0.00163	0.000282	6.320958	-0.53334	0.845902	0.065325	0.32561	2 to 5 year	15
-7.18E-05	-1.56E-05	7.911486	-0.3367	-0.94161	0.000646	0.422779	5 to 10 yea	9
2.64E-05	0.000763	5.972576	0.988796	0.149274	0.012535	0.405665	1 to 2 year	1
-0.00064	-0.00037	8.810884	0.159075	-0.98727	0.017465	0.406714	5 to 10 yea	8
-0.00064	-0.00037	8.810884	0.159075	-0.98727	0.017465	0.406714	5 to 10 yea	8
-7.18E-05	-1.56E-05	7.911486	-0.3367	-0.94161	0.000646	0.422779	5 to 10 yea	9
-0.0004	0.000957	6.641533	0.442236	0.896899	0.005228	0.388217	5 to 10 yea	26
0.004773	0.002721	5.106052	-0.97229	0.23379	5.76E-05	0.3733	2 to 5 year	4
-8.74E-05	0.000909	7.239906	0.181161	-0.98345	0.033777	0.366614	2 to 5 year	11
-0.0017	-0.00024	7.246581	-0.94994	-0.31242	0.001859	0.392238	Over 10 ye	11
-0.00026	0.000462	8.163028	0.284725	-0.95861	0.024897	0.373615	1 to 2 year	2
0.000443	0.00158	6.237093	-0.99049	0.137557	0.001588	0.360391	Over 10 ye	31
0.002697	0.002325	5.800778	0.905334	-0.4247	0.001936	0.399948	Over 10 ye	17
0.001576	0.000457	6.224496	0.630085	0.776526	0.044078	0.371762	1 to 2 year	2
-0.00068	2.72E-05	8.409824	0.47124	0.882005	0.039537	0.326279	2 to 5 year	14
0.000135	-0.00132	8.828544	0.684457	-0.72905	0.043763	0.373741	2 to 5 year	19
-0.0004	0.000957	6.641533	0.442236	0.896899	0.005228	0.388217	5 to 10 yea	26
-0.00052	-0.00071	8.980148	0.447706	0.894181	0.08958	0.278293	Over 10 ye	28
-0.00138	-0.00091	7.085806	-0.68539	-0.72818	0.000877	0.41725	1 to 2 year	3
-0.00052	-0.00071	8.980148	0.447706	0.894181	0.08958	0.278293	Over 10 ye	28
0.001105	0.000415	5.860742	-0.63306	-0.7741	0.000107	0.404435	5 to 10 yea	5
-0.00111	-0.00027	10.13836	0.715309	-0.69881	0.036897	0.350057	Over 10 ye	10
0.000443	0.00158	6.237093	-0.99049	0.137557	0.001588	0.360391	Over 10 ye	31
0.00033	0.000413	7.68102	0.622847	0.782343	0.01425	0.411578	Over 10 ye	8
-0.00052	-0.00071	8.980148	0.447706	0.894181	0.08958	0.278293	Over 10 ye	28
-7.18E-05	-1.56E-05	7.911486	-0.3367	-0.94161	0.000646	0.422779	5 to 10 yea	9
-0.0004	0.000957	6.641533	0.442236	0.896899	0.005228	0.388217	5 to 10 yea	26
-0.0004	0.000669	7.70624	-0.99708	0.076361	0.019945	0.383244	2 to 5 year	6
-0.00052	-0.00071	8.980148	0.447706	0.894181	0.08958	0.278293	Over 10 ye	28
0.000443	0.00158	6.237093	-0.99049	0.137557	0.001588	0.360391	Over 10 ye	31
0.000443	0.00158	6.237093	-0.99049	0.137557	0.001588	0.360391	Over 10 ye	31
-0.00066	0.000356	7.34828	0.48702	-0.87339	0.004815	0.391996	2 to 5 year	5
0.001036	-4.26E-06	6.939217	0.941752	0.336307	0.001393	0.393455	5 to 10 yea	7
0.000443	0.00158	6.237093	-0.99049	0.137557	0.001588	0.360391	Over 10 ye	31
0.000393	0.000719	7.13387	-0.98446	-0.17562	0.046343	0.330844	1 to 2 year	2
-0.00052	-0.00071	8.980148	0.447706	0.894181	0.08958	0.278293	Over 10 ye	28
-0.00032	0.00039	7.819598	-0.16045	0.987043	0.013231	0.403303	2 to 5 year	2
0.000135	-0.00132	8.828544	0.684457	-0.72905	0.043763	0.373741	2 to 5 year	19
0.000135	-0.00132	8.828544	0.684457	-0.72905	0.043763	0.373741	2 to 5 year	19
0.001339	-0.00023	7.543291	0.125484	0.992096	0.005514	0.398555	5 to 10 yea	15
0.000443	0.00158	6.237093	-0.99049	0.137557	0.001588	0.360391	Over 10 ye	31
0.001339	-0.00023	7.543291	0.125484	0.992096	0.005514	0.398555	5 to 10 yea	15
-7.18E-05	-1.56E-05	7.911486	-0.3367	-0.94161	0.000646	0.422779	5 to 10 yea	9
0.000452	0.000842	6.690324	-0.85015	-0.52654	0.032563	0.348585	1 to 2 year	3
0.001339	-0.00023	7.543291	0.125484	0.992096	0.005514	0.398555	5 to 10 yea	15
0.000443	0.00158	6.237093	-0.99049	0.137557	0.001588	0.360391	Over 10 ye	31

Table S2 - Anonymized database

-0.00052	-0.00071	8.980148	0.447706	0.894181	0.08958	0.278293	Over 10 ye	28
-7.18E-05	-1.56E-05	7.911486	-0.3367	-0.94161	0.000646	0.422779	5 to 10 yea	9
0.004773	0.002721	5.106052	-0.97229	0.23379	5.76E-05	0.3733	2 to 5 year	4
-0.0017	-0.00024	7.246581	-0.94994	-0.31242	0.001859	0.392238	Over 10 ye	11
-0.00052	-0.00071	8.980148	0.447706	0.894181	0.08958	0.278293	Over 10 ye	28
-0.00052	-0.00071	8.980148	0.447706	0.894181	0.08958	0.278293	Over 10 ye	28
0.001296	-0.00068	8.44785	0.663142	0.748494	0.009909	0.387572	2 to 5 year	8
-0.00089	-0.00035	7.0788	-0.71109	-0.7031	0.003488	0.403009	2 to 5 year	5
-8.74E-05	0.000909	7.239906	0.181161	-0.98345	0.033777	0.366614	2 to 5 year	11
0.004773	0.002721	5.106052	-0.97229	0.23379	5.76E-05	0.3733	2 to 5 year	4
-0.00163	0.000282	6.320958	-0.53334	0.845902	0.065325	0.32561	2 to 5 year	15
0.000135	-0.00132	8.828544	0.684457	-0.72905	0.043763	0.373741	2 to 5 year	19
-0.0024	-0.00012	11.78486	0.988351	-0.15219	0.013105	0.383563	1 to 2 year	1
0.000891	0.00392	5.304569	0.842642	0.538474	4.06E-05	0.399017	2 to 5 year	9
0.00033	0.000413	7.68102	0.622847	0.782343	0.01425	0.411578	Over 10 ye	8
0.000722	-0.00228	10.3242	-0.90898	-0.41683	0.002737	0.389425	Less than 1	3
0.000406	0.00014	7.523566	0.726085	-0.6876	0.034812	0.339728	2 to 5 year	6
-0.0004	0.000669	7.70624	-0.99708	0.076361	0.019945	0.383244	2 to 5 year	6
-0.00052	-0.00071	8.980148	0.447706	0.894181	0.08958	0.278293	Over 10 ye	28
-0.00547	-0.00471	9.736187	-0.29985	-0.95399	0.005529	0.407736	5 to 10 yea	5
0.001296	-0.00068	8.44785	0.663142	0.748494	0.009909	0.387572	2 to 5 year	8
-0.00138	-0.00091	7.085806	-0.68539	-0.72818	0.000877	0.41725	1 to 2 year	3
-0.00064	-0.00037	8.810884	0.159075	-0.98727	0.017465	0.406714	5 to 10 yea	8
-0.00052	-0.00071	8.980148	0.447706	0.894181	0.08958	0.278293	Over 10 ye	28
-0.0004	0.000957	6.641533	0.442236	0.896899	0.005228	0.388217	5 to 10 yea	26
0.000393	0.000719	7.13387	-0.98446	-0.17562	0.046343	0.330844	1 to 2 year	2
0.001105	0.000415	5.860742	-0.63306	-0.7741	0.000107	0.404435	5 to 10 yea	5
-0.00163	0.000282	6.320958	-0.53334	0.845902	0.065325	0.32561	2 to 5 year	15
0.000135	-0.00132	8.828544	0.684457	-0.72905	0.043763	0.373741	2 to 5 year	19
0.001296	-0.00068	8.44785	0.663142	0.748494	0.009909	0.387572	2 to 5 year	8
0.001296	-0.00068	8.44785	0.663142	0.748494	0.009909	0.387572	2 to 5 year	8
-8.74E-05	0.000909	7.239906	0.181161	-0.98345	0.033777	0.366614	2 to 5 year	11
0.004535	-0.00052	5.5207	0.989703	-0.14314	0.000434	0.426494	2 to 5 year	2
-0.0004	0.000957	6.641533	0.442236	0.896899	0.005228	0.388217	5 to 10 yea	26
0.000406	0.00014	7.523566	0.726085	-0.6876	0.034812	0.339728	2 to 5 year	6
0.000443	0.00158	6.237093	-0.99049	0.137557	0.001588	0.360391	Over 10 ye	31
0.002697	0.002325	5.800778	0.905334	-0.4247	0.001936	0.399948	Over 10 ye	17
-0.00068	2.72E-05	8.409824	0.47124	0.882005	0.039537	0.326279	2 to 5 year	14
0.001296	-0.00068	8.44785	0.663142	0.748494	0.009909	0.387572	2 to 5 year	8
-0.00163	0.000282	6.320958	-0.53334	0.845902	0.065325	0.32561	2 to 5 year	15
0.00033	0.000413	7.68102	0.622847	0.782343	0.01425	0.411578	Over 10 ye	8
0.000443	0.00158	6.237093	-0.99049	0.137557	0.001588	0.360391	Over 10 ye	31
-0.0004	0.000669	7.70624	-0.99708	0.076361	0.019945	0.383244	2 to 5 year	6
-0.0004	0.000957	6.641533	0.442236	0.896899	0.005228	0.388217	5 to 10 yea	26
-0.00111	-0.00027	10.13836	0.715309	-0.69881	0.036897	0.350057	Over 10 ye	10
0.000443	0.00158	6.237093	-0.99049	0.137557	0.001588	0.360391	Over 10 ye	31
-0.0004	0.000957	6.641533	0.442236	0.896899	0.005228	0.388217	5 to 10 yea	26
-0.00052	-0.00071	8.980148	0.447706	0.894181	0.08958	0.278293	Over 10 ye	28
-0.0004	0.000957	6.641533	0.442236	0.896899	0.005228	0.388217	5 to 10 yea	26
-0.0004	0.000957	6.641533	0.442236	0.896899	0.005228	0.388217	5 to 10 yea	26

Table S2 - Anonymized database

0.000891	0.00392	5.304569	0.842642	0.538474	4.06E-05	0.399017	2 to 5 year	9
-0.00068	2.72E-05	8.409824	0.47124	0.882005	0.039537	0.326279	2 to 5 year	14
0.001339	-0.00023	7.543291	0.125484	0.992096	0.005514	0.398555	5 to 10 yea	15
-8.74E-05	0.000909	7.239906	0.181161	-0.98345	0.033777	0.366614	2 to 5 year	11
-0.00163	0.000282	6.320958	-0.53334	0.845902	0.065325	0.32561	2 to 5 year	15
0.000443	0.00158	6.237093	-0.99049	0.137557	0.001588	0.360391	Over 10 ye	31
-0.00048	0.00019	9.078908	0.467368	-0.88406	0.00014	0.39744	2 to 5 year	3
0.000891	0.00392	5.304569	0.842642	0.538474	4.06E-05	0.399017	2 to 5 year	9
-0.00068	2.72E-05	8.409824	0.47124	0.882005	0.039537	0.326279	2 to 5 year	14
0.00022	-0.00022	7.385865	0.999496	0.031747	0.062085	0.318606	5 to 10 yea	1
0.000333	0.000143	7.315743	0.993855	-0.11069	0.055491	0.279209	2 to 5 year	3
0.000443	0.00158	6.237093	-0.99049	0.137557	0.001588	0.360391	Over 10 ye	31
-0.0004	0.000957	6.641533	0.442236	0.896899	0.005228	0.388217	5 to 10 yea	26
0.001339	-0.00023	7.543291	0.125484	0.992096	0.005514	0.398555	5 to 10 yea	15
-0.00027	0.00029	7.187155	0.29133	-0.95662	0.027795	0.37041	Less than 1	2
-0.00052	-0.00071	8.980148	0.447706	0.894181	0.08958	0.278293	Over 10 ye	28
-0.00068	2.72E-05	8.409824	0.47124	0.882005	0.039537	0.326279	2 to 5 year	14
-0.00052	-0.00071	8.980148	0.447706	0.894181	0.08958	0.278293	Over 10 ye	28
-0.00163	0.000282	6.320958	-0.53334	0.845902	0.065325	0.32561	2 to 5 year	15
-0.0004	0.000957	6.641533	0.442236	0.896899	0.005228	0.388217	5 to 10 yea	26
-0.0004	0.000957	6.641533	0.442236	0.896899	0.005228	0.388217	5 to 10 yea	26
-0.00052	-0.00071	8.980148	0.447706	0.894181	0.08958	0.278293	Over 10 ye	28
0.000135	-0.00132	8.828544	0.684457	-0.72905	0.043763	0.373741	2 to 5 year	19
0.000135	-0.00132	8.828544	0.684457	-0.72905	0.043763	0.373741	2 to 5 year	19
-0.00547	-0.00471	9.736187	-0.29985	-0.95399	0.005529	0.407736	5 to 10 yea	5
0.001296	-0.00068	8.44785	0.663142	0.748494	0.009909	0.387572	2 to 5 year	8
-0.00052	-0.00071	8.980148	0.447706	0.894181	0.08958	0.278293	Over 10 ye	28
-0.0017	-0.00024	7.246581	-0.94994	-0.31242	0.001859	0.392238	Over 10 ye	11
-0.00163	0.000282	6.320958	-0.53334	0.845902	0.065325	0.32561	2 to 5 year	15
-0.00111	-0.00027	10.13836	0.715309	-0.69881	0.036897	0.350057	Over 10 ye	10
0.004773	0.002721	5.106052	-0.97229	0.23379	5.76E-05	0.3733	2 to 5 year	4
0.000406	0.00014	7.523566	0.726085	-0.6876	0.034812	0.339728	2 to 5 year	6
-0.00064	-0.00037	8.810884	0.159075	-0.98727	0.017465	0.406714	5 to 10 yea	8
0.000891	0.00392	5.304569	0.842642	0.538474	4.06E-05	0.399017	2 to 5 year	9
-0.0017	-0.00024	7.246581	-0.94994	-0.31242	0.001859	0.392238	Over 10 ye	11
0.001547	0.000517	6.168585	0.008704	0.999962	0.001499	0.425114	5 to 10 yea	3
-0.00045	-0.0004	9.139278	-0.97586	-0.21837	0.009431	0.390315	2 to 5 year	20
0.000872	0.000622	7.015728	-0.62153	-0.78339	0.011947	0.403054	1 to 2 year	2
-0.00019	0.000252	9.080857	-0.15098	0.988537	0.023393	0.371974	Over 10 ye	10
0.001131	0.00063	7.54068	0.908182	-0.41858	0.339062	0.343287	5 to 10 yea	5
0.000754	3.40E-05	6.545462	0.443489	-0.89628	0.014039	0.374165	Over 10 ye	2
0.001105	0.000415	5.860742	-0.63306	-0.7741	0.000107	0.404435	5 to 10 yea	7
-0.00149	-0.00148	8.976081	-0.75992	0.650019	0.009797	0.373252	2 to 5 year	8
-0.00023	0.000294	7.396209	-0.85928	0.511503	0.102343	0.287067	5 to 10 yea	13
-0.00037	0.000636	6.427949	-0.7724	-0.63513	0.003948	0.402743	Over 10 ye	11
-3.90E-05	-0.00066	10.38832	-0.13929	-0.99025	0.046155	0.323061	Over 10 ye	15
0.002444	-0.00025	6.068515	-0.99992	-0.01246	0.00288	0.395734	2 to 5 year	4
-0.00054	-0.00053	7.974645	0.37949	-0.9252	0.003664	0.395188	Over 10 ye	16
-0.002	-0.0005	7.043015	-0.99784	-0.06576	0.002133	0.399649	2 to 5 year	6
-0.00324	-9.00E-04	12.5425	-0.65231	0.757956	0.052029	0.311606	2 to 5 year	3

Table S2 - Anonymized database

3.00E-06	-0.00061	12.34398	0.695892	0.718146	3.18E-05	0.406127	2 to 5 year	3
-0.00069	-0.00049	8.958983	0.132123	-0.99123	0.044799	0.359558	Over 10 ye	34
0.000585	-9.20E-05	7.951361	0.67058	0.741837	0.039557	0.340828	2 to 5 year	5
0.000373	0.000481	7.152483	-0.42747	-0.90403	0.016893	0.387055	2 to 5 year	11
-0.00054	0.000921	7.719733	0.996977	-0.0777	0.024062	0.382715	5 to 10 yea	1
-0.00181	-3.80E-05	11.36418	-0.63714	0.770751	0.001584	0.411519	5 to 10 yea	5
2.80E-05	0.000158	7.935713	0.986909	-0.16128	0.01295	0.403363	5 to 10 yea	6
0.000928	0.000315	7.07586	-0.01795	-0.99984	0.047869	0.34643	5 to 10 yea	26
0.000827	0.000962	6.420393	-0.99141	-0.13081	0.001287	0.426577	1 to 2 year	3
-0.00351	-0.00493	9.698227	0.9886	-0.15057	0.05117	0.320754	5 to 10 yea	10
0.001535	0.000484	6.717156	-0.14995	-0.98869	0.058258	0.334687	1 to 2 year	2
0.001039	0	7.701547	0.757136	-0.65326	0.030647	0.366727	5 to 10 yea	3
-0.00028	0.000453	6.854851	0.797983	0.60268	0.009312	0.395292	2 to 5 year	16
-0.00037	0.000395	7.243243	-0.01524	0.999884	0.037988	0.325697	2 to 5 year	6
-0.00351	-0.00493	9.698227	0.9886	-0.15057	0.05117	0.320754	5 to 10 yea	10
0.002295	0.00551	4.841191	-0.19558	0.980687	0.006205	0.387621	5 to 10 yea	10
0.000928	0.000315	7.07586	-0.01795	-0.99984	0.047869	0.34643	5 to 10 yea	26
-3.90E-05	-0.00066	10.38832	-0.13929	-0.99025	0.046155	0.323061	Over 10 ye	15
-0.00019	0.000252	9.080857	-0.15098	0.988537	0.023393	0.371974	Over 10 ye	10
0.00364	0.001644	5.660378	-0.83004	-0.55771	0.062955	0.326097	1 to 2 year	5
-0.00125	-0.00089	6.507705	0.751696	-0.65951	0.059097	0.317329	2 to 5 year	8
0.000112	0.000241	7.972994	0.449417	0.893322	0.002402	0.419275	2 to 5 year	7
0.003221	0.001054	5.788663	0.94556	0.325447	0.005833	0.395439	2 to 5 year	8
-3.40E-05	-0.00087	9.206155	-0.75518	0.65552	0.021835	0.387149	5 to 10 yea	6
3.30E-05	0.000103	7.984262	0.497598	-0.86741	0.004843	0.391127	5 to 10 yea	11
-0.00075	0.000153	8.199074	0.444781	-0.89564	0.002887	0.393638	2 to 5 year	1
-0.00149	-0.00148	8.976081	-0.75992	0.650019	0.009797	0.373252	2 to 5 year	8
-1.40E-05	3.80E-05	9.22682	0.72213	0.691757	6.65E-07	0.412395	2 to 5 year	3
-0.00023	0.000294	7.396209	-0.85928	0.511503	0.102343	0.287067	5 to 10 yea	13
0.000125	0.000106	8.09158	-0.47147	-0.88188	0.001293	0.395439	5 to 10 yea	12
0.000928	0.000315	7.07586	-0.01795	-0.99984	0.047869	0.34643	5 to 10 yea	26
-0.00149	-0.00148	8.976081	-0.75992	0.650019	0.009797	0.373252	2 to 5 year	8
0.000125	0.000106	8.09158	-0.47147	-0.88188	0.001293	0.395439	5 to 10 yea	12
0.000928	0.000315	7.07586	-0.01795	-0.99984	0.047869	0.34643	5 to 10 yea	26
-3.90E-05	-0.00066	10.38832	-0.13929	-0.99025	0.046155	0.323061	Over 10 ye	15
0.002565	0.000252	7.01588	-0.81028	-0.58604	0.008547	0.386603	5 to 10 yea	8
-0.00351	-0.00493	9.698227	0.9886	-0.15057	0.05117	0.320754	5 to 10 yea	10
0.000125	0.000106	8.09158	-0.47147	-0.88188	0.001293	0.395439	5 to 10 yea	12
-0.00037	0.000395	7.243243	-0.01524	0.999884	0.037988	0.325697	2 to 5 year	6
0.00096	9.00E-05	6.846452	0.995135	-0.09852	0.035441	0.339374	2 to 5 year	5
0.0011	-0.00039	9.418569	0.243551	-0.96989	0.002535	0.408747	2 to 5 year	6
-0.00048	-0.00042	8.983812	-0.95159	0.307365	0.001385	0.40293	2 to 5 year	5
3.30E-05	0.000103	7.984262	0.497598	-0.86741	0.004843	0.391127	5 to 10 yea	11
-0.00069	-0.00049	8.958983	0.132123	-0.99123	0.044799	0.359558	Over 10 ye	34
-0.00037	0.000636	6.427949	-0.7724	-0.63513	0.003948	0.402743	Over 10 ye	11
-3.90E-05	-0.00066	10.38832	-0.13929	-0.99025	0.046155	0.323061	Over 10 ye	15
-0.00087	-0.00049	8.240852	0.281379	-0.9596	0.004232	0.388678	1 to 2 year	4
0.000928	0.000315	7.07586	-0.01795	-0.99984	0.047869	0.34643	5 to 10 yea	26
-0.00317	-0.00758	8.285575	0.381979	-0.92417	0.008867	0.385166	Over 10 ye	17
0.000928	0.000315	7.07586	-0.01795	-0.99984	0.047869	0.34643	5 to 10 yea	26

Table S2 - Anonymized database

0.000524	-0.00082	7.77387	-0.9394	-0.34282	0.019976	0.370704	2 to 5 year	3
-0.00069	-0.00049	8.958983	0.132123	-0.99123	0.044799	0.359558	Over 10 ye	34
-0.00054	-0.00053	7.974645	0.37949	-0.9252	0.003664	0.395188	Over 10 ye	16
-0.00019	0.000252	9.080857	-0.15098	0.988537	0.023393	0.371974	Over 10 ye	10
-0.00023	0.000294	7.396209	-0.85928	0.511503	0.102343	0.287067	5 to 10 yea	13
0.002139	-0.00178	6.95804	-0.13969	0.990196	0.009714	0.385306	1 to 2 year	7
-0.00069	-0.00049	8.958983	0.132123	-0.99123	0.044799	0.359558	Over 10 ye	34
0.000699	-0.00033	7.434845	-0.42134	-0.9069	0.036834	0.356765	2 to 5 year	2
-0.00181	-3.80E-05	11.36418	-0.63714	0.770751	0.001584	0.411519	5 to 10 yea	5
3.70E-05	0.000344	6.792089	-0.99023	0.139451	0.012965	0.387951	2 to 5 year	12
0.000242	-4.50E-05	7.396961	-0.96834	0.249643	0.024769	0.391333	1 to 2 year	3
-0.00037	0.000636	6.427949	-0.7724	-0.63513	0.003948	0.402743	Over 10 ye	11
-0.00029	-6.10E-05	10.92574	0.009512	-0.99995	0.040339	0.342214	2 to 5 year	8
-0.00043	0.000222	6.91947	-0.59886	-0.80085	0.003814	0.39002	2 to 5 year	4
0.000928	0.000315	7.07586	-0.01795	-0.99984	0.047869	0.34643	5 to 10 yea	26
-0.00045	-0.0004	9.139278	-0.97586	-0.21837	0.009431	0.390315	2 to 5 year	20
-0.00037	0.000636	6.427949	-0.7724	-0.63513	0.003948	0.402743	Over 10 ye	11
0.002295	0.00551	4.841191	-0.19558	0.980687	0.006205	0.387621	5 to 10 yea	10
-0.00351	-0.00493	9.698227	0.9886	-0.15057	0.05117	0.320754	5 to 10 yea	10
-0.00317	-0.00758	8.285575	0.381979	-0.92417	0.008867	0.385166	Over 10 ye	17
-0.00354	-0.0001	7.882977	0.95887	-0.28384	0.000103	0.423653	1 to 2 year	12
-0.00013	0.000117	9.363936	-0.85537	-0.51802	0.068087	0.336834	2 to 5 year	5
-0.00018	0.000166	9.809126	-0.98811	-0.15374	0.017034	0.383016	2 to 5 year	4
-0.00317	-0.00758	8.285575	0.381979	-0.92417	0.008867	0.385166	Over 10 ye	17
0.000928	0.000315	7.07586	-0.01795	-0.99984	0.047869	0.34643	5 to 10 yea	26
0.000179	-0.00019	9.676757	0.818019	0.575191	0.014334	0.395231	Over 10 ye	7
0.002565	0.000252	7.01588	-0.81028	-0.58604	0.008547	0.386603	5 to 10 yea	8
0.000928	0.000315	7.07586	-0.01795	-0.99984	0.047869	0.34643	5 to 10 yea	26
-0.00061	-0.00019	8.427426	-0.53394	0.845524	0.000184	0.415632	2 to 5 year	3
-0.00351	-0.00493	9.698227	0.9886	-0.15057	0.05117	0.320754	5 to 10 yea	10
-0.00069	-0.00049	8.958983	0.132123	-0.99123	0.044799	0.359558	Over 10 ye	34
-0.00125	-0.00089	6.507705	0.751696	-0.65951	0.059097	0.317329	2 to 5 year	8
0.002295	0.00551	4.841191	-0.19558	0.980687	0.006205	0.387621	5 to 10 yea	10
-0.00108	-0.00118	9.110424	0.81459	-0.58004	0.043763	0.373741	2 to 5 year	24
-0.00069	-0.00049	8.958983	0.132123	-0.99123	0.044799	0.359558	Over 10 ye	34
-0.00018	0.000577	7.314416	0.452999	0.891511	0.078283	0.307638	5 to 10 yea	11
3.30E-05	0.000103	7.984262	0.497598	-0.86741	0.004843	0.391127	5 to 10 yea	11
-0.00069	-0.00049	8.958983	0.132123	-0.99123	0.044799	0.359558	Over 10 ye	34
0.000179	-0.00019	9.676757	0.818019	0.575191	0.014334	0.395231	Over 10 ye	7
-0.00276	0.000517	8.721258	-0.29706	-0.95486	0.057471	0.335248	2 to 5 year	3
0.008767	0.001926	4.580526	0.724124	-0.68967	0.001702	0.420842	2 to 5 year	2
-0.00014	0.000165	10.64129	0.875599	-0.48304	0.003819	0.382867	2 to 5 year	5
2.80E-05	0.000158	7.935713	0.986909	-0.16128	0.01295	0.403363	5 to 10 yea	6
-0.00069	-0.00049	8.958983	0.132123	-0.99123	0.044799	0.359558	Over 10 ye	34
-0.00019	-0.00194	11.11409	-0.99996	0.009151	9.26E-06	0.432676	Over 10 ye	1
0.00096	9.00E-05	6.846452	0.995135	-0.09852	0.035441	0.339374	2 to 5 year	5
-0.00023	0.000294	7.396209	-0.85928	0.511503	0.102343	0.287067	5 to 10 yea	13
0.00033	0.000413	7.68102	0.622847	0.782343	0.01425	0.411578	Over 10 ye	14
0.000928	0.000315	7.07586	-0.01795	-0.99984	0.047869	0.34643	5 to 10 yea	26
0.000179	-0.00019	9.676757	0.818019	0.575191	0.014334	0.395231	Over 10 ye	7

Table S2 - Anonymized database

-9.40E-05	0.000331	7.918806	-0.84816	0.529745	0.002653	0.416487	1 to 2 year	2
8.00E-06	-1.60E-05	13.27315	0.748272	-0.66339	8.31E-07	0.311308	2 to 5 year	3
-0.002	-0.0005	7.043015	-0.99784	-0.06576	0.002133	0.399649	2 to 5 year	6
-0.0006	0.001251	7.111355	-0.90249	0.430702	0.002676	0.421914	5 to 10 yea	7
-0.0006	0.001251	7.111355	-0.90249	0.430702	0.002676	0.421914	5 to 10 yea	7
-0.00029	-6.10E-05	10.92574	0.009512	-0.99995	0.040339	0.342214	2 to 5 year	8
0.001447	0.000749	5.42723	0.013815	0.999905	0.000165	0.401891	Over 10 ye	5
0.000265	-0.00173	8.467335	0.624563	-0.78097	0.032531	0.341907	2 to 5 year	9
-0.00317	-0.00758	8.285575	0.381979	-0.92417	0.008867	0.385166	Over 10 ye	17
0.000404	-0.00101	6.550444	-0.98501	0.172514	0.000392	0.415925	5 to 10 yea	9
0.000112	0.000241	7.972994	0.449417	0.893322	0.002402	0.419275	2 to 5 year	7
0.000585	-9.20E-05	7.951361	0.67058	0.741837	0.039557	0.340828	2 to 5 year	5
8.00E-06	-1.60E-05	13.27315	0.748272	-0.66339	8.31E-07	0.311308	2 to 5 year	3
-3.90E-05	-0.00066	10.38832	-0.13929	-0.99025	0.046155	0.323061	Over 10 ye	15
-0.00048	-0.00042	10.2381	-0.54978	0.835311	0.021104	0.374728	2 to 5 year	3
0.001105	0.000415	5.860742	-0.63306	-0.7741	0.000107	0.404435	5 to 10 yea	7
-0.00045	-0.0004	9.139278	-0.97586	-0.21837	0.009431	0.390315	2 to 5 year	20
-0.00317	-0.00758	8.285575	0.381979	-0.92417	0.008867	0.385166	Over 10 ye	17
-0.0006	0.001251	7.111355	-0.90249	0.430702	0.002676	0.421914	5 to 10 yea	7
0.00033	0.000413	7.68102	0.622847	0.782343	0.01425	0.411578	Over 10 ye	14
-0.00149	-0.00148	8.976081	-0.75992	0.650019	0.009797	0.373252	2 to 5 year	8
-0.00317	-0.00758	8.285575	0.381979	-0.92417	0.008867	0.385166	Over 10 ye	17
0.00096	0.005301	5.114022	0.944001	-0.32994	4.06E-05	0.399017	5 to 10 yea	10
0.00364	0.001644	5.660378	-0.83004	-0.55771	0.062955	0.326097	1 to 2 year	5
-6.90E-05	0.000241	7.275188	0.854634	-0.51923	0.007868	0.389344	2 to 5 year	2
-0.00054	-0.00053	7.974645	0.37949	-0.9252	0.003664	0.395188	Over 10 ye	16
0.000705	0.000384	6.483116	-0.75141	0.659836	0.016717	0.333957	Over 10 ye	9
0.000545	-0.00015	7.788049	0.711975	-0.70221	0.079883	0.293708	2 to 5 year	7
0.003221	0.001054	5.788663	0.94556	0.325447	0.005833	0.395439	2 to 5 year	8
-0.00063	-6.20E-05	8.821591	-0.96326	-0.26858	0.050648	0.295265	2 to 5 year	7
-0.00351	-0.00493	9.698227	0.9886	-0.15057	0.05117	0.320754	5 to 10 yea	10
0.000322	-4.70E-05	9.028348	-0.99976	-0.02187	2.64E-05	0.421283	Less than 1	1
-0.00899	0.000177	8.424847	-0.31745	0.948276	0.010962	0.400156	1 to 2 year	2
0.002565	0.000252	7.01588	-0.81028	-0.58604	0.008547	0.386603	5 to 10 yea	8
-0.00069	-0.00049	8.958983	0.132123	-0.99123	0.044799	0.359558	Over 10 ye	34
-0.00276	0.000517	8.721258	-0.29706	-0.95486	0.057471	0.335248	2 to 5 year	3
-0.00126	0.000191	7.233391	0.378898	0.925438	0.008101	0.393838	1 to 2 year	6
-0.00028	0.000453	6.854851	0.797983	0.60268	0.009312	0.395292	2 to 5 year	16
0.001039	0	7.701547	0.757136	-0.65326	0.030647	0.366727	5 to 10 yea	3
-1.40E-05	3.80E-05	9.22682	0.72213	0.691757	6.65E-07	0.412395	2 to 5 year	3
-0.00354	-0.0001	7.882977	0.95887	-0.28384	0.000103	0.423653	1 to 2 year	12
-0.00201	6.60E-05	14.15868	0.97946	-0.20164	0.020628	0.384548	2 to 5 year	3
-0.00037	0.000636	6.427949	-0.7724	-0.63513	0.003948	0.402743	Over 10 ye	11
0.000373	0.000481	7.152483	-0.42747	-0.90403	0.016893	0.387055	2 to 5 year	11
0.004227	0.002337	5.757184	-0.80577	-0.59222	0.000278	0.402242	Over 10 ye	7
3.70E-05	0.000344	6.792089	-0.99023	0.139451	0.012965	0.387951	2 to 5 year	12
0.0011	-0.00039	9.418569	0.243551	-0.96989	0.002535	0.408747	2 to 5 year	6
-0.00249	-0.00183	7.791164	0.833364	0.552724	0.000654	0.433276	5 to 10 yea	6
0.000373	0.000481	7.152483	-0.42747	-0.90403	0.016893	0.387055	2 to 5 year	11
-0.00028	0.000453	6.854851	0.797983	0.60268	0.009312	0.395292	2 to 5 year	16

Table S2 - Anonymized database

-0.00126	0.000191	7.233391	0.378898	0.925438	0.008101	0.393838	1 to 2 year	6
0.001072	0.000296	8.071708	0.069514	-0.99758	0.024897	0.373615	2 to 5 year	3
0.000125	0.000106	8.09158	-0.47147	-0.88188	0.001293	0.395439	5 to 10 yea	12
-0.00201	6.60E-05	14.15868	0.97946	-0.20164	0.020628	0.384548	2 to 5 year	3
-0.00091	-0.00011	7.181785	0.96959	0.244737	0.003353	0.400542	2 to 5 year	5
-0.00317	-0.00758	8.285575	0.381979	-0.92417	0.008867	0.385166	Over 10 ye	17
0.000125	0.000106	8.09158	-0.47147	-0.88188	0.001293	0.395439	5 to 10 yea	12
-0.00019	-0.00348	9.576899	-0.63885	0.769334	0.001559	0.412951	1 to 2 year	2
0.0011	-0.00039	9.418569	0.243551	-0.96989	0.002535	0.408747	2 to 5 year	6
0.000125	0.000106	8.09158	-0.47147	-0.88188	0.001293	0.395439	5 to 10 yea	12
-0.00354	-0.0001	7.882977	0.95887	-0.28384	0.000103	0.423653	1 to 2 year	12
-0.00069	-0.00049	8.958983	0.132123	-0.99123	0.044799	0.359558	Over 10 ye	34
2.80E-05	0.000158	7.935713	0.986909	-0.16128	0.01295	0.403363	5 to 10 yea	6
-7.20E-05	-4.70E-05	11.12294	0.04965	0.998767	0.103332	0.286539	Over 10 ye	12
-0.00045	-0.0004	9.139278	-0.97586	-0.21837	0.009431	0.390315	2 to 5 year	20
0.000928	0.000315	7.07586	-0.01795	-0.99984	0.047869	0.34643	5 to 10 yea	26
0.003221	0.001054	5.788663	0.94556	0.325447	0.005833	0.395439	2 to 5 year	8
-0.00108	-0.00118	9.110424	0.81459	-0.58004	0.043763	0.373741	2 to 5 year	24
0.003221	0.001054	5.788663	0.94556	0.325447	0.005833	0.395439	2 to 5 year	8
-0.00125	-0.00089	6.507705	0.751696	-0.65951	0.059097	0.317329	2 to 5 year	8
-0.00249	-0.00183	7.791164	0.833364	0.552724	0.000654	0.433276	5 to 10 yea	6
-0.00125	-0.00089	6.507705	0.751696	-0.65951	0.059097	0.317329	2 to 5 year	8
-0.00107	-5.70E-05	7.900374	-0.21527	-0.97655	0.002407	0.37604	5 to 10 yea	2
0.000382	-0.00059	12.14705	0.290701	0.956814	0.001242	0.395773	1 to 2 year	11
8.70E-05	2.70E-05	6.958606	-0.76012	-0.64978	0.003537	0.38875	Over 10 ye	3
-0.00045	-0.0004	9.139278	-0.97586	-0.21837	0.009431	0.390315	2 to 5 year	20
-0.00253	-7.40E-05	13.57139	-0.22779	0.97371	0.032885	0.357136	1 to 2 year	1
0.000928	0.000315	7.07586	-0.01795	-0.99984	0.047869	0.34643	5 to 10 yea	26
0.001133	0.000546	6.975701	-0.65862	0.752475	0.004293	0.399878	5 to 10 yea	4
0.000754	3.40E-05	6.545462	0.443489	-0.89628	0.014039	0.374165	Over 10 ye	2
-0.00014	0.000165	10.64129	0.875599	-0.48304	0.003819	0.382867	2 to 5 year	5
3.70E-05	0.000344	6.792089	-0.99023	0.139451	0.012965	0.387951	2 to 5 year	12
-0.00019	-0.00033	9.448408	0.474164	0.880436	1.61E-05	0.406384	1 to 2 year	1
0.000918	0.000957	7.354772	0.678338	0.73475	0.014464	0.321976	Less than 1	2
0.000524	-0.00082	7.77387	-0.9394	-0.34282	0.019976	0.370704	2 to 5 year	3
-0.00054	-0.00053	7.974645	0.37949	-0.9252	0.003664	0.395188	Over 10 ye	16
0.000486	0.000696	7.21388	-0.99999	0.004943	0.003964	0.413122	5 to 10 yea	4
-0.00045	-0.0004	9.139278	-0.97586	-0.21837	0.009431	0.390315	2 to 5 year	20
-0.00181	-3.80E-05	11.36418	-0.63714	0.770751	0.001584	0.411519	5 to 10 yea	5
-0.00069	-0.00049	8.958983	0.132123	-0.99123	0.044799	0.359558	Over 10 ye	34
0.001447	0.000749	5.42723	0.013815	0.999905	0.000165	0.401891	Over 10 ye	5
0.000705	0.000384	6.483116	-0.75141	0.659836	0.016717	0.333957	Over 10 ye	9
-0.00899	0.000177	8.424847	-0.31745	0.948276	0.010962	0.400156	1 to 2 year	2
-0.00023	0.000294	7.396209	-0.85928	0.511503	0.102343	0.287067	5 to 10 yea	13
0.008767	0.001926	4.580526	0.724124	-0.68967	0.001702	0.420842	2 to 5 year	2
-0.00354	-0.0001	7.882977	0.95887	-0.28384	0.000103	0.423653	1 to 2 year	12
3.70E-05	0.000344	6.792089	-0.99023	0.139451	0.012965	0.387951	2 to 5 year	12
0.000382	-0.00059	12.14705	0.290701	0.956814	0.001242	0.395773	1 to 2 year	11
-0.00597	-0.00256	9.492289	0.701878	-0.7123	0.000216	0.430965	2 to 5 year	2
0.00364	0.001644	5.660378	-0.83004	-0.55771	0.062955	0.326097	1 to 2 year	5

Table S2 - Anonymized database

-0.00054	-0.00053	7.974645	0.37949	-0.9252	0.003664	0.395188	Over 10 ye	16
-0.00031	0.000414	6.752109	-0.48257	-0.87585	0.000372	0.396149	2 to 5 year	4
0.000437	0.00135	6.904528	0.998935	0.04614	0.000293	0.425109	1 to 2 year	2
-0.00011	-0.00023	10.92632	0.31408	-0.9494	0.064053	0.313069	Over 10 ye	5
0.000384	0.000594	7.174697	-0.35256	0.935788	0.042672	0.359447	2 to 5 year	5
0.000708	0.000582	7.037695	0.990504	0.137486	0.014558	0.400028	Over 10 ye	7
-0.00125	-0.00089	6.507705	0.751696	-0.65951	0.059097	0.317329	2 to 5 year	8
0.000524	-0.00082	7.77387	-0.9394	-0.34282	0.019976	0.370704	2 to 5 year	3
0.00096	0.005301	5.114022	0.944001	-0.32994	4.06E-05	0.399017	5 to 10 yea	10
-0.00028	-0.00062	10.31837	-0.92986	-0.3679	0.062812	0.32094	2 to 5 year	5
0.000108	1.90E-05	10.09791	0.863383	0.50455	0.003253	0.431537	2 to 5 year	3
-4.00E-05	0.000166	6.602221	-0.24178	0.97033	0.042414	0.328016	2 to 5 year	5
0.000382	-0.00059	12.14705	0.290701	0.956814	0.001242	0.395773	1 to 2 year	11
-0.00023	0.000294	7.396209	-0.85928	0.511503	0.102343	0.287067	5 to 10 yea	13
-0.00107	-5.70E-05	7.900374	-0.21527	-0.97655	0.002407	0.37604	5 to 10 yea	2
-9.40E-05	0.000331	7.918806	-0.84816	0.529745	0.002653	0.416487	1 to 2 year	2
-0.00045	-0.0004	9.139278	-0.97586	-0.21837	0.009431	0.390315	2 to 5 year	20
0.000404	-0.00101	6.550444	-0.98501	0.172514	0.000392	0.415925	5 to 10 yea	9
-0.0005	0.00166	6.079113	-0.63624	-0.77149	0.009163	0.405865	1 to 2 year	2
-0.00028	0.000453	6.854851	0.797983	0.60268	0.009312	0.395292	2 to 5 year	16
0.000827	0.000962	6.420393	-0.99141	-0.13081	0.001287	0.426577	1 to 2 year	3
-0.00108	-0.00118	9.110424	0.81459	-0.58004	0.043763	0.373741	2 to 5 year	24
0.00033	0.000413	7.68102	0.622847	0.782343	0.01425	0.411578	Over 10 ye	14
-0.00103	-0.0005	9.234109	-0.53885	0.842403	0.077446	0.322683	2 to 5 year	2
-0.00243	-0.00465	7.259941	0.896445	0.443156	6.97E-05	0.422515	2 to 5 year	2
-0.00069	-0.00049	8.958983	0.132123	-0.99123	0.044799	0.359558	Over 10 ye	34
0.000263	0.000325	7.37233	0.554118	0.832438	0.035376	0.346586	1 to 2 year	1
0.002295	0.00551	4.841191	-0.19558	0.980687	0.006205	0.387621	5 to 10 yea	10
-0.00029	-6.10E-05	10.92574	0.009512	-0.99995	0.040339	0.342214	2 to 5 year	8
-0.00037	0.000636	6.427949	-0.7724	-0.63513	0.003948	0.402743	Over 10 ye	11
-0.0006	0.001251	7.111355	-0.90249	0.430702	0.002676	0.421914	5 to 10 yea	7
-7.20E-05	-4.70E-05	11.12294	0.04965	0.998767	0.103332	0.286539	Over 10 ye	12
-0.00054	-0.00053	7.974645	0.37949	-0.9252	0.003664	0.395188	Over 10 ye	16
0.004227	0.002337	5.757184	-0.80577	-0.59222	0.000278	0.402242	Over 10 ye	7
-0.00108	-0.00118	9.110424	0.81459	-0.58004	0.043763	0.373741	2 to 5 year	24
0.000486	0.000696	7.21388	-0.99999	0.004943	0.003964	0.413122	5 to 10 yea	4
0.001447	0.000749	5.42723	0.013815	0.999905	0.000165	0.401891	Over 10 ye	5
-0.00069	-0.00049	8.958983	0.132123	-0.99123	0.044799	0.359558	Over 10 ye	34
0.000486	0.000696	7.21388	-0.99999	0.004943	0.003964	0.413122	5 to 10 yea	4
0.000382	-0.00059	12.14705	0.290701	0.956814	0.001242	0.395773	1 to 2 year	11
-0.00028	0.000453	6.854851	0.797983	0.60268	0.009312	0.395292	2 to 5 year	16
0.00033	0.000413	7.68102	0.622847	0.782343	0.01425	0.411578	Over 10 ye	14
0.000373	0.000481	7.152483	-0.42747	-0.90403	0.016893	0.387055	2 to 5 year	11
-0.00019	-0.00348	9.576899	-0.63885	0.769334	0.001559	0.412951	1 to 2 year	2
0.000705	0.000384	6.483116	-0.75141	0.659836	0.016717	0.333957	Over 10 ye	9
-0.00063	-6.20E-05	8.821591	-0.96326	-0.26858	0.050648	0.295265	2 to 5 year	7
-0.00013	0.000117	9.363936	-0.85537	-0.51802	0.068087	0.336834	2 to 5 year	5
0.002301	0.003581	5.215646	0.603308	0.797508	0.01096	0.389974	1 to 2 year	5
-0.00028	0.000453	6.854851	0.797983	0.60268	0.009312	0.395292	2 to 5 year	16
-0.00023	0.000294	7.396209	-0.85928	0.511503	0.102343	0.287067	5 to 10 yea	13

Table S2 - Anonymized database

0.00033	0.000413	7.68102	0.622847	0.782343	0.01425	0.411578	Over 10 ye	14
-0.00108	0.000143	9.864983	-0.52344	0.852061	0.018665	0.380921	2 to 5 year	2
-0.00018	0.000166	9.809126	-0.98811	-0.15374	0.017034	0.383016	2 to 5 year	4
3.70E-05	0.000344	6.792089	-0.99023	0.139451	0.012965	0.387951	2 to 5 year	12
0.000453	0.000402	7.477843	0.861715	-0.50739	0.01583	0.397747	2 to 5 year	3
0.000928	0.000315	7.07586	-0.01795	-0.99984	0.047869	0.34643	5 to 10 yea	26
-0.00317	-0.00758	8.285575	0.381979	-0.92417	0.008867	0.385166	Over 10 ye	17
0.000928	0.000315	7.07586	-0.01795	-0.99984	0.047869	0.34643	5 to 10 yea	26
0.000545	-0.00015	7.788049	0.711975	-0.70221	0.079883	0.293708	2 to 5 year	7
0.000498	0.000841	8.941735	0.823845	-0.56681	0.013135	0.383289	2 to 5 year	2
0.000708	0.000582	7.037695	0.990504	0.137486	0.014558	0.400028	Over 10 ye	7
-7.20E-05	-4.70E-05	11.12294	0.04965	0.998767	0.103332	0.286539	Over 10 ye	12
0.000453	0.000402	7.477843	0.861715	-0.50739	0.01583	0.397747	2 to 5 year	3
0.002295	0.00551	4.841191	-0.19558	0.980687	0.006205	0.387621	5 to 10 yea	10
-0.00023	0.000294	7.396209	-0.85928	0.511503	0.102343	0.287067	5 to 10 yea	13
-0.00045	-0.0004	9.139278	-0.97586	-0.21837	0.009431	0.390315	2 to 5 year	20
-0.00317	-0.00758	8.285575	0.381979	-0.92417	0.008867	0.385166	Over 10 ye	17
-0.00069	-0.00049	8.958983	0.132123	-0.99123	0.044799	0.359558	Over 10 ye	34
0.000265	-0.00173	8.467335	0.624563	-0.78097	0.032531	0.341907	2 to 5 year	9
-0.00108	-0.00118	9.110424	0.81459	-0.58004	0.043763	0.373741	2 to 5 year	24
0.00096	0.005301	5.114022	0.944001	-0.32994	4.06E-05	0.399017	5 to 10 yea	10
0.00033	0.000413	7.68102	0.622847	0.782343	0.01425	0.411578	Over 10 ye	14
0.00033	0.000413	7.68102	0.622847	0.782343	0.01425	0.411578	Over 10 ye	14
0.00033	0.000413	7.68102	0.622847	0.782343	0.01425	0.411578	Over 10 ye	14
0.002295	0.00551	4.841191	-0.19558	0.980687	0.006205	0.387621	5 to 10 yea	10
-0.00046	0.002523	5.318647	0.781001	0.62453	0.042118	0.349723	Over 10 ye	3
-0.00018	0.000577	7.314416	0.452999	0.891511	0.078283	0.307638	5 to 10 yea	11
0.000585	-9.20E-05	7.951361	0.67058	0.741837	0.039557	0.340828	2 to 5 year	5
-1.40E-05	3.80E-05	9.22682	0.72213	0.691757	6.65E-07	0.412395	2 to 5 year	3
-0.00069	-0.00049	8.958983	0.132123	-0.99123	0.044799	0.359558	Over 10 ye	34
0.000708	0.000582	7.037695	0.990504	0.137486	0.014558	0.400028	Over 10 ye	7
3.30E-05	0.000103	7.984262	0.497598	-0.86741	0.004843	0.391127	5 to 10 yea	11
0.000545	-0.00015	7.788049	0.711975	-0.70221	0.079883	0.293708	2 to 5 year	7
-0.00019	0.000252	9.080857	-0.15098	0.988537	0.023393	0.371974	Over 10 ye	10
0.000382	-0.00059	12.14705	0.290701	0.956814	0.001242	0.395773	1 to 2 year	11
0.000928	0.000315	7.07586	-0.01795	-0.99984	0.047869	0.34643	5 to 10 yea	26
-0.00019	0.000252	9.080857	-0.15098	0.988537	0.023393	0.371974	Over 10 ye	10
0.001468	-3.60E-05	6.365498	0.172739	0.984968	0.056563	0.311263	1 to 2 year	2
0.001105	0.000415	5.860742	-0.63306	-0.7741	0.000107	0.404435	5 to 10 yea	7
0.000106	0.000435	10.30726	-0.07783	0.996966	0.000332	0.395629	5 to 10 yea	4
0.000545	-0.00015	7.788049	0.711975	-0.70221	0.079883	0.293708	2 to 5 year	7
0.000928	0.000315	7.07586	-0.01795	-0.99984	0.047869	0.34643	5 to 10 yea	26
0.00096	0.005301	5.114022	0.944001	-0.32994	4.06E-05	0.399017	5 to 10 yea	10
-0.00028	0.000453	6.854851	0.797983	0.60268	0.009312	0.395292	2 to 5 year	16
-0.00237	0.000735	6.755063	0.091776	-0.99578	0.017132	0.354799	2 to 5 year	2
-0.00019	0.000252	9.080857	-0.15098	0.988537	0.023393	0.371974	Over 10 ye	10
-3.90E-05	-0.00066	10.38832	-0.13929	-0.99025	0.046155	0.323061	Over 10 ye	15
-0.00048	-0.00042	8.983812	-0.95159	0.307365	0.001385	0.40293	2 to 5 year	5
-0.00037	0.000395	7.243243	-0.01524	0.999884	0.037988	0.325697	2 to 5 year	6
-7.20E-05	-4.70E-05	11.12294	0.04965	0.998767	0.103332	0.286539	Over 10 ye	12

Table S2 - Anonymized database

-0.00108	-0.00118	9.110424	0.81459	-0.58004	0.043763	0.373741	2 to 5 year	24
0.000404	-0.00101	6.550444	-0.98501	0.172514	0.000392	0.415925	5 to 10 yea	9
0.000705	0.000384	6.483116	-0.75141	0.659836	0.016717	0.333957	Over 10 ye	9
0.000382	-0.00059	12.14705	0.290701	0.956814	0.001242	0.395773	1 to 2 year	11
-0.00013	0.000117	9.363936	-0.85537	-0.51802	0.068087	0.336834	2 to 5 year	5
-0.00102	-0.00061	11.12156	0.96399	0.265938	0.050741	0.34748	1 to 2 year	3
0.000827	0.000962	6.420393	-0.99141	-0.13081	0.001287	0.426577	1 to 2 year	3
-0.00149	-0.00148	8.976081	-0.75992	0.650019	0.009797	0.373252	2 to 5 year	8
0.000425	0.000244	7.423181	-0.58327	0.81228	0.006118	0.419368	1 to 2 year	2
-0.00017	-8.30E-05	10.68071	-0.56871	-0.82254	0.00063	0.42541	5 to 10 yea	2
-0.00108	-0.00118	9.110424	0.81459	-0.58004	0.043763	0.373741	2 to 5 year	24
-0.00018	0.000577	7.314416	0.452999	0.891511	0.078283	0.307638	5 to 10 yea	11
-0.00245	0.000323	7.116082	-0.0026	0.999997	0.00016	0.411031	2 to 5 year	2
0.000928	0.000315	7.07586	-0.01795	-0.99984	0.047869	0.34643	5 to 10 yea	26
-0.00069	-0.00049	8.958983	0.132123	-0.99123	0.044799	0.359558	Over 10 ye	34
8.50E-05	-0.00011	8.182613	0.65839	-0.75268	0.00036	0.432406	2 to 5 year	5
0.001566	-0.00187	6.3935	0.976425	-0.21586	0.018741	0.371319	2 to 5 year	1
-0.00014	0.000165	10.64129	0.875599	-0.48304	0.003819	0.382867	2 to 5 year	5
-0.00011	-0.00023	10.92632	0.31408	-0.9494	0.064053	0.313069	Over 10 ye	5
-3.90E-05	-0.00066	10.38832	-0.13929	-0.99025	0.046155	0.323061	Over 10 ye	15
-0.00245	0.000323	7.116082	-0.0026	0.999997	0.00016	0.411031	2 to 5 year	2
-0.00045	-0.0004	9.139278	-0.97586	-0.21837	0.009431	0.390315	2 to 5 year	20
0.000332	0.001614	5.844646	0.999888	0.014955	0.022942	0.378116	Over 10 ye	4
0.000872	0.000622	7.015728	-0.62153	-0.78339	0.011947	0.403054	1 to 2 year	2
-0.00069	-0.00049	8.958983	0.132123	-0.99123	0.044799	0.359558	Over 10 ye	34
-0.00354	-0.0001	7.882977	0.95887	-0.28384	0.000103	0.423653	1 to 2 year	12
-6.90E-05	0.000241	7.275188	0.854634	-0.51923	0.007868	0.389344	2 to 5 year	2
0.001072	0.000296	8.071708	0.069514	-0.99758	0.024897	0.373615	2 to 5 year	3
0.001133	0.000546	6.975701	-0.65862	0.752475	0.004293	0.399878	5 to 10 yea	4
0.001105	0.000415	5.860742	-0.63306	-0.7741	0.000107	0.404435	5 to 10 yea	7
-0.00317	-0.00758	8.285575	0.381979	-0.92417	0.008867	0.385166	Over 10 ye	17
-0.00108	-0.00118	9.110424	0.81459	-0.58004	0.043763	0.373741	2 to 5 year	24
-3.40E-05	-0.00087	9.206155	-0.75518	0.65552	0.021835	0.387149	5 to 10 yea	6
2.20E-05	-0.00026	8.60884	0.084634	-0.99641	1.55E-05	0.411413	2 to 5 year	9
-0.00317	-0.00758	8.285575	0.381979	-0.92417	0.008867	0.385166	Over 10 ye	17
0.000332	0.001614	5.844646	0.999888	0.014955	0.022942	0.378116	Over 10 ye	4
-7.20E-05	-4.70E-05	11.12294	0.04965	0.998767	0.103332	0.286539	Over 10 ye	12
-0.00028	-0.00062	10.31837	-0.92986	-0.3679	0.062812	0.32094	2 to 5 year	5
0.000437	0.00135	6.904528	0.998935	0.04614	0.000293	0.425109	1 to 2 year	2
-0.00108	-0.00118	9.110424	0.81459	-0.58004	0.043763	0.373741	2 to 5 year	24
0.000928	0.000315	7.07586	-0.01795	-0.99984	0.047869	0.34643	5 to 10 yea	26
2.80E-05	0.000158	7.935713	0.986909	-0.16128	0.01295	0.403363	5 to 10 yea	6
-0.00069	-0.00049	8.958983	0.132123	-0.99123	0.044799	0.359558	Over 10 ye	34
-0.00046	0.002523	5.318647	0.781001	0.62453	0.042118	0.349723	Over 10 ye	3
-0.00028	0.000453	6.854851	0.797983	0.60268	0.009312	0.395292	2 to 5 year	16
0.000373	0.000481	7.152483	-0.42747	-0.90403	0.016893	0.387055	2 to 5 year	11
0.0011	-0.00039	9.418569	0.243551	-0.96989	0.002535	0.408747	2 to 5 year	6
0.000265	-0.00173	8.467335	0.624563	-0.78097	0.032531	0.341907	2 to 5 year	9
-0.00126	0.000191	7.233391	0.378898	0.925438	0.008101	0.393838	1 to 2 year	6
-0.00249	-0.00183	7.791164	0.833364	0.552724	0.000654	0.433276	5 to 10 yea	6

Table S2 - Anonymized database

-0.00069	-0.00049	8.958983	0.132123	-0.99123	0.044799	0.359558	Over 10 ye	34
0.001771	0.001576	6.105651	-0.99955	0.029905	0.000843	0.430229	Over 10 ye	2
0.000545	-0.00015	7.788049	0.711975	-0.70221	0.079883	0.293708	2 to 5 year	7
-3.40E-05	-0.00087	9.206155	-0.75518	0.65552	0.021835	0.387149	5 to 10 yea	6
0.002565	0.000252	7.01588	-0.81028	-0.58604	0.008547	0.386603	5 to 10 yea	8
-0.00018	0.000577	7.314416	0.452999	0.891511	0.078283	0.307638	5 to 10 yea	11
0.000928	0.000315	7.07586	-0.01795	-0.99984	0.047869	0.34643	5 to 10 yea	26
0.000199	-0.00019	8.966571	-0.91646	-0.40012	0.004935	0.377711	5 to 10 yea	2
0.000928	0.000315	7.07586	-0.01795	-0.99984	0.047869	0.34643	5 to 10 yea	26
-0.00037	0.000636	6.427949	-0.7724	-0.63513	0.003948	0.402743	Over 10 ye	11
-0.00171	0.000814	6.917393	0.837894	-0.54583	0.061167	0.312614	1 to 2 year	2
0.001131	0.00063	7.54068	0.908182	-0.41858	0.339062	0.343287	5 to 10 yea	5
-0.00324	-9.00E-04	12.5425	-0.65231	0.757956	0.052029	0.311606	2 to 5 year	3
0.00096	9.00E-05	6.846452	0.995135	-0.09852	0.035441	0.339374	2 to 5 year	5
-0.00317	-0.00758	8.285575	0.381979	-0.92417	0.008867	0.385166	Over 10 ye	17
0.003221	0.001054	5.788663	0.94556	0.325447	0.005833	0.395439	2 to 5 year	8
3.30E-05	0.000103	7.984262	0.497598	-0.86741	0.004843	0.391127	5 to 10 yea	11
0.000699	-0.00033	7.434845	-0.42134	-0.9069	0.036834	0.356765	2 to 5 year	2
0.004227	0.002337	5.757184	-0.80577	-0.59222	0.000278	0.402242	Over 10 ye	7
-0.00018	0.000577	7.314416	0.452999	0.891511	0.078283	0.307638	5 to 10 yea	11
-0.00125	-0.00089	6.507705	0.751696	-0.65951	0.059097	0.317329	2 to 5 year	8
0.000928	0.000315	7.07586	-0.01795	-0.99984	0.047869	0.34643	5 to 10 yea	26
3.30E-05	0.000103	7.984262	0.497598	-0.86741	0.004843	0.391127	5 to 10 yea	11
-0.00023	0.000294	7.396209	-0.85928	0.511503	0.102343	0.287067	5 to 10 yea	13
0.001547	0.000517	6.168585	0.008704	0.999962	0.001499	0.425114	5 to 10 yea	3
3.70E-05	0.000344	6.792089	-0.99023	0.139451	0.012965	0.387951	2 to 5 year	12
0.00033	0.000413	7.68102	0.622847	0.782343	0.01425	0.411578	Over 10 ye	14
-4.00E-05	0.000166	6.602221	-0.24178	0.97033	0.042414	0.328016	2 to 5 year	5
-0.00019	0.000252	9.080857	-0.15098	0.988537	0.023393	0.371974	Over 10 ye	10
0.000108	1.90E-05	10.09791	0.863383	0.50455	0.003253	0.431537	2 to 5 year	3
0.000106	0.000435	10.30726	-0.07783	0.996966	0.000332	0.395629	5 to 10 yea	4
-0.0006	0.001251	7.111355	-0.90249	0.430702	0.002676	0.421914	5 to 10 yea	7
-0.00011	-0.00023	10.92632	0.31408	-0.9494	0.064053	0.313069	Over 10 ye	5
0.002139	-0.00178	6.95804	-0.13969	0.990196	0.009714	0.385306	1 to 2 year	7
-0.00029	-6.10E-05	10.92574	0.009512	-0.99995	0.040339	0.342214	2 to 5 year	8
-0.00028	0.000453	6.854851	0.797983	0.60268	0.009312	0.395292	2 to 5 year	16
-3.40E-05	-0.00087	9.206155	-0.75518	0.65552	0.021835	0.387149	5 to 10 yea	6
0.001072	0.000296	8.071708	0.069514	-0.99758	0.024897	0.373615	2 to 5 year	3
-0.00045	-0.0004	9.139278	-0.97586	-0.21837	0.009431	0.390315	2 to 5 year	20
0.004227	0.002337	5.757184	-0.80577	-0.59222	0.000278	0.402242	Over 10 ye	7
0.000498	0.000841	8.941735	0.823845	-0.56681	0.013135	0.383289	2 to 5 year	2
0.001105	0.000415	5.860742	-0.63306	-0.7741	0.000107	0.404435	5 to 10 yea	7
0.001133	0.000546	6.975701	-0.65862	0.752475	0.004293	0.399878	5 to 10 yea	4
0.000265	-0.00173	8.467335	0.624563	-0.78097	0.032531	0.341907	2 to 5 year	9
-0.00037	0.000636	6.427949	-0.7724	-0.63513	0.003948	0.402743	Over 10 ye	11
-0.00069	-0.00049	8.958983	0.132123	-0.99123	0.044799	0.359558	Over 10 ye	34
0.000971	0.00096	6.294988	0.703294	-0.7109	0.039269	0.364845	5 to 10 yea	2
-0.00069	-0.00049	8.958983	0.132123	-0.99123	0.044799	0.359558	Over 10 ye	34
0.00033	0.000413	7.68102	0.622847	0.782343	0.01425	0.411578	Over 10 ye	14
-0.00028	0.000453	6.854851	0.797983	0.60268	0.009312	0.395292	2 to 5 year	16

Table S2 - Anonymized database

-0.00018	0.000577	7.314416	0.452999	0.891511	0.078283	0.307638	5 to 10 yea	11
-3.90E-05	-0.00066	10.38832	-0.13929	-0.99025	0.046155	0.323061	Over 10 ye	15
-0.00029	-6.10E-05	10.92574	0.009512	-0.99995	0.040339	0.342214	2 to 5 year	8
-0.00354	-0.0001	7.882977	0.95887	-0.28384	0.000103	0.423653	1 to 2 year	12
-0.00276	0.000517	8.721258	-0.29706	-0.95486	0.057471	0.335248	2 to 5 year	3
-0.00069	-0.00049	8.958983	0.132123	-0.99123	0.044799	0.359558	Over 10 ye	34
0.000708	0.000582	7.037695	0.990504	0.137486	0.014558	0.400028	Over 10 ye	7
3.30E-05	0.000103	7.984262	0.497598	-0.86741	0.004843	0.391127	5 to 10 yea	11
0.00018	-0.00015	9.943292	0.995409	-0.09571	0.010153	0.375219	2 to 5 year	2
0.000242	-4.50E-05	7.396961	-0.96834	0.249643	0.024769	0.391333	1 to 2 year	3
-0.00045	-0.0004	9.139278	-0.97586	-0.21837	0.009431	0.390315	2 to 5 year	20
-0.00045	-0.0004	9.139278	-0.97586	-0.21837	0.009431	0.390315	2 to 5 year	20
-0.00043	0.000222	6.91947	-0.59886	-0.80085	0.003814	0.39002	2 to 5 year	4
-0.00069	-0.00049	8.958983	0.132123	-0.99123	0.044799	0.359558	Over 10 ye	34
-3.90E-05	-0.00066	10.38832	-0.13929	-0.99025	0.046155	0.323061	Over 10 ye	15
0.000476	-0.0004	9.722341	0.966119	0.258098	0.036207	0.34751	5 to 10 yea	3
0.001105	0.000415	5.860742	-0.63306	-0.7741	0.000107	0.404435	5 to 10 yea	7
-0.00054	-0.00053	7.974645	0.37949	-0.9252	0.003664	0.395188	Over 10 ye	16
-0.00045	-0.0004	9.139278	-0.97586	-0.21837	0.009431	0.390315	2 to 5 year	20
-0.00028	0.000453	6.854851	0.797983	0.60268	0.009312	0.395292	2 to 5 year	16
-0.00031	0.000414	6.752109	-0.48257	-0.87585	0.000372	0.396149	2 to 5 year	4
-4.00E-05	0.000166	6.602221	-0.24178	0.97033	0.042414	0.328016	2 to 5 year	5
3.00E-06	-0.00061	12.34398	0.695892	0.718146	3.18E-05	0.406127	2 to 5 year	3
0.000705	0.000384	6.483116	-0.75141	0.659836	0.016717	0.333957	Over 10 ye	9
0.002444	-0.00025	6.068515	-0.99992	-0.01246	0.00288	0.395734	2 to 5 year	4
0.000404	-0.00101	6.550444	-0.98501	0.172514	0.000392	0.415925	5 to 10 yea	9
-0.00126	0.000191	7.233391	0.378898	0.925438	0.008101	0.393838	1 to 2 year	6
0.002301	0.003581	5.215646	0.603308	0.797508	0.01096	0.389974	1 to 2 year	5
-0.0006	0.001251	7.111355	-0.90249	0.430702	0.002676	0.421914	5 to 10 yea	7
-0.00054	-0.00053	7.974645	0.37949	-0.9252	0.003664	0.395188	Over 10 ye	16
0.000112	0.000241	7.972994	0.449417	0.893322	0.002402	0.419275	2 to 5 year	7
-0.00351	-0.00493	9.698227	0.9886	-0.15057	0.05117	0.320754	5 to 10 yea	10
-0.00249	-0.00183	7.791164	0.833364	0.552724	0.000654	0.433276	5 to 10 yea	6
2.20E-05	-0.00026	8.60884	0.084634	-0.99641	1.55E-05	0.411413	2 to 5 year	9
0.001535	0.000484	6.717156	-0.14995	-0.98869	0.058258	0.334687	1 to 2 year	2
-7.20E-05	-4.70E-05	11.12294	0.04965	0.998767	0.103332	0.286539	Over 10 ye	12
3.70E-05	0.000344	6.792089	-0.99023	0.139451	0.012965	0.387951	2 to 5 year	12
0.002139	-0.00178	6.95804	-0.13969	0.990196	0.009714	0.385306	1 to 2 year	7
0.000125	0.000106	8.09158	-0.47147	-0.88188	0.001293	0.395439	5 to 10 yea	12
-0.00045	-0.0004	9.139278	-0.97586	-0.21837	0.009431	0.390315	2 to 5 year	20
-0.00108	-0.00118	9.110424	0.81459	-0.58004	0.043763	0.373741	2 to 5 year	24
-0.00018	0.000577	7.314416	0.452999	0.891511	0.078283	0.307638	5 to 10 yea	11
0.000382	-0.00059	12.14705	0.290701	0.956814	0.001242	0.395773	1 to 2 year	11
-0.00354	-0.0001	7.882977	0.95887	-0.28384	0.000103	0.423653	1 to 2 year	12
0.0011	-0.00039	9.418569	0.243551	-0.96989	0.002535	0.408747	2 to 5 year	6
0.002295	0.00551	4.841191	-0.19558	0.980687	0.006205	0.387621	5 to 10 yea	10
-0.00028	-0.00062	10.31837	-0.92986	-0.3679	0.062812	0.32094	2 to 5 year	5
0.000453	0.000402	7.477843	0.861715	-0.50739	0.01583	0.397747	2 to 5 year	3
-0.00046	0.002523	5.318647	0.781001	0.62453	0.042118	0.349723	Over 10 ye	3
-0.00037	0.000395	7.243243	-0.01524	0.999884	0.037988	0.325697	2 to 5 year	6

Table S2 - Anonymized database

-0.00061	-0.00019	8.427426	-0.53394	0.845524	0.000184	0.415632	2 to 5 year	3
-0.00108	-0.00118	9.110424	0.81459	-0.58004	0.043763	0.373741	2 to 5 year	24
0.002565	0.000252	7.01588	-0.81028	-0.58604	0.008547	0.386603	5 to 10 yea	8
0.000404	-0.00101	6.550444	-0.98501	0.172514	0.000392	0.415925	5 to 10 yea	9
0.00033	0.000413	7.68102	0.622847	0.782343	0.01425	0.411578	Over 10 ye	14
-0.00354	-0.0001	7.882977	0.95887	-0.28384	0.000103	0.423653	1 to 2 year	12
-0.00108	-0.00118	9.110424	0.81459	-0.58004	0.043763	0.373741	2 to 5 year	24
0.00096	0.005301	5.114022	0.944001	-0.32994	4.06E-05	0.399017	5 to 10 yea	10
-0.00045	-0.0004	9.139278	-0.97586	-0.21837	0.009431	0.390315	2 to 5 year	20
0.004227	0.002337	5.757184	-0.80577	-0.59222	0.000278	0.402242	Over 10 ye	7
2.80E-05	0.000158	7.935713	0.986909	-0.16128	0.01295	0.403363	5 to 10 yea	6
3.30E-05	0.000103	7.984262	0.497598	-0.86741	0.004843	0.391127	5 to 10 yea	11
0.001447	0.000749	5.42723	0.013815	0.999905	0.000165	0.401891	Over 10 ye	5
3.70E-05	0.000344	6.792089	-0.99023	0.139451	0.012965	0.387951	2 to 5 year	12
-0.00019	0.000252	9.080857	-0.15098	0.988537	0.023393	0.371974	Over 10 ye	10
-7.20E-05	-4.70E-05	11.12294	0.04965	0.998767	0.103332	0.286539	Over 10 ye	12
0.002791	-0.00029	5.59797	0.994875	-0.10112	0.000414	0.426809	2 to 5 year	2
2.20E-05	-0.00026	8.60884	0.084634	-0.99641	1.55E-05	0.411413	2 to 5 year	9
0.003221	0.001054	5.788663	0.94556	0.325447	0.005833	0.395439	2 to 5 year	8
0.000125	0.000106	8.09158	-0.47147	-0.88188	0.001293	0.395439	5 to 10 yea	12
-3.90E-05	-0.00066	10.38832	-0.13929	-0.99025	0.046155	0.323061	Over 10 ye	15
0.005883	0.000297	5.461362	0.999997	-0.00237	0.000945	0.432409	2 to 5 year	1
-7.20E-05	-4.70E-05	11.12294	0.04965	0.998767	0.103332	0.286539	Over 10 ye	12
-0.00048	-0.00042	10.2381	-0.54978	0.835311	0.021104	0.374728	2 to 5 year	3
-0.00091	-0.00011	7.181785	0.96959	0.244737	0.003353	0.400542	2 to 5 year	5
-0.00069	-0.00049	8.958983	0.132123	-0.99123	0.044799	0.359558	Over 10 ye	34
-0.00108	-0.00118	9.110424	0.81459	-0.58004	0.043763	0.373741	2 to 5 year	24
0.000708	0.000582	7.037695	0.990504	0.137486	0.014558	0.400028	Over 10 ye	7
0.000242	-4.50E-05	7.396961	-0.96834	0.249643	0.024769	0.391333	1 to 2 year	3
-0.00011	-0.00023	10.92632	0.31408	-0.9494	0.064053	0.313069	Over 10 ye	5
-0.00045	-0.0004	9.139278	-0.97586	-0.21837	0.009431	0.390315	2 to 5 year	20
0.000125	0.000106	8.09158	-0.47147	-0.88188	0.001293	0.395439	5 to 10 yea	12
-0.00351	-0.00493	9.698227	0.9886	-0.15057	0.05117	0.320754	5 to 10 yea	10
0.002444	-0.00025	6.068515	-0.99992	-0.01246	0.00288	0.395734	2 to 5 year	4
0.000382	-0.00059	12.14705	0.290701	0.956814	0.001242	0.395773	1 to 2 year	11
-0.00013	0.000117	9.363936	-0.85537	-0.51802	0.068087	0.336834	2 to 5 year	5
-3.40E-05	-0.00087	9.206155	-0.75518	0.65552	0.021835	0.387149	5 to 10 yea	6
0.001468	-3.60E-05	6.365498	0.172739	0.984968	0.056563	0.311263	1 to 2 year	2
-0.00108	-0.00118	9.110424	0.81459	-0.58004	0.043763	0.373741	2 to 5 year	24
-0.00018	0.000166	9.809126	-0.98811	-0.15374	0.017034	0.383016	2 to 5 year	4
0.000382	-0.00059	12.14705	0.290701	0.956814	0.001242	0.395773	1 to 2 year	11
-0.00028	0.000453	6.854851	0.797983	0.60268	0.009312	0.395292	2 to 5 year	16
0.002565	0.000252	7.01588	-0.81028	-0.58604	0.008547	0.386603	5 to 10 yea	8
2.20E-05	-0.00026	8.60884	0.084634	-0.99641	1.55E-05	0.411413	2 to 5 year	9
0.000106	0.000435	10.30726	-0.07783	0.996966	0.000332	0.395629	5 to 10 yea	4
0.000108	1.90E-05	10.09791	0.863383	0.50455	0.003253	0.431537	2 to 5 year	3
8.00E-06	-1.60E-05	13.27315	0.748272	-0.66339	8.31E-07	0.311308	2 to 5 year	3
2.20E-05	-0.00026	8.60884	0.084634	-0.99641	1.55E-05	0.411413	2 to 5 year	9
-0.00171	0.000814	6.917393	0.837894	-0.54583	0.061167	0.312614	1 to 2 year	2
-0.00023	0.000294	7.396209	-0.85928	0.511503	0.102343	0.287067	5 to 10 yea	13

Table S2 - Anonymized database

0.001131	0.00063	7.54068	0.908182	-0.41858	0.339062	0.343287	5 to 10 yea	5
0.002139	-0.00178	6.95804	-0.13969	0.990196	0.009714	0.385306	1 to 2 year	7
-0.00069	-0.00049	8.958983	0.132123	-0.99123	0.044799	0.359558	Over 10 ye	34
8.70E-05	2.70E-05	6.958606	-0.76012	-0.64978	0.003537	0.38875	Over 10 ye	3
0.002301	0.003581	5.215646	0.603308	0.797508	0.01096	0.389974	1 to 2 year	5
8.50E-05	-0.00011	8.182613	0.65839	-0.75268	0.00036	0.432406	2 to 5 year	5
-0.00054	-0.00053	7.974645	0.37949	-0.9252	0.003664	0.395188	Over 10 ye	16
0.0011	-0.00039	9.418569	0.243551	-0.96989	0.002535	0.408747	2 to 5 year	6
-0.00063	-6.20E-05	8.821591	-0.96326	-0.26858	0.050648	0.295265	2 to 5 year	7
-0.00126	0.000191	7.233391	0.378898	0.925438	0.008101	0.393838	1 to 2 year	6
0.000384	0.000594	7.174697	-0.35256	0.935788	0.042672	0.359447	2 to 5 year	5
-0.00063	-6.20E-05	8.821591	-0.96326	-0.26858	0.050648	0.295265	2 to 5 year	7
0.003221	0.001054	5.788663	0.94556	0.325447	0.005833	0.395439	2 to 5 year	8
-0.00181	-3.80E-05	11.36418	-0.63714	0.770751	0.001584	0.411519	5 to 10 yea	5
0.000332	0.001614	5.844646	0.999888	0.014955	0.022942	0.378116	Over 10 ye	4
-0.00054	-0.00053	7.974645	0.37949	-0.9252	0.003664	0.395188	Over 10 ye	16
0.000404	-0.00101	6.550444	-0.98501	0.172514	0.000392	0.415925	5 to 10 yea	9
-0.00201	6.60E-05	14.15868	0.97946	-0.20164	0.020628	0.384548	2 to 5 year	3
-0.00354	-0.0001	7.882977	0.95887	-0.28384	0.000103	0.423653	1 to 2 year	12
0.000928	0.000315	7.07586	-0.01795	-0.99984	0.047869	0.34643	5 to 10 yea	26
-0.00063	-6.20E-05	8.821591	-0.96326	-0.26858	0.050648	0.295265	2 to 5 year	7
-0.00048	-0.00042	8.983812	-0.95159	0.307365	0.001385	0.40293	2 to 5 year	5
8.70E-05	2.70E-05	6.958606	-0.76012	-0.64978	0.003537	0.38875	Over 10 ye	3
0.000705	0.000384	6.483116	-0.75141	0.659836	0.016717	0.333957	Over 10 ye	9
0.002565	0.000252	7.01588	-0.81028	-0.58604	0.008547	0.386603	5 to 10 yea	8
-0.00029	-6.10E-05	10.92574	0.009512	-0.99995	0.040339	0.342214	2 to 5 year	8
0.000265	-0.00173	8.467335	0.624563	-0.78097	0.032531	0.341907	2 to 5 year	9
2.20E-05	-0.00026	8.60884	0.084634	-0.99641	1.55E-05	0.411413	2 to 5 year	9
-0.00317	-0.00758	8.285575	0.381979	-0.92417	0.008867	0.385166	Over 10 ye	17
0.001547	0.000517	6.168585	0.008704	0.999962	0.001499	0.425114	5 to 10 yea	3
